# Infection prevention and control measures for Ebola and Marburg disease: a series of rapid reviews

**DOI:** 10.1136/bmjopen-2025-115610

**Published:** 2026-07-09

**Authors:** Nicole Shaver, Ba Pham, Niyati Vyas, Erin Collins, Alexandria Bennett, Andrew Beck, Becky Skidmore, Maura R Grossman, Gordon V Cormack, Sharmistha Mishra, Adrienne K Chan, David Moher, Melissa Brouwers, Andrea C Tricco, Victoria Willet, Devika Dixit, April Baller, Julian Little

**Affiliations:** 1 Knowledge Synthesis and Application Unit, School of Epidemiology and Public Health, University of Ottawa, Ottawa, Ontario, Canada; 2 Institute of Health Policy Management and Evaluation, University of Toronto, Toronto, Ontario, Canada; 3 Western University, London, UK, Canada; 4 Independent Information Specialist, Ottawa, Ontario, Canada; 5 School of Computer Science and School of Public Health Sciences, University of Waterloo, Waterloo, Ontario, Canada; 6 Osgoode Hall Law School, York University, Toronto, Ontario, Canada; 7 School of Computer Science, University of Waterloo, Waterloo, Ontario, Canada; 8 Department of Medicine, St. Michael’s Hospital, University of Toronto, Toronto, Ontario, Canada; 9 MAP Centre for Urban Health Solutions, Li Ka Shing Knowledge Institute, St Michael’s Hospital, Toronto, Ontario, Canada; 10 Sunnybrook Health Sciences Centre, Toronto, Ontario, Canada; 11 Dalla Lana School of Public Health, University of Toronto, Toronto, Ontario, Canada; 12 Ottawa Hospital Research Institute Ottawa Methods Centre, Ottawa, Ontario, Canada; 13 Li Ka Shing Knowledge Institute, St Michael’s Hospital, Toronto, Ontario, Canada; 14 Epidemiology Division and Institute of Health Policy, Management, and Evaluation, Dalla Lana School of Public Health, University of Toronto, Toronto, Ontario, Canada; 15 Infection Prevention & Control, WHO Health Emergencies (WHE) Programme, World Health Organization, Geneva, Switzerland; 16 Cumming School of Medicine, University of Calgary, Alberta, Edmonton, Canada

**Keywords:** Review, Systematic Review, Disease Outbreaks, Infection control, Disease Management, EPIDEMIOLOGY

## Abstract

**Abstract:**

**Objectives:**

An evidence synthesis informed the 2023 WHO infection prevention and control (IPC) guideline for Ebola disease (EBOD) and Marburg disease (MARD).

**Design:**

A series of rapid reviews addressed 14 key questions (KQs) related to IPC measures for EBOD and MARD across three themes: (1) transmission/exposure, (2) personal protective equipment (PPE) and (3) decontamination/disinfection.

**Data sources:**

Databases (Embase, Cochrane Database of Systematic Reviews, CENTRAL, Global Index Medicus) were searched from inception to May 2024, in addition to searches in MEDLINE and bio/medRxiv servers through an artificial intelligence tool (continuous active learning, CAL).

**Eligibility criteria:**

Eligible populations included health workers (HWs), staff and patients in healthcare and/or community settings. Interventions included PPE evaluation, hand hygiene/glove decontamination, spraying of HWs, handling of heavily soiled linen, management of the deceased, environmental decontamination, work exclusion and the IPC ring approach. Infection with EBOD or MARD was a primary outcome of interest and secondary outcomes of interest varied by key question.

**Data extraction and synthesis:**

Screening, data extraction, quality assessment (Cochrane RoB-2, Risk Of Bias In Non-Randomized Studies - of Interventions (ROBINS-I)) and evaluation of the certainty of evidence (Grading of Recommendations Assessment, Development and Evaluation (GRADE)) were conducted by an independent reviewer with verification. Data and relevant contextual information were narratively synthesised.

**Results:**

Following screening of 5540 studies in CAL and 1062 full texts, nine studies were included that addressed four KQs: one related to transmission/exposure (IPC ring approach), two related to PPE (head/neck skin and mucous membranes coverage; order of eye protection and head covering) and one related to disinfection/decontamination (spraying of HWs during PPE doffing). No studies were included for the remaining KQs, but relevant contextual information in reviewed studies was summarised for all KQs. Evidence was judged to be of low or very low certainty using the GRADE framework due to risk of bias, indirectness and imprecision, and substantive conclusions could not be drawn on the effectiveness of the evaluated IPC interventions.

**Conclusions:**

While providing the evidence base for the WHO IPC guideline for EBOD and MARD, this series of rapid reviews identified several critical research gaps.

**PROSPERO registration number:**

CRD#42022331537.

**STRENGTHS AND LIMITATIONS OF THIS STUDY**

- This series of rapid reviews integrated evidence across multiple infection prevention and control (IPC) domains to inform the WHO IPC guideline for Ebola and Marburg disease (August 2023).
- The reviews followed an a priori protocol, standardised methods and reporting frameworks (ie, Preferred Reporting Items for Systematic Reviews and Meta-Analyses (PRISMA) and Grading of Recommendations Assessment, Development and Evaluation) to support transparency and reproducibility.
- Several key questions resulted in empty or near-empty reviews, reflecting both the sparse primary evidence base and narrow eligibility criteria.
- An artificial intelligence-supported search and screening tool was used in these rapid reviews.
- Content expert verification and summaries of contextual data were used to mitigate the chance of missed studies.

## Background

 Ebola disease (EBOD) and Marburg disease (MARD) have posed significant challenges to global health. The largest Ebola virus (EBOV) outbreak to date occurred in West Africa in 2014–2016 and outbreaks continue to pose threats.^1^ Several EBOD outbreaks were reported in 2022,^1 2^ and two confirmed MARD outbreaks occurred in Equatorial Guinea and Tanzania in 2023.^3^ In 2024, the largest Marburg virus outbreak to date was reported in Rwanda^4^ and as of 2026, there is an ongoing outbreak of EBOD caused by *Bundibugyo* virus in remote areas of the Democratic Republic of the Congo and Uganda that is a public health emergency of international concern.^5^ EBOD and MARD patients present to and require inpatient care in healthcare facilities, some of which have experienced nosocomial transmission in past outbreaks.^6^ There is a need to review evidence on effective infection prevention and control (IPC) measures, particularly for health workers (HWs) on the front lines.

EBOD and MARD are caused by species of viruses belonging to the *Filoviridae* family that are pathogenic to humans, *orthoebolaviruses* (*Orthoebolavirus sudanense* virus name Sudan virus*, Orthoebolavirus taiense* virus name Taï Forest virus*, Orthoebolavirus zairense* virus name EBOV and *Orthoebolavirus bundibugyoense* virus name Bundibugyo virus) and *orthomarburgviruses* (*Marburg and Ravn*), respectively.^7 8^ Both diseases have similar clinical presentations, high case-fatality rates and modes of transmission.^9^ The natural hosts of these filoviruses are not definitively known, although fruit bats are suspected to play a role in their spread.^9^ Transmission to humans occurs through contact with infected animals (zoonotic transmission) or via human-to-human transmission through direct or indirect contact with body fluids, contaminated medical equipment or environmental surfaces.^10^

HWs caring for patients infected with filoviruses can potentially face elevated or repeated exposures to these viruses, thereby resulting in increased transmission risks due to their proximity and frequent contact with patients and contaminated materials.^6 10^ Compounding these risks, outbreaks have often occurred in low-resource settings. A 2018 review found that insufficient or incorrect use of personal protective equipment (PPE) was the most frequently cited exposure risk for HWs.^6^ Transmission risks may be particularly high for traditional/community healers, who are the first point of healthcare in many rural and urban regions across countries in sub-Saharan Africa, but who may not have access to or training in the use of PPE or other resources to safely interact with or treat those infected.^11 12^

The clinical severity of EBOD and MARD and the ongoing risks of spillover into the human population with the potential for human-to-human transmission through blood and body fluids and environmental/contaminated materials emphasise the importance of effective IPC measures to facilitate the provision of safe care for patients and HWs. New practices have emerged since the WHO released their 2016 interim guidance on PPE for filovirus haemorrhagic fever outbreaks in healthcare settings.^13^ Additionally, new evidence on IPC practices has emerged since the 2014 rapid review^14^ that informed the 2016 guideline recommendations. The 2014 rapid review identified a lack of comparative evidence on PPE effectiveness for HWs caring for EBOD and MARD patients, preventing definitive conclusions.^14^ Additionally, gaps in the understanding of EBOD and MARD transmission, addressed in a separate scoping review,^15^ further informed the need for updated rapid reviews and IPC guidance. These evidence gaps necessitated updated evidence-based recommendations.

### Rationale and objectives

This series of rapid reviews aimed to synthesise the latest evidence used to inform the WHO Health Emergencies Programme *Infection Prevention and Control Guideline for Ebola and Marburg Disease*.^15^ The rapid reviews addressed 14 key questions (KQs) developed by the WHO Health Emergencies Programme, Country Readiness Strengthening Unit, IPC and WASH (water, sanitation and hygiene) team (table 1). While the reviews focused on IPC in healthcare facilities, evidence in community settings was also considered for the majority of KQs, as these care settings often overlap/blur in emergency scenarios.

**Table 1:** Key questions (KQs) by theme

Theme	Study design
Transmission/exposure (n=3 questions)	(KQ1) Should health workers who have had Ebola Virus Disease (EVD) exposure other than high-risk be excluded versus not excluded from work?
	(KQ2) Should bodies of patients deceased from Ebola or Marburg disease be disinfected versus not disinfected prior to handling/moving into a body bag?
	(KQ3) Should the IPC ring approach* be used versus not used to prevent and control transmission of EVD in healthcare facility and community settings?
Personal protective equipment (PPE) (n=7 questions)	(KQ4) Should health workers conducting EVD-related screening and triage activities wear a face shield alone versus in combination with a medical (non-structured) mask?
	(KQ5) Should health workers in direct contact and/or indirect contact with patients with EVD or Marburg virus disease cover head and neck skin and mucous membranes or just cover mucous membranes?
	(KQ6) Should health workers providing direct care or indirect** care to patients with EBOD or Marburg virus disease and using eye protection (goggles/face shield) wear them under versus over the head and neck covering?
	(KQ7a) Should health workers conducting Ebola or Marburg virus-related screening activities wear a gown versus wear a coverall? (KQ7b) Should health workers conducting Ebola or Marburg virus-related triage activities wear a gown versus wear a coverall?
	(KQ8) Should health workers using waterproof aprons to cover gowns or coveralls while providing direct or indirect care to patients with Ebola or Marburg virus disease, use disposable versus reusable versus biodegradable types of aprons?
	Additional PICO 1: Should health workers conducting screening where at least 1 m distance and a no touch technique can be maintained and PPE (such as gown and facial protection) is not expected wear no gloves and perform hand hygiene versus wear 1 pair of gloves?
	Additional PICO 2: Should health workers conducting screening and/or triage activities where at least 1 m distance cannot be maintained and PPE (such as gown and facial protection) is expected, wear 2 pairs of gloves versus 1 pair of gloves?
Decontamination and disinfection (n=4 questions)	(KQ9) Should surfaces and materials in healthcare facilities, Ebola treatment units (ETUs) and community settings providing care to patients with Ebola or Marburg disease be disinfected using a wiping method versus a spraying method?
	(KQ10) Should health workers providing direct or indirect care to patients with Ebola or Marburg disease be sprayed versus not sprayed during doffing of personal protective equipment?
	(KQ11a) Should health workers providing direct or indirect care to patients with Ebola or Marburg disease in ETUs and healthcare facilities wash hands (soap and water) or wash the glove (soap and water) between patients? (KQ11b) Should health workers providing direct or indirect care to patients with Ebola or Marburg disease in ETUs and healthcare facilities disinfect hands with ABHR or disinfect the glove with ABHR between patients? (KQ11c) Should health workers providing direct or indirect care to patients with Ebola or Marburg disease in ETUs and healthcare facilities disinfect hands (with chlorine) or disinfect the glove (with chlorine) between patients?
	(KQ12) Should heavily soiled linen resulting from care to patients with Ebola or Marburg in health care, ETUs or community settings be incinerated versus disinfected? Risks related to staff/person handling the linens (washing manually or by machine wash)

*The ring IPC approach rapidly mobilizes teams to assist affected health facilities and the community in implementing IPC measures to reduce Ebola transmission in a predetermined risk area whenever a case is identified.

**Indirect care (not patient-facing in the red zone) could include all activities in the green zone including charting, care provider team meetings, handover of clinical care plans, preparing medications, preparing and/or packaging food for transfer into the ETU and calling and/or discussing with family members and caregivers of patients.

ABHR, Alcohol-Based Hand Rub; EBOD, Ebola disease; EVD, Ebola Virus Disease; IPC, infection prevention control; PICO, Population, Intervention, Comparator, Outcome.

## Methods

### Review approach

The 14 KQs were organised into three themes: (1) transmission/exposure, (2) PPE and (3) decontamination and disinfection (table 1). A planned staged approach was undertaken to address the KQs by first identifying a relevant systematic review or knowledge synthesis to update. However, as no high-quality, recent systematic reviews were identified on any of the KQs, de novo rapid reviews of comparative primary studies were carried out.

### Collaborator and Patient and public involvement

A core team consisting of the WHO Health Emergencies Programme, Country Readiness Strengthening Unit, IPC and WASH team members, and methodologists formulated the KQs and PICOS (Population, Interventions, Comparators, Outcomes and Setting/Study design) for each KQ with involvement from the evidence review team. The eligibility criteria for the search strategy for each KQ were also reviewed by a methodologist and the Guideline Development Group (GDG) and steering committee members before finalisation. The steering committee included internal WHO members from different programmes with varying areas of expertise. The GDG consisted of independent researchers, clinical experts, representatives from organisations such as the US Centers for Disease Control and Prevention (CDC), Center for Disease Control-Kenya and Nigeria Centre for Disease Control and Prevention, MSF (Médecins Sans Frontières), and a patient representative with lived experience of EBOD. The WHO core team, steering group and GDG were not involved in the conduct of the review, including the selection of studies and data analysis, but reviewed the draft report and provided input on interpretations of findings. The final data and results of the literature reviews were presented to the GDG to formulate the guideline recommendations.

### Registration and reporting

As there is no reporting guideline for rapid reviews, this series of reviews was informed through the Preferred Reporting Items for Systematic Reviews and Meta-Analyses (PRISMA) 2020 statement (online supplemental file 1).^16^ Rapid review methods were informed by the WHO handbook for guideline development.^17^ A protocol for the rapid reviews (informed by PRISMA-Protocols (PRISMA-P)^18^) was publicly registered in PROSPERO (CRD#42022331537) before review commencement. The protocol and other review materials are available on the Open Science Framework (https://osf.io/8raby/).^19^ Deviations from the initial protocol included the addition of two PICO questions requested by the WHO beyond the 12 KQs listed in the protocol (additional PICO 1 and additional PICO 2). The original PICO questions and protocol were written using the terms ‘EVD’ and ‘MVD’ for Ebola Virus Disease and Marburg Virus Disease, respectively. However, a nomenclature change to ‘EBOD’ and ‘MARD’, respectively, occurred during the writing of the guideline, and therefore, this nomenclature is used in the final WHO guideline and the present manuscript.^20^ The original abbreviations were not used in the search strategy, so this change should not have influenced any aspect of the search or study selection.

### Data sources and search strategy

A patented machine learning algorithm, the continuous active learning (CAL) tool was used to accelerate searching and title/abstract screening. The CAL tool was selected based on two primary considerations: our teams have extensive prior experience implementing the tool in evidence synthesis projects, and it is a validated, high-recall system^21^,^23^ with a strong track record in systematic review contexts.^24^ The tool uses a supervised machine learning approach (CAL) in which a logistic regression algorithm uses relevance decisions made by a reviewer to continuously re-rank the remaining unscreened literature from most to least likely relevant, presenting the reviewer with the next most likely relevant study at each step. Stopping criteria for CAL screening are described in the Study Selection section below. Multiple screening sessions using different seed studies (ie, starting articles) were conducted for each KQ to reduce the risk of algorithmic bias and support high recall. While artificial intelligence (AI)-assisted prioritisation helped to accelerate searching and title/abstract screening, all final inclusion and exclusion decisions at the full-text stage were made by human reviewers.

MEDLINE and bio/medRxiv pre-print servers were searched using the CAL tool. Searches were performed with no date limits until 12 February 2022. Additionally, using the Ovid platform, Cochrane Database of Systematic Reviews, CENTRAL and Embase were searched on 12 February 2022, as well as Global Index Medicus. The database searches using the same terms were updated on 1 May 2024. The electronic database search strategies were developed and tested by an experienced medical information specialist (BS) in consultation with the review team (online supplemental file 2). The Embase search strategy was peer-reviewed by another librarian using the Peer Review of Electronic Search Strategies checklist^25^ (online supplemental file 3). The search included all languages and dissemination modalities (eg, websites, publications, letters to the editor). Where possible, CAL searches for KQs with similar PICO criteria were combined for efficiency (online supplemental file 5).

### Study selection

Results from the literature search conducted outside of the CAL tool were uploaded into the tool for a single database for searching/screening. Following a calibration exercise among the review team, titles and abstracts were screened by one reviewer within the CAL tool. Given the continuous re-ranking nature of the CAL algorithm (where the most relevant articles are presented first), screening continued until 100 sequential references were excluded or there was consensus among the review team that no further relevant studies were being identified, consistent with recommended practice for active learning-based tools. Studies identified as relevant after titles/abstract screening were manually de-duplicated and uploaded into the Distiller SR tool for full-text screening.^26^ After a pilot exercise, full-text articles were independently screened by one reviewer with verification by a second reviewer. As an additional measure to ensure comprehensiveness of literature capture, the included reference lists of relevant systematic reviews identified during CAL searching were screened. Articles that were unavailable electronically were ordered via interlibrary loan and excluded if not received within 14 days due to time constraints.

### Eligibility criteria

Detailed eligibility criteria are presented in online supplemental file 4. In brief, populations of interest varied by KQ and included HWs, burial teams and/or patients in healthcare facilities, Ebola treatment units and/or community settings. Eligible interventions included a broad range of IPC supplies and practices across three themes: (1) transmission and exposure, including exclusion of exposed HWs from work and the IPC ring approach; (2) PPE, including head and neck coverage, eye protection positioning, gown versus coverall use, disposable versus reusable aprons and single versus double gloving and (3) decontamination and disinfection, including environmental disinfection methods, spraying of HWs during PPE doffing, hand hygiene and glove decontamination approaches, and handling of heavily soiled linen. Where applicable, comparators included alternative IPC configurations or the use of standard precautions. The primary outcome of interest was infection with EBOD or MARD, while secondary outcomes of interest varied depending on the KQ. For PPE-related questions, these outcomes included HW confidence, PPE comfort, PPE breaches and human factors. Eligible study designs were comparative primary studies, including randomised controlled trials (RCTs), cohort studies and nested case–control designs. Simulation studies were eligible if the overarching study design was comparative. In the absence of comparative evidence for a high-priority KQ, the WHO Core Team and evidence review team jointly considered whether a non-comparative study that met all other eligibility criteria should be included. While no language restrictions were applied at the search stage, full text review was restricted to English-language studies due to resource limitations in accessing translation services within the rapid review timeframe. Studies screened at full text that did not meet the eligibility criteria but provided additional KQ-relevant information related to equity, feasibility, acceptability or the implementation of interventions were summarised as ‘contextual information’. Contextual information was not summarised for studies reviewed at the search update phase due to resource constraints.

### Data extraction and risk of bias assessment

Both data extraction and risk of bias assessments were completed by one reviewer, and the information was verified by a second reviewer, with disagreements resolved by consensus. No pilot testing was completed due to the small number of included studies for each KQ. Standardised data extraction forms were developed a priori in Microsoft Excel. Items of interest included patient characteristics (eg, infection with EBOD or MARD, age, country of residence), study characteristics (eg, study design, funding, country of conduct), intervention details and outcome results. For RCTs, the Cochrane Risk of Bias 2 Tool for RCTs was used.^27^ For non-randomised simulation studies, the ROBINS-I tool was used.^28^ Given the lack of validated instruments for non-comparative study designs,^29^ risk of bias for our outbreak study was assessed using a modified approach informed by established domains from the Newcastle-Ottawa Scale and ROBINS-I. Studies were evaluated for: demonstration that the outcome of interest was absent at study initiation and completeness of outcome follow-up. The absence of a comparator group across these studies was noted as a uniform limitation of the evidence base.

### Synthesis of included studies

Participant and study characteristics, quality and key findings for each KQ were summarised descriptively. To judge if pooling of studies was appropriate for a single outcome, we first summarised the characteristics of each included study and compared/contrasted each element of the PICO criteria to estimate the level of clinical heterogeneity. Due to the small number of studies and high degree of heterogeneity in the outcomes included for each KQ, pooling of the studies was deemed inappropriate, and we did not perform any subgroup analyses or meta-regression analysis. For each outcome, the effect magnitudes, statistical significance (as reported by the study), and 95% CIs were presented. For binary outcomes, relative risks and 95% CI and the associated absolute effects per 1000 participants were calculated following Cochrane guidance.^30^ Relative risks and CIs were not calculated in cases where no events occurred. For continuous outcomes, mean differences and 95% CIs were calculated using standard frequentist statistics.^30^ CIs were not estimated for differences in medians. For crossover studies, 95% CIs were calculated using standard frequentist statistics without adjusting for the crossover design, due to the lack of raw paired data from the original studies. While this may have underestimated variance to increase apparent precision, this approach was applied cautiously to provide directional insights and support the interpretation of results in a standardised Grading the Certainty of Evidence and Interpretation (Grading of Recommendations Assessment, Development and Evaluation, GRADE) format.

### Grading the certainty of evidence and interpretation

Evidence for KQs was assessed using the GRADE framework.^31^ One reviewer appraised the certainty of the evidence, with verification by a second reviewer. The GRADEpro Guideline Development Tool online software was used to produce evidence profiles.^32^ For each outcome, four domains were assessed (risk of bias, indirectness, inconsistency, imprecision). Publication bias was not assessed due to an insufficient number of studies included for each outcome.^33^ As per GRADE recommendations, the overall certainty of evidence for RCTs was started at ‘high’ certainty of evidence and then downrated as appropriate for each domain and non-randomised studies began at ‘low’ certainty of evidence.^31^ Footnotes explained the grading of each of the domains and the overall certainty of evidence. To inform imprecision ratings, the GRADE guidance for imprecision ratings (ie, a minimum of 400 events for dichotomous outcomes or 800 participants for continuous outcomes) was used, together with best judgement.^34^,^36^

## Results

### Search summary

A total of 5540 records were screened across 14 rapid review KQs using the CAL software. Full screening details for each KQ for the initial search and search update are documented via a PRISMA flow diagram (online supplemental file 5). A total of 1062 studies were screened at the full-text stage (see online supplemental file 6 for list of excluded studies with reasons). Overall, nine studies were included across three themes (one under transmission/exposure,^37^ six related to PPE^38^,^43^ and two related to disinfection/decontamination).^44 45^ No eligible studies to address the other 10 KQs were found. Relevant studies that did not meet the inclusion criteria yet provided contextual information were described (online supplemental file 7).

### Characteristics of included studies

A summary of study characteristics is presented in table 2. Two studies were based on real-world observational evidence: one non-comparative outbreak investigation conducted during the 2014–2016 West African EBOD outbreak^37^ and one cross-sectional comparative study conducted in the same context.^44^ The remaining studies consisted of four non-randomised simulation studies^38^,^4045^ and three crossover RCTs,^41^,^43^ all performed under simulated or laboratory conditions. No included studies provided information specific to MARD transmission. Simulation studies evaluated PPE for highly infectious disease/filovirus outbreaks (EBOD or MARD) in simulated ambient conditions for West African countries or controlled laboratory environments.

**Table 2:** Characteristics of studies relating to IPC for EBOD and MARD

Citation (author, year)	Study design	Outbreak, virus species	Study setting	# Total health workers (HWs)	# healthcare facilities	Description of intervention/exposure	Description of HW contact/care with patients
Casalino, *et al* 2015^38^	Non-randomised simulation study	N/A	Location that simulated a hospital room and an external area comprising a dirty zone and a clean zone	120 students (30 in each group)	N/A; courses held in Paris, France, Lima, Peru and Guadalajara, Mexico	A 60-minute theoretical course covering EBOD epidemiology and PPE donning/doffing, followed by a practical course comprising 2 distinct training methods: a conventional training programme and a reinforced training programme for basic and enhanced PPE ensembles.	N/A
Casanova, *et al *2016^45^	Non-randomised simulation study	N/A, mixture of MS2 (non-enveloped virus surrogate) and Φ6 (enveloped virus surrogate)	Patient room in a large tertiary care academic medical centre	15 HWs from an Ebola care team (11 RNs and 4 MDs)^*^	1	Participants were assigned to extra glove sanitisation through spraying of the hands with hypochlorite solution or use of an ABHR	Mixture of virus surrogate applied to four PPE sites on HWs to simulate contamination through droplet exposure during patient care^†^
Chughtai, *et al *2018^42^	Crossover randomised controlled trial	N/A, fluorescent solution^‡^ on the PPE surface to simulate Ebola virus	Healthcare simulation room	10 participants	N/A; one simulation room	Ten different donning/doffing protocols by various health organisation to protect from EBOD were tested.	Fluorescent lotion applied on external PPE to simulate contamination and sprayed (1 m) to mimic droplet infection
Coca, *et al* 2017^39^	Non-randomised simulation study	N/A	Simulated ambient conditions for West African countries^§^	6 healthy individuals to simulate HWs	N/A; one environmental chamber	Three different PPEs (E1, E2 and E3): All subjects wore medical scrubs and PPE items. E1 also had a face shield and fluid-resistant surgical gown; E2 additionally included goggles, coverall and separate hood; and E3 also contained a highly impermeable coverall, separate hood, and surgical mask cover over the N95 respirator.	Exercise intensity was set to the average for nursing care^¶^
Coca, *et al* 2015^40^	Non-randomised simulation study	N/A	Simulated ambient conditions for warmest months in West African countries^**^	N/A; sweating thermal manikins (each test replicated 3 times)	N/A; one environmental chamber	Four PPE ensembles similar to those recommended by the WHO and MSF at 2 different ambient conditions (32°C/92% relative humidity and 26°C/80% relative humidity) compared with a control ensemble.	Metabolic work rate (work intensity) was set to the average for nursing care^††^
Houlihan, *et al *2017^44^	Cross-sectional study	West Africa 2014–2016, Ebola	94% participants Sierra Leone, 4.5% Liberia, 1.1% Guinea	268 UK/Irish workers who responded to 2014–2016 West African Ebola epidemic^‡‡^	Not reported	The study collected information on the PPE used among HWs, including whether removal of PPE was performed with or without chlorine spray	Risk of Ebola virus disease exposure/transmission ranged from high risk (n=1, 0%) to very low (n=27, 10%) risk
Nyenswah, *et al *2015^51^	Outbreak investigation	Liberia, 2015 outbreak, Ebola	Health facility	166 HWs exposed in St. Paul Bridge Cluster	59 HCFs across 4 IPC rings (10 HCFs in St. Paul Bridge Cluster)	Rapid IPC needs assessments focused on triage procedures and personal protective equipment use. Following assessment, PPE distribution, general IPC training and specialised triage training.	HW exposure varied. Possible exposures included providing treatment or performing laboratory tests on blood specimens of Ebola patients, or cleaning a room/mattress where an Ebola patient had slept
Suen, *et al* 2018^43^	Crossover randomised controlled trial	N/A, fluorescent solution^§§^ on the PPE surface to simulate Ebola virus	Air-conditioned room with an average temperature of 23°C±2°C and a relative humidity of 60%±3%	59 HWs (all evaluated in each of PPE ensembles)	N/A; one air-conditioned room	Donning and doffing of three PPEs: Hospital Authority (HA) Standard Ebola PPE set (PPE1), Dupont Tyvek Model, style 1422A (PPE2), and HA isolation gown for routine patient care and performing aerosol-generating procedures (PPE3)	Fluorescent solution sprayed on PPE at the length of a stethoscope to simulate usual working distance between a patient and an HW^¶¶^; contamination events during doffing
Zamora, *et al* 2006^41^	Crossover randomised controlled trial	N/A, fluorescent solution^***^ on the PPE surface to simulate HID	Not reported	50 HWs	N/A	Donning and doffing of enhanced respiratory and contact precautions and PPE attire including a full body suit and a PAPR	Participants’ front face shield, torso, hands, forearms and elbows were contaminated with fluorescent solution/paste

* Members of the Ebola team were > 18 years of age and had undergone extensive training in a simulation laboratory in the use of EBOD-specific PPE, including donning and doffing.

† Mixture (25 µL in 5 drops of 5 µL) was applied to 4 sites: (1) the palm of the dominant hand, (2) the shoulder of the gown opposite the dominant hand, (3) the top side of the face shield on the same side as the dominant hand, and (4) the toe of the rubber boot opposite the dominant hand.

‡ GlitterBug. Glitterbug kits. Available from: https://glitterbug.net.au/products/

§ For each testing protocol, three periods with different conditions were simulated: 15-minute pre-exercise stabilization period (22°C, 50% relative humidity) and a 60-minute exercise period (32°C, 92% relative humidity), followed by a 30-minute recovery period in ambient conditions (22°C, 50% relative humidity).

¶ The exercise protocol consisted of 60 minutes of continuous walking, within an environmental chamber, on a treadmill at an intensity of three METs (2.5 mph, 0% grade). This exercise intensity was chosen to represent the working intensity seen in hospital nurses during patient care, such as walking, standing, and carrying light objects.

** Two conditions were simulated. Condition A consisted of 32°C, 92% relative humidity, Condition B consisted of 26°C, 80% relative humidity

†† Average work intensity for nursing corresponded to patient care that includes standing and walking slowly [2.5 mph] and carrying light objects [<11.3 kg]) of 3 METs (metabolic equivalent, or the measure of the intensity of aerobic exercise) over 80 min of continuous activity.

‡‡ Roles included clinical (physician/nurse), laboratory, research, as well as management/operations, trainer, epidemiologist, community engagement/tracing, WASH staff, finance, engineer, pharmacist, and social worker/burial team/information technology/journalist/visitor/logistician/nutritionist.

§§ UV GERM Hygiene Spray, Glow Tec Ltd., London, England

¶¶ Three strokes of fluorescent solution were sprayed onto the face shield, two upper limb/ gloves and anterior surfaces of the gown at a distance of 60 cm from the participants (total 12 strokes per case). There was an average of 1.99 g fluorescent solution/per stroke.

*** Fluorescein solution (1 mL of a 25% solution in 100 mL of sterile water). A Devilbiss atomizer (model DV15-RD, Sunrise Medical Products, Carlsbad, Calif.) was used to apply 5 mL of solution to each participant’s front face shield and torso. “Invisible” Detection Paste (15 mL; Sirchie, Youngsville, NC) was applied from the forearms to the elbow and to the palmar aspects of participants’ hands

ABHR, alcohol-based hand rub; EBOD, Ebola disease; HCF, healthcare facility; HID, highly infectious diseases; IPC, infection prevention and control; MARD, Marburg disease; MD, medical doctor; MSF, Médecins Sans Frontières; N/A, not available; PAPR, powered air-purifying respirator; PPE, personal protective equipment; RN, registered nurse.

### Risk of bias assessment summary

The risk of bias of each study is summarised in online supplemental file 8. The three included RCTs related to PPE (addressing KQ5 and KQ6) and were rated at high risk of bias.^41^,^43^ Common issues of bias included a lack of protocol, poor reporting of the randomisation/allocation process, and an absence of prespecified outcomes. Using the ROBINS-I tool, three of the included non-randomised studies were assessed as at high risk of bias^38 39 44^ and two studies at moderate risk of bias.^40 45^ Two studies were rated high risk due to the potential for bias in the measurement of outcome by an unblinded assessor.^38 39^ One study was downrated due to the use of a convenience sample, reliance on self-reports for ascertainment of exposures, and lack of reporting of details on PPE equipment or doffing protocols used between HW roles.^44^

### Summary of findings

#### Theme 1: transmission and exposure

No studies met the inclusion criteria for KQ1 (work exclusion for HWs following high-risk filovirus exposures) or KQ2 (effectiveness of disinfecting the bodies of patients deceased from EBOD or MARD), but relevant contextual information is summarised in online supplemental file 7 for all KQs.

(KQ3) Should the IPC ring approach be used versus not used to prevent and control transmission of EVD in health care facility and community settings?

No comparative studies were found that evaluated the effectiveness of the IPC ring approach (KQ3). One non-comparative observational study of a 2015 EBOD outbreak investigation in Liberia was included.^37^ Across 10 non-EBOD healthcare facilities, 166 HWs were exposed to EBOD through contact with an infected patient. The IPC ring approach was implemented after recognition that three patients had received care in a non-Ebola Treatment Unit. The IPC ring approach involves identifying HWs and healthcare facilities at elevated risk of EBOD exposure (ie, those treating confirmed patients, neighbouring facilities or facilities near a patient’s residence) and implementing IPC interventions. In this study, the intervention consisted of rapid IPC needs assessments focused on triage procedures and PPE use, as well as PPE distribution, general IPC training, and specialised triage training. Among the 166 HWs deemed exposed within the cluster, only one HW had confirmed EBOD after treating a patient. The authors suggested that the low secondary infection rates in HWs were due to the implementation of IPC measures. However, the overall certainty of evidence was rated ‘very low’ due to risk of bias and imprecision (online supplemental file 9), and no substantive conclusions could be drawn due to the lack of comparative evidence.

#### Theme 2: PPE

No studies met the inclusion criteria for KQ4 (screening/triage wearing a face shield vs mask), KQ7 (screening/triage wearing a gown vs coverall), KQ8 (disposable vs biodegradable aprons), additional PICO 1 or additional PICO 2 (screening/triage glove pairs). Relevant contextual information is summarised in online supplemental file 7 for all KQs.

(KQ5) Should HWs in direct contact and/or indirect contact to patients with EBOD or MARD cover head and neck skin and mucous membranes or just cover mucous membranes?

Five studies were included that evaluated outcomes related to heat tolerance and human errors (KQ5). Although no studies directly evaluated EBOD/MARD infection, two studies simulated filovirus contamination-related outcomes. A summary of results is provided in online supplemental file 10 Table 1.

#### Heat tolerance outcomes

The two simulation studies evaluated heat tolerance outcomes for simulated nursing conditions in ambient conditions for West African countries.^39 40^ One study^39^ evaluated the performance of two PPE ensembles with additional head/neck skin covering (Tyvek hood) in each and two ensembles with limited head/neck skin covering in six healthy individuals (full PPE details are described in online supplemental file 10 Table 1). Outcomes included core temperature at the end of exercise, skin temperature, heart rate, sweat loss, heat sensation, thermal comfort, perceived rate of exertion, breathing comfort and wetness. In the other study^40^ sweating thermal manikins with a Tyvek hood were compared with manikins with only a cap (no additional neck covering) (see online supplemental file 10 Table 1 for full PPE description). Outcomes included time to reach critical core temperature under two simulated environmental conditions, as well as skin/core temperature, heat sensation and discomfort after 80 min of activity. Across both studies, almost all heat-related outcomes were reported to be worse in groups with additional head/neck skin covering (online supplemental file 10 Table 1). For example, the time to reach a critical core temperature of 39°C under the simulated environmental conditions ranged from a mean difference of 18 min fewer (95% CI 8.2 to 27.7 min fewer) when comparing E4 (full head and neck coverage with Tyvek hood, surgical mask and surgical cap) to E1 (head and neck exposed, with a surgical mask and face shield) to 13 min fewer (95% CI 1.22 to 30.78 min fewer) comparing E3 (full head and neck coverage with hood and surgical mask) and E2 (fluid-resistant surgical cap for partial coverage). However, the effect size and statistical significance varied widely depending on the evaluated outcome and PPE sets. The overall certainty of evidence for all outcomes was judged to be of very low certainty due to risk of bias, indirectness and imprecision (online supplemental file 9).

#### Contamination during PPE doffing

Two crossover RCTs^41 43^ simulated the efficacy of PPE ensembles. One of the crossover RCTs included 59 HWs and examined the efficacy of a Hospital Authority Standard Ebola PPE set (a neck-to-ankle overall with an overlying water-resistant gown double and long nitrate gloves, boots, hood, disposable face shield and N95 respirator) and a DuPont Tyvek set, which both included a hood.^43^ These ensembles were compared against a Health Authority isolation gown for routine patient care with no hood. Another crossover trial compared PPE ensembles in 50 HWs,^41^ with one ensemble consisting of a powered air-purifying respirator (PAPR) in addition to a Tyvek hood, and the other ensemble including a bouffant hair cover (no additional neck covering). In both studies, a fluorescent solution was applied to participants to simulate contamination of PPE during clinical activities. Outcomes included the level of contamination following doffing of PPE on various areas (eg, hair, head, neck). Higher numbers of contamination patches were generally observed in groups without additional neck covering (and less PPE in general), but this varied depending on the area of examination. For one of the crossover trials, the difference of median number of contamination patches for sets with head/neck skin coverage compared with sets without the hood ranged between 17 patches higher (comparing PPE2 DuPont Tyvek, Model 1422A set with face shield, N95 respirator hood with elasticated facial opening vs PPE3 Hospital Authority isolation gown for routine patient care and performing aerosol generating procedures set with face shield and N95 respirator but no hood for extra-large patches on hair/head) and 24 patches lower (comparing PPE2 or PPE1 Hospital Authority Standard Ebola PPE set vs PPE3). Effects varied depending on the size of the contamination patch studied, the PPE comparison and the contamination area examined on the PPE.^43^ Another crossover trial reported a range between 0 fewer and 845 fewer (95% CI 621 to 924 fewer) contamination patches per 1000 for a PAPR set compared with PPE without the additional neck covering.^41^ The certainty in the level of evidence was again judged to be very low due to risk of bias, indirectness and imprecision.

#### Human errors

The two crossover RCTs^42 44^ also examined human errors, including errors and the deviation rate during donning or doffing of various pieces of PPE. Errors appeared to be slightly higher in the groups with more PPE equipment (additional cover of head/neck skin), but this was inconsistent across outcomes. A third non-randomised simulation study^39^ compared the number of errors for two training programmes (conventional and reinforced). At the first training session, there was little difference in the number of errors across outcomes for the enhanced PPE group (including a hood and surgical cap) compared with those with basic PPE (including a surgical cap but no neck covering). Depending on the specific PPE item and donning/doffing sequence, studies reported that the absolute difference in total errors ranged between 297 fewer errors per 1000 (conventional training programme with enhanced PPE (CTP-E) containing hood and surgical cap vs CTP basic PPE (CTP-B) with surgical cap but no hood; 95% CI 792 fewer to 1000 more) to 340 more errors per 1000 (PAPR set vs enhanced respiratory and contact precautions set with bouffant hair cover, face shield and N95 mask; 95% CI 53 more to 1000 more). The difference in critical errors between sets ranged between 36 fewer errors (CTP-E vs CTP-B, 95% CI 180 fewer to 144 more) to 269 more errors per 1000 (reinforced training programme with enhanced PPE (RTP-E) versus RTP with basic PPE (RTP-B), 95% CI 4 more to 689 more). One study reported the overall deviation rate during PPE doffing ranged between 0 fewer per 1000 (PPE1 Hospital Authority Standard Ebola PPE set vs PPE3 Hospital Authority isolation gown, 95% CI 30 fewer to 207 more) and 17 more per 1000 (PPE2 DuPont Tyvek, Model 1422A set vs PPE3, 95% CI 58 fewer to 226 more).^44^ Overall, very low certainty evidence showed that PPE ensembles that covered the head/neck skin resulted in more human errors during donning/doffing of equipment, compared with ensembles only covering mucous membranes.

(KQ6) Should HWs providing direct care or indirect care to patients with EBOD or MARD and using eye protection (goggles/face shield) wear them under versus over the head and neck covering?

Two studies^43 44^ met the criteria for KQ6, addressing outcomes related to simulated contamination during PPE doffing and human factors (protocol deviations during PPE doffing).

#### Contamination during PPE doffing

Both included studies were crossover RCTs^43 44^ and evaluated the efficacy of different PPE ensembles in simulated patient care scenarios (online supplemental file 10 Table 2). In both trials,^43 44^ fluorescent solution was applied to participants to simulate contamination of PPE during clinical activities. Contamination was assessed by counting the number of contaminated patches after doffing^44^ and by counting the number of participants with patches of varying sizes.^43^ While the first crossover trial’s^44^ aim was not specifically to compare protection conferred by wearing eye protection under or over the head covering, the order of layering eye protection and head covering was inferred from the reported doffing order of the items. The DuPont Tyvek set (PPE2) provided data relevant to the intervention of wearing eye protection (face shield) under the head/neck covering (hood). The Hospital Authority Standard (PPE1, a neck-to-ankle overall with an overlying water-resistant gown and long nitrate gloves, boots, hood, disposable face shield and N95 respirator) Ebola set served as the comparator, as the hood was placed under the face shield. Depending on the size and area of contamination examined following doffing of both sets of PPEs, the difference of median patches of contamination ranged between 1 patch fewer (PPE2 vs PPE1 for neck (posterior) small-sized contaminated patches during doffing) to 17 more patches (PPE2 vs PPE1 for hair and head extralarge sized contamination patches during doffing).

The other study tested 10 different EBOD PPE donning/doffing protocols from various health organisations.^43^ Of the 10 protocols, only the WHO coverall and N95 set recommend donning a face shield before the hood. All other protocols recommend face shields to be worn over the head/neck covering (see online supplemental file 10 Table 1 for full PPE details). No clear pattern emerged for whether the head cover worn under or over the head/neck covering resulted in greater contamination. The absolute difference in the number of participants with small patches following various doffing protocols ranged between 0 fewer participants per 1000 (WHO N95 protocol vs CDC PAPR, European Centers for Disease Control (ECDC), North Carolina (NC) coverall and N95, New South Wales (NSW) PAPR, NSW N95, MSF and WHO N95 protocols, respectively) and 333 fewer participants (WHO N95 protocol vs CDC N95 and Health Canada protocols, respectively) (online supplemental file 10 Table 2. The absolute difference in participants with large patches ranged between 0 fewer participants per 1000 (WHO N95 protocol vs NC protocol) and 333 more participants (WHO N95 protocol vs CDC PAPR, CDC N95, ECDC, Health Canada, NC, NSW PAPR, NSW N95, MSF, WHO N95 protocols, respectively). The certainty of evidence was rated as very low due to concerns of bias, indirectness and imprecision (online supplemental file 9).

#### Human errors

The aforementioned crossover study also reported human errors during the doffing process^43^ (online supplemental file 10 Table 2). Deviation rates appeared to be slightly higher in the groups wearing the equipment with the face shield worn under the hood, but this group also had more steps in their doffing procedure, and therefore, more opportunities for error. The absolute difference in deviation rate (%) ranged between 166 percentage points fewer per 1000 (PPE2 vs PPE1, 95% CI 192 fewer to 58 fewer) to 59 points more per 1000 (PPE2 vs PPE1, 95% CI 11 fewer to 392 more). Overall, the evidence for human errors was judged to be very low due to indirectness and imprecision (online supplemental file 9).

### Theme 3 (decontamination and disinfection)

No studies met the inclusion criteria for KQ9 (wiping vs spraying), KQ11 (hand hygiene between patients), KQ12 (handling of heavily soiled linen), but relevant contextual information is summarised in online supplemental file 7) for all KQs.

(KQ10) Should HWs to patients who have direct or indirect contact with patients with EBOD or MARD be sprayed versus not sprayed during doffing of PPE?

Two studies^44 45^ met the inclusion criteria for KQ10 and addressed outcomes related to contamination (transfer of Φ6 or MS2) and IgG antibodies against EBOV as an indicator of previous infection.

#### Contamination

One non-randomised parallel group simulation study^45^ assessed self-contamination after HWs performed a 16-step Ebola PPE doffing protocol. Participants were assigned to extra glove sanitisation through spraying of the hands with hypochlorite solution or use of an alcohol-based hand rub (ABHR). The viral load of surrogate viruses, MS2 and bacteriophage Φ6, on the hands, face, or scrubs of HWs was ascertained following inner glove removal (online supplemental file 10 Table 3). Overall, there was no detectable transfer of enveloped bacteriophage Φ6 for either intervention group, and the certainty of evidence was judged to be very low comparing the effects of hypochlorite spray and ABHR for prevention of Φ6 transfer. Depending on the location measured, spraying with chlorine resulted in a range of 800 fewer HWs per 1000 to 200 more HWs per 1000 reporting MS2 non-enveloped bacteriophage transfer after PPE doffing compared with glove sanitisation with ABHR. The certainty of evidence was judged very low due to concerns with indirectness and imprecision (online supplemental file 9).

#### Infection

One retrospective cohort study^44^ assessed the level of EBOV IgG antibody and prior exposure events among responders of the 2014–2016 West African Ebola epidemic. The study collected information on the PPE used, including whether removal of PPE was performed with or without chlorine spray (online supplemental file 10 Table 3). Indication of previous infection was higher in those who reported spraying of PPE with chlorine (180 more HWs per 1000, 95% CI 44 more to 470 more). However, almost all participants who reported performing clinical work used spray, and almost all participants who did not use spray reported having a role in laboratory work, making it impossible to analyse the independent effect of spraying PPE with chlorine due to collinearity with exposure risk. The overall certainty of evidence for the effectiveness of spraying PPE with chlorine prior to PPE removal to mitigate the risk of EBOV transmission was judged to be very low.

## Discussion

In this series of rapid reviews, nine studies were included across three different themes: transmission/exposure, PPE and disinfection/decontamination. Despite the need for practical and evidence-informed IPC recommendations during public health emergencies, persistent evidence gaps were identified. While contextual information was summarised for all KQs, relevant comparative evidence meeting the inclusion criteria was not found for the majority of KQs.

Across all themes, evidence was judged to be of low certainty using the GRADE framework. Many observational studies on the transmission of *orthoebolavirus* failed to consider potential confounding factors. For example, several studies noted that HWs also provided care in community and informal-care settings, making it difficult to ascertain what care activities and PPE failures may have led to exposure and/or transmission. Adherence to IPC protocols and the actual PPE worn at the time of suspected exposure was also often poorly reported or omitted entirely. Studies also tended to include small numbers of HWs leading to increased variability, lower statistical power and potential violations of normality assumptions in the calculation of effects. This imprecision in estimates lowered the certainty in our evidence assessments.

Some KQs focused on IPC measures in specific care contexts for HWs (ie, screening or triage scenarios). Limited evidence surrounding interventions in such contexts was found, despite the recognised risk at the point of care to HWs from patients with unrecognised EBOD or MARD.^6^

Empirical simulation studies evaluating PPE ensembles provided limited evidence surrounding the effectiveness of preventing *orthoebolavirus* or *orthomarburgvirus* transmission. Across KQs, papers noted that basic PPE ensembles did not seem to work as well as more protected PPE ensembles in preventing contamination. Generally, ensembles with additional PPE also led to an increased risk of mistakes during donning and doffing and a lowered heat tolerance. However, the evaluated sets of PPE had many differences beyond the intervention of interest. This indirectness resulted in considerable limitations when evaluating the comparative effectiveness of the interventions of interest. The trade-off between more protection and usability (eg, acceptability, cost-effectiveness, equity, heat-related outcomes) remains unclear due to a lack of high-quality comparative data.

Evidence supporting the effectiveness of various disinfection practices in real-world settings remains limited. Studies suggested that disinfection solutions can be effective against filoviruses and surrogate viruses (eg, Φ6), dependent on the disinfectant used, concentration and contact time.^46^,^48^ Importantly, there was no direct comparative evidence on the efficacy of wiping versus spraying methods for disinfection or the effectiveness of spraying HWs with chlorine. Additionally, there was a lack of research on the benefits and harms of alternative methods for decontaminating heavily soiled linens, such as chlorine washing. This knowledge gap is particularly important for low-resource settings where incineration might not be feasible. The persistence of the virus on different fabric types under various environmental conditions is also not well understood,^6 49 50^ making it difficult to determine the best practices for safely handling contaminated linens.

Finally, there were limited data available for the targeted interventions related to transmission/exposure prevention strategies, including the IPC ring approach and work exclusion policies. While some of these measures have been implemented in real-life EBOD outbreaks, such as the IPC ring approach,^37 51^ there was a lack of empirical comparative data regarding their effectiveness compared with alternative IPC strategies. Additionally, although handling of bodies of patients deceased from EBOD or MARD was noted as a major transmission risk, aligning with the conclusions from prior outbreak reports,^52^,^56^ there was a lack of strong evidence on body disinfection practices before handling or burial. Further investigation is needed to ensure these practices are both evidence-based and culturally sensitive, as issues in the acceptability of safe burial practices were identified as constituting a consistent theme in reviewed studies.^57^,^59^ These gaps support the need for targeted, comparative research to validate and optimise IPC strategies, ensuring they are both effective and practical in both healthcare and community settings.

### Implications for policy and practice

Despite the challenges in obtaining high-certainty comparative evidence, this series of rapid reviews outlines the importance of IPC measures in managing EBOD and MARD outbreaks. These rapid reviews were undertaken as part of the guideline development process to inform a consolidated WHO guideline.^15^ The guideline outlines IPC recommendations related to filovirus outbreaks in various healthcare settings, with a focus on screening, triage and care for patients with EBOD and MARD. Even informed by limited data, practical IPC recommendations can provide valuable guidance during such public health emergencies. The final WHO recommendations were formulated using the GRADE approach and considered conditional due to the low-very-low certainty of evidence, outlining the conditions that need to be considered for implementation.^15^ Recommendations were also supplemented by a mixed-methods study on HW perceptions related to the various IPC measures.^60^

### Future research

Despite almost a decade since the last review to inform WHO recommendations, high-quality comparative data on IPC interventions for EBOD and MARD remain scarce. The rarity of these diseases, combined with the geopolitical and logistical challenges of conducting research during active outbreaks, limits the feasibility of large-scale IPC studies.^14 61^ A WHO-led prioritisation exercise confirmed that PPE use and IPC training remain key areas of interest for HWs and policymakers.^62^

Given these challenges, future research should focus on feasible and ethically sound approaches. Standardised simulation studies using surrogate viruses may provide a practical and cost-effective first step in evaluating the efficacy of PPE components and disinfection strategies under controlled conditions. Insights from PPE research following the COVID-19 pandemic could also inform future work, particularly in relation to material standards and user compliance. Although transmission dynamics differ, indirect evidence from comparative studies for other high-consequence viral pathogens (eg, SARS-CoV-2, Nipah virus, other filoviruses) may help identify generalisable IPC principles to improve outbreak preparedness. Complementary qualitative and mixed-methods research is also needed to assess the acceptability and feasibility of IPC protocols (eg, discomfort, heat tolerance, ability to perform tasks, resource constraints), HW experiences, and the effects of PPE on the carer-patient relationship when caring for patients with EBOD or MARD.^63^ The recurring failure to address lessons learnt from past EBOD outbreaks has been described as a moral failing and a reflection of global health inequities.^64^ Sustained research and capacity-building are needed to break this cycle.

### Strengths and limitations

This series of rapid reviews has several strengths. The use of an a priori registered protocol established a framework and predetermined methods for the review, promoting a transparent and systematic investigation and minimising bias. Additionally, the application of standardised reporting guidelines, such as PRISMA, promotes the reliability and reproducibility of the findings across rapid reviews. The use of the GRADE framework for evaluating the overall certainty of evidence supported a standardised critical evaluation and aided in the guideline development process. Information was consolidated across various IPC themes, providing a comprehensive review of the current filovirus research landscape. Importantly, this manuscript identified ongoing gaps in the literature, highlighting critical areas requiring further investigation and guiding future research.

Several limitations should be noted. First, the number of ‘empty reviews’ for several KQs,^65^ which may reflect both the specificity of the KQs and the structural challenges of generating comparative evidence during active outbreaks. For rare diseases such as EBOD and MARD, where data are often sparse, there is an inherent challenge in balancing methodological rigour with inclusivity. Stricter eligibility criteria may minimise heterogeneity and support internal validity, but could inadvertently exclude the limited evidence available in outbreak settings where large comparative studies are rarely feasible. While empty reviews are valuable in signalling research gaps and defining the state of knowledge at a given time, overly narrow criteria may limit their broader utility.^66^ Future reviews could therefore consider more flexible inclusion criteria (eg, indirect evidence from other pathogen-based studies) and involve the research team in scoping exercises to map available types of data before finalising the KQs and eligibility criteria. Additionally, a broader range of information was included in the contextual summary to supplement the comparative effectiveness findings. Second, searches were conducted using the CAL AI-assisted screening tool. While CAL is a validated high-recall system and multiple screening sessions with different seed studies were conducted to support comprehensiveness, formal quantitative validation metrics were not captured. Performance was instead monitored through the rate of relevant studies identified across sessions, with screening stopped when 100 sequential references were excluded or when the review team reached consensus that no further relevant studies were being suggested. Third, 61 unique studies were excluded due to full-text unavailability within the 14-day retrieval window. However, a substantial proportion of these were conference abstracts for which full-text retrieval is inherently limited. Fourth, full-text review was restricted to English-language studies due to resource limitations, potentially omitting relevant evidence. Finally, due to the rapid nature of the reviews, quality and GRADE assessments were verified by a second reviewer rather than performed by two independent reviewers. Clinical experts who were part of the knowledge synthesis team considered that no important evidence had been missed; however, the possibility that some evidence was missed cannot be completely excluded.

## Conclusions

This series of rapid reviews critically examined the available evidence on IPC measures in the context of EBOD and MARD outbreaks, to help inform the IPC guideline for EBOD and MARD published in August 2023.^14^ Despite the inclusion of nine studies across the themes of transmission/exposure, PPE and disinfection/decontamination themes, high-quality comparative evidence remains scarce. Nevertheless, when combined with the qualitative data and expert opinions gathered during the guideline development process, this evidence synthesis provided the foundation for practical and contextually relevant IPC recommendations. While an urgent need for additional primary research remains, the current evidence synthesis and associated guideline provide the first step in continuing to advance preparedness and response strategies.

## Supplementary material

10.1136/bmjopen-2025-115610 online supplemental file 1

10.1136/bmjopen-2025-115610 online supplemental file 2

10.1136/bmjopen-2025-115610 online supplemental file 3

10.1136/bmjopen-2025-115610 online supplemental file 4

10.1136/bmjopen-2025-115610 online supplemental file 5

10.1136/bmjopen-2025-115610 online supplemental file 6

10.1136/bmjopen-2025-115610 online supplemental file 7

10.1136/bmjopen-2025-115610 online supplemental file 8

10.1136/bmjopen-2025-115610 online supplemental file 9

10.1136/bmjopen-2025-115610 online supplemental file 10

## Data Availability

Data are available in a public, open access repository. Data are available on reasonable request.
