# Supplementary material for: Infection prevention and control measures for Ebola and Marburg disease: a series of rapid reviews
Source: BMJ Open. 2026 Jul 9;16(7):e115610. doi: 10.1136/bmjopen-2025-115610 (PMC13358256; doi:10.1136/bmjopen-2025-115610)
Supplement: online supplemental file 2 [file bmjopen-16-7-s002.docx]

Supplementary file 2. Electronic Search Strategies

Ebola and Marburg Viruses

Final Searches

2022 Feb 12

Embase

Database: Embase Classic+Embase <1947 to 2022 February 10>

Search Strategy:

--------------------------------------------------------------------------------

1 exp filovirus/ (4751)

2 exp filovirus infection/ (7409)

3 (ebola or ebolavir* or marburgvir* or filovir* or filo vir*).tw,kw,kf. (11848)

4 (ebola* adj5 (disease* or fever* or infect* or strain? or syndrome? or virus* or epidemic* or outbreak* or pandemic*)).tw,kw,kf. (9308)

5 (marburg* adj5 (disease* or fever* or infect* or strain? or syndrome? or virus* or epidemic* or outbreak* or pandemic*)).tw,kw,kf. (1658)

6 (BDBV or EBOV or RESTV or SUDV or TAFV).tw,kw,kf. (1669)

7 ((green monkey? or vervet monkey?) adj5 (disease* or fever* or infect* or strain? or syndrome? or virus* or epidemic* or outbreak* or pandemic*)).tw,kw,kf. (952)

8 lassa fever/ (1129)

9 lassa virus/ (1198)

10 (lassa adj5 (disease* or fever* or infect* or strain? or syndrome? or virus* or epidemic* or outbreak* or pandemic*)).tw,kw,kf. (1742)

11 arenavirus*.tw,kw,kf. (1521)

12 or/1-11 [EBOLA, ETC.] (18416)

13 exp animal/ or exp animal experimentation/ or exp animal model/ or exp animal experiment/ or nonhuman/ or exp vertebrate/ (32255259)

14 exp human/ or exp human experimentation/ or exp human experiment/ (24613350)

15 13 not 14 (7643202)

16 12 not 15 [ANIMAL-ONLY REMOVED] (14357)

***************************

CENTRAL

Database: EBM Reviews - Cochrane Central Register of Controlled Trials <January 2022>

Search Strategy:

--------------------------------------------------------------------------------

1 exp Filoviridae/ (36)

2 exp Filoviridae Infections/ (104)

3 (ebola or ebolavir* or marburgvir* or filovir* or filo vir*).tw,kw. (359)

4 (ebola* adj5 (disease* or fever* or infect* or strain? or syndrome? or virus* or epidemic* or outbreak* or pandemic*)).tw,kw. (300)

5 (marburg* adj5 (disease* or fever* or infect* or strain? or syndrome? or virus* or epidemic* or outbreak* or pandemic*)).tw,kw. (21)

6 (BDBV or EBOV or RESTV or SUDV or TAFV).tw,kw. (44)

7 ((green monkey? or vervet monkey?) adj5 (disease* or fever* or infect* or strain? or syndrome? or virus* or epidemic* or outbreak* or pandemic*)).tw,kw. (3)

8 Lassa Fever/ (4)

9 Lassa Virus/ (0)

10 (lassa adj5 (disease* or fever* or infect* or strain? or syndrome? or virus* or epidemic* or outbreak* or pandemic*)).tw,kw. (10)

11 arenavirus*.tw,kw. (4)

12 or/1-11 [EBOLA, ETC.] (389)

***************************

Cochrane Database of Systematic Reviews

Database: EBM Reviews - Cochrane Database of Systematic Reviews <2005 to February 9, 2022>

Search Strategy:

--------------------------------------------------------------------------------

1 (ebola or ebolavir* or marburgvir* or filovir* or filo vir*).ti,ab,kw. (4)

2 (ebola* adj5 (disease* or fever* or infect* or strain? or syndrome? or virus* or epidemic* or outbreak* or pandemic*)).ti,ab,kw. (4)

3 (marburg* adj5 (disease* or fever* or infect* or strain? or syndrome? or virus* or epidemic* or outbreak* or pandemic*)).ti,ab,kw. (0)

4 (BDBV or EBOV or RESTV or SUDV or TAFV).ti,ab,kw. (0)

5 ((green monkey? or vervet monkey?) adj5 (disease* or fever* or infect* or strain? or syndrome? or virus* or epidemic* or outbreak* or pandemic*)).ti,ab,kw. (0)

6 (lassa adj5 (disease* or fever* or infect* or strain? or syndrome? or virus* or epidemic* or outbreak* or pandemic*)).ti,ab,kw. (0)

7 arenavirus*.ti,ab,kw. (0)

8 or/1-7 [EBOLA VIRUS, ETC.] (4)

***************************

Global Index Medicus

(tw:(ebola or ebolavir* or marburgvir* or filovir* or "filo virus" or "filo viruses")) OR (tw:(ebola*)) OR (tw:(marburg*)) OR (tw:(BDBV or EBOV or RESTV or SUDV or TAFV)) OR (tw:(lassa)) OR (tw:(arenavirus*)) – 589 results

Update

2024 May 1

Updated from 2022 Feb 12

Embase

Database: Embase Classic+Embase <1947 to 2024 April 30>

Search Strategy:

--------------------------------------------------------------------------------

1 exp filovirus/ (5958)

2 exp filovirus infection/ (8759)

3 (ebola or ebolavir* or marburgvir* or filovir* or filo vir*).tw,kw,kf. (13407)

4 (ebola* adj5 (disease* or fever* or infect* or strain? or syndrome? or virus* or epidemic* or outbreak* or pandemic*)).tw,kw,kf. (10554)

5 (marburg* adj5 (disease* or fever* or infect* or strain? or syndrome? or virus* or epidemic* or outbreak* or pandemic*)).tw,kw,kf. (1918)

6 (BDBV or EBOV or RESTV or SUDV or TAFV).tw,kw,kf. (2001)

7 ((green monkey? or vervet monkey?) adj5 (disease* or fever* or infect* or strain? or syndrome? or virus* or epidemic* or outbreak* or pandemic*)).tw,kw,kf. (987)

8 lassa fever/ (1376)

9 lassa virus/ (1412)

10 (lassa adj5 (disease* or fever* or infect* or strain? or syndrome? or virus* or epidemic* or outbreak* or pandemic*)).tw,kw,kf. (2031)

11 arenavirus*.tw,kw,kf. (1692)

12 or/1-11 [EBOLA, ETC.] (21275)

13 exp animal/ or exp animal experimentation/ or exp animal model/ or exp animal experiment/ or nonhuman/ or exp vertebrate/ (36049565)

14 exp human/ or exp human experimentation/ or exp human experiment/ (27828677)

15 13 not 14 (8222515)

16 12 not 15 [ANIMAL-ONLY REMOVED] (16673)

17 (2022021* or 2022022* or 202203* or 202204* or 202205* or 202206* or 202207* or 202208* or 202209* or 202210* or 202211* or 202212* or 2023* or 2024*).dc. (4809122)

18 16 and 17 [UPDATE PERIOD] (2555)

***************************

CENTRAL

Database: EBM Reviews - Cochrane Central Register of Controlled Trials <March 2024>

Search Strategy:

--------------------------------------------------------------------------------

1 exp Filoviridae/ (65)

2 exp Filoviridae Infections/ (174)

3 (ebola or ebolavir* or marburgvir* or filovir* or filo vir*).tw,kw. (366)

4 (ebola* adj5 (disease* or fever* or infect* or strain? or syndrome? or virus* or epidemic* or outbreak* or pandemic*)).tw,kw. (297)

5 (marburg* adj5 (disease* or fever* or infect* or strain? or syndrome? or virus* or epidemic* or outbreak* or pandemic*)).tw,kw. (29)

6 (BDBV or EBOV or RESTV or SUDV or TAFV).tw,kw. (51)

7 ((green monkey? or vervet monkey?) adj5 (disease* or fever* or infect* or strain? or syndrome? or virus* or epidemic* or outbreak* or pandemic*)).tw,kw. (1)

8 Lassa Fever/ (10)

9 Lassa Virus/ (1)

10 (lassa adj5 (disease* or fever* or infect* or strain? or syndrome? or virus* or epidemic* or outbreak* or pandemic*)).tw,kw. (21)

11 arenavirus*.tw,kw. (5)

12 or/1-11 [EBOLA, ETC.] (416)

13 (202202* or 202203* or 202204* or 202205* or 202206* or 202207* or 202208* or 202209* or 202210* or 202211* or 202212* or 2023* or 2024*).up. (937465)

14 12 and 13 [UPDATE PERIOD] (261)

***************************

Cochrane Database of Systematic Reviews

Database: EBM Reviews - Cochrane Database of Systematic Reviews <2005 to May 1, 2024>

Search Strategy:

--------------------------------------------------------------------------------

1 (ebola or ebolavir* or marburgvir* or filovir* or filo vir*).ti,ab,kw. (9)

2 (ebola* adj5 (disease* or fever* or infect* or strain? or syndrome? or virus* or epidemic* or outbreak* or pandemic*)).ti,ab,kw. (8)

3 (marburg* adj5 (disease* or fever* or infect* or strain? or syndrome? or virus* or epidemic* or outbreak* or pandemic*)).ti,ab,kw. (0)

4 (BDBV or EBOV or RESTV or SUDV or TAFV).ti,ab,kw. (0)

5 ((green monkey? or vervet monkey?) adj5 (disease* or fever* or infect* or strain? or syndrome? or virus* or epidemic* or outbreak* or pandemic*)).ti,ab,kw. (0)

6 (lassa adj5 (disease* or fever* or infect* or strain? or syndrome? or virus* or epidemic* or outbreak* or pandemic*)).ti,ab,kw. (0)

7 arenavirus*.ti,ab,kw. (0)

8 or/1-7 [EBOLA VIRUS, ETC.] (9)

9 ("20220209" or 2022021* or 2022022* or 202203* or 202204* or 202205* or 202206* or 202207* or 202208* or 202209* or 202210* or 202211* or 202212* or 2023* or 2024*).up. (5344)

10 8 and 9 [UPDATE PERIOD] (8)

***************************

Global Index Medicus

(tw:(ebola or ebolavir* or marburgvir* or filovir* or "filo virus" or "filo viruses")) OR (tw:(ebola*)) OR (tw:(marburg*)) OR (tw:(BDBV or EBOV or RESTV or SUDV or TAFV)) OR (tw:(lassa)) OR (tw:(arenavirus*)) – 668 results (no date limits applied)
