# Supplementary material for: Infection prevention and control measures for Ebola and Marburg disease: a series of rapid reviews
Source: BMJ Open. 2026 Jul 9;16(7):e115610. doi: 10.1136/bmjopen-2025-115610 (PMC13358256; doi:10.1136/bmjopen-2025-115610)
Supplement: online supplemental file 3 [file bmjopen-16-7-s003.docx]

**Appendix C. PRESS Checklist**

***PRESS Guideline* 2015— Search Submission & Peer Review Assessment**

Reference: McGowan J, Sampson M, Salzwedel DM, Cogo E, Foerster V, Lefebvre C. PRESS Peer Review of Electronic Search Strategies: 2015 guideline statement. *J Clin Epidemiol* 2016;75:40-6. Available: <http://www.jclinepi.com/article/S0895-4356(16)00058-5/pdf>.

**Search submission: This section to be filled in by the searcher**

Searcher: Becky Skidmore

Date submitted: 8 Feb 2022

Date requested by: 10 Feb 2022

| 1. **Systematic Review Title** |  |
| --- | --- |

A rapid systematic review series addressing multiple questions related to Ebola or Marburg Disease (see under PICO).

1. **This search strategy is …**

| X | My PRIMARY (core) database strategy — First time submitting a strategy for search question and database |
| --- | --- |
|  | My PRIMARY (core) strategy — Follow-up review NOT the first time submitting a strategy for search question and database. If this is a response to peer review, itemize the changes made to the review suggestions |
|  | SECONDARY search strategy— First time submitting a strategy for search question and database |
|  | SECONDARY search strategy — NOT the first time submitting a strategy for search question and database. If  this is a response to peer review, itemize the changes made to the review suggestions |

1. **Database** (e.g., MEDLINE, CINAHL)

Embase

1. **Interface** (e.g., Ovid, EbscoHost…)

 Ovid

1. **Research Question** (Describe the purpose of the search)

See under PICO

1. **PICO Format** Outline the PICOs for your question — i.e., Patient, Intervention, Comparison, Outcome, and Study Design — as applicable

*Multiple themes and questions (12):*

| **PICO Question** | **Population** | **Intervention (I)/ Comparator (C)** | **Outcome*** | **Study Design** |
| --- | --- | --- | --- | --- |
| Theme 1: Transmission/exposure | | | | |
| Should health workers who have had a low-risk EVD exposure be excluded versus not excluded from work? | Healthcare workers with a low-risk EVD exposure | I – excluded from work  C – not excluded from work | Number of Ebola / Marburg cases | High-quality and recent systematic review supplemented by primary studies |
| Should bodies of patients deceased from Ebola or Marburg disease be disinfected versus not disinfected prior to handling/moving? | Staff working in health care facilities, ETU | I – Wear a disposable apron  C – disinfection of dead bodies by wiping prior to handling/moving, spraying dead bodies with disinfectant prior to handling/moving | Number of Ebola / Marburg cases | High-quality and recent systematic review supplemented by primary studies |
| Should the IPC ring approach* be used versus not used to prevent and control transmission of Ebola Virus Disease (EVD) in health care facility and community settings? (*The IPC ring approach rapidly mobilizes teams to assist affected health facilities and the community in implementing IPC measures to reduce Ebola transmission in a predetermined risk area whenever a case is identified) | Staff, communities, organizations responsible for management of Ebola or Marburg cases | I – Implement the ring approach  C – Single intervention, single health facility prioritization | Number of Ebola / Marburg cases | High-quality and recent systematic review supplemented by primary studies |
| Theme 2: Personal Protective Equipment (PPE) | | | | |
| Should health workers providing direct or indirect care to patients with Ebola or Marburg virus disease have all intact skin and mucous membranes covered versus have mucous membranes (eyes nose and mouth) covered? | Healthcare workers providing direct or indirect care to patients with Ebola or Marburg virus disease | I – all intact skin and mucous membranes covered  C – mucous membranes covered | Number of Ebola / Marburg cases | High-quality and recent systematic review supplemented by primary studies |
| Should health workers providing direct care or indirect care to patients with Ebola or Marburg disease and using eye protection (goggles /face shield) wear them under versus over the head and neck covering? | Healthcare workers providing direct or indirect care to patients with Ebola or Marburg virus disease | I – wear eye protection over head and neck covering  C – wear eye protection under head and neck covering | Number of Ebola / Marburg cases | High-quality and recent systematic review supplemented by primary studies |
| Should health workers conducting Ebola or Marburg virus related screening and triage activities wear a gown versus wear a coverall? (Contexts to be considered: ETU use vs. healthcare facility) | Healthcare workers conducting EVD-related screening and triage activities in ETU and healthcare settings | I – wear a gown  C – wear a coverall | Number of Ebola / Marburg cases | High-quality and recent systematic review supplemented by primary studies |
| Should health workers conducting Ebola Virus Disease (EVD)-related screening and triage activities wear a face shield alone versus in combination with a medical (non-structured) mask? (Contexts to consider: ETU use vs. healthcare facility; outbreak vs readiness vs. high alert scenario). | Healthcare workers conducting EVD-related screening and triage activities in ETU, healthcare settings and across outbreak, readiness, and high alter scenarios | I – face shield plus medical (non-structured) mask  I – face shield alone | Number of Ebola / Marburg cases | High-quality and recent systematic review supplemented by primary studies |
| Should health workers using waterproof aprons to cover gowns or coveralls while providing direct or indirect care to patients with Ebola or Marburg virus disease, use disposable versus reusable versus biodegradable types of aprons? | Healthcare workers providing direct or indirect care to patients with Ebola or Marburg virus disease | I – disposable aprons  C – reusable or biodegradable aprons | Number of Ebola / Marburg cases | High-quality and recent systematic review supplemented by primary studies |
| Theme 3: Decontamination and Disinfection | | | | |
| Should surfaces and materials in Ebola treatment units (ETUs), healthcare facilities and community settings providing care to patients with Ebola or Marburg disease be disinfected using a wiping method versus a spraying method | Adult (>18 years) patients with Ebola or Marburg disease in ETUs, healthcare facilities, or community settings | I – wiping method  C – spray method | Number of Ebola / Marburg cases | High-quality and recent systematic review supplemented by primary studies |
| Should health workers providing direct or indirect care to patients with Ebola or Marburg disease be sprayed versus not sprayed during doffing of personal protective equipment? | Healthcare workers providing direct or indirect care to patients with Ebola or Marburg disease in ETUs and healthcare facilities | I – sprayed  C – not sprayed during doffing of PPE | Number of Ebola / Marburg cases    Harms associated with spray | High-quality and recent systematic review supplemented by primary studies |
| Should health workers providing direct or indirect care to patients with Ebola or Marburg disease in ETUs and healthcare facilities wash or disinfect hands OR wash or disinfect the glove between patients? | Healthcare workers providing direct or indirect care to patients with Ebola or Marburg disease in ETUs and healthcare facilities | I – decontaminate outer and inner gloves  C – wash or disinfect the glove between patients? | Number of Ebola / Marburg cases    Harms associated with decontamination or disinfection | High-quality and recent systematic review supplemented by primary studies |
| Should heavily soiled linen resulting from care to patients with Ebola or Marburg in health care, ETUs or community settings be incinerated versus disinfected? | Adult (>18 years) patients with Ebola or Marburg disease in ETUs or  healthcare facilities | I – incinerated  C – disinfected heavily soiled linen | Number of Ebola / Marburg cases    Harms associated with decontamination | High-quality and recent systematic review supplemented by primary studies |

[Note: Phi6 suggested by WHO WG as possible surrogate virus for some questions after PRESS peer-review done]

| **P** | Ebola, Marburg Disease/Fever (*including Lassa Fever for indirect evidence only*) |
| --- | --- |
| **I / Exposure** |  |
| **C** |  |
| **O** |  |
| **S** |  |

1. **Inclusion Criteria** (List criteria such as age groups, study designs, etc., to be included) *[optional]*
2. **This search strategy is …**

Search will include conference abstracts and preprints

1. **Exclusion Criteria** (List criteria such as study designs, date limits, etc., to be excluded) **[optional]**
2. **Was a search filter applied?** No

In-h

**If YES, which one(s) (e.g., Cochrane RCT filter, PubMed Clinical Queries filter)? Provide the source if this is a published filter.** *[mandatory if YES to previous question* — *textbox]*

1. **Notes or comments you feel would be useful for the peer reviewer** *[optional]*

This project will be using the CAL® tool (developed by team members Grossman and Cormack) to simultaneously search and select relevant systematic reviews and primary studies from MEDLINE, pre-print servers, and other online sources (e.g., clinicaltrials.gov, WHO International Clinical Trials Registry Platform). The CAL® tool is a patented system invented for electronic discovery in legal disputes and has won 7 international competitions in high-recall information retrieval. The CAL® tool has been used in systematic review projects to expedite the literature search and selection processes from months to weeks.

To complement the records identified using the CAL tool, we will be searching Embase and Cochrane only using traditional literature search techniques. (Note: will possibly include a few other databases – TBD.) The strategy is to be kept deliberately broad and as such we will search the “population” (i.e., disease condition) only.

Lassa Fever is also of interest (for indirect evidence) but not other haemorrhagic fevers.  [Note: Phi6 suggested by WHO WG as possible surrogate virus for some questions after PRESS peer-review done]

1. **Please copy and paste your search strategy here, exactly as run, including the number of hits per line. [mandatory]**

Database: Embase Classic+Embase <1947 to 2022 February 07>

Search Strategy:

--------------------------------------------------------------------------------

1     exp filovirus/ (4743)

2     exp filovirus infection/ (7397)

3     (ebola or ebolavir* or marburgvir* or filovir* or filo vir*).tw,kw,kf. (11830)

4     (ebola* adj5 (disease* or fever* or infect* or strain? or syndrome? or virus* or epidemic* or outbreak* or pandemic*)).tw,kw,kf. (9292)

5     (marburg* adj5 (disease* or fever* or infect* or strain? or syndrome? or virus* or epidemic* or outbreak* or pandemic*)).tw,kw,kf. (1657)

6     (BDBV or EBOV or RESTV or SUDV or TAFV).tw,kw,kf. (1666)

7     ((green monkey? or velvet monkey?) adj5 (disease* or fever* or infect* or strain? or syndrome? or virus* or epidemic* or outbreak* or pandemic*)).tw,kw,kf. (812)

8     lassa fever/ (1128)

9     (lassa adj5 (disease* or fever* or infect* or strain? or syndrome? or virus* or epidemic* or outbreak* or pandemic*)).tw,kw,kf. (1741)

10     or/1-9 [EBOLA, ETC.] (17180)

11     exp animal/ or exp animal experimentation/ or exp animal model/ or exp animal experiment/ or nonhuman/ or exp vertebrate/ (32230501)

12     exp human/ or exp human experimentation/ or exp human experiment/ (24591954)

13     11 not 12 (7639838)

14     10 not 13 [ANIMAL-ONLY REMOVED] (13755)

***************************

**Peer review assessment: this section to be filled in by the reviewer**

|  | Reviewer: Kaitryn Campbell | Email: | Date completed: 9 Feb 2022 |
| --- | --- | --- | --- |

Do you wish to be acknowledged? (If yes, the review team will be advised to add an acknowledgement to any publications related to this work).    Yes please.

The suggested acknowledgement is “We thank Kaitryn Campbell, MLIS, MSc (St. Joseph’s Healthcare Hamilton/McMaster University) for peer review of the Embase search strategy.”

1. **Translation**

| A -­‐No revisions | X |
| --- | --- |
| B -­‐ Revision(s) suggested |  |
| C -­‐ Revision(s) required |  |

 If “B” or “C,” please provide an explanation or example:

1. Boolean and Proximity Operators

| A -­‐No revisions | X |
| --- | --- |
| B -­‐ Revision(s) suggested |  |
| C -­‐ Revision(s) required |  |

1. Subject Headings

| A -­‐No revisions |  |
| --- | --- |
| B -­‐ Revision(s) suggested | X |
| C -­‐ Revision(s) required |  |

     If “B” or “C,” please provide an explanation or example:

Line 8, there’s also a heading for: Lassa virus/

1. Text Word Searching

| A -­‐No revisions |  |
| --- | --- |
| B -­‐ Revision(s)suggested |  |
| C -­‐ Revision(s) required | X |

If “B” or “C,” please provide an explanation or example:

Line 3, suggest adding: marburg-vir*

Line 7, I think this got auto-corrected, I believe should be: *vervet* monkey?

Line 9, suggest adding: arenavirus*

1. Spelling, syntax, and line numbers

| A -­‐No revisions | X |
| --- | --- |
| B -­‐ Revision(s)suggested |  |
| C -­‐ Revision(s) required |  |

       If “B” or “C,” please provide an explanation or example:

1. Limits and Filters

| A -­‐No revisions | X |
| --- | --- |
| B -­‐ Revision(s) suggested |  |
| C -­‐ Revision(s) required |  |

 If “B” or “C,” please provide an explanation or example:

1. Overall Evaluation Note

| A -­‐No revisions |  |
| --- | --- |
| B -­‐ Revision(s) suggested |  |
| C -­‐ Revision(s) required | X |

Additional comments:

Nicely done (a lot simpler than I thought it was going to be as I was reading through the questions!)—a few suggested additions and one correction made.
