# Supplementary material for: Infection prevention and control measures for Ebola and Marburg disease: a series of rapid reviews
Source: BMJ Open. 2026 Jul 9;16(7):e115610. doi: 10.1136/bmjopen-2025-115610 (PMC13358256; doi:10.1136/bmjopen-2025-115610)
Supplement: online supplemental file 4 [file bmjopen-16-7-s004.docx]

**Supplementary file 4. Eligibility (PICO) criteria (by theme)**

**Theme 1: Transmission/Exposure**

**Question (1): Should health workers who have had EVD or Marburg virus disease exposure other than high-risk be excluded versus not excluded from work?**

Background: 1) What is the risk of EVD or Marburg virus disease acquisition with different types of occupational exposures? 2) If acquired, what is the risk of transmitting the virus?

| Setting | Health care facilities, ETU, *community* |
| --- | --- |
| Population | Staff working in health care facilities, ETU    Sub-groups:  **High risk patient care activity** Broken skin or mucous membrane contact with a patient with Ebola virus disease (alive or deceased) or their bodily fluids:   - Bodily fluid in direct contact with mucous membrane (e.g. eyes, nose or mouth) - Penetrating sharps injury from used device or through contaminate - Performed finger prick - Put in IV - Delivered babies - Performed invasive procedure - Performed major surgery - Performed autopsy - Drew blood - Cleaned blood spill - Controlled bleeding - Performed minor surgery - Moved dead bodies - Cleaned or disinfected latrines     **Intermediate risk patient care activities** (intact-skin-only contact with a patient with Ebola virus disease or their body fluids):d   - Clinical assessment of an individual with suspected Ebola virus disease before diagnosis without appropriate personal protective equipment (PPE), Close contact with a patient, body or body fluid, linen or clothes of an infected patient/person - Bathes or cleaned patients - Gave injection - Handled urinary catheter - Contact with contaminated surfaces - Recapped needle - Handled IV line (e.g., gave IV medications) - Handled waste - Handled linen or clothes or mattresses     **Low risk patient care activities** (No direct contact with a patient with Ebola virus disease or their body fluids):   - Living in the same house as a patient with Ebola virus disease but no direct contact with their bodily fluids) - Breach of personal protective equipment (PPE) without risk of contamination - Provided general patient care (took vital signs, examined patients, moved patients) - Fed patients or administered oral medications - Discarded sharps (appropriately) - Cleaned patient room or ward Living in same house as a patient with EVD but no direct contact with their body fluids - Moved/ transported patients |
| Background interventions  (Standard of care) | Continue with normal duties (no work exclusion) |
| Intervention | Continue with normal duties (no work exclusion) |
| Comparator(s) | Exclude from work for 21 days |
| Outcome | Infection with Ebola or Marburg virus, *health-care associated transmission of Ebola*    **Indirect evidence:** Lassa fever |
| Potential effect modifiers | Impact of vaccination status on post exposure actions  *Community exposures during exclusion period, type of exposure, vaccination* |

**Question (2): Should bodies of patients deceased from Ebola or Marburg virus disease be disinfected versus not disinfected prior to handling/moving *into a body bag?***

| Setting | Health care facility, ETU, community |
| --- | --- |
| Population | Health workers and Burial teams handling bodies of Ebola and Marburg patients |
| Background interventions | Varies by organization. WHO says remains should not be sprayed, washed or embalmed. |
| Intervention | no disinfection of dead bodies prior to handling/moving |
| Comparator(s) | 1) disinfection of dead bodies by wiping prior to handling/moving, 2) spraying dead bodies with disinfectant prior to handling/moving |
| Outcome | Symptoms of chemical exposure from spraying dead bodies, exposure during handling dead bodies, infection with Ebola or Marburg |
| Potential effect modifiers | *Ventilation in the area where bodies are sprayed may affect the outcome, vaccination* |

**Question (3): Should the IPC ring approach* be used versus not used to prevent and control transmission of Ebola Virus Disease (EVD) or Marburg virus disease in health care facility and community settings?** (How effective is ring IPC at preventing Ebola or Marburg transmission in health care and community settings?)

*The ring ICP approach rapidly mobilizes teams to assist affected health facilities and the community in implementing ICP measures to reduce Ebola transmission in a predetermined risk area whenever a case is identified.

| Setting | Health care facility, community |
| --- | --- |
| Population | Staff, communities, organizations responsible for management of Ebola or Marburg cases |
| Background interventions  (Standard of care) | New approach: Use the IPC ring approach when a new case of EVD is identified. The IPC ring approach rapidly mobilizes teams to assist affected health facilities and the community in implementing IPC measures to reduce Ebola transmission in a predetermined risk area whenever a case is identified. |
| Intervention | Implement the ring approach, which Includes identification of nearby health centres, household and public places visited by the positive case for case finding, environmental cleaning/decontamination, IPC assessment, education, PPE supplies. |
| Comparator(s) | Single intervention, Single health facility prioritization |
| Outcome | Transmission of Ebola or Marburg, *score of IPC standard in the HCF* |
| Potential effect modifiers | **Effect modifier** – conflict zone |

 *The IPC ring approach rapidly mobilizes teams to assist affected health facilities and the community in implementing IPC measures to reduce Ebola transmission in a predetermined risk area whenever a case is identified.

**Theme 2: Personal Protective Equipment (PPE)**

**Question (4): Should health workers conducting EVD or Marburg virus disease related screening and triage activities wear a face shield alone versus in combination with a medical (non-structured) mask?**

| Population | Staff performing screening and triage activities in health care facility or ETU |
| --- | --- |
| Background interventions  (Standard of care) | WHO current guidance: *“Staff in the triage area should wear a scrub suit, a gown, examination gloves and a face shield. The area should be large enough to keep the patient at a 1-metre distance...”* |
| Intervention | wearing a medical mask in combination with the face shield |
| Comparator(s) | Not wearing a medical mask with the face shield |
| Outcome | Infection with Ebola or Marburg, *PPE breaches, compliance and/or breaches (touching face) related to heat/humidity and comfort, human factors, health worker confidence*  **Indirect evidence:** Lassa fever |
| Potential effect modifiers | *Vaccination status, Design of face shield used  may affect the face protection, heat/humidity and comfort* |

**Question (5): Should Health workers in direct contact and/or indirect contact to patients with Ebola Virus Disease (EVD) or Marburg virus disease cover head and neck skin and mucous membranes or just cover mucous membranes?**

| Setting | Health care facilities, ETU, *community (e.g. burial teams)*  *Contexts to consider: ETU use vs. healthcare facility; outbreak vs readiness vs. high alert scenario. |
| --- | --- |
| Population | Staff in HCF, ETU, community (e.g. burial teams) |
| Background interventions  (Standard of care) | The mucous membranes of eyes, mouth and nose are covered by PPE. Use of a head cover that covers head and neck. |
| Intervention | Use a cover for the head and neck. |
| Comparator(s) | Not use a cover for head and neck.  *Direct contact, indirect contact* |
| Outcome | Infection with Ebola or Marburg, PPE breaches, compliance related to heat and comfort, *dehydration, heat tolerance, human factors, health worker confidence*    **Indirect evidence:** Lassa fever |
| Potential effect modifiers | *Frequency and type of exposure, vaccination* |

**Question (6): Should health workers providing direct care or indirect care to patients with Ebola or Virus Marburg disease and using eye protection (goggles /face shield) wear them under versus over the head and neck covering?**

| Setting | Health care facilities, ETU, community (e.g., burial teams) |
| --- | --- |
| Population | Health workers in health care facilities, ETU and community |
| Background interventions  (Standard of care) | Wearing eye protection (goggles /face shield) and head & neck covering. |
| Intervention | Wearing (goggles /face shield) under the head/neck covering, |
| Comparator(s) | Wearing eye protection (goggles/face shield) over the head /neck covering |
| Outcome | Infection with Ebola or Marburg, PPE breaches (exposures), *comfort, visibility and communication, human factors*    **Indirect evidence:** Lassa fever |
| Potential effect modifiers | PPE design  Doffing procedure employed during doffing  PPE supply (goggles versus face shields)  *Spaying vs. not spraying, vaccination* |

**Question (7)- (a): Should health workers conducting Ebola or Marburg virus disease related screening activities wear a gown versus wear a coverall?**

| Setting | Health care facilities, ETU    *Contexts to consider: ETU use vs. healthcare facility; outbreak vs readiness vs. high alert scenario. |
| --- | --- |
| Population | Staff performing screening activities in health care facility or ETU |
| Background interventions  (Standard of care) | Staff in the screening area should wear a scrub suit, a gown, examination gloves and a face shield. |
| Intervention | wearing a gown |
| Comparator(s) | wearing coverall |
| Outcome | Infection with Ebola or Marburg, PPE breaches, compliance related to heat and comfort, *human factors, health worker confidence*    **Indirect evidence:** Lassa fever |
| Potential effect modifiers | *Vaccination, Receiving training for proper doffing,*  *Staff job duties/activities in triage (pass/receive things, escort them to a new location, etc.)*  *volume of patients, physical distance from patients, and hours of work (long vs. short shift)* |

**Question (7)- (b): Should health workers conducting Ebola or Marburg virus disease related triage activities wear a gown versus wear a coverall?**

| Setting | Health care facilities, ETU    *Contexts to consider: ETU use vs. healthcare facility; outbreak vs readiness vs. high alert scenario. |
| --- | --- |
| Population | Staff performing triage activities in health care facility or ETU |
| Background interventions  (Standard of care) | Staff in the triage area should wear a scrub suit, a gown, examination gloves and a face shield. |
| Intervention | wearing a gown |
| Comparator(s) | wearing coverall |
| Outcome | Infection with Ebola or Marburg, PPE breaches, compliance related to heat and comfort, *human factors, health worker confidence*  **Indirect evidence:** Lassa fever |
| Potential effect modifiers | *Vaccination, Receiving  training for proper doffing,*  *Staff job duties/activities in triage (pass/receive things, escort them to a new location, etc.)*  *volume of patients, physical distance from patients, and hours of work  (long vs. short shift)* |

**Question (8): Should health workers using waterproof aprons to cover gowns or coveralls while providing direct or indirect care to patients with Ebola or Marburg virus disease, use disposable versus reusable versus biodegradable types of aprons?**

| Setting | Health care facilities, ETU |
| --- | --- |
| Population | Staff working in health care facilities, ETU |
| Background interventions  (Standard of care) | The choice of apron should be, in order of preference:  • a disposable, waterproof apron  • if disposable aprons are not available, heavy duty, reusable waterproof aprons may be used provided that they are appropriately cleaned and disinfected between patients |
| Intervention | Wear a disposable waterproof apron |
| Comparator(s) | 1) wear a reusable waterproof heavy-duty apron, 2) wear a biodegradable waterproof apron |
| Outcome | Environmental impact of single-use disposable PPE, exposures while cleaning and disinfecting aprons, breaches in cleaning and disinfection practice infection/transmission of EVD, *PPE breaches/exposures, ease of doffing PPE* |
| Potential effect modifiers | *The design of apron, vaccination* |

**ADDITIONAL PICO 1 Should health workers conducting screening where at least 1m distance and a no touch technique can be maintained and PPE (such as gown, and facial protection) is not expected wear no gloves and perform hand hygiene versus wear 1 pair of gloves?**

|  |  |
| --- | --- |
| Population | Health workers conducting screening activities where 1 m distance and no touch technique can be maintained and PPE (such as gown, and facial protection) is not expected |
| Intervention | No gloves and perform hand hygiene |
| Comparator | 1 pair of gloves |
| Outcome | EVD/Marburg transmission to health workers conducting screening activities |
| Definitions | **Screening** – A sorting process separating the people who probably do have the condition from those who probably do not ([9789289054782-eng.pdf (who.int)](https://apps.who.int/iris/bitstream/handle/10665/330829/9789289054782-eng.pdf). For EVD this can be done with questions and a non-contact thermometer, thus does not require close or physical contact with the patient. |

**ADDITIONAL PICO 2 Should health workers conducting screening and/or triage activities where at least 1m distance cannot be maintained and PPE (such as gown, and facial protection) is expected, wear 2 pairs of gloves versus 1 pair of gloves?**

| Population | Health workers conducting screening and/or triage activities where at least 1 m distance cannot be maintained, and PPE (such as gown, and facial protection) is expected |
| --- | --- |
| Intervention | 2 pair of gloves |
| Comparator | 1 pair of gloves |
| Outcome | EVD/Marburg transmission to health workers conducting screening and/or triage activities |
| Definitions | **Screening** – A sorting process separating the people who probably do have the condition from those who probably do not ([9789289054782-eng.pdf (who.int)](https://apps.who.int/iris/bitstream/handle/10665/330829/9789289054782-eng.pdf). For EVD this can be done with questions and a non-contact thermometer, thus does not require close or physical contact with the patient.    **Triage** – The assessment of patients using validated tools to determine the severity of illness and prioritise care (<https://apps.who.int/iris/bitstream/handle/10665/43386/9241546875_eng.pdf?sequence=1)>​. For EVD this often requires close and physical contact with the patient in order to perform the assessment. |

**Theme 3: Decontamination and Disinfection**

**Question (9): Should surfaces and materials in healthcare facilities, Ebola treatment units (ETU) and community settings providing care to patients with Ebola or Marburg disease be disinfected using a wiping method versus a spraying method**

| Setting | Health care facility, ETU, community |
| --- | --- |
| Population | Staff and or patients in healthcare facilities (HCF), ETU and community |
| Background interventions  (Standard of care) | Disinfection of surfaces daily and when visibly soiled |
| Intervention | spray surfaces with disinfectant |
| Comparator(s) | wipe surfaces with disinfectant |
| Outcome | Adverse effects associated with chemical exposure, coverage of surfaces with disinfectant, log reduction of virus or surrogate on surface, infection with Ebola, *psychological effects (stigma) associated with spraying of homes with disinfectants, patient experience (e.g. extensive chlorine smell in the environment/skin exposure, etc.,* |
| Potential effect modifiers | Disinfectant chemical used  Design/spraying technology  Adequacy of spraying (surface coverage)  Surface cleaning first  *Time of exposure to disinfectant*  *Surface composition*  *Concentration of solution*  *Disinfectant product* |
| Setting | Health care facilities, ETU, community |

**Question (10): Should health workers to patients with Ebola or Marburg disease be sprayed versus not sprayed during doffing of personal protective equipment (PPE)?**

| Population | Staff in HCF, ETU and community (e.g., burial teams) |
| --- | --- |
| Background interventions  (Standard of care) | Varies by organization. WHO recommends staff remove PPE in correct order, no spraying |
| Intervention | Staff spraying with chlorine solution prior to removing PPE |
| Comparator(s) | No Staff spraying with chlorine solution prior to removing PPE |
| Outcome | Adverse effects associated with chemical exposure, infection with Ebola virus or Marburg, *health worker confidence* |
| Potential effect modifiers | Decontamination method and the types of PPE, *Chlorine concentration, chlorine type* |
| Setting | Health Care Facilities, ETU  *Contexts to consider: ETU use vs. healthcare facility; outbreak vs readiness vs. high alert scenario. |

**Question (11)-(a): Should health workers providing direct or indirect care to patients with Ebola or Marburg disease in ETUs and healthcare facilities wash hands (soap & water) OR wash the glove (soap & water) between patients?**

| Setting | Health care facilities, ETU |
| --- | --- |
| Population | Health workers working in health care facilities, ETU |
| Background interventions | 1) Disinfect outer gloves before removing them and 2) keep inner gloves on and disinfect them before putting on a fresh outer pair. |
| Intervention | Hand hygiene (including glove disinfection) between patients |
| Comparator(s) | 1. Removal of outer glove and hand hygiene (inner glove) w/ soap and water 2. Removal of both gloves and hand hygiene w/ soap and water 3. Disinfecting outer glove w/ soap and water |
| Outcome | Dermatitis, PPE breaches/exposures, compliance, Ebola or Marburg virus infection, *human factors, health worker confidence* |
| Potential effect modifiers | May depend on the nature of the patient contact (e.g., if there was contact with blood/body fluids or if the outer glove is visibly dirty)  Single-use versus reusable gloves  *Type of gloves (e.g.: nitrile versus latex*  *Number of times inner /outer gloves are disinfected*  *Health facility versus ETU Setting, vaccination* |

**Question (11)-(b): Should health workers providing direct or indirect care to patients with Ebola or Marburg disease in ETUs and healthcare facilities disinfect hands with ABHR OR disinfect the glove with ABHR between patients?**

| Setting | Health care facilities, ETU |
| --- | --- |
| Population | Health workers working in health care facilities, ETU |
| Background interventions | 1) Disinfect outer gloves before removing them and 2) keep inner gloves on and disinfect them before putting on a fresh outer pair. |
| Intervention | Hand hygiene (including glove disinfection) between patients |
| Comparator(s) | 1. Removal of outer glove and hand hygiene (inner glove) w/ alcohol-based hand rub 2. Removal of both gloves and hand hygiene w/ alcohol-based hand rub 3. Disinfecting outer glove w/ alcohol-based hand rub |
| Outcome | Dermatitis, PPE breaches/exposures, compliance, Ebola or Marburg virus infection, *human factors, health worker confidence* |
| Potential effect modifiers | May depend on the nature of the patient contact (e.g., if there was contact with blood/body fluids or if the outer glove is visibly dirty)  Single-use versus reusable gloves  *Type of gloves (e.g.: nitrile versus latex*  *Number of times inner /outer gloves are disinfected*  *Health facility versus ETU Setting, vaccination* |

**Question (11)-(c): Should health workers providing direct or indirect care to patients with Ebola or Marburg disease in ETUs and healthcare facilities disinfect hands (with chlorine) OR disinfect the glove(with chlorine) between patients?**

| Setting | Health care facilities, ETU |
| --- | --- |
| Population | Health workers working in health care facilities, ETU |
| Background interventions | 1) Disinfect outer gloves before removing them and 2) keep inner gloves on and disinfect them before putting on a fresh outer pair. |
| Intervention | Hand hygiene (including glove disinfection) between patients |
| Comparator(s) | 1. Removal of outer glove and hand hygiene (inner glove) w/ chlorine 2. Removal of both gloves and hand hygiene w/ chlorine 3. Disinfecting outer glove w/ chlorine (concentration) |
| Outcome | Dermatitis, PPE breaches/exposures, compliance, Ebola or Marburg virus infection, *human factors, health worker confidence* |
| Potential effect modifiers | May depend on the nature of the patient contact (e.g., if there was contact with blood/body fluids or if the outer glove is visibly dirty)  Single-use versus reusable gloves  *Type of gloves (e.g.: nitrile versus latex*  *Number of times inner /outer gloves are disinfected*  *Health facility versus ETU Setting, vaccination* |

**Question (12): Should heavily soiled linen resulting from care to patients with Ebola or Marburg disease in health care, ETUs or community settings be incinerated versus disinfected?** Risks related to staff/person handling the linens (washing manually or by machine wash)

| Setting | Health care facility, ETU, community (e.g., burial team) |
| --- | --- |
| Population | Staff working in Health care facility, ETU, community |
| Background interventions  (Standard of care) | Heavily soiled, contaminated linen should preferably be incinerated or processed by autoclaving.    Washing contaminated linen by hand should be discouraged, if washing machines are not available or power is not ensured, take the soiled linen out of the container and empty it into a large drum container of water and soap. Soak the linen in this drum and make sure it is totally covered with water. Use a stick to stir; then throw out the water, refill the drum with chlorine 0,05% (a solution containing 500 ppm available free chlorine) and soak for 15 minutes. |
| Intervention | Incineration of heavily soiled linen |
| Comparator(s) | Laundering heavily soiled linen |
| Outcome | Staff exposure during handling and laundering of linens, transmission of Ebola and Marburg |
| Potential effect modifiers | Investment in cleaning, decontamination and sterilization  Use of mechanical washers versus manual (by hand) washing, infra-estructure for proper laundry  Type of disinfectant used (toxicity, corrosion, environmentally safe to use)  *Quality of linens for re-use,*  *vaccination* |
