# Supplementary material for: Infection prevention and control measures for Ebola and Marburg disease: a series of rapid reviews
Source: BMJ Open. 2026 Jul 9;16(7):e115610. doi: 10.1136/bmjopen-2025-115610 (PMC13358256; doi:10.1136/bmjopen-2025-115610)

**Supplementary file 5. PRISMA Flow Diagrams**

Contents

[Theme 1: Transmission/Exposure 2](#_Toc173491510)

[KQ1 PRISMA Flow Diagram 2](#_Toc173491511)

[KQ2 PRISMA Flow Diagram 3](#_Toc173491512)

[KQ3 PRISMA Flow Diagram 4](#_Toc173491513)

[Theme 2: Personal Protective Equipment 5](#_Toc173491514)

[KQ4/7 PRISMA Flow Diagram 5](#_Toc173491515)

[KQ5 PRISMA Flow Diagram 6](#_Toc173491516)

[KQ6 PRISMA Flow Diagram 7](#_Toc173491517)

[KQ8 PRISMA Flow Diagram 8](#_Toc173491518)

[Additional PICO 1/2 PRISMA Flow Diagram 9](#_Toc173491519)

[Theme 3: Decontamination and Disinfection 10](#_Toc173491520)

[KQ9 PRISMA Flow Diagram 11](#_Toc173491521)

[KQ10 PRISMA Flow Diagram 12](#_Toc173491522)

[KQ11 PRISMA Flow Diagram 13](#_Toc173491523)

[KQ12 PRISMA Flow Diagram 14](#_Toc173491524)

[Search Update 1 (KQ1) PRISMA Flow Diagram 15](#_Toc173491525)

[Search Update 2 (KQ2, KQ9, KQ10) PRISMA Flow Diagram 16](#_Toc173491526)

[Search Update 3 (KQ3) PRISMA Flow Diagram 17](#_Toc173491527)

[Search Update 4 (KQ4, KQ5, KQ6, KQ7A, KQ7B, KQ8) PRISMA Flow Diagram 18](#_Toc173491528)

[Search Update 5 (KQ11A, KQ11B, KQ11C, Additional PICO 1, Additional PICO 2) PRISMA Flow Diagram 19](#_Toc173491529)

[Search Update 6 (KQ12) PRISMA Flow Diagram 20](#_Toc173491530)

# Theme 1: Transmission/Exposure

## KQ1 PRISMA Flow Diagram


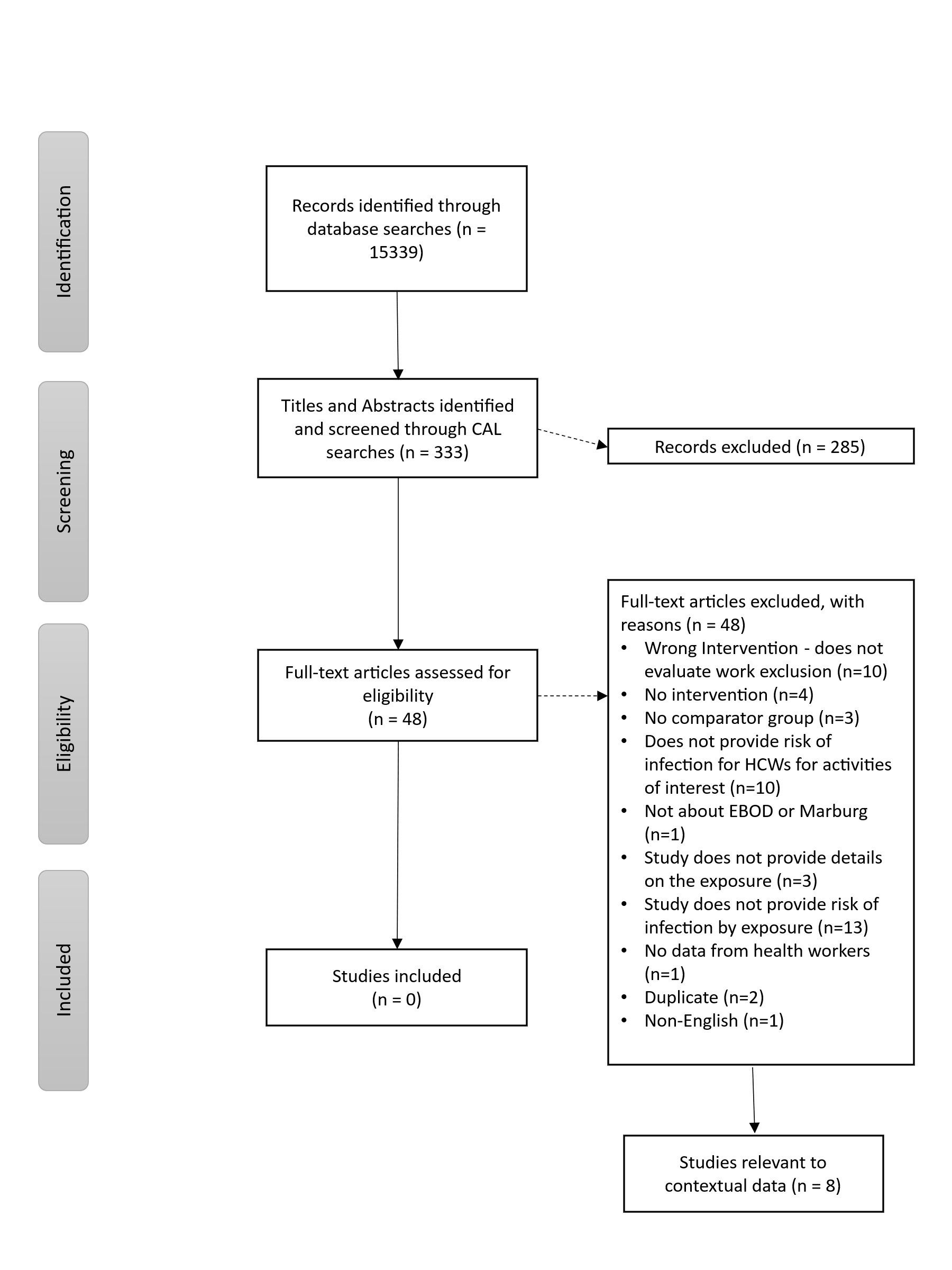


## KQ2 PRISMA Flow Diagram


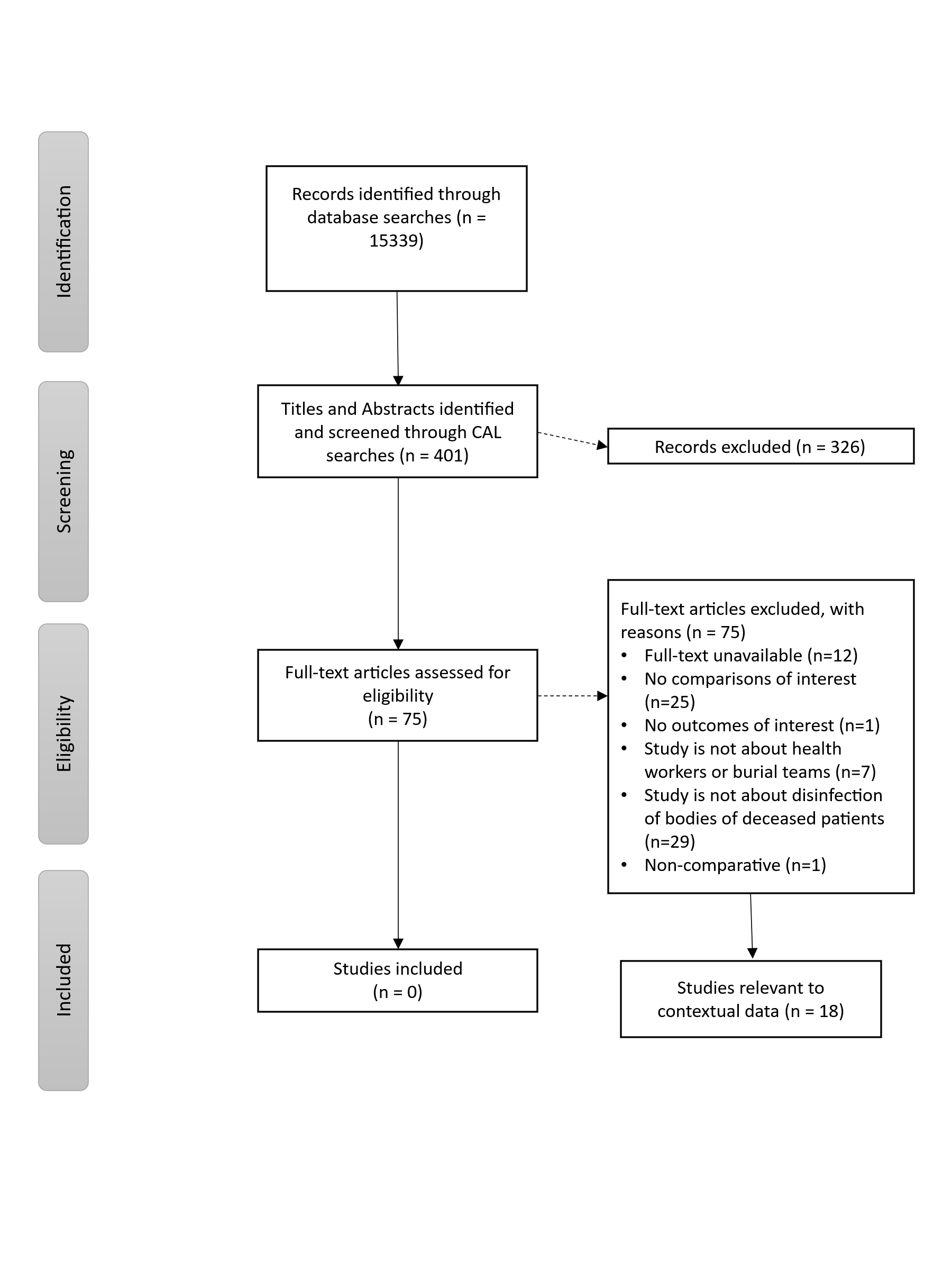


## KQ3 PRISMA Flow Diagram


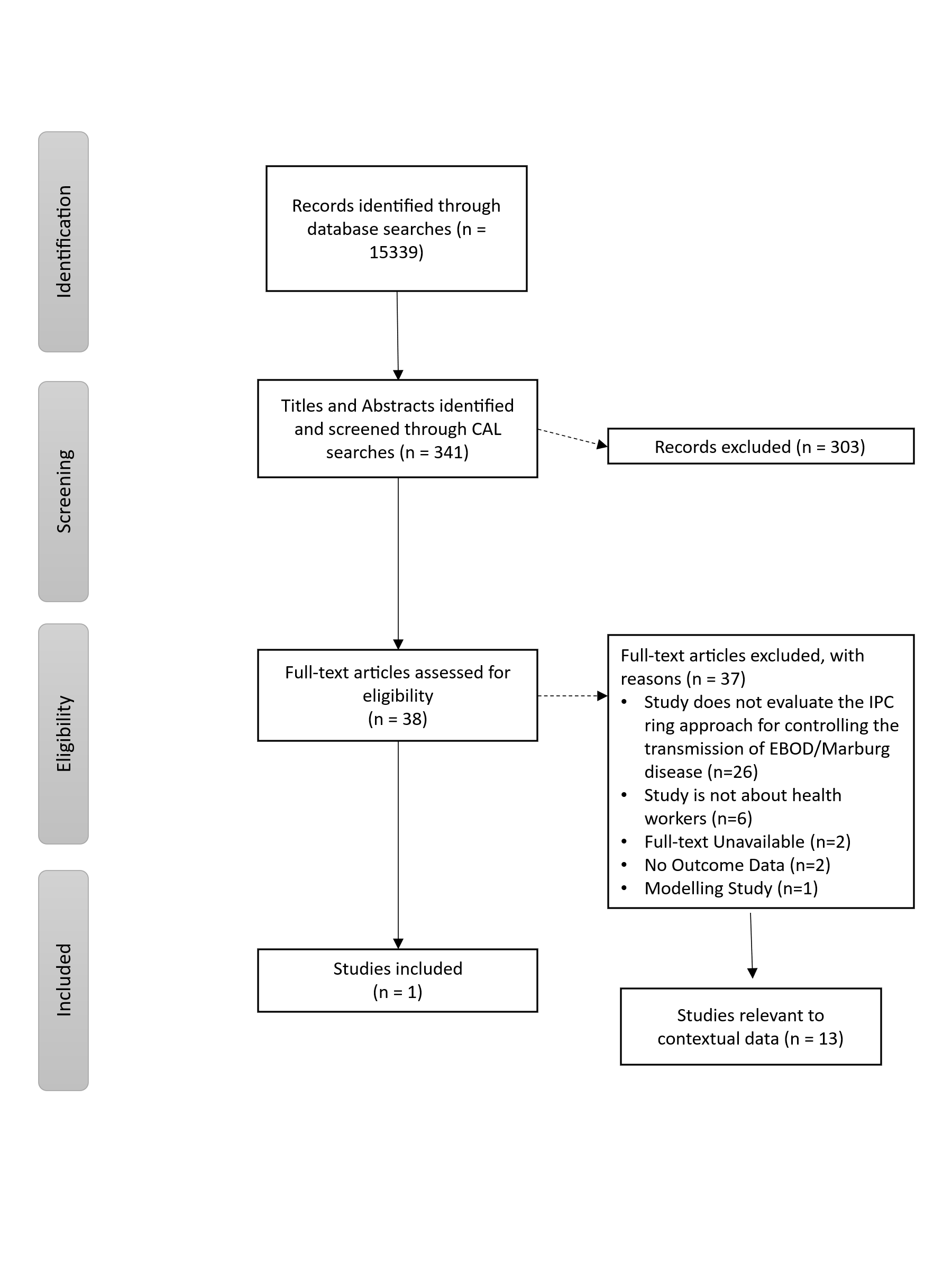


# Theme 2: Personal Protective Equipment

## KQ4/7 PRISMA Flow Diagram


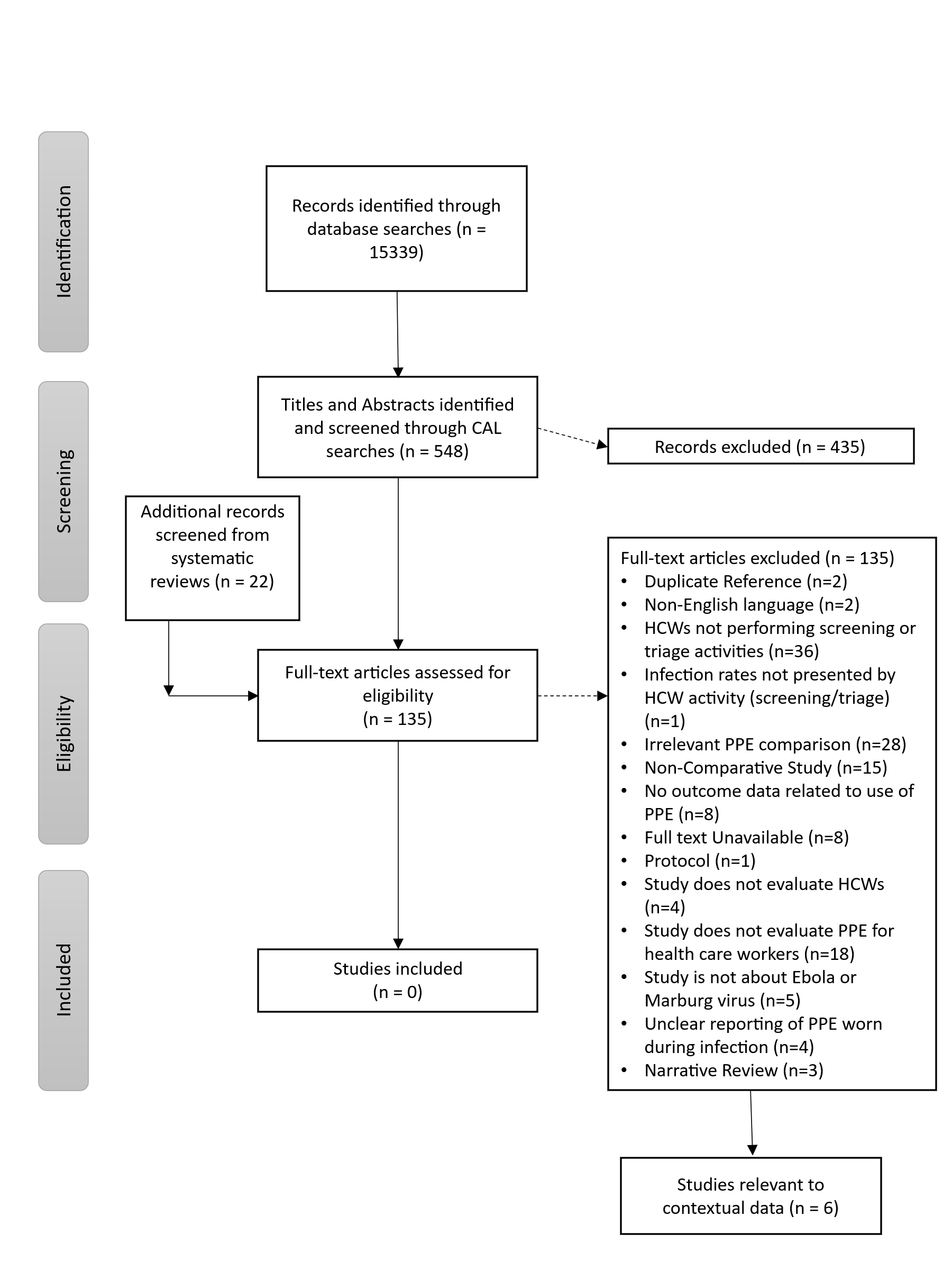


## KQ5 PRISMA Flow Diagram


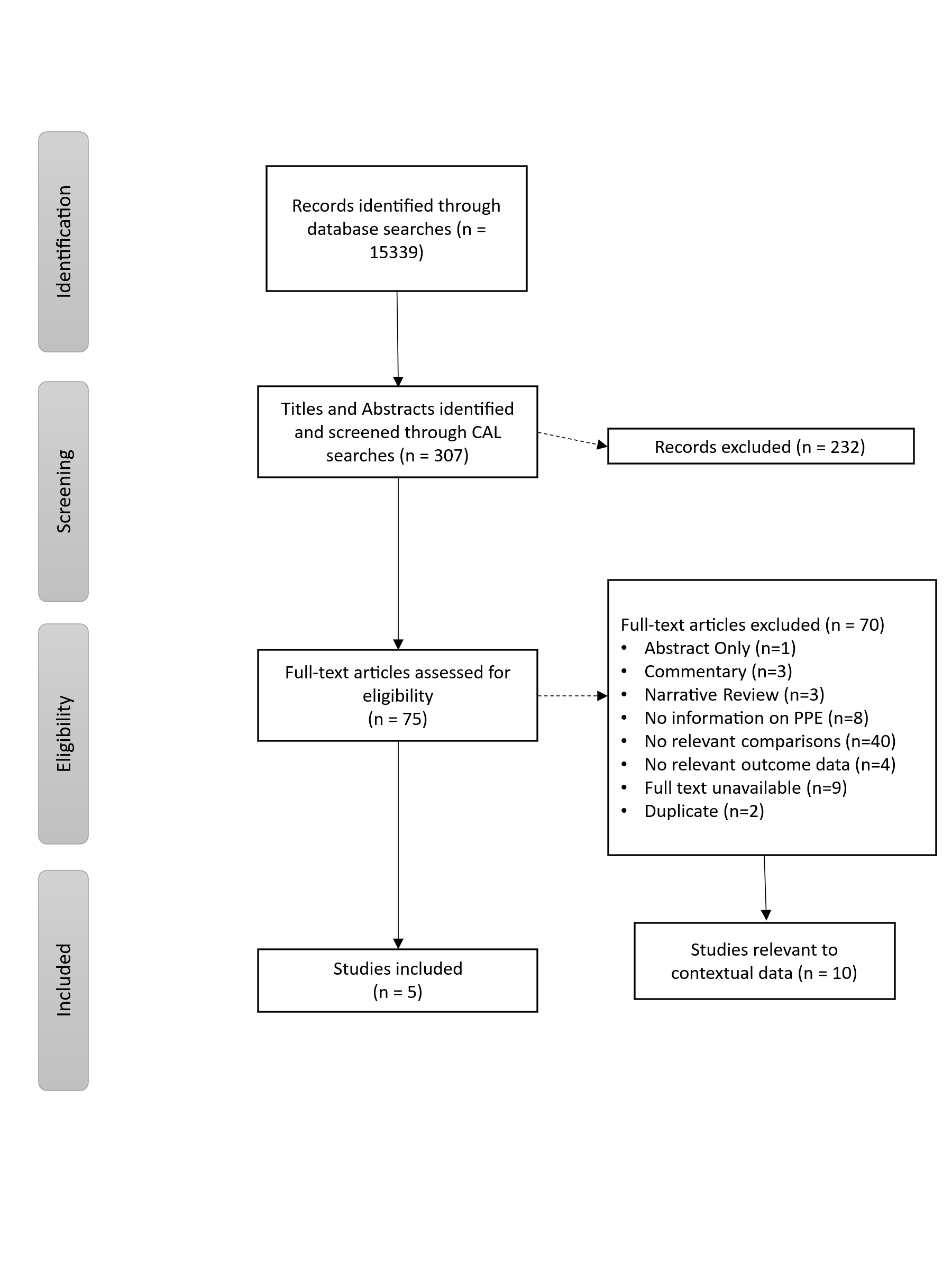


## KQ6 PRISMA Flow Diagram


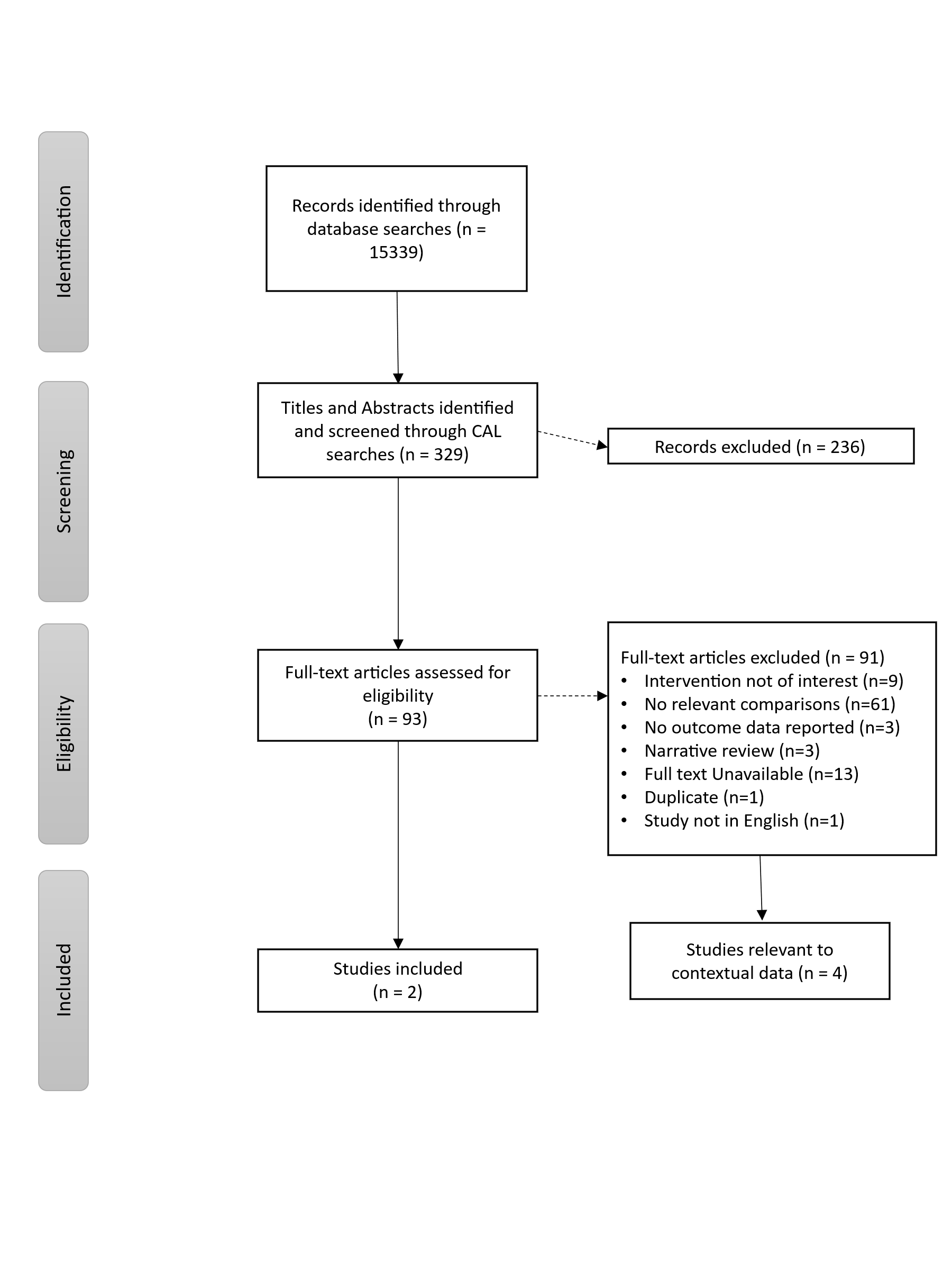


## KQ8 PRISMA Flow Diagram


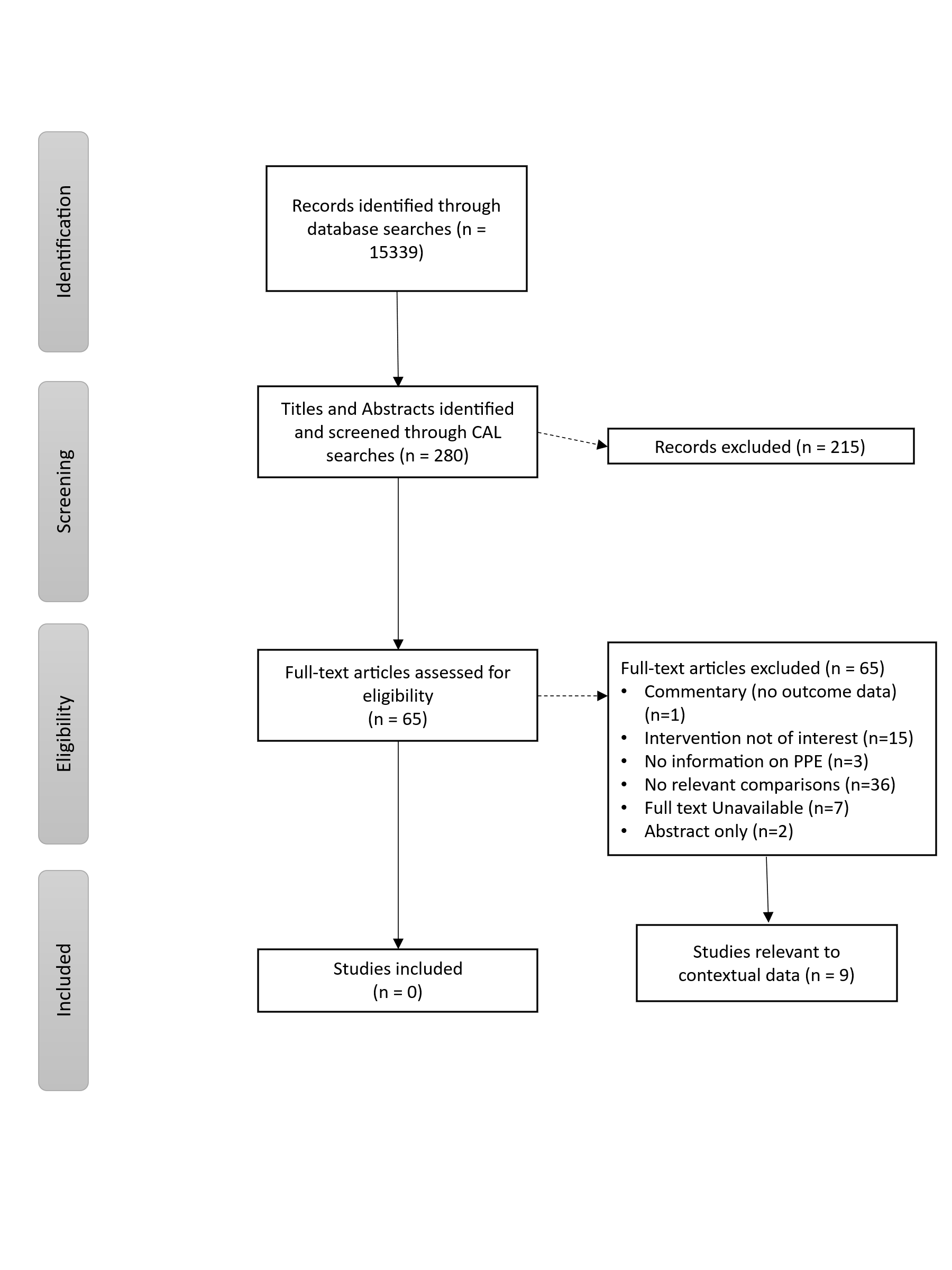


## Additional PICO 1/2 PRISMA Flow Diagram


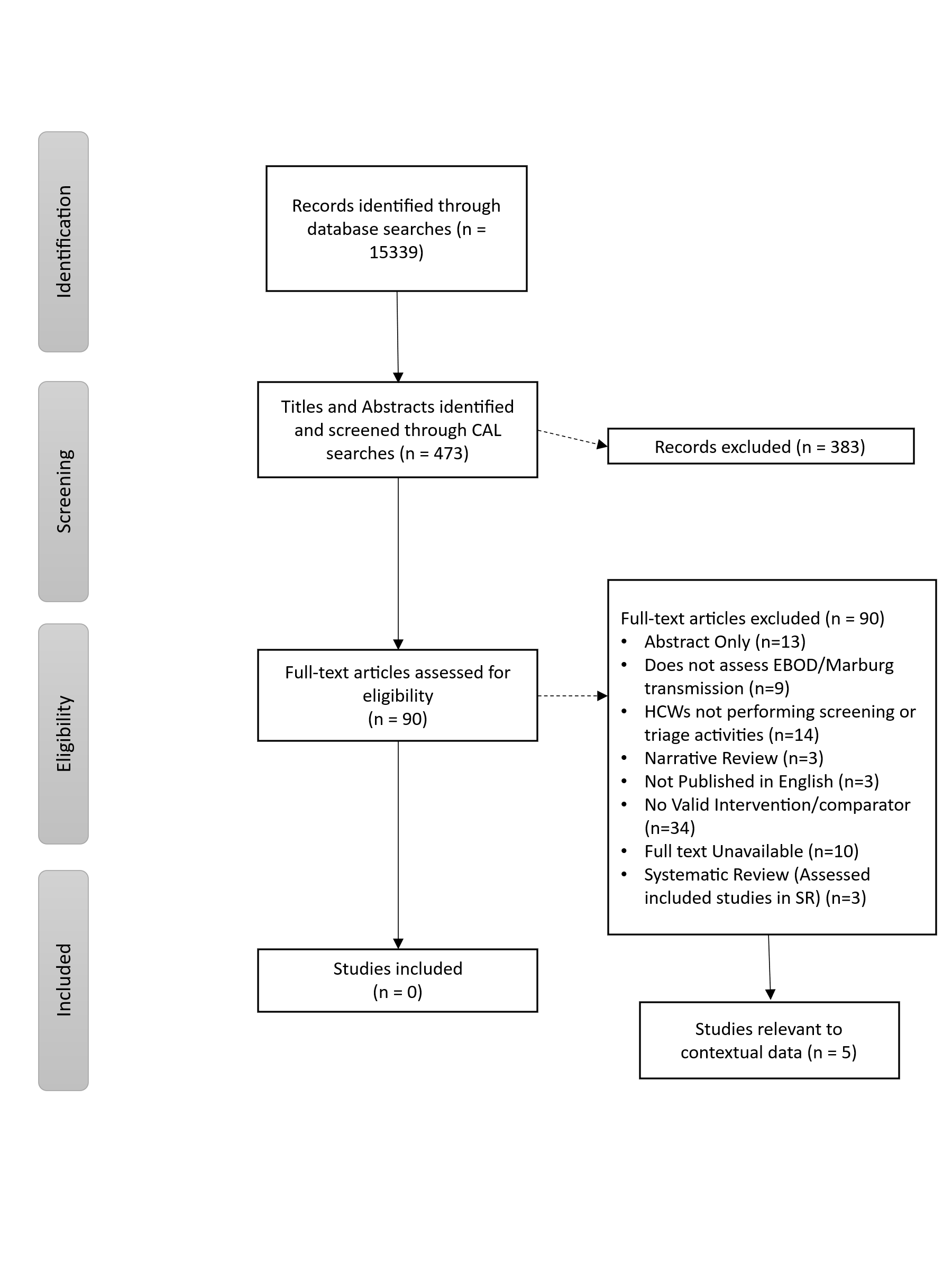


# Theme 3: Decontamination and Disinfection

## KQ9 PRISMA Flow Diagram


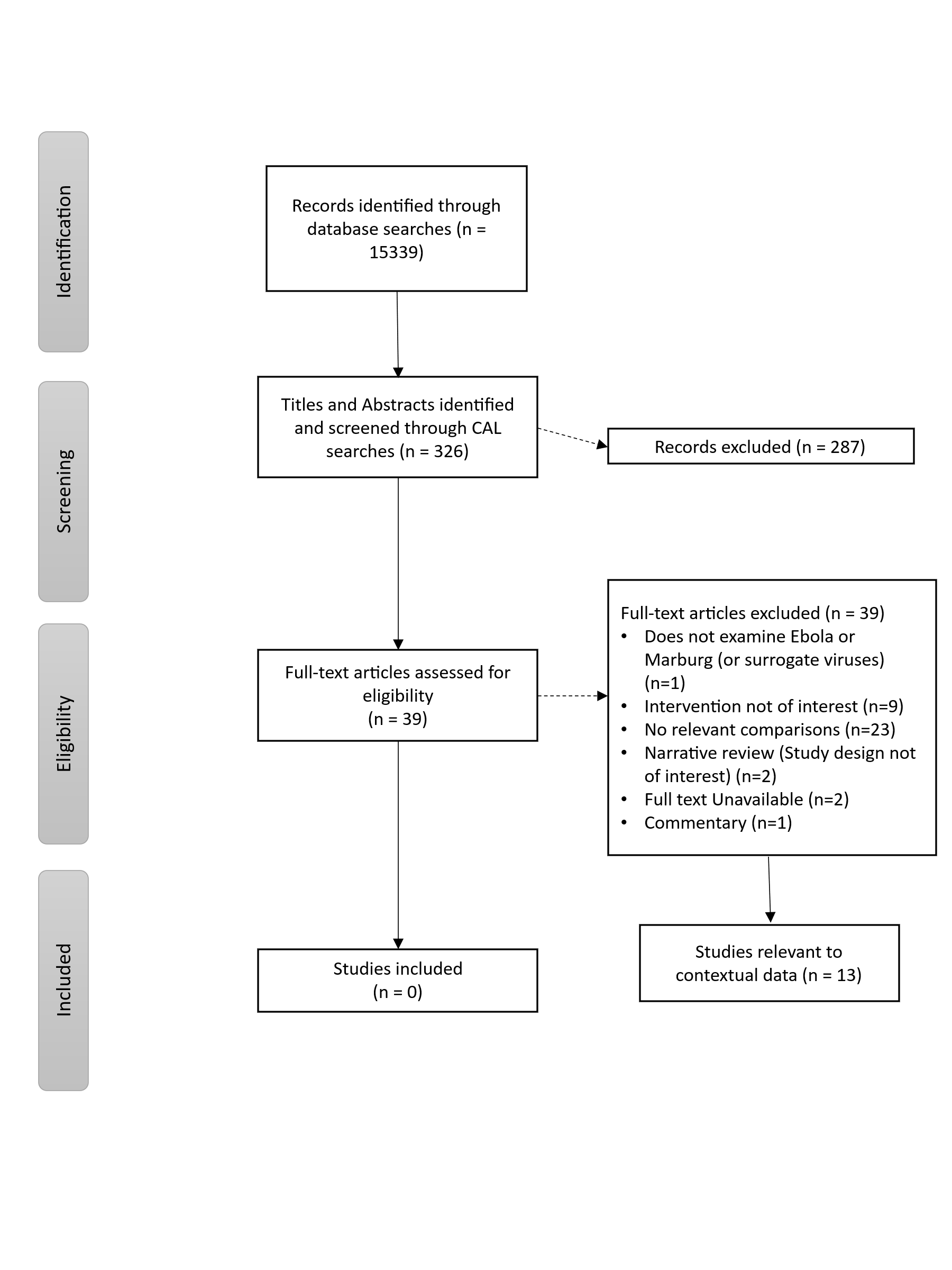


## KQ10 PRISMA Flow Diagram


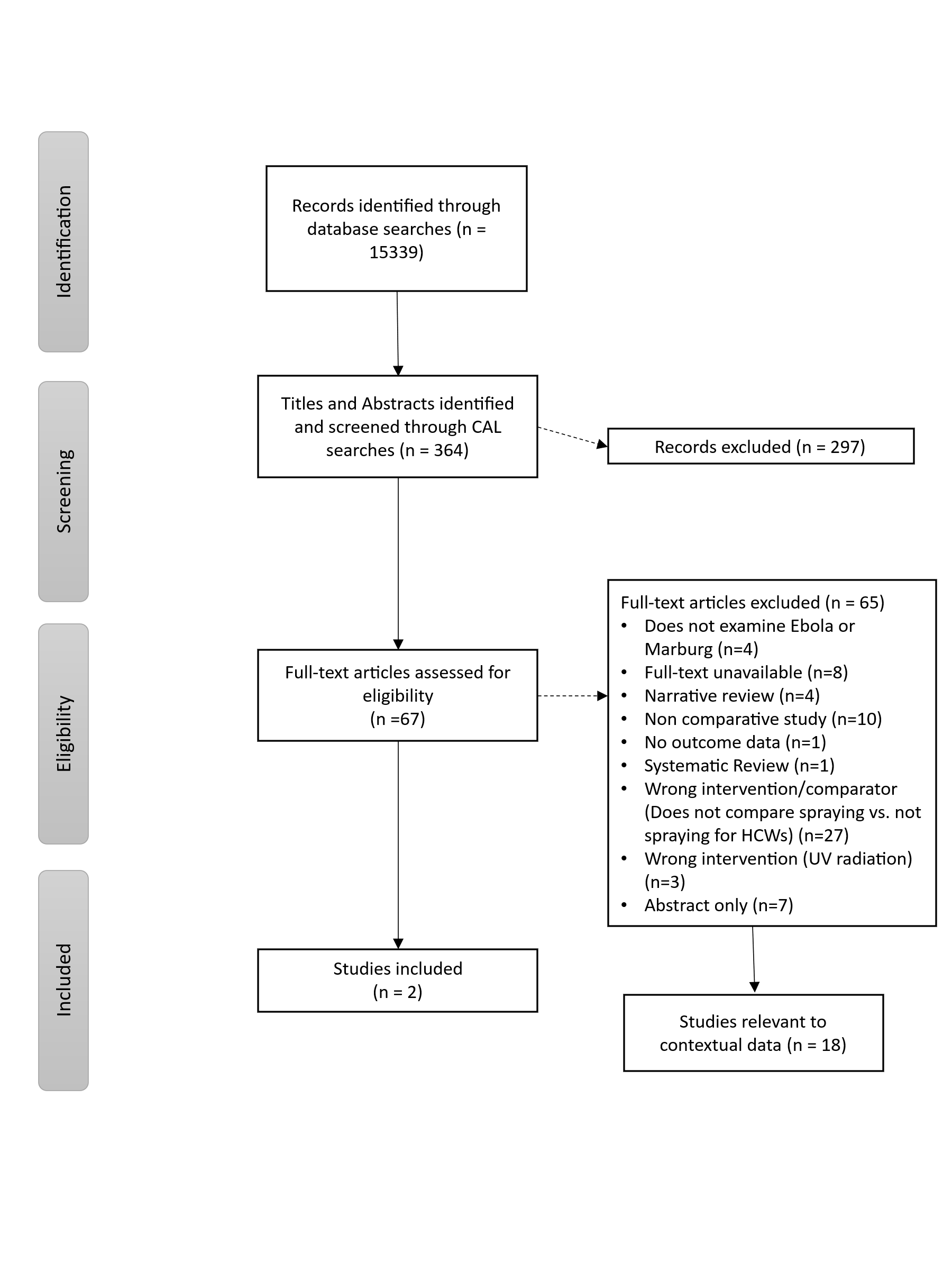


## KQ11 PRISMA Flow Diagram


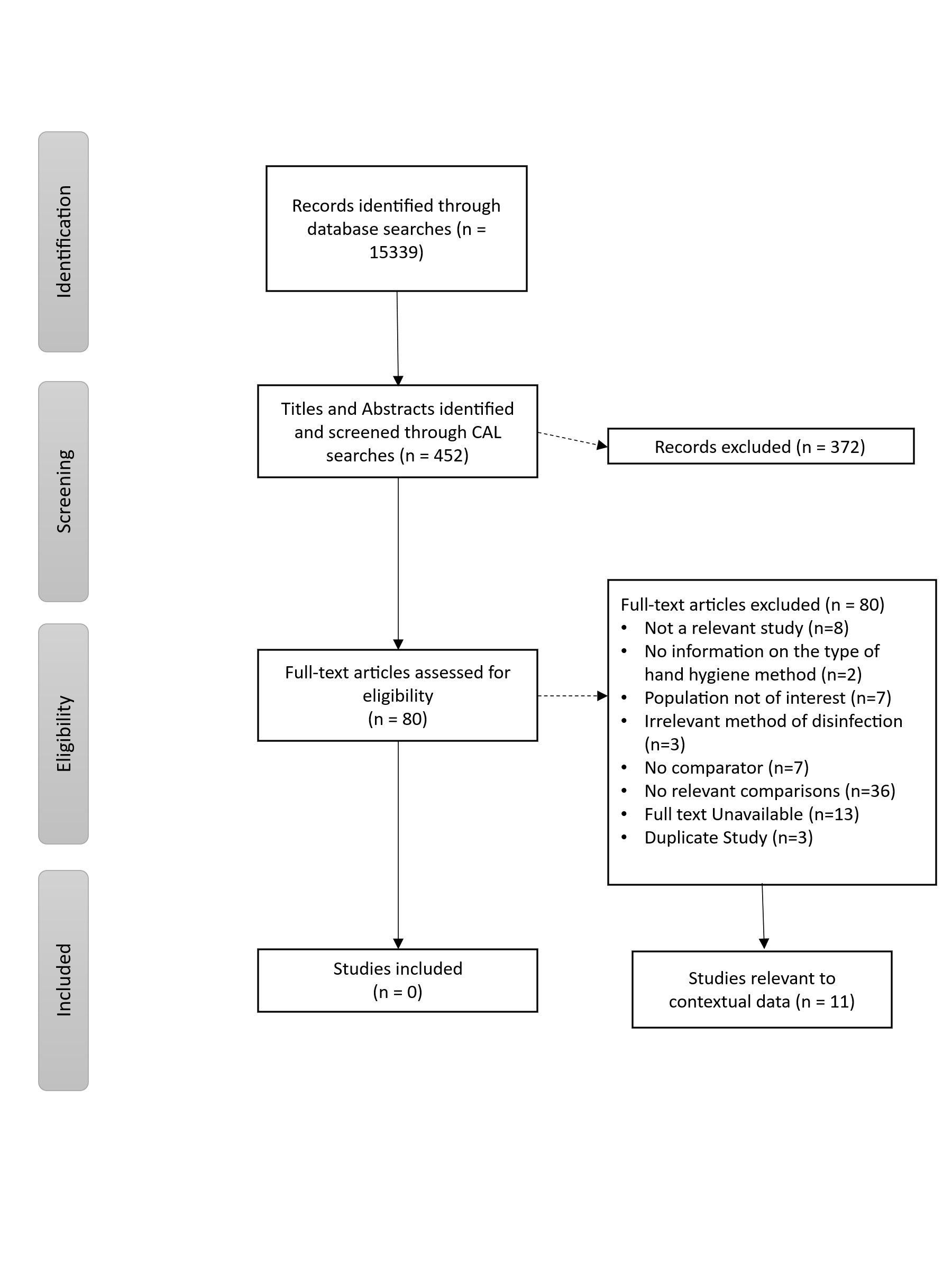


## KQ12 PRISMA Flow Diagram


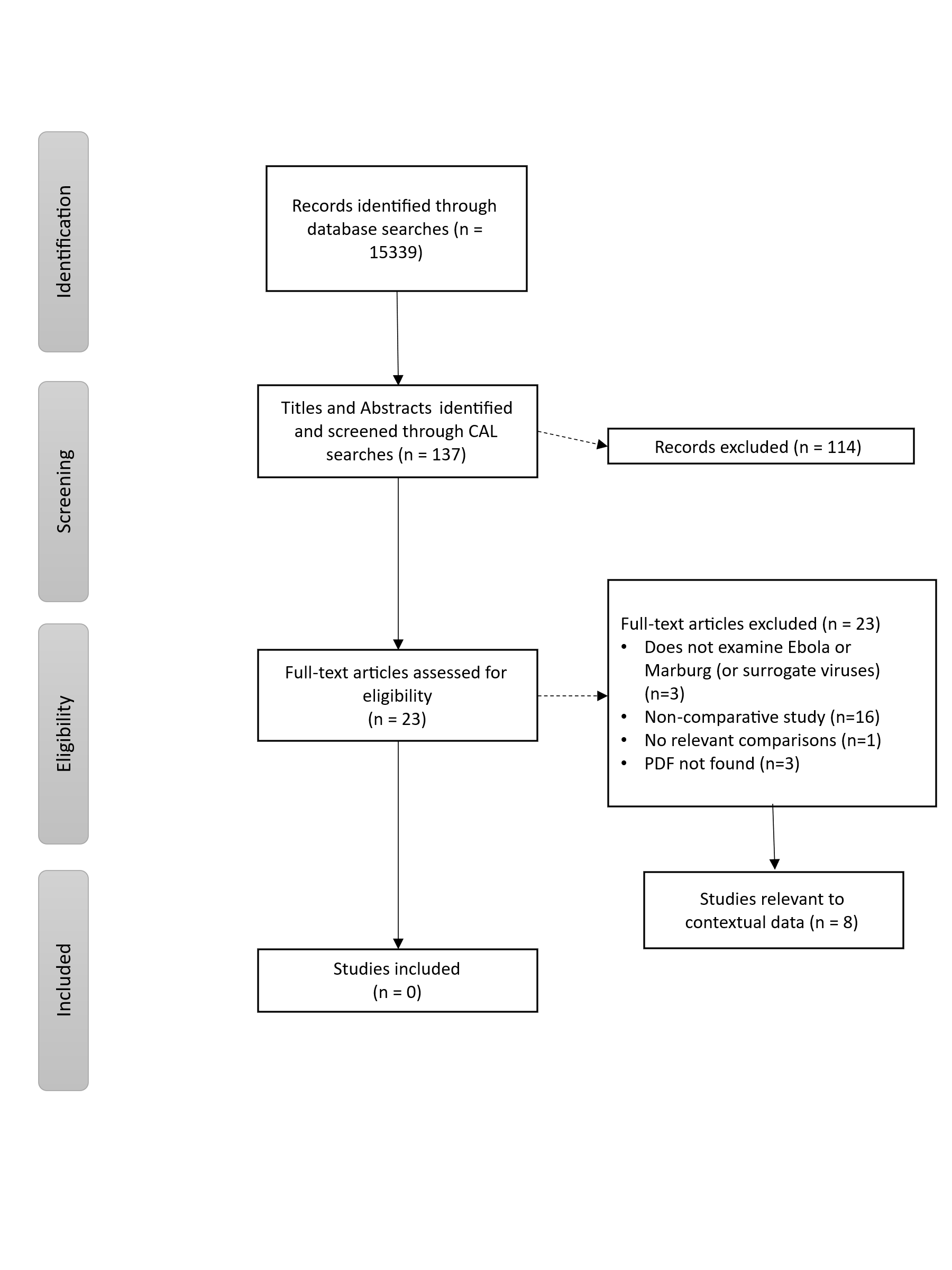


# Search Update 1 (KQ1) PRISMA Flow Diagram


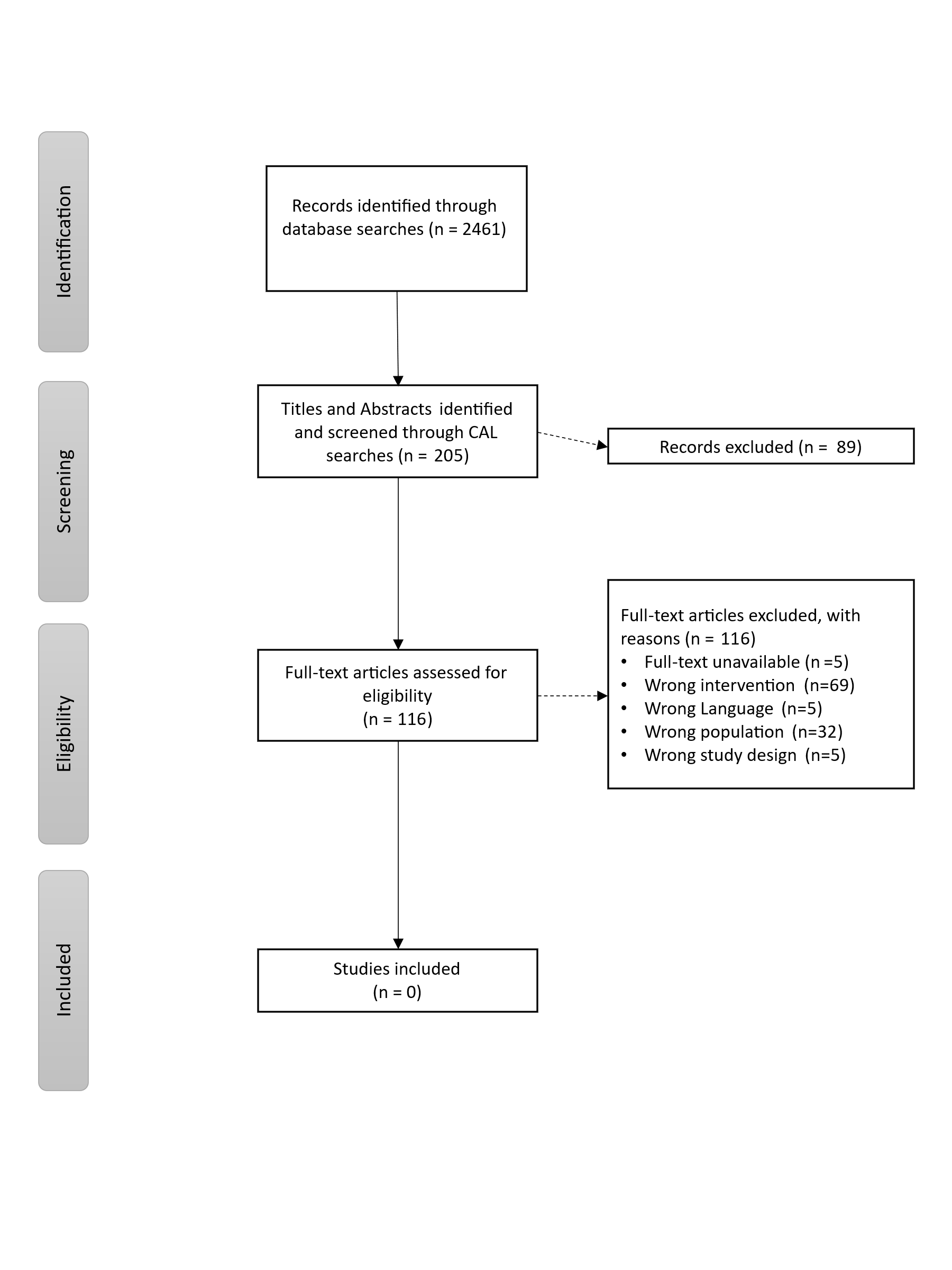


# Search Update 2 (KQ2, KQ9, KQ10) PRISMA Flow Diagram


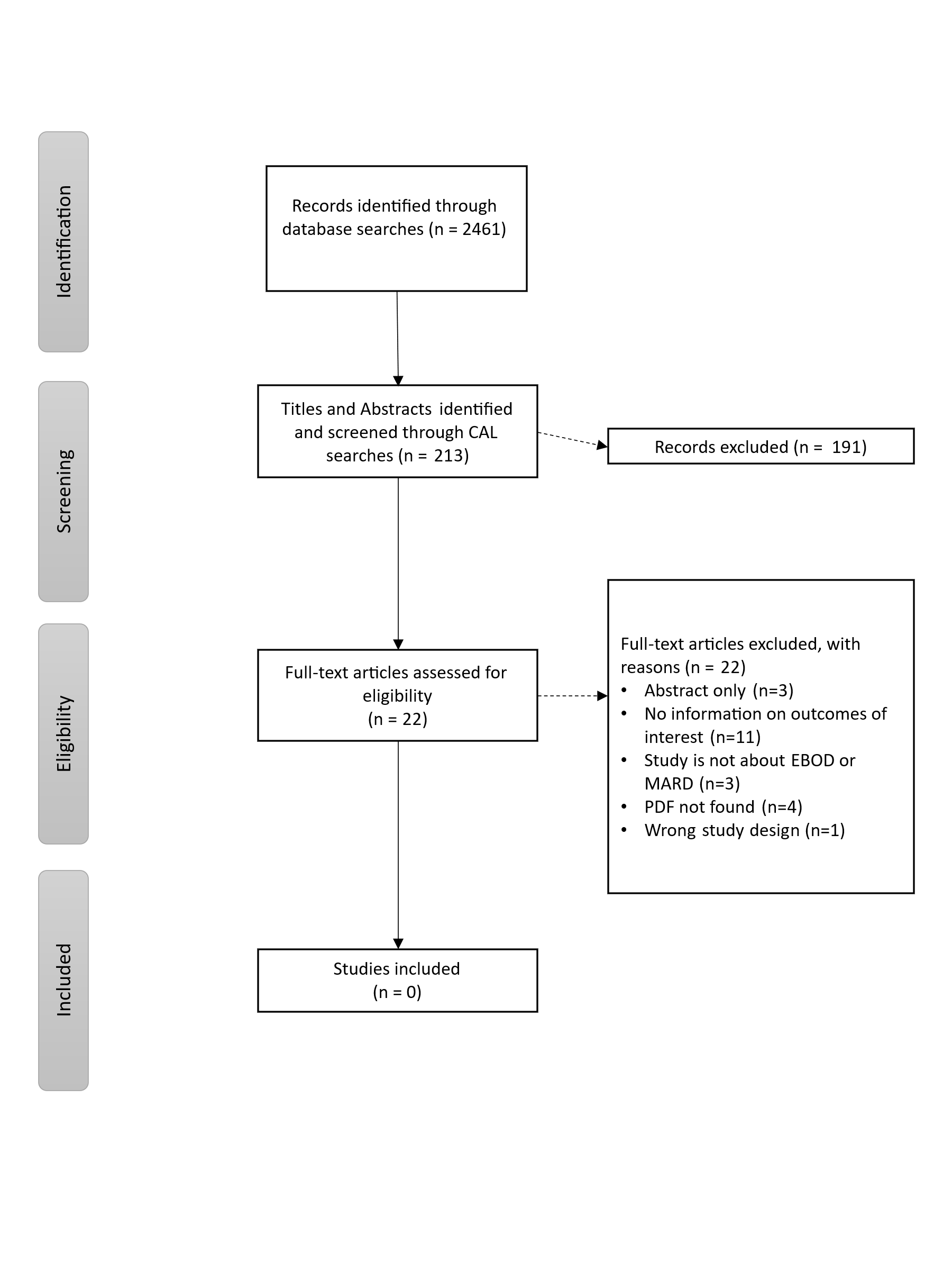


# Search Update 3 (KQ3) PRISMA Flow Diagram


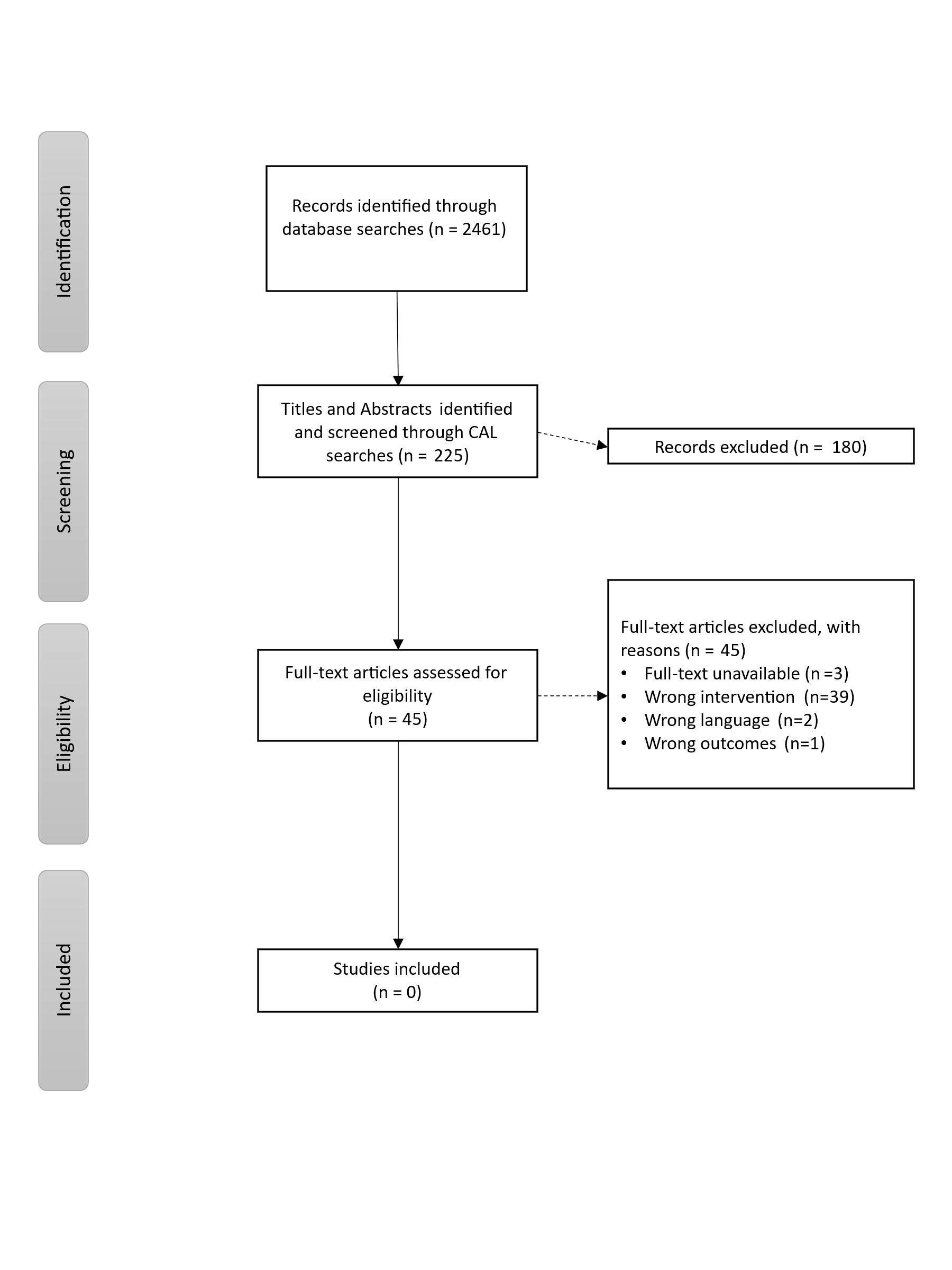


# Search Update 4 (KQ4, KQ5, KQ6, KQ7A, KQ7B, KQ8) PRISMA Flow Diagram


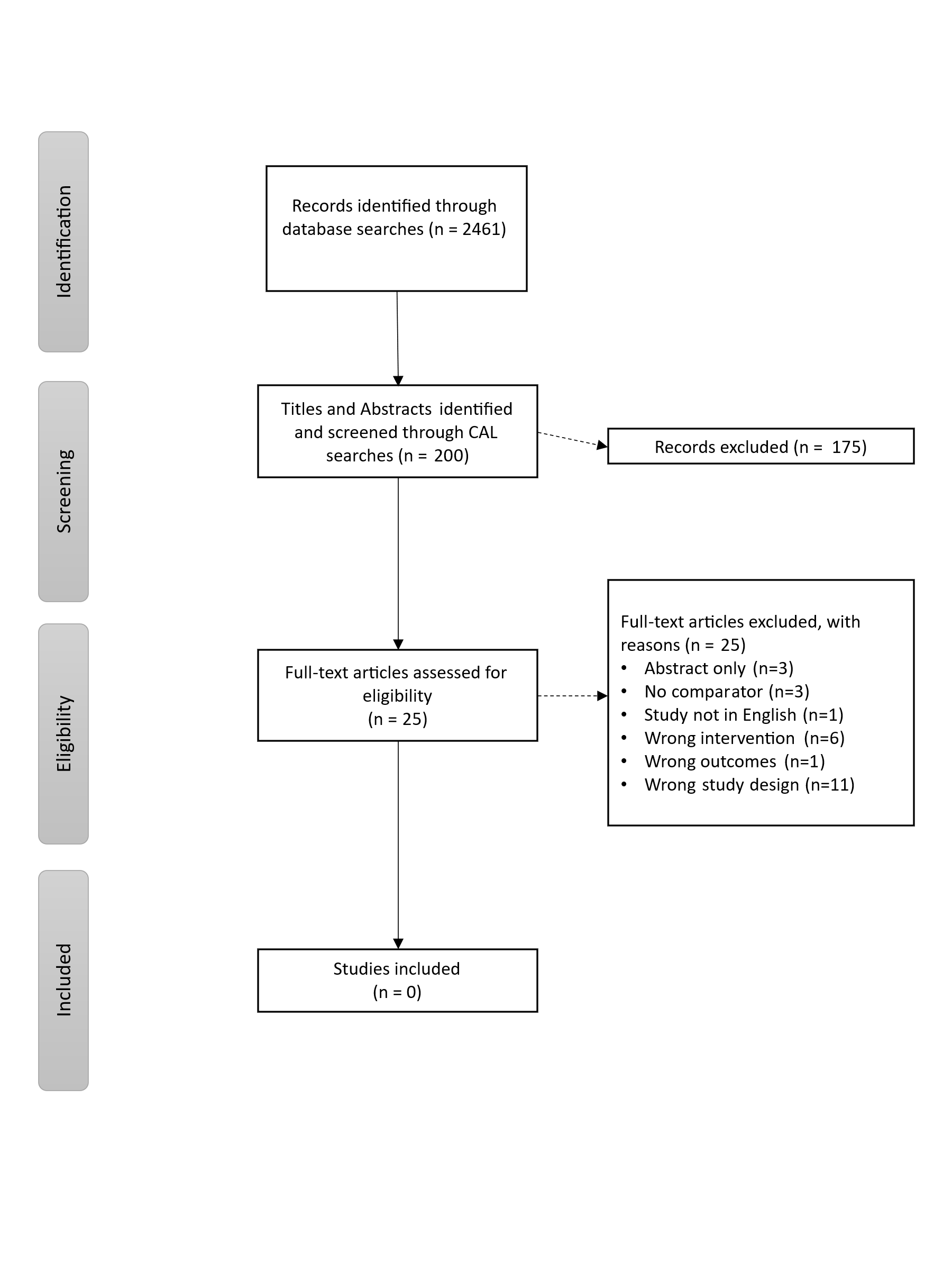


# Search Update 5 (KQ11A, KQ11B, KQ11C, Additional PICO 1, Additional PICO 2) PRISMA Flow Diagram


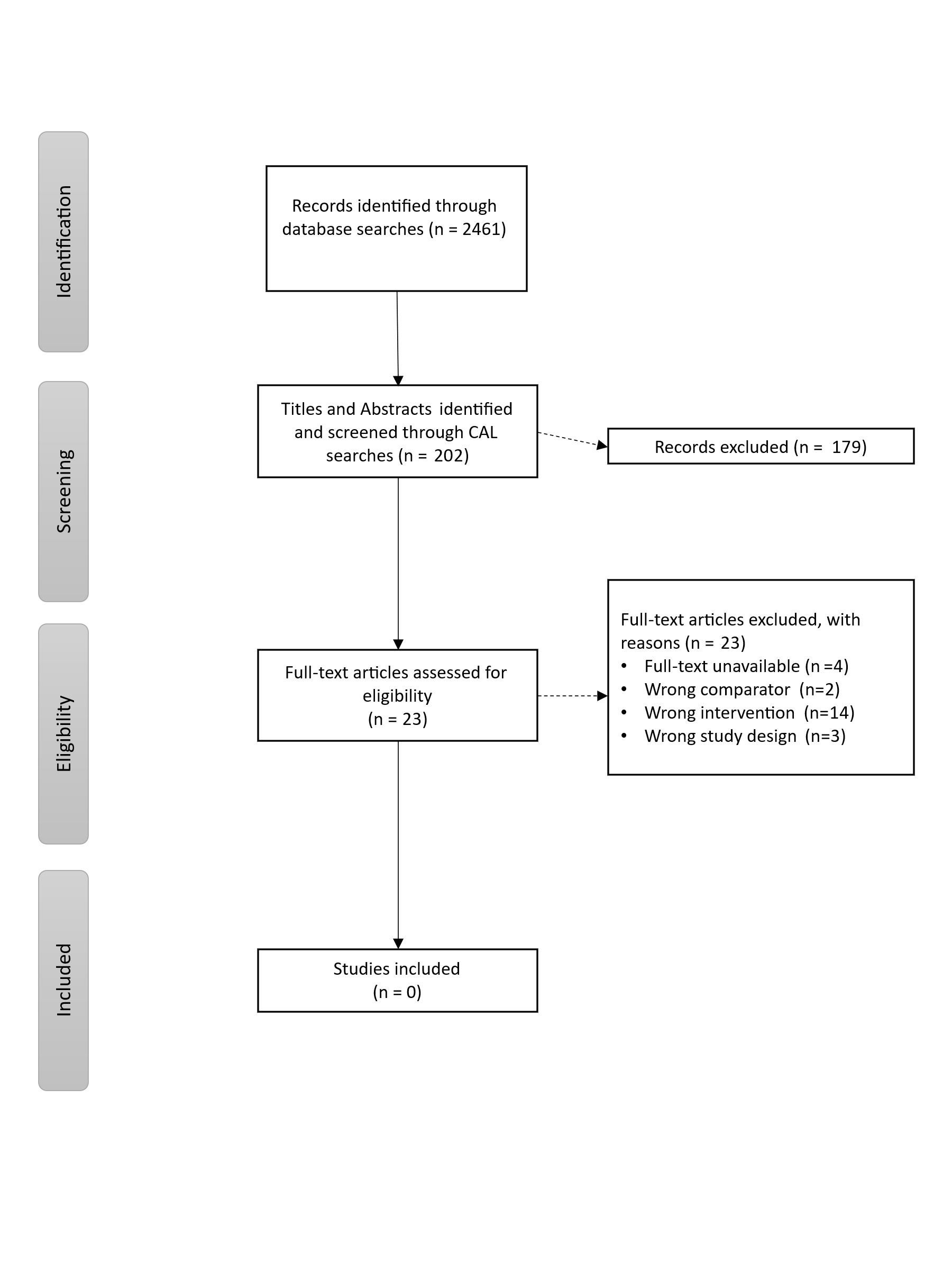


# Search Update 6 (KQ12) PRISMA Flow Diagram


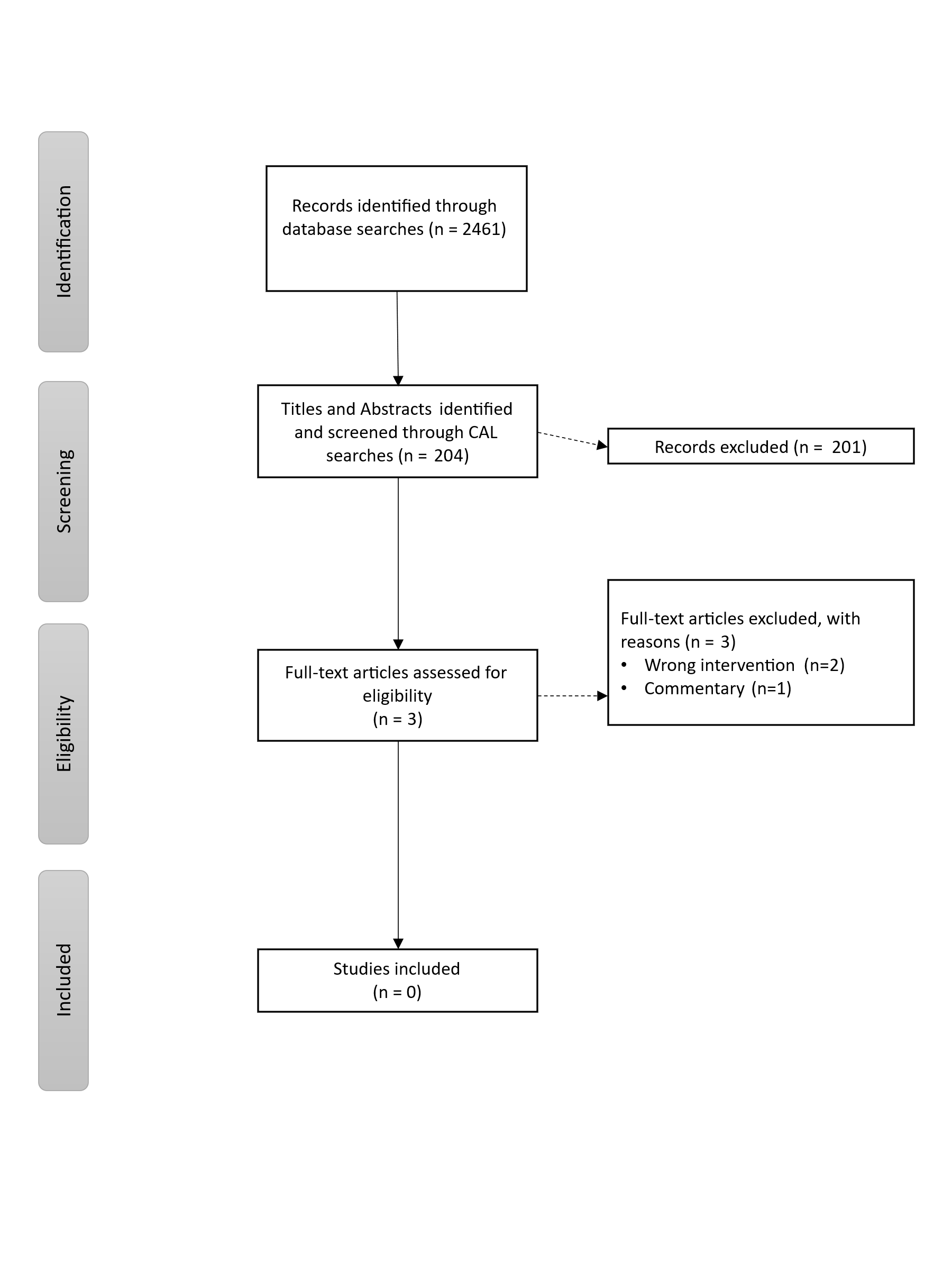

Supplement: online supplemental file 5 [file bmjopen-16-7-s005.docx]
