# Supplementary material for: Infection prevention and control measures for Ebola and Marburg disease: a series of rapid reviews
Source: BMJ Open. 2026 Jul 9;16(7):e115610. doi: 10.1136/bmjopen-2025-115610 (PMC13358256; doi:10.1136/bmjopen-2025-115610)
Supplement: online supplemental file 6 [file bmjopen-16-7-s006.docx]

**Supplementary file 6. List of Excluded Studies by KQ and Reason for Exclusion**

Contents

[Theme 1: Transmission/Exposure F2](#_Toc157695315)

[KQ1 F2](#_Toc157695316)

[KQ2 F6](#_Toc157695317)

[KQ3 F12](#_Toc157695318)

[Theme 2: Personal Protective Equipment (PPE) F15](#_Toc157695319)

[KQ4/KQ7 F15](#_Toc157695320)

[KQ5 F25](#_Toc157695321)

[KQ6 F30](#_Toc157695322)

[KQ8 F37](#_Toc157695323)

[Additional PICO 1 and 2 F42](#_Toc157695324)

[Theme 3: Disinfection/Decontamination F49](#_Toc157695325)

[KQ9 F49](#_Toc157695326)

[KQ10 F53](#_Toc157695327)

[KQ11 F59](#_Toc157695328)

[KQ12 F66](#_Toc157695329)

# Theme 1: Transmission/Exposure

# KQ1

**Does not provide risk of infection for HCWs for activities of interest**

Borchert M, Mulangu S, Lefèvre P, et al. Use of Protective Gear and the Occurrence of Occupational Marburg Hemorrhagic Fever in Health Workers from Watsa Health Zone, Democratic Republic of the Congo. J Infect Dis. 2007;196(s2):S168-S175. doi:10.1086/520540

Doshi RH, Hoff NA, Bratcher A, et al. Risk Factors for Ebola Exposure in Health Care Workers in Boende, Tshuapa Province, Democratic Republic of the Congo. The Journal of Infectious Diseases. Published online 2020:jiaa747. doi:10.1093/infdis/jiaa747

Dunn AC, Walker TA, Redd J, et al. Nosocomial transmission of Ebola virus disease on pediatric and maternity wards: Bombali and Tonkolili, Sierra Leone, 2014. American Journal of Infection Control. 2016;44(3):269-272. doi:10.1016/j.ajic.2015.09.016

Gozel MG, Dokmetas I, Oztop AY, Engin A, Elaldi N, Bakir M. Recommended precaution procedures protect healthcare workers from Crimean-Congo hemorrhagic fever virus. Int J Infect Dis. 2013;17(11):e1046-e1050. doi:10.1016/j.ijid.2013.05.005

Grinnell M, Dixon MG, Patton M, et al. Ebola Virus Disease in Health Care Workers — Guinea, 2014. MMWR Morb Mortal Wkly Rep. 2015;64(38):1083-1087. doi:10.15585/mmwr.mm6438a1

Hoff NA, Mwanza A, Doshi RH, et al. Possible high exposure to ebola among non-formal health care providers in a previous outbreak site boende democratic republic of congo. J Infect Dis. 2016;219:517-525.

Hoff NA, Mukadi P, Doshi RH, et al. Serologic Markers for Ebolavirus Among Healthcare Workers in the Democratic Republic of the Congo. The Journal of Infectious Diseases. 2019;219(4):517-525. doi:10.1093/infdis/jiy499

Lópaz MA, Amela C, Ordobas M, et al. First secondary case of Ebola outside Africa: epidemiological characteristics and contact monitoring, Spain, September to November 2014. Eurosurveillance. 2015;20(1). doi:10.2807/1560-7917.ES2015.20.1.21003

Matanock A, Arwady MA, Ayscue P, et al. Ebola Virus Disease Cases Among Health Care Workers Not Working in Ebola Treatment Units — Liberia, June–August, 2014. 2014;63(46):5.

Tomori O, Bertolli J, Rollin PE, et al. Serologic Survey among Hospital and Health Center Workers during the Ebola Hemorrhagic Fever Outbreak in Kikwit, Democratic Republic of the Congo, 1995. J Infect Dis. 1999;179(s1):S98-S101. doi:10.1086/514307

**Not about EVD or Marburg**

Guven G, Talan L, Altintas ND, Memikoglu KO, Yoruk F, Azap A. An Unexpected Fatal CCHF Case and Management of Exposed Health Care Workers. International Journal of Infectious Diseases. 2017;55:118-121. doi:10.1016/j.ijid.2016.12.026

**Study does not provide details on the exposure**

Adedire EB, Fatiregun A, Olayinka A, Sabitu K, Nguku P. Descriptive epidemiology of the EBOLA virus disease outbreak in Nigeria July to September 2014. 2015;Am J Tropic Med Hygiene.

Bausch DG. The Year That Ebola Virus Took Over West Africa: Missed Opportunities for Prevention. The American Journal of Tropical Medicine and Hygiene. 2015;92(2):229-232. doi:10.4269/ajtmh.14-0818

Doshi RH, Hoff NA, Mukadi P, et al. Seroprevalence of ebola virus among health care workers in the Tshuapa district democratic republic of congo. Am J Tropic Med Hygiene. Published online 2016.

**Study does not provide risk of infection by exposure**

Calkin S. British ebola nurse’s African colleague dies of the virus. Nursing Times. 2014;110(38).

Chevalier MS, Chung W, Smith J, et al. Ebola Virus Disease Cluster in the United States — Dallas County, Texas, 2014. :2.

Chung WM, Smith JC, Weil LM, et al. Active Tracing and Monitoring of Contacts Associated With the First Cluster of Ebola in the United States. Ann Intern Med. 2015;163(3):164-173. doi:10.7326/M15-0968

Clausen, L, Bothwell TH, Isaacson M, et al. Isolation and Handling of Patients with Dangerous Infectious Disease. S Afr med J. 1978;53(238):5.

Gear JSS, Cassel GA, Gear AJ, et al. Outbreak of Marburg virus disease in Johannesburg. BRITISH MEDICAL JOURNAL. Published online 1975:5.

Grinnell M, Dixon MG, Patton M, et al. Ebola Virus Disease in Health Care Workers — Guinea, 2014. MMWR Morb Mortal Wkly Rep. 2015;64(38):1083-1087. doi:10.15585/mmwr.mm6438a1

Musene KK, Hoff NA, Spencer D, et al. Occupational exposure of health care workers in kinshasa Democratic Republic of the Congo. Am J Tropic Med Hygiene. Published online 2018.

Nyenswah T, Fallah M, Sieh S, et al. Controlling the last known cluster of Ebola virus disease - Liberia, January-February 2015. MMWR Morb Mortal Wkly Rep. 2015;64(18):500-504.

Olu O, Kargbo B, Kamara S, et al. Epidemiology of Ebola virus disease transmission among health care workers in Sierra Leone, May to December 2014: a retrospective descriptive study. BMC Infect Dis. 2015;15(1):416. doi:10.1186/s12879-015-1166-7

Report of a WHO/International Study Team. Ebola haemorrhagic fever in Sudan, 1976. Report of a WHO/International Study Team. Bull World Health Organ. 1978;56(2):247-270.

Report of an International Commission. Ebola haemorrhagic fever in Zaire, 1976. Bull World Health Organ. 1978;56(2):271-293.

Senga M, Pringle K, Brett-Major D, et al. Largest documented cluster of ebola virus disease among health workers. Published online 2015.

Senga M, Pringle K, Ramsay A, et al. Factors Underlying Ebola Virus Infection Among Health Workers, Kenema, Sierra Leone, 2014–2015. Clin Infect Dis. 2016;63(4):454-459. doi:10.1093/cid/ciw327

**No data from health workers**

Olugbade OT, Israel O, Fadahunsi R, Ogunniyi A, Oladimeji A, Olayinka A. Outbreak of lassa fever in a healthcare setting : Epidemiologic surveillance and risk stratification for contact persons-Saki Oyo State Nigeria 2014. Antimicrob Resist Infect Contr. Published online 2017.

**Duplicate**

Borchert M, Mulangu S, Lefèvre P, et al. Use of Protective Gear and the Occurrence of Occupational Marburg Hemorrhagic Fever in Health Workers from Watsa Health Zone, Democratic Republic of the Congo. J Infect Dis. 2007;196(s2):S168-S175. doi:10.1086/520540

Kilmarx PH, Clarke KR, Dietz PM, et al. Ebola Virus Disease in Health Care Workers — Sierra Leone, 2014. 2014;63(49):4.

**Non-English**

Adachi T, Komiya N, Kato Y. Ebola Virus Disease Outbreak Response in West Africa. Kansenshogaku Zasshi. 2015;89(2):223-229. doi:doi: 10.11150/kansenshogakuzasshi.89.223.

**Wrong Intervention - does not evaluate work exclusion**

Benowitz I, Ackelsberg J, Balter SE, et al. Surveillance and Preparedness for Ebola Virus Disease—New York City, 2014. MMWR Morb Mortal Wkly Rep. 2015;15(1):278-280. doi:10.1111/ajt.13114

Biedron C, Lyman M, Stuckey MJ, et al. Evaluation of Infection Prevention and Control Readiness at Frontline Health Care Facilities in High-Risk Districts Bordering Ebola Virus Disease–Affected Areas in the Democratic Republic of the Congo — Uganda, 2018. MMWR Morb Mortal Wkly Rep. 2019;68(39):851-854. doi:10.15585/mmwr.mm6839a4

Desclaux A, Badji D, Ndione AG, Sow K. Accepted monitoring or endured quarantine? Ebola contacts’ perceptions in Senegal. Social Science & Medicine. 2017;178:38-45. doi:10.1016/j.socscimed.2017.02.009

Gatter R. Quarantine Controversy: Kaci Hickox v. Governor Chris Christie. Hastings Center Report. 2016;46(3):7-8. doi:10.1002/hast.584

Johnson O, Youkee D, Brown CS, et al. Ebola Holding Units at government hospitals in Sierra Leone: evidence for a flexible and effective model for safe isolation, early treatment initiation, hospital safety and health system functioning. BMJ Glob Health. 2016;1(1):e000030. doi:10.1136/bmjgh-2016-000030

Kilmarx PH, Clarke KR, Dietz PM, et al. Ebola Virus Disease in Health Care Workers — Sierra Leone, 2014. MMWR - Morbidity & Mortality Weekly Report. 2014;63(49). https://pubmed.ncbi.nlm.nih.gov/25503921/

O’Malley G, Narayan V, Gage G, John-Stewart G, Konjoh E, Speed C. Sierra Leone’s health workforce crisis: Drivers of suboptimal distribution and poor retention of primary healthcare workers in rural areas. Annals of Global Health. 2015;81(1):46. doi:10.1016/j.aogh.2015.02.614

Pathmanathan I, O’Connor KA, Adams ML, et al. Rapid Assessment of Ebola Infection Prevention and Control Needs — Six Districts, Sierra Leone, October 2014. MMWR Morb Mortal Wkly Rep. 2022;63(49).

Winters A, Iqbal M, Benowitz I, et al. Public Health Management of Persons Under Investigation for Ebola Virus Disease in New York City, 2014-2016. Public Health Rep. 2019;134(5):477-483. doi:10.1177/0033354919870200

**No intervention**

Cutsem GV. The Médecins Sans Frontières Experience With The Current Ebola Outbreaks. Topics in Antiviral Medicine. Published online 2015. doi:10.13140/RG.2.1.3524.1048

Folayan M, Haire B, Brown B. Critical role of ethics in clinical management and public health response to the West Africa Ebola epidemic. RMHP. Published online May 2016:55. doi:10.2147/RMHP.S83907

Yacisin K, Balter S, Fine A, et al. Ebola Virus Disease in a Humanitarian Aid Worker — New York City, October 2014. New York City. Published online 2014.

Yakubu A, Folayan MO, Sani-Gwarzo N, Nguku P, Peterson K, Brown B. The Ebola outbreak in Western Africa: ethical obligations for care. J Med Ethics. 2016;42(4):209-210. doi:10.1136/medethics-2014-102434

Exclusion reason: No comparator group

Desclaux A, Ndione AG, Badji D, Sow K. La surveillance des personnes contacts pour Ébola : effets sociaux et enjeux éthiques au Sénégal. *Bull Soc Pathol Exot*. 2016;109(4):296-302. doi:[10.1007/s13149-016-0477-2](https://doi.org/10.1007/s13149-016-0477-2)

Forrester JD, Pillai SK, Beer KD, et al. Assessment of Ebola Virus Disease, Health Care Infrastructure, and Preparedness — Four Counties, Southeastern Liberia, August 2014. *MMWR Morb Mortal Wkly Rep*. 2014;63(40). <https://www.ncbi.nlm.nih.gov/pmc/articles/PMC4584611/>

Reaves EJ, Mabande LG, Thoroughman DA, Arwady MA, Montgomery JM. Control of Ebola Virus Disease — Firestone District, Liberia, 2014. Published online 2014.

# KQ2

**Full text Unavailable**

Boardman A. Viral hemorrhagic fever. Primary Care Update for Ob/Gyns. Published online 2003.

Boumandouki P, Formenty P, Epelboin A, et al. [Clinical management of patients and deceased during the Ebola outbreak from October to December 2003 in Republic of Congo]. Bull Soc Pathol Exot. 2005;98(3):218-223.

Hoff NA, Mwanza A, Doshi RH, et al. Possible high exposure to ebola among non-formal health care providers in a previous outbreak site boende democratic republic of congo. The Journal of Infectious Diseases. 2016;219:517-525.

Kerstiëns B, Matthys F. Interventions to control virus transmission during an outbreak of Ebola hemorrhagic fever: experience from Kikwit, Democratic Republic of the Congo,  1995. J Infect Dis. 1999;179 Suppl 1:S263-267. doi:10.1086/514320

Klenk H. Marburg and Ebola viruses: Preface. Current Topics in Microbiology and Immunology. Published online 1998.

Kunii O, Kita E, Shibuya K. [Epidemics and related cultural factors for Ebola hemorrhagic fever in Gabon]. Nihon Koshu Eisei Zasshi. 2001;48(10):853-859.

McClelland A, Flemming J, Atchia V, Nugba-Ballah R. From dead body management to safe and dignified burials. Experiences of the Red Cross movement in managing safe and dignified burials in Guinea Liberia and Sierra Leone Ebola response. Tropical Medicine and International Health. 2015;20(1).

Miguel-Hernandez AS. International health alert: The new challenge by Ebola virus disease. [Spanish]. Gaceta Medica de Bilbao. Published online 2014.

Nkoghé D, Formenty P, Nnégué S, et al. [Practical guidelines for the management of Ebola infected patients in the field]. Med Trop (Mars). 2004;64(2):199-204.

Ondhia C, Levell N. Ebola: Diagnosis and Dermatology.; 2015:41.

Parzeller M, Wicker S, Rabenau H, Zehner R, Kettner M, Verhoff M. External examination of the corpse and autopsy in the times of Ebola: Medical and legal aspects. Published online 2015.

Splino M, Chlibek R. Continual of the tens ebola outbreak - Democratic Republic Congo 2018/2019. 2019. Vakcinologie.

**No comparisons of interest**

Bhatnagar N, Grover M, Kotwal A, Chauhan H. Study of recent Ebola virus outbreak and lessons learned: A scoping study. Ann Trop Med Public Health. 2016;9(3):145. doi:10.4103/1755-6783.181658

Blair RA, Morse BS, Tsai LL. Public health and public trust: Survey evidence from the Ebola Virus Disease epidemic in Liberia. Social Science & Medicine. 2017;172:89-97. doi:10.1016/j.socscimed.2016.11.016

Borchert M, Saez AM, Kratz T. A closer look at the Ebola outbreak in West Africa. Future Virology. 2015;10(5):483-490. doi:10.2217/fvl.15.46

Cordner S, Bouwer H, Tidball-Binz M. The Ebola epidemic in Liberia and managing the dead—A future role for Humanitarian Forensic Action? Forensic Science International. 2017;279:302-309. doi:10.1016/j.forsciint.2017.04.010

Curran KG, Gibson, JJ, MD, et al. Cluster of Ebola Virus Disease Linked to a Single Funeral — Moyamba District, Sierra Leone, 2014. MMWR Morb Mortal Wkly Rep. 2016;65(8):202-205. doi:10.15585/mmwr.mm6508a2

Fusco FM, Scappaticci L, Schilling S, et al. A 2009 cross-sectional survey of procedures for post-mortem management of highly infectious disease patients in 48 isolation facilities in 16 countries: data from EuroNHID. Infection. 2016;44(1):57-64. doi:10.1007/s15010-015-0831-5

Grover M, Bhatnagar N, Kotwal A, Chauhan H. Lessons learnt from a recent Ebola virus outbreak: A scoping study. International Journal of Infectious Diseases. 2016;45:174. doi:10.1016/j.ijid.2016.02.410

Hagan JE, Smith W, Pillai SK, et al. Implementation of Ebola Case-Finding Using a Village Chieftaincy Taskforce in a Remote Outbreak — Liberia, 2014. 2015;64(7):3.

Herstein JJ, Biddinger PD, Gibbs SG, et al. High-Level Isolation Unit Infection Control Procedures. Health Security. 2017;15(5):519-526. doi:10.1089/hs.2017.0026

Heymann DL. Ebola: learn from the past. Nature. 2014;514(7522):299-300. doi:10.1038/514299a

Heymann DL. Ebola: burying the bodies. The Lancet. 2015;386(10005):1729-1730. doi:10.1016/S0140-6736(15)00684-4

Kent W. Ebola in Western Africa. *Future Virology*. 2015;10(3). doi:<https://doi.org/10.2217/fvl.14.105>

Lever RA, Whitty CJM. Ebola virus disease: emergence, outbreak and future directions. Br Med Bull. 2016;117(1):95-106. doi:10.1093/bmb/ldw005

Lorente JÁ, Blanch L, Esteban A. Ebola Virus: Understanding the 2014 Outbreak. Archivos de Bronconeumología (English Edition). 2015;51(2):59-60. doi:10.1016/j.arbr.2014.12.025

McClelland A, Flemming J, Atchia V, Nugba-Ballah R. From dead body management to safe and dignified burials. Experiences of the Red Cross movement in managing safe and dignified burials in Guinea Liberia and Sierra Leone Ebola response. Tropical Medicine and International Health. 2015;20(1).

Mokuwa E, Richards P. How Should Public Health Officials Respond When Important Local Rituals Increase Risk of Contagion? AMA Journal of Ethics. 2020;22(1):E5-9. doi:10.1001/amajethics.2020.5

Musong M, Muyembe T, Kibasa. Update: Outbreak of Ebola Viral Hemorrhagic Fever Zaire,1995. JAMA. 274(5).

Muyembe‐Tamfum JJ, Kipasa M, Kiyungu C, Colebunders R. Ebola Outbreak in Kikwit, Democratic Republic of the Congo: Discovery and Control Measures. J INFECT DIS. 1999;179(s1):S259-S262. doi:10.1086/514302

Namahoro J, Hogan U. A surveillance and control of Ebola Outbreak Disease at Télimélé, Guinea Conakry 2014. Antimicrob Resist Infect Control. 2015;4(S1):P3, 2047-2994-4-S1-P3. doi:10.1186/2047-2994-4-S1-P3

Nielsen CF, Kidd S, Sillah ARM, Davis E, Mermin J, Kilmarx PH. Improving Burial Practices and Cemetery Management During an Ebola Virus Disease Epidemic — Sierra Leone, 2014. 2015;64(1):8.

Raabe V, Borchert M. Infection control during filoviral hemorrhagic fever outbreaks. *J Global Infect Dis*. 2012;4(1):69. doi:[10.4103/0974-777X.93765](https://doi.org/10.4103/0974-777X.93765)

Suwalowska H, Amara F, Roberts N, Kingori P. Ethical and sociocultural challenges in managing dead bodies during epidemics and natural disasters. BMJ Glob Health. 2021;6(11):e006345. doi:10.1136/bmjgh-2021-006345

The Ebola Gbalo Research Group. Responding to the Ebola virus disease outbreak in DR Congo: when will we learn from Sierra Leone? The Lancet. 2019;393(10191):2647-2650. doi:10.1016/S0140-6736(19)31211-5

Tiffany A, Dalziel BD, Kagume Njenge H, et al. Estimating the number of secondary Ebola cases resulting from an unsafe burial and risk factors for transmission during the West Africa Ebola epidemic. Akogun OB, ed. PLoS Negl Trop Dis. 2017;11(6):e0005491. doi:10.1371/journal.pntd.0005491

Tiffany A, Dalziel B, Johnson G, Bedford J, McClelland A. Quantification of the impact of safe and dignified burials during the 2013-2016 west African ebola virus disease epidemic. Published online 2016.

**No outcomes of interest**

Sikakulya FK, Ilumbulumbu MK, Djuma SF, Bunduki GK, Sivulyamwenge AK, Jones MK. Safe and dignified burial of a deceased from a highly contagious infectious disease ebolavirus: Socio-cultural and anthropological implications in the Eastern DR Congo. One Health. 2021;13:100309. doi:10.1016/j.onehlt.2021.100309

**Study is not about health workers or burial teams**

Anonymous. Ebola outbreak update reported in MMWR. Journal of Environmental Health. 1995;58(1).

Anonymous. Death toll from suspected Ebola reaches 51 in Congo. Clinical Infectious Diseases. 2003;36.

Anonymous. Congo death toll from Ebola outbreak rises to 29. Clinical Infectious Diseases. 2004;38.

Anonymous. Ebola virus death toll hits 30 in central Africa. Clinical Infectious Diseases. 2002;34.

Dixon MG, Schafer IJ. Ebola Viral Disease Outbreak — West Africa, 2014. Morbidity and Mortality Weekly Report. 2014;63(25):20.

Halfmann P, Neumann G, Feldmann H, Kawaoka Y. Ebola Conquers West Africa — More to Come? EBioMedicine. 2014;1(1):2-3. doi:10.1016/j.ebiom.2014.10.004

Klenk HD. Lessons to be learned from the ebolavirus outbreak in West Africa. Emerging Microbes & Infections. 2014;3(1):1-1. doi:10.1038/emi.2014.68

Study is not about disinfection of bodies of deceased patients

Blumberg L. Viral hemorrhagic fevers: Ebola and beyond. International Journal of Infectious Diseases. 2016;45:8. doi:10.1016/j.ijid.2016.02.049

Brainard J, Hooper L, Pond K, Edmunds K, Hunter PR. Risk factors for transmission of Ebola or Marburg virus disease: a systematic review and meta-analysis. Int J Epidemiol. 2016;45(1):102-116. doi:10.1093/ije/dyv307

Cherif MS, Dumre SP, Kassé D, et al. Prognostic and Predictive Factors of Ebola Virus Disease Outcome in Elderly People during the 2014 Outbreak in Guinea. The American Journal of Tropical Medicine and Hygiene. 2018;98(1):198-202. doi:10.4269/ajtmh.17-0372

Curran KG, Gibson, JJ, MD, et al. Cluster of Ebola Virus Disease Linked to a Single Funeral — Moyamba District, Sierra Leone, 2014. MMWR Morb Mortal Wkly Rep. 2016;65(8):202-205. doi:10.15585/mmwr.mm6508a2

Dallatomasina S, Crestani R, Sylvester Squire J, et al. Ebola outbreak in rural West Africa: epidemiology, clinical features and outcomes. Trop Med Int Health. 2015;20(4):448-454. doi:10.1111/tmi.12454

Diallo MSK, Rabilloud M, Ayouba A, et al. Prevalence of infection among asymptomatic and paucisymptomatic contact persons exposed to Ebola virus in Guinea: a retrospective, cross-sectional observational study. The Lancet Infectious Diseases. 2019;19(3):308-316. doi:10.1016/S1473-3099(18)30649-2

Do TS, Lee YS. Modeling the Spread of Ebola. Osong Public Health and Research Perspectives. 2016;7(1):43-48. doi:10.1016/j.phrp.2015.12.012

Doshi RH, Hoff NA, Mukadi P, et al. Seroprevalence of ebola virus among health care workers in the Tshuapa district democratic republic of congo. Am J Tropic Med Hygiene. Published online 2016.

Dowell SF, Mukunu R, Ksiazek TG, et al. Transmission of Ebola Hemorrhagic Fever: A Study of Risk Factors in Family Members, Kikwit, Democratic Republic of the Congo, 1995. J INFECT DIS. 1999;179(s1):S87-S91. doi:10.1086/514284

Fang LQ, Yang Y, Jiang JF, et al. Transmission dynamics of Ebola virus disease and intervention effectiveness in Sierra Leone. Proc Natl Acad Sci USA. 2016;113(16):4488-4493. doi:10.1073/pnas.1518587113

Faye O, Boëlle PY, Heleze E, et al. Chains of transmission and control of Ebola virus disease in Conakry, Guinea, in 2014: an observational study. The Lancet Infectious Diseases. 2015;15(3):320-326. doi:10.1016/S1473-3099(14)71075-8

Hersey, S, Martel, LD, Jambai, A, et al. Ebola Virus Disease — Sierra Leone and Guinea, August 2015. MMWR Morb Mortal Wkly Rep. 2015;64(35). doi:10.15585/mmwr.mm6435a2

Houlihan CF, McGowan C, Roberts C, et al. Antibodies to ebola in international responders to the west africa ebola epidemic. Journal of Tropical Medicine and Hygiene. Published online 2016.

Jalloh MF, Robinson SJ, Corker J, et al. Knowledge, Attitudes, and Practices Related to Ebola Virus Disease at the End of a National Epidemic — Guinea, August 2015. MMWR Morb Mortal Wkly Rep. 2017;66(41):1109-1115. doi:10.15585/mmwr.mm6641a4

Jalloh MF, Sengeh P, Bunnell RE, et al. Evidence of behaviour change during an Ebola virus disease outbreak, Sierra Leone. Bull World Health Organ. 2020;98(5):330-340B. doi:10.2471/BLT.19.245803

Ka D, Fall G, Diallo VC, et al. Ebola Virus Imported from Guinea to Senegal, 2014. Emerg Infect Dis. 2017;23(6):1026-1028. doi:10.3201/eid2306.161092

Karwowski, Mateusz P, Meites, Elissa, Fullerton, Kathleen E, et al. Clinical Inquiries Regarding Ebola Virus Disease Received by CDC United States, July 9–November 15, 2014. MMWR - Morbidity & Mortality Weekly Report. 2014;63(49).

Keïta M, Conté F, Diallo B, et al. Lessons learned by surveillance during the tail-end of the Ebola outbreak in Guinea, June-October 2015: a case series. BMC Infect Dis. 2017;17(1):304. doi:10.1186/s12879-017-2405-x

Knust B, Schafer IJ, Wamala J, et al. Multidistrict Outbreak of Marburg Virus Disease—Uganda, 2012. J Infect Dis. 2015;212(suppl 2):S119-S128. doi:10.1093/infdis/jiv351

Lokuge K, Caleo G, Greig J, et al. Successful Control of Ebola Virus Disease: Analysis of Service Based Data from Rural Sierra Leone. Akogun OB, ed. PLoS Negl Trop Dis. 2016;10(3):e0004498. doi:10.1371/journal.pntd.0004498

Merler S, Ajelli M, Fumanelli L, et al. Spatiotemporal spread of the 2014 outbreak of Ebola virus disease in Liberia and the effectiveness of non-pharmaceutical interventions: a computational modelling analysis. The Lancet Infectious Diseases. 2015;15(2):204-211. doi:10.1016/S1473-3099(14)71074-6

Miglietta A, Solimini A, Djeunang Dongho GB, et al. The Ebola virus disease outbreak in Tonkolili district, Sierra Leone: a retrospective analysis of the Viral Haemorrhagic Fever surveillance system, July 2014–June 2015. Epidemiol Infect. 2019;147:e103. doi:10.1017/S0950268819000177

Mokuwa, E, Richards, P. How Should Public Health Officials Respond When Important Local Rituals Increase Risk of Contagion? AMA Journal of Ethics. 2020;22(1):E5-9. doi:10.1001/amajethics.2020.5

Namahoro J, Hogan U. A surveillance and control of Ebola Outbreak Disease at Télimélé, Guinea Conakry 2014. Antimicrob Resist Infect Control. 2015;4(S1):P3, 2047-2994-4-S1-P3. doi:10.1186/2047-2994-4-S1-P3

Nielsen CF, Kidd S, Sillah ARM, Davis E, Mermin J, Kilmarx PH. Improving Burial Practices and Cemetery Management During an Ebola Virus Disease Epidemic — Sierra Leone, 2014. 2015;64(1).

Nyakarahuka L, Schafer IJ, Balinandi S, et al. A retrospective cohort investigation of seroprevalence of Marburg virus and ebolaviruses in two different ecological zones in Uganda. BMC Infect Dis. 2020;20(1):461. doi:10.1186/s12879-020-05187-0

Pandey A, Atkins KE, Medlock J, et al. Strategies for containing Ebola in West Africa. Science. 2014;346(6212):991-995. doi:10.1126/science.1260612

Weppelmann TA, Donewell B, Haque U, et al. Determinants of patient survival during the 2014 Ebola Virus Disease outbreak in Bong County, Liberia. glob health res policy. 2016;1(1):5. doi:10.1186/s41256-016-0005-8

Yin X, Buyuktahtakın E. Risk-Averse Multi-Stage Stochastic Programming to Optimizing Vaccine Allocation and Treatment Logistics for Effective Epidemic Response. IISE Transactions on Healthcare Systems Engineering. Published online 2021.

Non-comparative

Tiffany A, Dalziel BD, Kagume Njenge H, et al. Estimating the number of secondary Ebola cases resulting from an unsafe burial and risk factors for transmission during the West Africa Ebola epidemic. Akogun OB, ed. *PLoS Negl Trop Dis*. 2017;11(6):e0005491. doi:[10.1371/journal.pntd.0005491](https://doi.org/10.1371/journal.pntd.0005491)

# KQ3

**Study does not evaluate the IPC ring approach for controlling the transmission of EVD/Marburg disease**

Arkell P, Youkee D, Brown CS, et al. Quantifying the risk of nosocomial infection within Ebola Holding Units: a retrospective cohort study of negative patients discharged from five Ebola Holding Units in Western Area, Sierra Leone. Trop Med Int Health. 2017;22(1):32-40. doi:10.1111/tmi.12802

Bangura I, Conteh C. The Impact of Quality Improvement Methodology to Improve Infection Control Practices. Antimicrobial Resistance & Infection Control. 2019;8(1):P405.

Bemah P, Baller A, Cooper C, et al. Strengthening healthcare workforce capacity during and post Ebola outbreaks in Liberia: an innovative and effective approach to epidemic preparedness and response. Pan Afr Med J. 2019;33. doi:10.11604/pamj.supp.2019.33.2.17619

Biedron C, Lyman M, Stuckey MJ, et al. Evaluation of Infection Prevention and Control Readiness at Frontline Health Care Facilities in High-Risk Districts Bordering Ebola Virus Disease–Affected Areas in the Democratic Republic of the Congo — Uganda, 2018. MMWR Morb Mortal Wkly Rep. 2019;68(39):851-854. doi:10.15585/mmwr.mm6839a4

Biedron C, Lyman M, Stuckey MJ, et al. Evaluation of Infection Prevention and Control Readiness at Frontline Health Care Facilities in High-Risk Districts Bordering Ebola Virus Disease–Affected Areas in the Democratic Republic of the Congo — Uganda, 2018. MMWR Morb Mortal Wkly Rep. 2019;68(39):851-854. doi:10.15585/mmwr.mm6839a4

Buregyeya E. Leveraging ebola viral disease emergency preparedness for infection prevention and control in health care facilities. International Journal of Infectious Diseases. 2020;101:318. doi:10.1016/j.ijid.2020.09.829

Cooper C. Using Data to Enhance Implementation in a Low Resource Setting - Liberia Experience. Antimicrobial Resistance & Infection Control. 2017;6(Supp 3):175.

Forrester JD, Hunter JC, Pillai SK, et al. Cluster of Ebola Cases Among Liberian and U.S. Health Care Workers in an Ebola Treatment Unit and Adjacent Hospital — Liberia, 2014. 2014;63(41):5.

Garde DL, Hall AMR, Marsh RH, Barron KP, Dierberg KL, Koroma AP. Implementation of the first dedicated Ebola screening and isolation for maternity patients in Sierra Leone. Annals of Global Health. 2016;82(3):418. doi:10.1016/j.aogh.2016.04.164

Janke C, Heim KM, Steiner F, et al. Beyond Ebola treatment units: severe infection temporary treatment units as an essential element of Ebola case management during an outbreak. BMC Infect Dis. 2017;17(1):124. doi:10.1186/s12879-017-2235-x

Johnson O, Youkee D, Brown CS, et al. Ebola Holding Units at government hospitals in Sierra Leone: evidence for a flexible and effective model for safe isolation, early treatment initiation, hospital safety and health system functioning. BMJ Glob Health. 2016;1(1):e000030. doi:10.1136/bmjgh-2016-000030

Keïta M, Camara AY, Traoré F, et al. Impact of infection prevention and control training on health facilities during the Ebola virus disease outbreak in Guinea. BMC Public Health. 2018;18(1):547. doi:10.1186/s12889-018-5444-3

Matanock A, Arwady MA, Ayscue P, et al. Ebola Virus Disease Cases Among Health Care Workers Not Working in Ebola Treatment Units — Liberia, June–August, 2014. 2014;63(46):5.

Mehtar S. The impact of education on reducing Ebola virus disease transmission in healthcare facilities. International Journal of Infectious Diseases. 2016;45:66-67. doi:10.1016/j.ijid.2016.02.193

Nanziri C, Ario AR, Ntono V, et al. Ebola Virus Disease Preparedness Assessment and Risk Mapping in Uganda, August-September 2018. Health Security. 2020;18(2):105-113. doi:10.1089/hs.2019.0118

Nyenswah T, Blackley DJ, Freeman T, et al. Community Quarantine to Interrupt Ebola Virus Transmission — Mawah Village, Bong County, Liberia, August–October, 2014. MMWR Morb Mortal Wkly Rep. 2015;64(7).

Nyenswah TG, Westercamp M, Kamali AA, et al. Evidence for Declining Numbers of Ebola Cases — Montserrado County, Liberia, June–October 2014. MMWR Morb Mortal Wkly Rep. 2023;63(46).

Oji MO, Haile M, Baller A, et al. Implementing infection prevention and control capacity building strategies within the context of Ebola outbreak in a “Hard-to-Reach” area of Liberia. Pan Afr Med J. 2018;31. doi:10.11604/pamj.2018.31.107.15517

Pathmanathan I, O’Connor KA, Adams ML, et al. Rapid Assessment of Ebola Infection Prevention and Control Needs — Six Districts, Sierra Leone, October 2014. MMWR Morb Mortal Wkly Rep. 2023;63(49).

Pronyk P, Rogers B, Lee S, et al. The Effect of Community-Based Prevention and Care on Ebola Transmission in Sierra Leone. Am J Public Health. 2016;106(4):727-732. doi:10.2105/AJPH.2015.303020

Reaves EJ, Mabande LG, Thoroughman DA, Arwady MA, Montgomery JM. Control of Ebola Virus Disease — Firestone District, Liberia, 2014. MMWR Morb Mortal Wkly Rep. 2023;63(42).

Rubin J, Hill ME. Implementing a High-Consequence Infectious Disease Preparedness Program at an Urban Community Hospital. American Journal of Infection Control. 2020;48(8):S44-S45. doi:10.1016/j.ajic.2020.06.050

Russo N, Archer M, Kinzie L, Pfeiffer CD. Beyond Ebola: Standardizing the Approach to High Consequence Infection Preparation. American Journal of Infection Control. 2018;46(6):S110-S111. doi:10.1016/j.ajic.2018.04.196

Tremblay N, Musa E, Cooper C. Infection prevention and control in health facilities in post-Ebola Liberia: don’t forget the private sector! Public Health Action.:6.

Weah VD, Doedeh JS, Wiah SQ, Nyema E, Lombeh S, Naiene J. Enhancing Ebola Virus Disease Surveillance and Prevention in Counties Without Confirmed Cases in Rural Liberia: Experiences from Sinoe County During the Flare-up in Monrovia, April to June, 2016. PLoS Curr. Published online 2017. doi:10.1371/currents.outbreaks.2b7f352af0866accbd7e5a82f165432a

Wolfe CM, Hamblion EL, Schulte J, et al. Ebola virus disease contact tracing activities, lessons learned and best practices during the Duport Road outbreak in Monrovia, Liberia, November 2015. Scarpino SV, ed. PLoS Negl Trop Dis. 2017;11(6):e0005597. doi:10.1371/journal.pntd.0005597

**Study is not about health workers**

Fallah M, Dahn B, Nyenswah TG, et al. Interrupting Ebola Transmission in Liberia Through Community-Based Initiatives. Ann Intern Med. 2016;164(5):367. doi:10.7326/M15-1464

Nyenswah T, Fahnbulleh M, Massaquoi M, et al. Ebola Epidemic — Liberia, March–October 2014. 2014;63(46):5.

Nyenswah T, Fallah M, Sieh S, et al. Controlling the Last Known Cluster of Ebola Virus Disease — Liberia, January–February 2015. 2015;64(18):5.

Logan G, Vora NM, Nyensuah TG, et al. Establishment of a Community Care Center for Isolation and Management of Ebola Patients — Bomi County, Liberia, October 2014. 2014;63(44):3.

Mesman AW, Bangura M, Kanawa SM, et al. A comprehensive district-level laboratory intervention after the Ebola epidemic in Sierra Leone. *Afr J Lab Med*. 2019;8(1). doi:[10.4102/ajlm.v8i1.885](https://doi.org/10.4102/ajlm.v8i1.885)

McLean KE, Abramowitz SA, Ball JD, et al. Community-based reports of morbidity, mortality, and health-seeking behaviours in four Monrovia communities during the West African Ebola epidemic. *Global Public Health*. 2018;13(5):528-544. doi:[10.1080/17441692.2016.1208262](https://doi.org/10.1080/17441692.2016.1208262)

Full text Unavailable

Nelson AR, Fiedler A, Zikeh T, Moses N, Ruparelia C, Oseni L. Institutionalizing infection prevention and control practices in health facilities in liberia following the Ebola epidemic. *American Journal of Tropical Medicine and Hygiene*. Published online 2018.

Froeschl G. From Ebola emergency to health systems restoration: Temporary infectious disease units in the Ebola campaign in Liberia. *Tropical Medicine and International Health*. Published online 2015.

No Outcome Data

Abramowitz SA, McLean KE, McKune SL, et al. Community-Centered Responses to Ebola in Urban Liberia: The View from Below. Bausch DG, ed. *PLoS Negl Trop Dis*. 2015;9(4):e0003706. doi:[10.1371/journal.pntd.0003706](https://doi.org/10.1371/journal.pntd.0003706)

Abramowitz, Sharon Alane, McLean, Kristen E, McKune, Sarah Lindley, et al. Correction: Community-centered responses to Ebola in urban Liberia: the view from below.

Modelling Study

Washington ML, Meltzer ML. Effectiveness of Ebola Treatment Units and Community Care Centers — Liberia, September 23–October 31, 2014. *MMWR Morb Mortal Wkly Rep*. 2015;64(3).

# Theme 2: Personal Protective Equipment (PPE)

# KQ4/KQ7

Duplicate Reference (Different versions of same systematic review)

Verbeek JH, Ijaz S, Mischke C, et al. Personal protective equipment for preventing highly infectious diseases due to exposure to contaminated body fluids in healthcare staff. Cochrane Work Group, ed. Cochrane Database of Systematic Reviews. Published online April 19, 2016. doi:[10.1002/14651858.CD011621.pub2](https://doi.org/10.1002/14651858.CD011621.pub2)

Verbeek JH, Rajamaki B, Ijaz S, et al. Personal protective equipment for preventing highly infectious diseases due to exposure to contaminated body fluids in healthcare staff. Cochrane Work Group, ed. Cochrane Database of Systematic Reviews. Published online April 15, 2020. doi:[10.1002/14651858.CD011621.pub4](https://doi.org/10.1002/14651858.CD011621.pub4)

Exclude due to non-English language

Bosl E, Dersch W, Fehling SK, et al. Ebola virus disease - handling of personal protective equipment (ppe). [German]. Intensiv- und Notfallbehandlung. Published online 2014.

Schmiedel S, Kreuels B. [Ebola virus disease in West Africa and Germany : clinical presentation, management and practical experience with medevacuated patients in Germany]. Bundesgesundheitsblatt Gesundheitsforschung Gesundheitsschutz. 2015;58(7):679-685. doi:[10.1007/s00103-015-2162-1](https://doi.org/10.1007/s00103-015-2162-1)

Health workers not performing screening or triage activities

Andonian J, Kazi S, Therkorn J, et al. Effect of an Intervention Package and Teamwork Training to Prevent Healthcare Personnel Self-contamination During Personal Protective Equipment Doffing. Clinical Infectious Diseases. 2019;69(Supplement_3):S248-S255. doi:[10.1093/cid/ciz618](https://doi.org/10.1093/cid/ciz618)

Bell T, Smoot J, Patterson J, Smalligan R, Jordan R. Ebola virus disease: The use of fluorescents as markers of contamination for personal protective equipment. IDCases. 2015;2(1):27-30. doi:[10.1016/j.idcr.2014.12.003](https://doi.org/10.1016/j.idcr.2014.12.003)

Buianov VV, Kolesnikov NV, Malyshev NA, Suprun IP. Use of new individual protection substances in Mel'tser boxes. Vestnik Rossiiskoi Akademii Meditsinskikh Nauk / Rossiiskaia Akademiia Meditsinskikh Nauk 2004; 1:30-5.

Casalino E, Astocondor E, Sanchez JC, Díaz-Santana DE, del Aguila C, Carrillo JP. Personal protective equipment for the Ebola virus disease: A comparison of 2 training programs. American Journal of Infection Control. 2015;43(12):1281-1287. doi:[10.1016/j.ajic.2015.07.007](https://doi.org/10.1016/j.ajic.2015.07.007)

Casanova LM, Rutala WA, Weber DJ, Sobsey MD. E.ect of single- versus double-gloving on virus transfer to health care workers' skin and clothing during removal of personal protective equipment. American Journal of Infection Control 2012; 40(4):369-74.

Casanova LM, Teal LJ, Sickbert-Bennett EE, et al. Assessment of Self-Contamination During Removal of Personal Protective Equipment for Ebola Patient Care. Infect Control Hosp Epidemiol. 2016;37(10):1156-1161. doi:[10.1017/ice.2016.169](https://doi.org/10.1017/ice.2016.169)

Chughtai AA, Chen X, Macintyre CR. Risk of self-contamination during doffing of personal protective equipment. American Journal of Infection Control. 2018;46(12):1329-1334. doi:[10.1016/j.ajic.2018.06.003](https://doi.org/10.1016/j.ajic.2018.06.003)

Drews FA, Mulvey D, Stratford K, Samore MH, Mayer J. Evaluation of a Redesigned Personal Protective Equipment Gown. Clinical Infectious Diseases. 2019;69(Supplement_3):S199-S205. doi:[10.1093/cid/ciz520](https://doi.org/10.1093/cid/ciz520)

Dunn AC, Walker TA, Redd J, et al. Nosocomial transmission of Ebola virus disease on pediatric and maternity wards: Bombali and Tonkolili, Sierra Leone, 2014. American Journal of Infection Control. 2016;44(3):269-272. doi:[10.1016/j.ajic.2015.09.016](https://doi.org/10.1016/j.ajic.2015.09.016)

Fogel I, David O, Balik CH, et al. The association between self-perceived proficiency of personal protective equipment and objective performance: An observational study during a bioterrorism simulation drill. American Journal of Infection Control. 2017;45(11):1238-1242. doi:[10.1016/j.ajic.2017.05.018](https://doi.org/10.1016/j.ajic.2017.05.018)

Guo YP, Li Y, Wong PLH. Environment and body contamination: a comparison of two di.erent removal methods in three types of personal protective clothing. American Journal of Infection Control 2014; 42(4):e39-e45.

Gurses AP, Dietz AS, Nowakowski E, et al. Human factors–based risk analysis to improve the safety of doffing enhanced personal protective equipment. Infect Control Hosp Epidemiol. 2019;40(2):178-186. doi:[10.1017/ice.2018.292](https://doi.org/10.1017/ice.2018.292)

Hajar Z, Mana TS, Tomas ME, Alhmidi H, Wilson BM, Donskey CJ. A crossover trial comparing contamination of healthcare personnel during removal of a standard gown versus a modified gown with increased skin coverage at the hands and wrists. Infection Control and Hospital Epidemiology 2019; 40(11):1278-80. [DOI: 10.1017/ice.2019.211]

Huber K, Jones I, Dousa T, et al. An Evidence Based Approach to Testing PPE for Enhanced Isolation Precautions during Ebola Virus Disease Preparedness Planning. American Journal of Infection Control. 2015;43(6):S69. doi:10.1016/j.ajic.2015.04.170

Kogutt BK, Sheffield JS, Garibaldi BT. 680: Assessing effectiveness of PPE in a simulated SVD of a highly infectious disease patient. American Journal of Obstetrics and Gynecology. 2019;220(1):S451. doi:[10.1016/j.ajog.2018.11.703](https://doi.org/10.1016/j.ajog.2018.11.703)

Kwon JH, Burnham CAD, Reske KA, et al. Assessment of Healthcare Worker Protocol Deviations and Self-Contamination During Personal Protective Equipment Donning and Doffing. Infect Control Hosp Epidemiol. 2017;38(9):1077-1083. doi:[10.1017/ice.2017.121](https://doi.org/10.1017/ice.2017.121)

Lim SM, Cha WC, Chae MK, Jo IJ. Contamination during doffing of personal protective equipment by healthcare providers. Clin Exp Emerg Med. 2015;2(3):162-167. doi:[10.15441/ceem.15.019](https://doi.org/10.15441/ceem.15.019)

Mana TS, Tomas ME, Cadnum JL, Jencson AL, Piedrahita CT, Donskey CJ. A randomized trial of two cover gowns comparing contamination of healthcare personnel during removal of personal protective equipment. Infection Control and Hospital Epidemiology 2018; 39(1):97-100.

Mumma JM, Durso FT, Casanova LM, et al. Common Behaviors and Faults When Doffing Personal Protective Equipment for Patients With Serious Communicable Diseases. Clinical Infectious Diseases. 2019;69(Supplement_3):S214-S220. doi:[10.1093/cid/ciz614](https://doi.org/10.1093/cid/ciz614)

Mumma JM, Durso FT, Ferguson AN, et al. Human Factors Risk Analyses of a Doffing Protocol for Ebola-Level Personal Protective Equipment: Mapping Errors to Contamination. Clinical Infectious Diseases. 2018;66(6):950-958. doi:[10.1093/cid/cix957](https://doi.org/10.1093/cid/cix957)

Osei-Bonsu K, Masroor N, Cooper K, Doern C, Je.erson KK,  Major Y, et al. Alternative do.ing strategies of personal protective equipment to prevent self-contamination in the health care setting. American Journal of Infection Control 2019; 47(5):534-9. [PMID: 30578138]

Poller B, Tunbridge A, Hall S, et al. A unified personal protective equipment ensemble for clinical response to possible high consequence infectious diseases: A consensus document on behalf of the HCID programme. Journal of Infection. 2018;77(6):496-502. doi:[10.1016/j.jinf.2018.08.016](https://doi.org/10.1016/j.jinf.2018.08.016)

Poller B, Hall S, Bailey C, et al. ‘VIOLET’: a fluorescence-based simulation exercise for training healthcare workers in the use of personal protective equipment. Journal of Hospital Infection. 2018;99(2):229-235. doi:[10.1016/j.jhin.2018.01.021](https://doi.org/10.1016/j.jhin.2018.01.021)

Shigayeva A, Green K, Raboud JM, Henry B, Simor AE, Vearncombe M, et al. Factors associated with critical-care healthcare workers' adherence to recommended barrier precautions during the Toronto severe acute respiratory syndrome outbreak. Infection Control 2007; 28(11):1275-

Suen LKP, Guo YP, Tong DWK, et al. Self-contamination during doffing of personal protective equipment by healthcare workers to prevent Ebola transmission. Antimicrob Resist Infect Control. 2018;7(1):157. doi:[10.1186/s13756-018-0433-y](https://doi.org/10.1186/s13756-018-0433-y)

Tomas ME, Cadnum JL, Mana TS, Jencson AL, Donskey CJ. Seamless suits: reducing personnel contamination through improved personal protective equipment design. Infection Control and Hospital Epidemiology 2016; 37(6):742-4.

Wong TK, Chung JW, Li Y, Chan WF, Ching PT, Lam CH, et al. E.ective personal protective clothing for health care workers attending patients with severe acute respiratory syndrome. American Journal of Infection Control 2004; 32(2):90-6.

Zamora JE, Murdoch J, Simchison B, Day AG. Contamination: a comparison of 2 personal protective systems. Canadian Medical Association Journal 2006; 175(3):249-54.

Garibaldi BT, Ruparelia C, Shaw-Saliba K, et al. A novel personal protective equipment coverall was rated higher than standard Ebola virus personal protective equipment in terms of comfort, mobility and perception of safety when tested by health care workers in Liberia and in a United States biocontainment unit. *American Journal of Infection Control*. 2019;47(3):298-304. doi:[10.1016/j.ajic.2018.08.014](https://doi.org/10.1016/j.ajic.2018.08.014)

Coca A, DiLeo T, Kim JH, Roberge R, Shaffer R. Baseline Evaluation With a Sweating Thermal Manikin of Personal Protective Ensembles Recommended for Use in West Africa. *Disaster med public health prep*. 2015;9(5):536-542. doi:[10.1017/dmp.2015.97](https://doi.org/10.1017/dmp.2015.97)

Potter AW, Gonzalez JA, Xu X. Ebola Response: Modeling the Risk of Heat Stress from Personal Protective Clothing. Bouchama A, ed. *PLoS ONE*. 2015;10(11):e0143461. doi:[10.1371/journal.pone.0143461](https://doi.org/10.1371/journal.pone.0143461)

Andonian J, Kazi S, Therkorn J, et al. Effect of an Intervention Package and Teamwork Training to Prevent Healthcare Personnel Self-contamination During Personal Protective Equipment Doffing. *Clinical Infectious Diseases*. 2019;69(Supplement_3):S248-S255. doi:[10.1093/cid/ciz618](https://doi.org/10.1093/cid/ciz618)

Jaques PA, Gao P, Kilinc-Balci S, et al. Evaluation of gowns and coveralls used by medical personnel working with Ebola patients against simulated bodily fluids using an Elbow Lean Test. *Journal of Occupational and Environmental Hygiene*. 2016;13(11):881-893. doi:[10.1080/15459624.2016.1186279](https://doi.org/10.1080/15459624.2016.1186279)

Den Boon S, Vallenas C, Ferri M, Norris SL. Incorporating health workers’ perspectives into a WHO guideline on personal protective equipment developed during an Ebola virus disease outbreak. *F1000Res*. 2018;7:45. doi:[10.12688/f1000research.12922.1](https://doi.org/10.12688/f1000research.12922.1)

Coca A, Quinn T, Kim JH, et al. Physiological Evaluation of Personal Protective Ensembles Recommended for Use in West Africa. *Disaster med public health prep*. 2017;11(5):580-586. doi:[10.1017/dmp.2017.13](https://doi.org/10.1017/dmp.2017.13)

Hall S, Poller B, Bailey C, et al. Use of ultraviolet-fluorescence-based simulation in evaluation of personal protective equipment worn for first assessment and care of a patient with suspected high-consequence infectious disease. *Journal of Hospital Infection*. 2018;99(2):218-228. doi:[10.1016/j.jhin.2018.01.002](https://doi.org/10.1016/j.jhin.2018.01.002)

Infection rates not presented by HW activity (screening/triage) and use of PPE equipment

Doshi RH, Hoff NA, Bratcher A, et al. Risk Factors for Ebola Exposure in Health Care Workers in Boende, Tshuapa Province, Democratic Republic of the Congo. The Journal of Infectious Diseases. Published online December 3, 2020:jiaa747. doi:[10.1093/infdis/jiaa747](https://doi.org/10.1093/infdis/jiaa747)

Irrelevant PPE comparison

Bustamante ND, O’Keeffe D, Bradley D, Pozner CN. Targeted interprofessional simulation-based training for safe patient management of Ebola virus disease. Academic Emergency Medicine. Published online 2015.

Doshi RH, Hoff NA, Mukadi P, et al. Seroprevalence of ebola virus among health care workers in the Tshuapa district democratic republic of congo. Am J Tropic Med Hygiene. Published online 2016.

Drew JL, Turner J, Mugele J, et al. Beating the Spread: Developing a Simulation Analog for Contagious Body Fluids. Simulation in Healthcare: The Journal of the Society for Simulation in Healthcare. 2016;11(2):100-105. doi:[10.1097/SIH.0000000000000157](https://doi.org/10.1097/SIH.0000000000000157)

Garde DL, Hall AMR, Marsh RH, Barron KP, Dierberg KL, Koroma AP. Implementation of the first dedicated Ebola screening and isolation for maternity patients in Sierra Leone. Annals of Global Health. 2016;82(3):418. doi:[10.1016/j.aogh.2016.04.164](https://doi.org/10.1016/j.aogh.2016.04.164)

Garibaldi BT, Ruparelia C, Shaw-Saliba K, et al. A novel personal protective equipment coverall was rated higher than standard Ebola virus personal protective equipment in terms of comfort, mobility and perception of safety when tested by health care workers in Liberia and in a United States biocontainment unit. American Journal of Infection Control. 2019;47(3):298-304. doi:[10.1016/j.ajic.2018.08.014](https://doi.org/10.1016/j.ajic.2018.08.014)

Fischer WA, Weber DJ, Wohl DA. Personal Protective Equipment: Protecting Health Care Providers in an Ebola Outbreak. Clinical Therapeutics. 2015;37(11):2402-2410. doi:[10.1016/j.clinthera.2015.07.007](https://doi.org/10.1016/j.clinthera.2015.07.007)

Hoff NA, Mwanza A, Doshi RH, et al. Possible high exposure to ebola among non-formal health care providers in a previous outbreak site boende democratic republic of congo. The Journal of Infectious Diseases. 2016;219:517-525.

Hopman J, Kubilay Z, Allen T, Edrees H, Pittet D, Allegranzi B. Efficacy of chlorine solutions used for hand hygiene and gloves disinfection in Ebola settings: a systematic review. Antimicrob Resist Infect Control. 2015;4(S1):O13. doi:[10.1186/2047-2994-4-S1-O13](https://doi.org/10.1186/2047-2994-4-S1-O13)

Houlihan CF, McGowan CR, Dicks S, Baguelin M, Moore DA, Mabey D, et al. Ebola exposure, illness experience and Ebola antibody prevelence in international responders to the West African Ebola epidemic 2014-2016: a cross sectional study. PLoS Medicine 2017; 14(4):e1002300.

Ijarotimi IT, Ilesanmi OS, Aderinwale A, Abiodun-Adewusi O. Knowledge of Lassa fever and use of infection prevention and control facilities among health care workers during Lassa fever outbreak in Ondo State, Nigeria. :13.

Kilinc FS. A Review of Isolation Gowns in Healthcare: Fabric and Gown Properties. Journal of Engineered Fibers and Fabrics. 2015;10(3):155892501501000. doi:[10.1177/155892501501000313](https://doi.org/10.1177/155892501501000313)

Kwon JH, Burnham CAD, Reske K, et al. Healthcare Worker Self-Contamination During Standard and Ebola Virus Disease Personal Protective Equipment Doffing. Open Forum Infectious Diseases. 2016;3(suppl_1):1387. doi:[10.1093/ofid/ofw172.1090](https://doi.org/10.1093/ofid/ofw172.1090)

Laff MH, Michelfelder A, Vlasses F, Parise NA, Quinones D. Hand-in-Hand: Interprofessional Education for Infection Prevention. American Journal of Infection Control. 2016;44(6):S62. doi:[10.1016/j.ajic.2016.04.064](https://doi.org/10.1016/j.ajic.2016.04.064)

Lee M a, Huh K, Jeong J, et al. Adherence to Protocols by Healthcare Workers and Self-Contamination During Doffing of Personal Protective Equipment. American Journal of Infection Control. 2018;46(6):S11. doi:[10.1016/j.ajic.2018.04.024](https://doi.org/10.1016/j.ajic.2018.04.024)

Licina A, Silvers A, Stuart RL. Use of powered air-purifying respirator (PAPR) by healthcare workers for preventing highly infectious viral diseases—a systematic review of evidence. Syst Rev. 2020;9(1):173. doi:[10.1186/s13643-020-01431-5](https://doi.org/10.1186/s13643-020-01431-5)

Mallow M, Gary L, Jeng T, et al. WASH activities at two Ebola treatment units in Sierra Leone. Ikegami T, ed. PLoS ONE. 2018;13(5):e0198235. doi:[10.1371/journal.pone.0198235](https://doi.org/10.1371/journal.pone.0198235)

Mohammed HM. Ebola virus disease: Effects of respiratory protection on healthcare workers. Egyptian Journal of Chest Diseases and Tuberculosis. 2015;64(3):639-644. doi:[10.1016/j.ejcdt.2015.04.015](https://doi.org/10.1016/j.ejcdt.2015.04.015)

Nelson AR, Fiedler A, Zikeh T, Moses N, Ruparelia C, Oseni L. Institutionalizing infection prevention and control practices in health facilities in liberia following the Ebola epidemic. American Journal of Tropical Medicine and Hygiene. Published online 2018.

Olugbade OT, Israel O, Fadahunsi R, Ogunniyi A, Oladimeji A, Olayinka A. Outbreak of lassa fever in a healthcare setting : Epidemiologic surveillance and risk stratification for contact persons-Saki Oyo State Nigeria 2014. Antimicrobial Resistance and Infection Control Conference: International Conference on Prevention and Infection Control ICPIC. 2017.

Raj D. What Are the Appropriate Personal Protective Equipment (PPE) for Front-line Workers (FLW) Caring for Filovirus/Ebola Virus Disease (EVD) Patients? Open Forum Infectious Diseases. 2017;4(suppl_1):S170-S170. doi:[10.1093/ofid/ofx163.302](https://doi.org/10.1093/ofid/ofx163.302)

Reidy P, Fletcher T, Shieber C, et al. Personal protective equipment solution for UK military medical personnel working in an Ebola virus disease treatment unit in Sierra Leone. Journal of Hospital Infection. 2017;96(1):42-48. doi:[10.1016/j.jhin.2017.03.018](https://doi.org/10.1016/j.jhin.2017.03.018)

Strauch AL, Brady TM, Niezgoda G, Almaguer CM, Sha.er RE, Fisher EM. Assessing the e.icacy of tabs on filtering facepiece respirator straps to increase proper do.ing techniques while reducing contact transmission of pathogens. Journal of Occupational and Environmental Hygiene 2016; 13(10):794-801.

Wong MF, Matić Z, Campiglia GC, et al. Design Strategies for Biocontainment Units to Reduce Risk During Doffing of High-level Personal Protective Equipment. *Clinical Infectious Diseases*. 2019;69(Supplement_3):S241-S247. doi:[10.1093/cid/ciz617](https://doi.org/10.1093/cid/ciz617)

DuBose JR, Matić Z, Sala MFW, et al. Design strategies to improve healthcare worker safety in biocontainment units: learning from ebola preparedness. *Infect Control Hosp Epidemiol*. 2018;39(8):961-967. doi:[10.1017/ice.2018.125](https://doi.org/10.1017/ice.2018.125)

Kilinc-Balci FS, Nwoko J, Hillam T. Evaluation of the Performance of Isolation Gowns. *American Journal of Infection Control*. 2015;43(6):S44. doi:[10.1016/j.ajic.2015.04.112](https://doi.org/10.1016/j.ajic.2015.04.112)

Baloh J, Reisinger HS, Dukes K, et al. Healthcare Workers’ Strategies for Doffing Personal Protective Equipment. *Clinical Infectious Diseases*. 2019;69(Supplement_3):S192-S198. doi:[10.1093/cid/ciz613](https://doi.org/10.1093/cid/ciz613)

Rubin J, Hill ME. Implementing a High-Consequence Infectious Disease Preparedness Program at an Urban Community Hospital. *American Journal of Infection Control*. 2020;48(8):S44-S45. doi:[10.1016/j.ajic.2020.06.050](https://doi.org/10.1016/j.ajic.2020.06.050)

Licina A, Silvers A, Stuart RL. Use of powered air-purifying respirator (PAPR) by healthcare workers for preventing highly infectious viral diseases—a systematic review of evidence. *Syst Rev*. 2020;9(1):173. doi:[10.1186/s13643-020-01431-5](https://doi.org/10.1186/s13643-020-01431-5)

Non-Comparative Study

Borchert M, Mutyaba I, Van Kerkhove MD, Lutwama J, Luwaga H, Bisoborwa G, et al. Ebola haemorrhagic fever outbreak in Masindi District, Uganda: outbreak description and lessons learned. BMC Infect Dis 2011; 11:357. [PMID: 22204600] doi: 10.1186/1471-2334-11-357

Ebola haemorrhagic fever in Sudan, 1976. Report of a WHO/International Study Team. Bull World Health Organ 1978; 56(2):247–70. [PMID: 307455]

Ebola haemorrhagic fever in Zaire, 1976. Bull World Health Organ 1978; 56(2):271–93. [PMID: 307456]

Formenty P, Hatz C, Le GB, Stoll A, Rogenmoser P, Widmer A. Human infection due to Ebola virus, subtype Cote d'Ivoire: clinical and biologic presentation. J Infect Dis 1999 Feb; 179 Suppl 1:S48–S53. [PMID: 9988164]

Gear JS, Cassel GA, Gear AJ, Trappler B, Clausen L, Meyers AM, et al. Outbreake of Marburg virus disease in Johannesburg. Br Med J 1975 Nov 29; 4(5995):489–93. [PMID: 811315]

Hall S, Poller B, Bailey C, et al. Use of ultraviolet-fluorescence-based simulation in evaluation of personal protective equipment worn for first assessment and care of a patient with suspected high-consequence infectious disease. Journal of Hospital Infection. 2018;99(2):218-228. doi:[10.1016/j.jhin.2018.01.002](https://doi.org/10.1016/j.jhin.2018.01.002)

Kalongi Y, Mwanza K, Tshisuaka M, Lusiama N, Ntando E, Kanzake L, et al. Isolated case of Ebola hemorrhagic fever with mucormycosis complications, Kinshasa, Democratic Republic of the Congo. J Infect Dis 1999 Feb; 179 Suppl 1:S15–S17. [PMID: 9988159]

Khan AS, Tshioko FK, Heymann DL, Le GB, Nabeth P, Kerstiens B, et al. The reemergence of Ebola hemorrhagic fever, Democratic Republic of the Congo, 1995. Commission de Lutte contre les Epidemies a Kikwit. J Infect Dis 1999 Feb; 179 Suppl 1:S76–S86. [PMID: 9988168]

Martini GA. Marburg agent disease: in man. Trans R Soc Trop Med Hyg 1969; 63(3):295–302. [PMID: 5815873]

Richards GA, Murphy S, Jobson R, Mer M, Zinman C, Taylor R, et al. Unexpected Ebola virus in a tertiary setting: clinical and epidemiologic aspects. Crit Care Med 2000 Jan; 28(1):240–4. [PMID: 10667531]

Shoemaker T, MacNeil A, Balinandi S, Campbell S, Wamala JF, McMullan LK, et al. Reemerging Sudan Ebola virus disease in Uganda, 2011. Emerg Infect Dis 2012 Sep; 18(9):1480–3. [PMID:22931687] doi: 10.3201/eid1809.111536

Smith DH, Johnson BK, Isaacson M, Swanapoel R, Johnson KM, Killey M, et al. Marburg-virus disease in Kenya. Lancet 1982 Apr 10; 1(8276):816–20. [PMID: 6122054]

Grélot L, Koulibaly F, Maugey N, et al. Moderate Thermal Strain in Healthcare Workers Wearing Personal Protective Equipment During Treatment and Care Activities in the Context of the 2014 Ebola Virus Disease Outbreak. *J Infect Dis*. 2016;213(9):1462-1465. doi:[10.1093/infdis/jiv585](https://doi.org/10.1093/infdis/jiv585)

Eiras D, Echeverri A, Toale K, Tennill P, Evans L. Painting the Gown Red: Using a Colored Paint Quality Improvement Process to Evaluate Healthcare Worker Personal Protective Equipment for Highly Pathogenic Infections. Published online 2017. <https://www.ncbi.nlm.nih.gov/pmc/articles/PMC5630798/>

Zellmer C, Van Hoof S, Safdar N. Variation in health care worker removal of personal protective equipment. *American Journal of Infection Control*. 2015;43(7):750-751. doi:[10.1016/j.ajic.2015.02.005](https://doi.org/10.1016/j.ajic.2015.02.005)

No outcome data related to use of PPE

Chu M, Bausch D, Velazquez-Berumen A, Vallenas C, A. Committees. Report from the world health organization’s advisory committees on innovative personal protective equipment for front line health workers. 2017;American Journal of Tropical Medicine and Hygiene.

Den Boon S, Vallenas C, Ferri M, Norris SL. Incorporating health workers’ perspectives into a WHO guideline on personal protective equipment developed during an Ebola virus disease outbreak. F1000Res. 2018;7:45. doi:[10.12688/f1000research.12922.2](https://doi.org/10.12688/f1000research.12922.2)

Franklin SM. A Comparison of Personal Protective Standards: Caring for Patients With Ebola Virus. Clinical Nurse Specialist. 2016;30(2):E1-E8. doi:[10.1097/NUR.0000000000000183](https://doi.org/10.1097/NUR.0000000000000183)

Grélot L, Koulibaly F, Maugey N, et al. Moderate Thermal Strain in Healthcare Workers Wearing Personal Protective Equipment During Treatment and Care Activities in the Context of the 2014 Ebola Virus Disease Outbreak. J Infect Dis. 2016;213(9):1462-1465. doi:[10.1093/infdis/jiv585](https://doi.org/10.1093/infdis/jiv585)

Jalloh MB, Chu MC. A doctor’s experience: The dilemma faced using personal protective equipment while working in an ebola treatment unit. American Journal of Tropical Medicine and Hygiene. Published online 2019.

Holt A, Hornsey E, Seale AC, et al. A mixed-methods analysis of personal protective equipment used in Lassa fever treatment centres in Nigeria. Infection Prevention in Practice. 2021;3(3):100168. doi:[10.1016/j.infpip.2021.100168](https://doi.org/10.1016/j.infpip.2021.100168)

Raj D, Hornsey E, Perl TM. Personal protective equipment for viral hemorrhagic fevers: Current Opinion in Infectious Diseases. 2019;32(4):337-347. doi:[10.1097/QCO.0000000000000562](https://doi.org/10.1097/QCO.0000000000000562)

With strengthened guidelines for health care workers, the CDC ups its game against the deadly Ebola virus. ED management : the monthly update on emergency department management. 2014;26(12):133-136.

**Full text Unavailable**

Ahoyo T, Gazard DK, Gounongbe M. Evaluation of basic infection prevention practices in health care set-ups in Benin. Antimicrobial Resistance and Infection Control Conference: International Conference on Prevention and Infection Control ICPIC. Published online 2017.

Laubscher A, Toubkin M. Managing the risk of suspected and confirmed cases of Viral Haemorrhagic Fever (VHF) specifically Ebola Virus Disease (EVD) in a private hospital group. Southern African Journal of Epidemiology and Infection. Published online 2015.

Lee MH, Meerbach A, Straub J, et al. Which personal protective equipment to provide?-Challenges during the Ebola outbreak and lessons learned. Tropical Medicine and International Health. Published online 2017.

Senga M, Pringle K, Brett-Major D, et al. Factors associated with mortality of health workers with Ebola virus disease in Kenema district Sierra Leone. Tropical Medicine and International Health. Published online 2015.

Senga M, Pringle K, Brett-Major D, et al. Largest documented cluster of ebola virus disease among health workers. Published online 2015.

Somers Y, Verbiest M. Suspecting ebola: When the dress code becomes life saving! Personal protective equipment-a practical demonstration. Anaesthesiology Intensive Therapy. Published online 2014.

Garibaldi BT, Rainwater-Lovett K, Pilholski T, et al. Transmission of fluorescent aerosolized particles in a clinical biocontainment unit. *American Journal of Respiratory and Critical Care Medicine Conference: American Thoracic Society International Conference ATS*. Published online 2017.

Raj D. What Are the Appropriate Personal Protective Equipment (PPE) for Front line Workers (FLW) Caring for Filovirus/Ebola Virus Disease (EVD) Patients?

Protocol

Verbeek JH, Ijaz, S, Mischke, C, Ruotsalainen, JH, Mäkelä, E, Neuvonen, K, Edmond, MB, Garner, P, Sauni, R, Hopping K. Personal protective equipment for preventing highly infectious diseases due to contact with contaminated body fluids in health care staff. Cochrane Database of Systematic Reviews. 2015;(4). doi:[10.1002/14651858.CD011621](https://doi.org/10.1002/14651858.CD011621)

Study does not evaluate health workers

McCulloch KL, Michael F, Goren M, et al. Creating an Environment of Safety for the Treatment of Patients with Ebola. American Journal of Infection Control. 2015;43(6):S73. doi:[10.1016/j.ajic.2015.04.193](https://doi.org/10.1016/j.ajic.2015.04.193)

Moody J, Cormier S, Hickok J, Septimus E, Chari R, Perlin J. The Creation and Rapid Deployment of a Preparedness Plan for Ebola Virus Disease: Lessons from a Large Healthcare System. American Journal of Infection Control. 2015;43(6):S15-S16. doi:[10.1016/j.ajic.2015.04.038](https://doi.org/10.1016/j.ajic.2015.04.038)

Tseng CP, Chan YJ. Overview of Ebola virus disease in 2014. Journal of the Chinese Medical Association. 2015;78(1):51-55. doi:[10.1016/j.jcma.2014.11.007](https://doi.org/10.1016/j.jcma.2014.11.007)

Wang YF. Ebola bio-safety and laboratory testing. Journal of Microbiology, Immunology and Infection. 2015;48(2):S17. doi:[10.1016/j.jmii.2015.02.161](https://doi.org/10.1016/j.jmii.2015.02.161)

Study does not evaluate PPE for health care workers

Abela N, Bonnici ET, Parascandalo A, Borg M. Lessons learnt and challenges in adopting the ECDC and who Ebola guidelines at Mater Dei Hospital. Antimicrob Resist Infect Control. 2015;4(S1):P5, 2047-2994-4-S1-P5. doi:[10.1186/2047-2994-4-S1-P5](https://doi.org/10.1186/2047-2994-4-S1-P5)

Berry L, Button T, Fonnie C, King M. How to set up an Ebola isolation unit: Lessons learned from Rokupa. Journal of Clinical Virology. 2015;70:S17. doi:[10.1016/j.jcv.2015.07.046](https://doi.org/10.1016/j.jcv.2015.07.046)

Curtis HA, Trang K, Chason KW, Biddinger PD. Video-based learning vs traditional lecture for instructing emergency medicine residents in disaster medicine principles of mass triage, decontamination, and personal protective equipment. Prehospital and Disaster Medicine 2018; 33(1):7-12.

De Clerck H. Protecting the health care worker during outbreaks – The case of viral hemorrhagic fever outbreaks. International Journal of Infectious Diseases. 2016;45:67. doi:[10.1016/j.ijid.2016.02.194](https://doi.org/10.1016/j.ijid.2016.02.194)

Drews FA, Mulvey D, Stratford K, Samore MH, Mayer J. Evaluation of a redesigned personal protective equipment gown. Clinical Infectious Diseases 2019; 69 Suppl 3:S199-205. [PMID: 31517973]

Gleser M, Schwab F, Solbach P, Vonberg RP. Modified gloves: a chance for the prevention of nosocomial infections. American Journal of Infection Control 2018; 46(3):266-9.

Hall S, Poller B, Bailey C, Gregory S, Clark R, Roberts P, et al. Use of ultraviolet-fluorescence-based simulation in evaluation of personal protective equipment worn for first assessment and care of a patient with suspected high-consequence infectious disease. Journal of Hospital Infection 2018; 99(2):218-28.

Hung PP, Choi KS, Chiang VC. Using interactive computer simulation for teaching the proper use of personal protective equipment. CIN: Computers, Informatics, Nursing 2015;33(2):49-57.

Kpadeh-Rogers Z, Robinson GL, Alserehi H, Morgan DJ, Harris AD, Herrera NB, et al. E.ect of glove decontamination on bacterial contamination of healthcare personnel hands. Clinical Infectious Diseases 2019; 69:S224-S227. [DOI: 10.1093/cid/ciz615]

Musene KK, Hoff NA, Spencer D, et al. Occupational exposure of health care workers in Kinshasa, Democratic Republic of the Congo. American Journal of Tropical Medicine and Hygiene. Published online 2018.

Obionu IM, Ochu CL, Ukponu W, et al. Evaluation of infection prevention and control practices in Lassa fever treatment centers in north-central Nigeria during an ongoing Lassa fever outbreak. Journal of Infection Prevention. 2021;22(6):275-282.

Ortega R, Bhadelia N, Obanor O, et al. Putting On and Removing Personal Protective Equipment. N Engl J Med. 2015;372(12):e16. doi:[10.1056/NEJMvcm1412105](https://doi.org/10.1056/NEJMvcm1412105)

Public health experts urge U.S. hospitals to be prepared as Ebola outbreak accelerates. ED management : the monthly update on emergency department management. [Public health experts urge U.S. hospitals to be prepared as Ebola outbreak accelerates](https://doi.org/Public%20health%20experts%20urge%20U.S.%20hospitals%20to%20be%20prepared%20as%20Ebola%20outbreak%20accelerates). Published 2014

Reddy SC, Valderrama AL, Kuhar DT. Improving the Use of Personal Protective Equipment: Applying Lessons Learned. Clinical Infectious Diseases. 2019;69(Supplement_3):S165-S170. doi:[10.1093/cid/ciz619](https://doi.org/10.1093/cid/ciz619)

Roberts R. To PAPR or not to PAPR? Can J respir ther. 2014;50(3):87-90.

Senga M, Pringle K, Ramsay A, et al. Factors Underlying Ebola Virus Infection Among Health Workers, Kenema, Sierra Leone, 2014–2015. Clin Infect Dis. 2016;63(4):454-459. doi:[10.1093/cid/ciw327](https://doi.org/10.1093/cid/ciw327)

Sprecher AG, Caluwaerts A, Draper M, et al. Personal Protective Equipment for Filovirus Epidemics: A Call for Better Evidence. J Infect Dis. 2015;212(suppl 2):S98-S100. doi:[10.1093/infdis/jiv153](https://doi.org/10.1093/infdis/jiv153)

Yarbrough ML, Kwon JH, Wallace MA, et al. Frequency of Instrument, Environment, and Laboratory Technologist Contamination during Routine Diagnostic Testing of Infectious Specimens. Caliendo AM, ed. J Clin Microbiol. 2018;56(6):e00225-18. doi:[10.1128/JCM.00225-18](https://doi.org/10.1128/JCM.00225-18)

Study is not about Ebola or Marburg Virus

Bangura I, Conteh C. The Impact of Quality Improvement Methodology to Improve Infection Control Practices. Antimicrobial Resistance & Infection Control. 2019;8(1):P405.

Fletcher TE, Gulzhan A, Ahmeti S, et al. Infection prevention and control practice for Crimean-Congo hemorrhagic fever—A multi-center cross-sectional survey in Eurasia. Ciccozzi M, ed. PLoS ONE. 2017;12(9):e0182315. doi:[10.1371/journal.pone.0182315](https://doi.org/10.1371/journal.pone.0182315)

Gao P, Horvatin M, Niezgoda G, Weible R, Shaffer R. Effect of multiple alcohol-based hand rub applications on the tensile properties of thirteen brands of medical exam nitrile and latex gloves. Journal of Occupational and Environmental Hygiene. 2016;13(12):905-914. doi:[10.1080/15459624.2016.1191640](https://doi.org/10.1080/15459624.2016.1191640)

Gozel MG, Dokmetas I, Oztop AY, Engin A, Elaldi N, Bakir M. Recommended precaution procedures protect healthcare workers from Crimean-Congo hemorrhagic fever virus. International Journal of Infectious Diseases. 2013;17(11):e1046-e1050. doi:[10.1016/j.ijid.2013.05.005](https://doi.org/10.1016/j.ijid.2013.05.005)

Pshenichnaya NY, Nenadskaya SA. Probable Crimean-Congo hemorrhagic fever virus transmission occurred after aerosol-generating medical procedures in Russia: nosocomial cluster. International Journal of Infectious Diseases. 2015;33:120-122. doi:[10.1016/j.ijid.2014.12.047](https://doi.org/10.1016/j.ijid.2014.12.047)

Unclear reporting of PPE worn during infection

Cummings KJ, Choi MJ, Esswein EJ, et al. Addressing Infection Prevention and Control in the First U.S. Community Hospital to Care for Patients With Ebola Virus Disease: Context for National Recommendations and Future Strategies. Ann Intern Med. 2016;165(1):41. doi:[10.7326/M15-2944](https://doi.org/10.7326/M15-2944)

Dixon J, Houlihan C, Schmid ML. European preparedness for Ebola management. A tropnet (European Network for Tropical Medicine & Travel Health) survey. Tropical Medicine and International Health. Published online 2015.

Matanock A, Arwady MA, Ayscue P, et al. Ebola Virus Disease Cases Among Health Care Workers Not Working in Ebola Treatment Units — Liberia, June–August, 2014. 2014;63(46):5.

Mba S, Ukponu W, Saleh M, et al. Lassa fever infection among health care workers in Nigeria, 2019. International Journal of Infectious Diseases. 2020;101:279. doi:[10.1016/j.ijid.2020.09.731](https://doi.org/10.1016/j.ijid.2020.09.731)

Narrative Review

Kilinc Balci FS. Isolation gowns in health care settings: Laboratory studies, regulations and standards, and potential barriers of gown selection and use. *American Journal of Infection Control*. 2016;44(1):104-111. doi:[10.1016/j.ajic.2015.07.042](https://doi.org/10.1016/j.ajic.2015.07.042)

Honda H, Iwata K. Personal protective equipment and improving compliance among healthcare workers in high-risk settings: *Current Opinion in Infectious Diseases*. 2016;29(4):400-406. doi:[10.1097/QCO.0000000000000280](https://doi.org/10.1097/QCO.0000000000000280)

Brown L, Munro J, Rogers S. Use of personal protective equipment in nursing practice. *Nursing Standard*. 2019;34(5):59-66. doi:[10.7748/ns.2019.e11260](https://doi.org/10.7748/ns.2019.e11260)

KQ5

**Abstract Only**

Abela N, Bonnici ET, Parascandalo A, Borg M. Lessons learnt and challenges in adopting the ECDC and who Ebola guidelines at Mater Dei Hospital. *Antimicrob Resist Infect Control*. 2015;4(S1):P5, 2047-2994-4-S1-P5. doi:[10.1186/2047-2994-4-S1-P5](https://doi.org/10.1186/2047-2994-4-S1-P5)

**Commentary**

MacIntyre CR, Chughtai AA, Seale H, Richards GA, Davidson PM. Response to Martin-Moreno et al. (2014) Surgical mask or no mask for health workers not a defensible position for Ebola. *International Journal of Nursing Studies*. 2014;51(12):1694-1695. doi:[10.1016/j.ijnurstu.2014.10.004](https://doi.org/10.1016/j.ijnurstu.2014.10.004)

MacIntyre CR, Chughtai AA, Seale H, Richards GA, Davidson PM. Uncertainty, risk analysis and change for Ebola personal protective equipment guidelines. *International Journal of Nursing Studies*. 2015;52(5):899-903. doi:[10.1016/j.ijnurstu.2014.12.001](https://doi.org/10.1016/j.ijnurstu.2014.12.001)

Fischer WA, Weber DJ, Wohl DA. Personal Protective Equipment: Protecting Health Care Providers in an Ebola Outbreak. *Clinical Therapeutics*. 2015;37(11):2402-2410. doi:[10.1016/j.clinthera.2015.07.007](https://doi.org/10.1016/j.clinthera.2015.07.007)

**Narrative review**

Franklin SM. A Comparison of Personal Protective Standards: Caring for Patients With Ebola Virus. *Clinical Nurse Specialist*. 2016;30(2):E1-E8. doi:[10.1097/NUR.0000000000000183](https://doi.org/10.1097/NUR.0000000000000183)

Honda H, Iwata K. Personal protective equipment and improving compliance among healthcare workers in high-risk settings: *Current Opinion in Infectious Diseases*. 2016;29(4):400-406. doi:[10.1097/QCO.0000000000000280](https://doi.org/10.1097/QCO.0000000000000280)

Brown L, Munro J, Rogers S. Use of personal protective equipment in nursing practice. *Nursing Standard*. 2019;34(5):59-66. doi:[10.7748/ns.2019.e11260](https://doi.org/10.7748/ns.2019.e11260)

**No information on PPE**

De Clerck H. Protecting the health care worker during outbreaks – The case of viral hemorrhagic fever outbreaks. *International Journal of Infectious Diseases*. 2016;45:67. doi:[10.1016/j.ijid.2016.02.194](https://doi.org/10.1016/j.ijid.2016.02.194)

Fischer WA, Hynes NA, Perl TM. Protecting Health Care Workers From Ebola: Personal Protective Equipment Is Critical but Is Not Enough. *Ann Intern Med*. 2014;161(10):753. doi:[10.7326/M14-1953](https://doi.org/10.7326/M14-1953)

Martin-Moreno JM, Llinás G, Hernández JM. Is respiratory protection appropriate in the Ebola response? *The Lancet*. 2014;384(9946):856. doi:[10.1016/S0140-6736(14)61343-X](https://doi.org/10.1016/S0140-6736(14)61343-X)

Martin-Moreno JM, Llinás G, Martínez-Hernández J. Response to “MacIntyre et al., 2014: Respiratory protection for healthcare workers treating Ebola virus disease (EVD): are facemasks sufficient to meet occupational health and safety obligations?” *International Journal of Nursing Studies*. 2014;51(12):1693. doi:[10.1016/j.ijnurstu.2014.10.005](https://doi.org/10.1016/j.ijnurstu.2014.10.005)

Savini H, Janvier F, Karkowski L, et al. Occupational Exposures to Ebola Virus in Ebola Treatment Center, Conakry, Guinea. *Emerg Infect Dis*. 2017;23(8):1380-1383. doi:[10.3201/eid2308.161804](https://doi.org/10.3201/eid2308.161804)

 McCulloch KL, Michael F, Goren M, et al. Creating an Environment of Safety for the Treatment of Patients with Ebola. *American Journal of Infection Control*. 2015;43(6):S73. doi:[10.1016/j.ajic.2015.04.193](https://doi.org/10.1016/j.ajic.2015.04.193)

Olu O, Kargbo B, Kamara S, et al. Epidemiology of Ebola virus disease transmission among health care workers in Sierra Leone, May to December 2014: a retrospective descriptive study. *BMC Infect Dis*. 2015;15(1):416. doi:[10.1186/s12879-015-1166-7](https://doi.org/10.1186/s12879-015-1166-7)

Tseng CP, Chan YJ. Overview of Ebola virus disease in 2014. *Journal of the Chinese Medical Association*. 2015;78(1):51-55. doi:[10.1016/j.jcma.2014.11.007](https://doi.org/10.1016/j.jcma.2014.11.007)

**No relevant comparisons**

Den Boon S, Vallenas C, Ferri M, Norris SL. Incorporating health workers’ perspectives into a WHO guideline on personal protective equipment developed during an Ebola virus disease outbreak. *F1000Res*. 2018;7:45. doi:[10.12688/f1000research.12922.2](https://doi.org/10.12688/f1000research.12922.2)

Doshi RH, Hoff NA, Bratcher A, et al. Risk Factors for Ebola Exposure in Health Care Workers in Boende, Tshuapa Province, Democratic Republic of the Congo. *The Journal of Infectious Diseases*. Published online 2020:jiaa747. doi:[10.1093/infdis/jiaa747](https://doi.org/10.1093/infdis/jiaa747)

Doshi RH, Hoff NA, Mukadi P, et al. Seroprevalence of ebola virus among health care workers in the Tshuapa district democratic republic of congo. *Am J Tropic Med Hygiene*. Published online 2016.

Dunn AC, Walker TA, Redd J, et al. Nosocomial transmission of Ebola virus disease on pediatric and maternity wards: Bombali and Tonkolili, Sierra Leone, 2014. *American Journal of Infection Control*. 2016;44(3):269-272. doi:[10.1016/j.ajic.2015.09.016](https://doi.org/10.1016/j.ajic.2015.09.016)

Chughtai AA, Chen X, Macintyre CR. Risk of self-contamination during doffing of personal protective equipment. *American Journal of Infection Control*. 2018;46(12):1329-1334. doi:[10.1016/j.ajic.2018.06.003](https://doi.org/10.1016/j.ajic.2018.06.003)

Grélot L, Koulibaly F, Maugey N, et al. Moderate Thermal Strain in Healthcare Workers Wearing Personal Protective Equipment During Treatment and Care Activities in the Context of the 2014 Ebola Virus Disease Outbreak. *J Infect Dis*. 2016;213(9):1462-1465. doi:[10.1093/infdis/jiv585](https://doi.org/10.1093/infdis/jiv585)

Hanoa RO, Moen BE. Ebola Care and Lack of Consensus on Personal Protective Respiratory Equipment. *Workplace Health Saf*. 2016;64(2):48-50. doi:[10.1177/2165079915608405](https://doi.org/10.1177/2165079915608405)

Hersi M, Stevens A, Quach P, et al. Effectiveness of Personal Protective Equipment for Healthcare Workers Caring for Patients with Filovirus Disease: A Rapid Review. Kuhn JH, ed. *PLoS ONE*. 2015;10(10):e0140290. doi:[10.1371/journal.pone.0140290](https://doi.org/10.1371/journal.pone.0140290)

Hoff NA, Mwanza A, Doshi RH, et al. Possible high exposure to ebola among non-formal health care providers in a previous outbreak site boende democratic republic of congo. *The Journal of Infectious Diseases*. 2016;219:517-525.

Holt A, Hornsey E, Seale AC, et al. A mixed-methods analysis of personal protective equipment used in Lassa fever treatment centres in Nigeria. *Infection Prevention in Practice*. 2021;3(3):100168. doi:[10.1016/j.infpip.2021.100168](https://doi.org/10.1016/j.infpip.2021.100168)

Licina A, Silvers A, Stuart RL. Use of powered air-purifying respirator (PAPR) by healthcare workers for preventing highly infectious viral diseases—a systematic review of evidence. *Syst Rev*. 2020;9(1):173. doi:[10.1186/s13643-020-01431-5](https://doi.org/10.1186/s13643-020-01431-5)

MacIntyre CR, Chughtai AA, Seale H, Richards GA, Davidson PM. Respiratory protection for healthcare workers treating Ebola virus disease (EVD): Are facemasks sufficient to meet occupational health and safety obligations? *International Journal of Nursing Studies*. 2014;51(11):1421-1426. doi:[10.1016/j.ijnurstu.2014.09.002](https://doi.org/10.1016/j.ijnurstu.2014.09.002)

Mohammed HM. Ebola virus disease: Effects of respiratory protection on healthcare workers. *Egyptian Journal of Chest Diseases and Tuberculosis*. 2015;64(3):639-644. doi:[10.1016/j.ejcdt.2015.04.015](https://doi.org/10.1016/j.ejcdt.2015.04.015)

Musene KK, Hoff NA, Spencer D, et al. Occupational exposure of health care workers in kinshasa Democratic Republic of the Congo. *Am J Tropic Med Hygiene*. Published online 2018.

Patel A, D’Alessandro MM, Ireland KJ, Burel WG, Wencil EB, Rasmussen SA. Personal Protective Equipment Supply Chain: Lessons Learned from Recent Public Health Emergency Responses. *Health Secur*. 2017;15(3):244-252. doi:[10.1089/hs.2016.0129](https://doi.org/10.1089/hs.2016.0129)

Raj D, Hornsey E, Perl TM. Personal protective equipment for viral hemorrhagic fevers: *Current Opinion in Infectious Diseases*. 2019;32(4):337-347. doi:[10.1097/QCO.0000000000000562](https://doi.org/10.1097/QCO.0000000000000562)

Raj D. What Are the Appropriate Personal Protective Equipment (PPE) for Front-line Workers (FLW) Caring for Filovirus/Ebola Virus Disease (EVD) Patients? *Open Forum Infectious Diseases*. 2017;4(suppl_1):S170-S170. doi:[10.1093/ofid/ofx163.302](https://doi.org/10.1093/ofid/ofx163.302)

Selvaraj SA, Lee KE, Harrell M, Ivanov I, Allegranzi B. Infection Rates and Risk Factors for Infection Among Health Workers During Ebola and Marburg Virus Outbreaks: A Systematic Review. *The Journal of Infectious Diseases*. 2018;218(suppl_5):S679-S689. doi:[10.1093/infdis/jiy435](https://doi.org/10.1093/infdis/jiy435)

Sprecher AG, Caluwaerts A, Draper M, et al. Personal Protective Equipment for Filovirus Epidemics: A Call for Better Evidence. *J Infect Dis*. 2015;212(suppl 2):S98-S100. doi:[10.1093/infdis/jiv153](https://doi.org/10.1093/infdis/jiv153)

Verbeek JH, Rajamaki B, Ijaz S, et al. Personal protective equipment for preventing highly infectious diseases due to exposure to contaminated body fluids in healthcare staff. Cochrane Work Group, ed. *Cochrane Database of Systematic Reviews*. Published online April 15, 2020. doi:[10.1002/14651858.CD011621.pub4](https://doi.org/10.1002/14651858.CD011621.pub4)

(Wayne) Wang YF. Ebola bio-safety and laboratory testing. *Journal of Microbiology, Immunology and Infection*. 2015;48(2):S17. doi:[10.1016/j.jmii.2015.02.161](https://doi.org/10.1016/j.jmii.2015.02.161)

Kogutt BK, Sheffield JS, Garibaldi BT. 680: Assessing effectiveness of PPE in a simulated SVD of a highly infectious disease patient. *American Journal of Obstetrics and Gynecology*. 2019;220(1):S451. doi:[10.1016/j.ajog.2018.11.703](https://doi.org/10.1016/j.ajog.2018.11.703)

Garibaldi BT, Ruparelia C, Shaw-Saliba K, et al. A novel personal protective equipment coverall was rated higher than standard Ebola virus personal protective equipment in terms of comfort, mobility and perception of safety when tested by health care workers in Liberia and in a United States biocontainment unit. *American Journal of Infection Control*. 2019;47(3):298-304. doi:[10.1016/j.ajic.2018.08.014](https://doi.org/10.1016/j.ajic.2018.08.014)

Poller B, Tunbridge A, Hall S, et al. A unified personal protective equipment ensemble for clinical response to possible high consequence infectious diseases: A consensus document on behalf of the HCID programme. *Journal of Infection*. 2018;77(6):496-502. doi:[10.1016/j.jinf.2018.08.016](https://doi.org/10.1016/j.jinf.2018.08.016)

Poller B, Hall S, Bailey C, et al. ‘VIOLET’: a fluorescence-based simulation exercise for training healthcare workers in the use of personal protective equipment. *Journal of Hospital Infection*. 2018;99(2):229-235. doi:[10.1016/j.jhin.2018.01.021](https://doi.org/10.1016/j.jhin.2018.01.021)

Lee M a, Huh K, Jeong J, et al. Adherence to Protocols by Healthcare Workers and Self-Contamination During Doffing of Personal Protective Equipment. *American Journal of Infection Control*. 2018;46(6):S11. doi:[10.1016/j.ajic.2018.04.024](https://doi.org/10.1016/j.ajic.2018.04.024)

Kwon JH, Burnham CAD, Reske KA, et al. Assessment of Healthcare Worker Protocol Deviations and Self-Contamination During Personal Protective Equipment Donning and Doffing. *Infect Control Hosp Epidemiol*. 2017;38(9):1077-1083. doi:[10.1017/ice.2017.121](https://doi.org/10.1017/ice.2017.121)

Casanova LM, Teal LJ, Sickbert-Bennett EE, et al. Assessment of Self-Contamination During Removal of Personal Protective Equipment for Ebola Patient Care. *Infect Control Hosp Epidemiol*. 2016;37(10):1156-1161. doi:[10.1017/ice.2016.169](https://doi.org/10.1017/ice.2016.169)

Drew JL, Turner J, Mugele J, et al. Beating the Spread: Developing a Simulation Analog for Contagious Body Fluids. *Simulation in Healthcare: The Journal of the Society for Simulation in Healthcare*. 2016;11(2):100-105. doi:[10.1097/SIH.0000000000000157](https://doi.org/10.1097/SIH.0000000000000157)

Mumma JM, Durso FT, Casanova LM, et al. Common Behaviors and Faults When Doffing Personal Protective Equipment for Patients With Serious Communicable Diseases. *Clinical Infectious Diseases*. 2019;69(Supplement_3):S214-S220. doi:[10.1093/cid/ciz614](https://doi.org/10.1093/cid/ciz614)

Quinn T, Kim JH, Seo Y, Coca A. Comparison of Thermal Manikin Modeling and Human Subjects’ Response During Use of Cooling Devices Under Personal Protective Ensembles in the Heat. *Prehosp Disaster med*. 2018;33(3):279-287. doi:[10.1017/S1049023X18000328](https://doi.org/10.1017/S1049023X18000328)

Lim SM, Cha WC, Chae MK, Jo IJ. Contamination during doffing of personal protective equipment by healthcare providers. *Clin Exp Emerg Med*. 2015;2(3):162-167. doi:[10.15441/ceem.15.019](https://doi.org/10.15441/ceem.15.019)

Bell T, Smoot J, Patterson J, Smalligan R, Jordan R. Ebola virus disease: The use of fluorescents as markers of contamination for personal protective equipment. *IDCases*. 2015;2(1):27-30. doi:[10.1016/j.idcr.2014.12.003](https://doi.org/10.1016/j.idcr.2014.12.003)

Andonian J, Kazi S, Therkorn J, et al. Effect of an Intervention Package and Teamwork Training to Prevent Healthcare Personnel Self-contamination During Personal Protective Equipment Doffing. *Clinical Infectious Diseases*. 2019;69(Supplement_3):S248-S255. doi:[10.1093/cid/ciz618](https://doi.org/10.1093/cid/ciz618)

Kiiza P, Mullin SI, Teo K, et al. Establishing Healthcare Worker Performance and Safety in Providing Critical Care for Patients in a Simulated Ebola Treatment Unit: Non-Randomized Pilot Study. *Viruses*. 2021;13(11):2205. doi:[10.3390/v13112205](https://doi.org/10.3390/v13112205)

Kwon JH, Burnham CAD, Reske K, et al. Healthcare Worker Self-Contamination During Standard and Ebola Virus Disease Personal Protective Equipment Doffing. *Open Forum Infectious Diseases*. 2016;3(suppl_1):1387. doi:[10.1093/ofid/ofw172.1090](https://doi.org/10.1093/ofid/ofw172.1090)

Mumma JM, Durso FT, Ferguson AN, et al. Human Factors Risk Analyses of a Doffing Protocol for Ebola-Level Personal Protective Equipment: Mapping Errors to Contamination. *Clinical Infectious Diseases*. 2018;66(6):950-958. doi:[10.1093/cid/cix957](https://doi.org/10.1093/cid/cix957)

Maynard SL, Kao R, Craig D. Impact of personal protective equipment on clinical output and perceived exertion. *J R Army Med Corps*. 2016;162(3):180-183. doi:[10.1136/jramc-2015-000541](https://doi.org/10.1136/jramc-2015-000541)

Reidy P, Fletcher T, Shieber C, et al. Personal protective equipment solution for UK military medical personnel working in an Ebola virus disease treatment unit in Sierra Leone. *Journal of Hospital Infection*. 2017;96(1):42-48. doi:[10.1016/j.jhin.2017.03.018](https://doi.org/10.1016/j.jhin.2017.03.018)

Suen LKP, Guo YP, Tong DWK, et al. Self-contamination during doffing of personal protective equipment by healthcare workers to prevent Ebola transmission. *Antimicrob Resist Infect Control*. 2018;7(1):157. doi:[10.1186/s13756-018-0433-y](https://doi.org/10.1186/s13756-018-0433-y)

**No relevant outcome data**

Brown C, Matthews D, Thomas R, Edens A. Developing a Personal Protective Equipment Selection Matrix for Preventing Occupational Exposure to Ebola Virus.

Fischer WA, Weber DJ, Wohl DA. Personal Protective Equipment: Protecting Health Care Providers in an Ebola Outbreak. *Clinical Therapeutics*. 2015;37(11):2402-2410. doi:[10.1016/j.clinthera.2015.07.007](https://doi.org/10.1016/j.clinthera.2015.07.007)

Potter AW, Gonzalez JA, Xu X. Ebola Response: Modeling the Risk of Heat Stress from Personal Protective Clothing. Bouchama A, ed. *PLoS ONE*. 2015;10(11):e0143461. doi:[10.1371/journal.pone.0143461](https://doi.org/10.1371/journal.pone.0143461)

Roberts R. To PAPR or not to PAPR? *Can J respir ther*. 2014;50(3):87-90.

**Full text Unavailable**

Bosl E, Dersch W, Fehling SK, et al. Ebola virus disease - handling of personal protective equipment (ppe). [German]. *Intensiv- und Notfallbehandlung*. Published online 2014.

Chu M, Bausch D, Velazquez-Berumen A, Vallenas C, A. Committees. Report from the world health organization’s advisory committees on innovative personal protective equipment for front line health workers. 2017;American Journal of Tropical Medicine and Hygiene.

Lee MH, Meerbach A, Straub J, et al. Which personal protective equipment to provide?-Challenges during the Ebola outbreak and lessons learned. *Tropical Medicine and International Health*. Published online 2017.

With strengthened guidelines for health care workers, the CDC ups its game against the deadly Ebola virus. *ED management : the monthly update on emergency department management*. 2014;26(12):133-136.

Kiiza P, Mullin S, Teo K, et al. Establishing feasibility & safety of providing critical care for patients with Ebola through design of a simulated Ebola treatment unit. *Canadian journal of anesthesia*. Published online 2019.

Drew J, Turner J, Cooper D, Zaiser R, Duncan T, Mugele J. Novel use of ultraviolet tracer contagion in multiple-patient simulation and the effect of personal protective equipment on contagion spread: A feasibility study. *Academic Emergency Medicine*. Published online 2015.

Ortega R, Bhadelia N, Obanor O, et al. Putting On and Removing Personal Protective Equipment. *N Engl J Med*. 2015;372(12):e16. doi:[10.1056/NEJMvcm1412105](https://doi.org/10.1056/NEJMvcm1412105)

Lien YH, Wang HH, Hsu FH, Lin LC, Chern JPS, Tsai HC. Response to prevent from Ebola virus infection: Take east Taiwan strain hospital as an example. *Journal of Microbiology Immunology and Infection*. Published online 2015.

Garibaldi BT, Rainwater-Lovett K, Pilholski T, et al. Transmission of fluorescent aerosolized particles in a clinical biocontainment unit. *American Journal of Respiratory and Critical Care Medicine Conference: American Thoracic Society International Conference ATS*. Published online 2017.

Duplicate

Coca A, DiLeo T, Kim JH, Roberge R, Shaffer R. Baseline Evaluation With a Sweating Thermal Manikin of Personal Protective Ensembles Recommended for Use in West Africa. *Disaster med public health prep*. 2015;9(5):536-542. doi:[10.1017/dmp.2015.97](https://doi.org/10.1017/dmp.2015.97)

Coca A, Quinn T, Kim JH, et al. Physiological Evaluation of Personal Protective Ensembles Recommended for Use in West Africa. *Disaster med public health prep*. 2017;11(5):580-586. doi:[10.1017/dmp.2017.13](https://doi.org/10.1017/dmp.2017.13)

KQ6

**Intervention not of interest**

Andonian J, Kazi S, Therkorn J, et al. Effect of an Intervention Package and Teamwork Training to Prevent Healthcare Personnel Self-contamination During Personal Protective Equipment Doffing. Clinical Infectious Diseases. 2019;69(Supplement_3):S248-S255. doi:[10.1093/cid/ciz618](https://doi.org/10.1093/cid/ciz618)

Bell T, Smoot J, Patterson J, Smalligan R, Jordan R. Ebola virus disease: The use of fluorescents as markers of contamination for personal protective equipment. IDCases. 2015;2(1):27-30. doi:[10.1016/j.idcr.2014.12.003](https://doi.org/10.1016/j.idcr.2014.12.003)

Casanova LM, Teal LJ, Sickbert-Bennett EE, et al. Assessment of Self-Contamination During Removal of Personal Protective Equipment for Ebola Patient Care. Infect Control Hosp Epidemiol. 2016;37(10):1156-1161. doi:[10.1017/ice.2016.169](https://doi.org/10.1017/ice.2016.169)

Cummings KJ, Choi MJ, Esswein EJ, et al. Addressing Infection Prevention and Control in the First U.S. Community Hospital to Care for Patients With Ebola Virus Disease: Context for National Recommendations and Future Strategies. Ann Intern Med. 2016;165(1):41. doi:[10.7326/M15-2944](https://doi.org/10.7326/M15-2944)

Ho L, Ratnayake R, Brown H, et al. Precious. Lifesaving but not without problems; a mixed-methods study examining barriers and facilitators to infection prevention and control in health facilities during the ebola virus disease epidemic in Sierra Leone. Published online 2015.

Kogutt BK, Sheffield JS, Garibaldi BT. 680: Assessing effectiveness of PPE in a simulated SVD of a highly infectious disease patient. American Journal of Obstetrics and Gynecology. 2019;220(1):S451. doi:[10.1016/j.ajog.2018.11.703](https://doi.org/10.1016/j.ajog.2018.11.703)

Kwon JH, Burnham CAD, Reske K, et al. Healthcare Worker Self-Contamination During Standard and Ebola Virus Disease Personal Protective Equipment Doffing. Open Forum Infectious Diseases. 2016;3(suppl_1):1387. doi:[10.1093/ofid/ofw172.1090](https://doi.org/10.1093/ofid/ofw172.1090)

Lee M a, Huh K, Jeong J, et al. Adherence to Protocols by Healthcare Workers and Self-Contamination During Doffing of Personal Protective Equipment. American Journal of Infection Control. 2018;46(6):S11. doi:[10.1016/j.ajic.2018.04.024](https://doi.org/10.1016/j.ajic.2018.04.024)

Roberts R. To PAPR or not to PAPR? Can J respir ther. 2014;50(3):87-90.

**No relevant comparisons**

Abela N, Bonnici ET, Parascandalo A, Borg M. Lessons learnt and challenges in adopting the ECDC and who Ebola guidelines at Mater Dei Hospital. Antimicrob Resist Infect Control. 2015;4(S1):P5, 2047-2994-4-S1-P5. doi:10.1186/2047-2994-4-S1-P5

Adeke AS, Onoh RC, Umeokonkwo CD, Azuogu BN, Ogah EO. Knowledge, attitude and practice of infection prevention and control among healthcare workers: one year after an outbreak of nosocomial Lassa fever in a tertiary hospital in southeast NigeriaKnowledge, attitude and practice of infection prevention and con. Af J Clin Exp Micro. 2021;22(4):457-464. doi:10.4314/ajcem.v22i4.5

Adler MD, Krug S, Eiger C, et al. Impact of Personal Protective Equipment on the Performance of Emergency Pediatric Tasks. Pediatr Emer Care. 2021;37(12):e1326-e1330. doi:10.1097/PEC.0000000000002028

Andonian J, Kazi S, Therkorn J, et al. Effect of an Intervention Package and Teamwork Training to Prevent Healthcare Personnel Self-contamination During Personal Protective Equipment Doffing. Clinical Infectious Diseases. 2019;69(Supplement_3):S248-S255. doi:10.1093/cid/ciz618

Brown L, Munro J, Rogers S. Use of personal protective equipment in nursing practice. Nursing Standard. 2019;34(5):59-66. doi:10.7748/ns.2019.e11260

Casalino E, Astocondor E, Sanchez JC, Díaz-Santana DE, del Aguila C, Carrillo JP. Personal protective equipment for the Ebola virus disease: A comparison of 2 training programs. American Journal of Infection Control. 2015;43(12):1281-1287. doi:10.1016/j.ajic.2015.07.007

Casanova LM, Teal LJ, Sickbert-Bennett EE, et al. Assessment of Self-Contamination During Removal of Personal Protective Equipment for Ebola Patient Care. Infect Control Hosp Epidemiol. 2016;37(10):1156-1161. doi:10.1017/ice.2016.169

Coca A, DiLeo T, Kim JH, Roberge R, Shaffer R. Baseline Evaluation With a Sweating Thermal Manikin of Personal Protective Ensembles Recommended for Use in West Africa. Disaster med public health prep. 2015;9(5):536-542. doi:10.1017/dmp.2015.97

Coca A, Quinn T, Kim JH, et al. Physiological Evaluation of Personal Protective Ensembles Recommended for Use in West Africa. Disaster med public health prep. 2017;11(5):580-586. doi:10.1017/dmp.2017.13

Cummings KJ, Choi MJ, Esswein EJ, et al. Addressing Infection Prevention and Control in the First U.S. Community Hospital to Care for Patients With Ebola Virus Disease: Context for National Recommendations and Future Strategies. Ann Intern Med. 2016;165(1):41. doi:10.7326/M15-2944

Dan-Nwafor CC, Ipadeola O, Smout E, et al. A cluster of nosocomial Lassa fever cases in a tertiary health facility in Nigeria: Description and lessons learned, 2018. International Journal of Infectious Diseases. 2019;83:88-94. doi:10.1016/j.ijid.2019.03.030

Den Boon S, Vallenas C, Ferri M, Norris SL. Incorporating health workers’ perspectives into a WHO guideline on personal protective equipment developed during  an Ebola virus disease outbreak. F1000Research. 2018;7(45).

Doshi RH, Hoff NA, Bratcher A, et al. Risk Factors for Ebola Exposure in Health Care Workers in Boende, Tshuapa Province, Democratic Republic of the Congo. The Journal of Infectious Diseases. 2022;226(4):608-615. doi:10.1093/infdis/jiaa747

Doshi RH, Hoff NA, Mukadi P, et al. Seroprevalence of ebola virus among health care workers in the Tshuapa district democratic republic of congo. Am J Tropic Med Hygiene. Published online 2016.

Drew JL, Turner J, Mugele J, et al. Beating the Spread: Developing a Simulation Analog for Contagious Body Fluids. Simulation in Healthcare: The Journal of the Society for Simulation in Healthcare. 2016;11(2):100-105. doi:10.1097/SIH.0000000000000157

Dunn AC, Walker TA, Redd J, et al. Nosocomial transmission of Ebola virus disease on pediatric and maternity wards: Bombali and Tonkolili, Sierra Leone, 2014. American Journal of Infection Control. 2016;44(3):269-272. doi:10.1016/j.ajic.2015.09.016

Fischer WA, Weber DJ, Wohl DA. Personal Protective Equipment: Protecting Health Care Providers in an Ebola Outbreak. Clinical Therapeutics. 2015;37(11):2402-2410. doi:10.1016/j.clinthera.2015.07.007

Garibaldi B, Ernst N, Reimers M, et al. Establishing a New Biocontainment and Treatment Unit. Chest. 2015;148(4):248A. doi:10.1378/chest.2268190

Garibaldi BT, Kelen GD, Brower RG, et al. The Creation of a Biocontainment Unit at a Tertiary Care Hospital. The Johns Hopkins Medicine Experience. Annals ATS. 2016;13(5):600-608. doi:10.1513/AnnalsATS.201509-587PS

Garibaldi BT, Ruparelia C, Shaw-Saliba K, et al. A novel personal protective equipment coverall was rated higher than standard Ebola virus personal protective equipment in terms of comfort, mobility and perception of safety when tested by health care workers in Liberia and in a United States biocontainment unit. American Journal of Infection Control. 2019;47(3):298-304. doi:10.1016/j.ajic.2018.08.014

Grassi De Miranda B, Cais D, Nunes J, Duarte L, Moura ML, Costa A. Mass Gatherings: Experience and Difficulties in Elaborating an Ebola Virus Disease Outbreak Response Plan in a Private Hospital in Brazil. American Journal of Infection Control. 2016;44(6):S123-S124. doi:10.1016/j.ajic.2016.04.148

Grélot L, Koulibaly F, Maugey N, et al. Moderate Thermal Strain in Healthcare Workers Wearing Personal Protective Equipment During Treatment and Care Activities in the Context of the 2014 Ebola Virus Disease Outbreak. J Infect Dis. 2016;213(9):1462-1465. doi:10.1093/infdis/jiv585

Gurses AP, Dietz AS, Nowakowski E, et al. Human factors–based risk analysis to improve the safety of doffing enhanced personal protective equipment. Infect Control Hosp Epidemiol. 2019;40(2):178-186. doi:10.1017/ice.2018.292

Hall S, Poller B, Bailey C, et al. Use of ultraviolet-fluorescence-based simulation in evaluation of personal protective equipment worn for first assessment and care of a patient with suspected high-consequence infectious disease. Journal of Hospital Infection. 2018;99(2):218-228. doi:10.1016/j.jhin.2018.01.002

Hall S, Poller B, Bailey C, et al. Use of ultraviolet-fluorescence-based simulation in evaluation of personal protective equipment worn for first assessment and care of a patient with suspected high-consequence infectious disease. Journal of Hospital Infection. 2018;99(2):218-228. doi:10.1016/j.jhin.2018.01.002

Hersi M, Stevens A, Quach P, et al. Effectiveness of Personal Protective Equipment for Healthcare Workers Caring for Patients with Filovirus Disease: A Rapid Review. Kuhn JH, ed. PLoS ONE. 2015;10(10):e0140290. doi:10.1371/journal.pone.0140290

Hoff NA, Mwanza A, Doshi RH, et al. Possible high exposure to ebola among non-formal health care providers in a previous outbreak site boende democratic republic of congo. The Journal of Infectious Diseases. 2016;219:517-525.

Holt A, Hornsey E, Seale AC, et al. A mixed-methods analysis of personal protective equipment used in Lassa fever treatment centres in Nigeria. Infection Prevention in Practice. 2021;3(3):100168. doi:10.1016/j.infpip.2021.100168

Honda H, Iwata K. Personal protective equipment and improving compliance among healthcare workers in high-risk settings: Current Opinion in Infectious Diseases. 2016;29(4):400-406. doi:10.1097/QCO.0000000000000280

Huber K, Jones I, Dousa T, et al. An Evidence Based Approach to Testing PPE for Enhanced Isolation Precautions during Ebola Virus Disease Preparedness Planning. American Journal of Infection Control. 2015;43(6):S69. doi:10.1016/j.ajic.2015.04.170

Ijarotimi IT, Ilesanmi OS, Aderinwale A, Abiodun-Adewusi O. Knowledge of Lassa fever and use of infection prevention and control facilities among health care workers during Lassa fever outbreak in Ondo State, Nigeria. :13.

Kwon JH, Burnham CAD, Reske KA, et al. Assessment of Healthcare Worker Protocol Deviations and Self-Contamination During Personal Protective Equipment Donning and Doffing. Infect Control Hosp Epidemiol. 2017;38(9):1077-1083. doi:10.1017/ice.2017.121

Licina A, Silvers A, Stuart RL. Use of powered air-purifying respirator (PAPR) by healthcare workers for preventing highly infectious viral diseases—a systematic review of evidence. Syst Rev. 2020;9(1):173. doi:10.1186/s13643-020-01431-5

Lim SM, Cha WC, Chae MK, Jo IJ. Contamination during doffing of personal protective equipment by healthcare providers. Clin Exp Emerg Med. 2015;2(3):162-167. doi:10.15441/ceem.15.019

MacIntyre CR, Chughtai AA, Seale H, Richards GA, Davidson PM. Respiratory protection for healthcare workers treating Ebola virus disease (EVD): Are facemasks sufficient to meet occupational health and safety obligations? International Journal of Nursing Studies. 2014;51(11):1421-1426. doi:10.1016/j.ijnurstu.2014.09.002

MacIntyre CR, Chughtai AA, Seale H, Richards GA, Davidson PM. Response to Martin-Moreno et al. (2014) Surgical mask or no mask for health workers not a defensible position for Ebola. International Journal of Nursing Studies. 2014;51(12):1694-1695. doi:10.1016/j.ijnurstu.2014.10.004

MacIntyre CR, Chughtai AA, Seale H, Richards GA, Davidson PM. Uncertainty, risk analysis and change for Ebola personal protective equipment guidelines. International Journal of Nursing Studies. 2015;52(5):899-903. doi:10.1016/j.ijnurstu.2014.12.001

Martin-Moreno JM, Llinás G, Hernández JM. Is respiratory protection appropriate in the Ebola response? The Lancet. 2014;384(9946):856. doi:10.1016/S0140-6736(14)61343-X

Martin-Moreno JM, Llinás G, Martínez-Hernández J. Response to “MacIntyre et al., 2014: Respiratory protection for healthcare workers treating Ebola virus disease (EVD): are facemasks sufficient to meet occupational health and safety obligations?” International Journal of Nursing Studies. 2014;51(12):1693. doi:10.1016/j.ijnurstu.2014.10.005

Maynard SL, Kao R, Craig D. Impact of personal protective equipment on clinical output and perceived exertion. J R Army Med Corps. 2016;162(3):180-183. doi:10.1136/jramc-2015-000541

Mba S, Ukponu W, Saleh M, et al. Lassa fever infection among health care workers in Nigeria, 2019. International Journal of Infectious Diseases. 2020;101:279. doi:10.1016/j.ijid.2020.09.731

McCulloch KL, Michael F, Goren M, et al. Creating an Environment of Safety for the Treatment of Patients with Ebola. American Journal of Infection Control. 2015;43(6):S73. doi:10.1016/j.ajic.2015.04.193

Mumma JM, Durso FT, Casanova LM, et al. Common Behaviors and Faults When Doffing Personal Protective Equipment for Patients With Serious Communicable Diseases. Clinical Infectious Diseases. 2019;69(Supplement_3):S214-S220. doi:10.1093/cid/ciz614

Mumma JM, Durso FT, Ferguson AN, et al. Human Factors Risk Analyses of a Doffing Protocol for Ebola-Level Personal Protective Equipment: Mapping Errors to Contamination. Clinical Infectious Diseases. 2018;66(6):950-958. doi:10.1093/cid/cix957

Musene KK, Hoff NA, Spencer D, et al. Occupational exposure of health care workers in kinshasa Democratic Republic of the Congo. Am J Tropic Med Hygiene. Published online 2018.

Ngatu NR, Kayembe NJM, Phillips EK, et al. Epidemiology of ebolavirus disease (EVD) and occupational EVD in health care workers in Sub-Saharan Africa: Need for strengthened public health preparedness. Journal of Epidemiology. 2017;27(10):455-461. doi:10.1016/j.je.2016.09.010

Obionu IM, Ochu CL, Ukponu W, et al. Evaluation of infection prevention and control practices in Lassa fever treatment centers in north-central Nigeria during an ongoing Lassa fever outbreak. Journal of Infection Prevention. 2021;22(6):275-282.

Poller B, Hall S, Bailey C, et al. ‘VIOLET’: a fluorescence-based simulation exercise for training healthcare workers in the use of personal protective equipment. Journal of Hospital Infection. 2018;99(2):229-235. doi:10.1016/j.jhin.2018.01.021

Poller B, Tunbridge A, Hall S, et al. A unified personal protective equipment ensemble for clinical response to possible high consequence infectious diseases: A consensus document on behalf of the HCID programme. Journal of Infection. 2018;77(6):496-502. doi:10.1016/j.jinf.2018.08.016

Quinn T, Kim JH, Seo Y, Coca A. Comparison of Thermal Manikin Modeling and Human Subjects’ Response During Use of Cooling Devices Under Personal Protective Ensembles in the Heat. Prehosp Disaster med. 2018;33(3):279-287. doi:10.1017/S1049023X18000328

Raab M, Pfadenhauer LM, Millimouno TJ, Hoelscher M, Froeschl G. Knowledge, attitudes and practices towards viral haemorrhagic fevers amongst healthcare workers in urban and rural public healthcare facilities in the N’zérékoré prefecture, Guinea: a cross-sectional study. BMC Public Health. 2020;20(1):296. doi:10.1186/s12889-020-8433-2

Raj D. What Are the Appropriate Personal Protective Equipment (PPE) for Front-line Workers (FLW) Caring for Filovirus/Ebola Virus Disease (EVD) Patients? Open Forum Infectious Diseases. 2017;4(suppl_1):S170-S170. doi:10.1093/ofid/ofx163.302

Raj D. What Are the Appropriate Personal Protective Equipment (PPE) for Front-line Workers (FLW) Caring for Filovirus/Ebola Virus Disease (EVD) Patients? Open Forum Infectious Diseases. 2017;4(suppl_1):S170-S170. doi:10.1093/ofid/ofx163.302

Ratnayake R, Ho LS, Ansumana R, et al. Improving Ebola infection prevention and control in primary healthcare facilities in Sierra Leone: a single-group pretest post-test, mixed-methods study. BMJ Glob Health. 2016;1(4):e000103. doi:10.1136/bmjgh-2016-000103

Reddy SC, Valderrama AL, Kuhar DT. Improving the Use of Personal Protective Equipment: Applying Lessons Learned. Clinical Infectious Diseases. 2019;69(Supplement_3):S165-S170. doi:10.1093/cid/ciz619

Reidy P, Fletcher T, Shieber C, et al. Personal protective equipment solution for UK military medical personnel working in an Ebola virus disease treatment unit in Sierra Leone. Journal of Hospital Infection. 2017;96(1):42-48. doi:10.1016/j.jhin.2017.03.018

Selvaraj SA, Lee KE, Harrell M, Ivanov I, Allegranzi B. Infection Rates and Risk Factors for Infection Among Health Workers During Ebola and Marburg Virus Outbreaks: A Systematic Review. The Journal of Infectious Diseases. 2018;218(suppl_5):S679-S689. doi:10.1093/infdis/jiy435

Sprecher AG, Caluwaerts A, Draper M, et al. Personal Protective Equipment for Filovirus Epidemics: A Call for Better Evidence. J Infect Dis. 2015;212(suppl 2):S98-S100. doi:10.1093/infdis/jiv153

Tartari E, Parascandalo AF, Borg M. Ensuring healthcare workers’ safety in the management of Ebola virus disease: a novel competency assessment checklist for proper PPE use. Antimicrob Resist Infect Control. 2015;4(S1):P6, 2047-2994-4-S1-P6. doi:10.1186/2047-2994-4-S1-P6

Ukwenya VO, Fuwape TA, Fadahunsi TI, Ilesanmi OS. Disparities in knowledge, attitude, and practices of infection prevention and control of lassa fever among health care workers at The Federal Medical Centre, Owo, Ondo State, Nigeria. Pan Afr Med J. 2021;38. doi:10.11604/pamj.2021.38.357.26208

Verbeek JH, Rajamaki B, Ijaz S, et al. Personal protective equipment for preventing highly infectious diseases due to exposure to contaminated body fluids in healthcare staff. Cochrane Work Group, ed. Cochrane Database of Systematic Reviews. Published online April 15, 2020. doi:10.1002/14651858.CD011621.pub4

**No outcome data reported**

Poller B, Tunbridge A, Hall S, et al. A unified personal protective equipment ensemble for clinical response to possible high consequence infectious diseases: A consensus document on behalf of the HCID programme. *Journal of Infection*. 2018;77(6):496-502. doi:[10.1016/j.jinf.2018.08.016](https://doi.org/10.1016/j.jinf.2018.08.016)

Franklin SM. A Comparison of Personal Protective Standards: Caring for Patients With Ebola Virus. *Clinical Nurse Specialist*. 2016;30(2):E1-E8. doi:[10.1097/NUR.0000000000000183](https://doi.org/10.1097/NUR.0000000000000183)

McLaws ML, Chughtai AA, Salmon S, MacIntyre CR. A highly precautionary doffing sequence for health care workers after caring for wet Ebola patients to further reduce occupational acquisition of Ebola. *American Journal of Infection Control*. 2016;44(7):740-744. doi:[10.1016/j.ajic.2015.12.034](https://doi.org/10.1016/j.ajic.2015.12.034)

**Narrative review**

Raj D, Hornsey E, Perl TM. Personal protective equipment for viral hemorrhagic fevers: *Current Opinion in Infectious Diseases*. 2019;32(4):337-347. doi:[10.1097/QCO.0000000000000562](https://doi.org/10.1097/QCO.0000000000000562)

Funk DJ, Kumar A. Ebola virus disease: an update for anesthesiologists and intensivists. *Can J Anesth/J Can Anesth*. 2015;62(1):80-91. doi:[10.1007/s12630-014-0257-z](https://doi.org/10.1007/s12630-014-0257-z)

Mohammed HM. Ebola virus disease: Effects of respiratory protection on healthcare workers. *Egyptian Journal of Chest Diseases and Tuberculosis*. 2015;64(3):639-644. doi:[10.1016/j.ejcdt.2015.04.015](https://doi.org/10.1016/j.ejcdt.2015.04.015)

**Full text Unavailable**

Ortega R, Bhadelia N, Obanor O, et al. Putting On and Removing Personal Protective Equipment. N Engl J Med. 2015;372(12):e16. doi:10.1056/NEJMvcm1412105

Bosl E, Dersch W, Fehling SK, et al. Ebola virus disease - handling of personal protective equipment (ppe). [German]. Intensiv- und Notfallbehandlung. Published online 2014.

Bustamante ND, O’Keeffe D, Bradley D, Pozner CN. Targeted interprofessional simulation-based training for safe patient management of Ebola virus disease. Academic Emergency Medicine. Published online 2015.

Chu M, Bausch D, Velazquez-Berumen A, Vallenas C. Report from the world health organization’s advisory committees on innovative personal protective equipment for front line health workers. American Journal of Tropical Medicine and Hygiene. Published online 2017.

Drew J, Turner J, Cooper D, Zaiser R, Duncan T, Mugele J. Novel use of ultraviolet tracer contagion in multiple-patient simulation and the effect of personal protective equipment on contagion spread: A feasibility study. Academic Emergency Medicine. Published online 2015.

Garibaldi BT, Rainwater-Lovett K, Pilholski T, et al. Transmission of fluorescent aerosolized particles in a clinical biocontainment unit. American Journal of Respiratory and Critical Care Medicine Conference: American Thoracic Society International Conference ATS. Published online 2017.

Hotta H. [Viral hemorrhagic fever--Ebola hemorrhagic fever, Marburg disease and Lassa fever]. Rinsho Byori. 1998;46(7):651-655.

Jalloh MB, Chu MC. A doctor’s experience: The dilemma faced using personal protective equipment while working in an ebola treatment unit. American Journal of Tropical Medicine and Hygiene. Published online 2019.

Kratz T, Verbeek L. Discussion of two infection prevention and control training approaches to enhance biosafety in primary healthcare facilities during an outbreak of Ebola virus disease. Tropical Medicine and International Health. Published online 2017.

Lee MH, Meerbach A, Straub J, et al. Which personal protective equipment to provide?-Challenges during the Ebola outbreak and lessons learned. Tropical Medicine and International Health. Published online 2017.

Pang V, Carter Y, Scott J, Salazar G, Johnson V. How to use personal protective equipment. Nurs Times. 2014;110(51):14-16.

Somers Y, Verbiest M. Suspecting ebola: When the dress code becomes life saving! Personal protective equipment-a practical demonstration. Anaesthesiology Intensive Therapy. Published online 2014.

With strengthened guidelines for health care workers, the CDC ups its game against the deadly Ebola virus. ED management : the monthly update on emergency department management. 2014;26(12):133-136.

Duplicate

Suen LKP, Guo YP, Tong DWK, et al. Self-contamination during doffing of personal protective equipment by healthcare workers to prevent Ebola transmission. *Antimicrob Resist Infect Control*. 2018;7(1):157. doi:[10.1186/s13756-018-0433-y](https://doi.org/10.1186/s13756-018-0433-y)

Study not in english;

Schmiedel S, Kreuels B. [Ebola virus disease in West Africa and Germany : clinical presentation, management and practical experience with medevacuated patients in Germany]. *Bundesgesundheitsblatt Gesundheitsforschung Gesundheitsschutz*. 2015;58(7):679-685. doi:[10.1007/s00103-015-2162-1](https://doi.org/10.1007/s00103-015-2162-1)

KQ8

**Commentary (no outcome data)**

Fischer WA, Weber DJ, Wohl DA. Personal Protective Equipment: Protecting Health Care Providers in an Ebola Outbreak. *Clinical Therapeutics*. 2015;37(11):2402-2410. doi:[10.1016/j.clinthera.2015.07.007](https://doi.org/10.1016/j.clinthera.2015.07.007)

**Intervention not of interest**

Bell T, Smoot J, Patterson J, Smalligan R, Jordan R. Ebola virus disease: The use of fluorescents as markers of contamination for personal protective equipment. *IDCases*. 2015;2(1):27-30. doi:[10.1016/j.idcr.2014.12.003](https://doi.org/10.1016/j.idcr.2014.12.003)

Coca A, Quinn T, Kim JH, et al. Physiological Evaluation of Personal Protective Ensembles Recommended for Use in West Africa. *Disaster med public health prep*. 2017;11(5):580-586. doi:[10.1017/dmp.2017.13](https://doi.org/10.1017/dmp.2017.13)

Drew JL, Turner J, Mugele J, et al. Beating the Spread: Developing a Simulation Analog for Contagious Body Fluids. *Simulation in Healthcare: The Journal of the Society for Simulation in Healthcare*. 2016;11(2):100-105. doi:[10.1097/SIH.0000000000000157](https://doi.org/10.1097/SIH.0000000000000157)

Drews FA, Mulvey D, Stratford K, Samore MH, Mayer J. Evaluation of a Redesigned Personal Protective Equipment Gown. *Clinical Infectious Diseases*. 2019;69(Supplement_3):S199-S205. doi:[10.1093/cid/ciz520](https://doi.org/10.1093/cid/ciz520)

Eiras D, Echeverri A, Toale K, Tennill P, Evans L. Painting the Gown Red: Using a Colored Paint Quality Improvement Process to Evaluate Healthcare Worker Personal Protective Equipment for Highly Pathogenic Infections. *OFID*. 2017;4(Suppl 1).

Jinadatha C, Simmons S, Dale C, et al. Disinfecting personal protective equipment with pulsed xenon ultraviolet as a risk mitigation strategy for health care workers. *American Journal of Infection Control*. 2015;43(4):412-414. doi:[10.1016/j.ajic.2015.01.013](https://doi.org/10.1016/j.ajic.2015.01.013)

Kilinc-Balci FS, Nwoko J, Hillam T. Evaluation of the Performance of Isolation Gowns. *American Journal of Infection Control*. 2015;43(6):S44. doi:[10.1016/j.ajic.2015.04.112](https://doi.org/10.1016/j.ajic.2015.04.112)

Kilinc Balci FS. Isolation gowns in health care settings: Laboratory studies, regulations and standards, and potential barriers of gown selection and use. *American Journal of Infection Control*. 2016;44(1):104-111. doi:[10.1016/j.ajic.2015.07.042](https://doi.org/10.1016/j.ajic.2015.07.042)

Koenig K, Majestic C, Burns M. Ebola Virus Disease: Essential Public Health Principles for Clinicians. *WestJEM*. 2014;15(7):728-731. doi:[10.5811/westjem.2014.9.24011](https://doi.org/10.5811/westjem.2014.9.24011)

Kogutt BK, Sheffield JS, Garibaldi BT. 680: Assessing effectiveness of PPE in a simulated SVD of a highly infectious disease patient. *American Journal of Obstetrics and Gynecology*. 2019;220(1):S451. doi:[10.1016/j.ajog.2018.11.703](https://doi.org/10.1016/j.ajog.2018.11.703)

Poller B, Tunbridge A, Hall S, et al. A unified personal protective equipment ensemble for clinical response to possible high consequence infectious diseases: A consensus document on behalf of the HCID programme. *Journal of Infection*. 2018;77(6):496-502. doi:[10.1016/j.jinf.2018.08.016](https://doi.org/10.1016/j.jinf.2018.08.016)

Poller B, Hall S, Bailey C, et al. ‘VIOLET’: a fluorescence-based simulation exercise for training healthcare workers in the use of personal protective equipment. *Journal of Hospital Infection*. 2018;99(2):229-235. doi:[10.1016/j.jhin.2018.01.021](https://doi.org/10.1016/j.jhin.2018.01.021)

Raj D, Hornsey E, Perl TM. Personal protective equipment for viral hemorrhagic fevers: *Current Opinion in Infectious Diseases*. 2019;32(4):337-347. doi:[10.1097/QCO.0000000000000562](https://doi.org/10.1097/QCO.0000000000000562)

Roberts R. To PAPR or not to PAPR? *Can J respir ther*. 2014;50(3):87-90.

Licina A, Silvers A, Stuart RL. Use of powered air-purifying respirator (PAPR) by healthcare workers for preventing highly infectious viral diseases—a systematic review of evidence. *Syst Rev*. 2020;9(1):173. doi:[10.1186/s13643-020-01431-5](https://doi.org/10.1186/s13643-020-01431-5)

**No information on PPE**

Huber K, Jones I, Dousa T, et al. An Evidence Based Approach to Testing PPE for Enhanced Isolation Precautions during Ebola Virus Disease Preparedness Planning. *American Journal of Infection Control*. 2015;43(6):S69. doi:[10.1016/j.ajic.2015.04.170](https://doi.org/10.1016/j.ajic.2015.04.170)

Lee M a, Huh K, Jeong J, et al. Adherence to Protocols by Healthcare Workers and Self-Contamination During Doffing of Personal Protective Equipment. *American Journal of Infection Control*. 2018;46(6):S11. doi:[10.1016/j.ajic.2018.04.024](https://doi.org/10.1016/j.ajic.2018.04.024)

Maynard SL, Kao R, Craig D. Impact of personal protective equipment on clinical output and perceived exertion. *J R Army Med Corps*. 2016;162(3):180-183. doi:[10.1136/jramc-2015-000541](https://doi.org/10.1136/jramc-2015-000541)

**No relevant comparisons**

Andonian J, Kazi S, Therkorn J, et al. Effect of an Intervention Package and Teamwork Training to Prevent Healthcare Personnel Self-contamination During Personal Protective Equipment Doffing. *Clinical Infectious Diseases*. 2019;69(Supplement_3):S248-S255. doi:[10.1093/cid/ciz618](https://doi.org/10.1093/cid/ciz618)

Casanova LM, Erukunuakpor K, Kraft CS, et al. Assessing Viral Transfer During Doffing of Ebola-Level Personal Protective Equipment in a Biocontainment Unit. *Clinical Infectious Diseases*. 2018;66(6):945-949. doi:[10.1093/cid/cix956](https://doi.org/10.1093/cid/cix956)

Casanova LM, Teal LJ, Sickbert-Bennett EE, et al. Assessment of Self-Contamination During Removal of Personal Protective Equipment for Ebola Patient Care. *Infect Control Hosp Epidemiol*. 2016;37(10):1156-1161. doi:[10.1017/ice.2016.169](https://doi.org/10.1017/ice.2016.169)

Chughtai AA, Chen X, Macintyre CR. Risk of self-contamination during doffing of personal protective equipment. *American Journal of Infection Control*. 2018;46(12):1329-1334. doi:[10.1016/j.ajic.2018.06.003](https://doi.org/10.1016/j.ajic.2018.06.003)

Den Boon S, Vallenas C, Ferri M, Norris SL. Incorporating health workers’ perspectives into a WHO guideline on personal protective equipment developed during  an Ebola virus disease outbreak. *F1000Research*. 2018;7(45).

Garibaldi BT, Ruparelia C, Shaw-Saliba K, et al. A novel personal protective equipment coverall was rated higher than standard Ebola virus personal protective equipment in terms of comfort, mobility and perception of safety when tested by health care workers in Liberia and in a United States biocontainment unit. *American Journal of Infection Control*. 2019;47(3):298-304. doi:[10.1016/j.ajic.2018.08.014](https://doi.org/10.1016/j.ajic.2018.08.014)

Grélot L, Koulibaly F, Maugey N, et al. Moderate Thermal Strain in Healthcare Workers Wearing Personal Protective Equipment During Treatment and Care Activities in the Context of the 2014 Ebola Virus Disease Outbreak. *J Infect Dis*. 2016;213(9):1462-1465. doi:[10.1093/infdis/jiv585](https://doi.org/10.1093/infdis/jiv585)

Hall S, Poller B, Bailey C, et al. Use of ultraviolet-fluorescence-based simulation in evaluation of personal protective equipment worn for first assessment and care of a patient with suspected high-consequence infectious disease. *Journal of Hospital Infection*. 2018;99(2):218-228. doi:[10.1016/j.jhin.2018.01.002](https://doi.org/10.1016/j.jhin.2018.01.002)

Hersi M, Stevens A, Quach P, et al. Effectiveness of Personal Protective Equipment for Healthcare Workers Caring for Patients with Filovirus Disease: A Rapid Review. Kuhn JH, ed. *PLoS ONE*. 2015;10(10):e0140290. doi:[10.1371/journal.pone.0140290](https://doi.org/10.1371/journal.pone.0140290)

Jaques PA, Gao P, Kilinc-Balci S, et al. Evaluation of gowns and coveralls used by medical personnel working with Ebola patients against simulated bodily fluids using an Elbow Lean Test. *Journal of Occupational and Environmental Hygiene*. 2016;13(11):881-893. doi:[10.1080/15459624.2016.1186279](https://doi.org/10.1080/15459624.2016.1186279)

Kilinc FS. A Review of Isolation Gowns in Healthcare: Fabric and Gown Properties. *Journal of Engineered Fibers and Fabrics*. 2015;10(3):155892501501000. doi:[10.1177/155892501501000313](https://doi.org/10.1177/155892501501000313)

Kwon JH, Burnham CAD, Reske KA, et al. Assessment of Healthcare Worker Protocol Deviations and Self-Contamination During Personal Protective Equipment Donning and Doffing. *Infect Control Hosp Epidemiol*. 2017;38(9):1077-1083. doi:[10.1017/ice.2017.121](https://doi.org/10.1017/ice.2017.121)

Kwon JH, Burnham CAD, Reske K, et al. Healthcare Worker Self-Contamination During Standard and Ebola Virus Disease Personal Protective Equipment Doffing. *Open Forum Infectious Diseases*. 2016;3(suppl_1):1387. doi:[10.1093/ofid/ofw172.1090](https://doi.org/10.1093/ofid/ofw172.1090)

Mumma JM, Durso FT, Ferguson AN, et al. Human Factors Risk Analyses of a Doffing Protocol for Ebola-Level Personal Protective Equipment: Mapping Errors to Contamination. *Clinical Infectious Diseases*. 2018;66(6):950-958. doi:[10.1093/cid/cix957](https://doi.org/10.1093/cid/cix957)

Perpoint T, Valour F, Gerbier-Colomban S, et al. Knowledge Attitude and Practice (KAP) on Ebola Virus Disease (EVD) Among Health Care Workers (HCWs) From the Lyon Teaching Hospitals, France. *Open Forum Infectious Diseases*. 2016;3(suppl_1):602. doi:[10.1093/ofid/ofw172.465](https://doi.org/10.1093/ofid/ofw172.465)

Polgreen PM, Santibanez S, Koonin LM, Rupp ME, Beekmann SE, del Rio C. Infectious Disease Physician Assessment of Hospital Preparedness for Ebola Virus Disease. *Open Forum Infectious Diseases*. 2015;2(3):ofv087. doi:[10.1093/ofid/ofv087](https://doi.org/10.1093/ofid/ofv087)

Reidy P, Fletcher T, Shieber C, et al. Personal protective equipment solution for UK military medical personnel working in an Ebola virus disease treatment unit in Sierra Leone. *Journal of Hospital Infection*. 2017;96(1):42-48. doi:[10.1016/j.jhin.2017.03.018](https://doi.org/10.1016/j.jhin.2017.03.018)

Suen LKP, Guo YP, Tong DWK, et al. Self-contamination during doffing of personal protective equipment by healthcare workers to prevent Ebola transmission. *Antimicrob Resist Infect Control*. 2018;7(1):157. doi:[10.1186/s13756-018-0433-y](https://doi.org/10.1186/s13756-018-0433-y)

Verbeek JH, Rajamaki B, Ijaz S, et al. Personal protective equipment for preventing highly infectious diseases due to exposure to contaminated body fluids in healthcare staff. Cochrane Work Group, ed. *Cochrane Database of Systematic Reviews*. Published online April 15, 2020. doi:[10.1002/14651858.CD011621.pub4](https://doi.org/10.1002/14651858.CD011621.pub4)

Franklin SM. A Comparison of Personal Protective Standards: Caring for Patients With Ebola Virus. *Clinical Nurse Specialist*. 2016;30(2):E1-E8. doi:[10.1097/NUR.0000000000000183](https://doi.org/10.1097/NUR.0000000000000183)

Cummings KJ, Choi MJ, Esswein EJ, et al. Addressing Infection Prevention and Control in the First U.S. Community Hospital to Care for Patients With Ebola Virus Disease: Context for National Recommendations and Future Strategies. *Ann Intern Med*. 2016;165(1):41. doi:[10.7326/M15-2944](https://doi.org/10.7326/M15-2944)

Coca A, DiLeo T, Kim JH, Roberge R, Shaffer R. Baseline Evaluation With a Sweating Thermal Manikin of Personal Protective Ensembles Recommended for Use in West Africa. *Disaster med public health prep*. 2015;9(5):536-542. doi:[10.1017/dmp.2015.97](https://doi.org/10.1017/dmp.2015.97)

Nikiforuk AM, Cutts TA, Theriault SS, Cook BWM. Challenge of Liquid Stressed Protective Materials and Environmental Persistence of Ebola Virus. *Sci Rep*. 2017;7(1):4388. doi:[10.1038/s41598-017-04137-2](https://doi.org/10.1038/s41598-017-04137-2)

Mumma JM, Durso FT, Casanova LM, et al. Common Behaviors and Faults When Doffing Personal Protective Equipment for Patients With Serious Communicable Diseases. *Clinical Infectious Diseases*. 2019;69(Supplement_3):S214-S220. doi:[10.1093/cid/ciz614](https://doi.org/10.1093/cid/ciz614)

Lim SM, Cha WC, Chae MK, Jo IJ. Contamination during doffing of personal protective equipment by healthcare providers. *Clin Exp Emerg Med*. 2015;2(3):162-167. doi:[10.15441/ceem.15.019](https://doi.org/10.15441/ceem.15.019)

Wong MF, Matić Z, Campiglia GC, et al. Design Strategies for Biocontainment Units to Reduce Risk During Doffing of High-level Personal Protective Equipment. *Clinical Infectious Diseases*. 2019;69(Supplement_3):S241-S247. doi:[10.1093/cid/ciz617](https://doi.org/10.1093/cid/ciz617)

DuBose JR, Matić Z, Sala MFW, et al. Design strategies to improve healthcare worker safety in biocontainment units: learning from ebola preparedness. *Infect Control Hosp Epidemiol*. 2018;39(8):961-967. doi:[10.1017/ice.2018.125](https://doi.org/10.1017/ice.2018.125)

Mohammed HM. Ebola virus disease: Effects of respiratory protection on healthcare workers. *Egyptian Journal of Chest Diseases and Tuberculosis*. 2015;64(3):639-644. doi:[10.1016/j.ejcdt.2015.04.015](https://doi.org/10.1016/j.ejcdt.2015.04.015)

Baloh J, Reisinger HS, Dukes K, et al. Healthcare Workers’ Strategies for Doffing Personal Protective Equipment. *Clinical Infectious Diseases*. 2019;69(Supplement_3):S192-S198. doi:[10.1093/cid/ciz613](https://doi.org/10.1093/cid/ciz613)

Gurses AP, Dietz AS, Nowakowski E, et al. Human factors–based risk analysis to improve the safety of doffing enhanced personal protective equipment. *Infect Control Hosp Epidemiol*. 2019;40(2):178-186. doi:[10.1017/ice.2018.292](https://doi.org/10.1017/ice.2018.292)

Reddy SC, Valderrama AL, Kuhar DT. Improving the Use of Personal Protective Equipment: Applying Lessons Learned. *Clinical Infectious Diseases*. 2019;69(Supplement_3):S165-S170. doi:[10.1093/cid/ciz619](https://doi.org/10.1093/cid/ciz619)

Dunn AC, Walker TA, Redd J, et al. Nosocomial transmission of Ebola virus disease on pediatric and maternity wards: Bombali and Tonkolili, Sierra Leone, 2014. *American Journal of Infection Control*. 2016;44(3):269-272. doi:[10.1016/j.ajic.2015.09.016](https://doi.org/10.1016/j.ajic.2015.09.016)

Honda H, Iwata K. Personal protective equipment and improving compliance among healthcare workers in high-risk settings: *Current Opinion in Infectious Diseases*. 2016;29(4):400-406. doi:[10.1097/QCO.0000000000000280](https://doi.org/10.1097/QCO.0000000000000280)

Sprecher AG, Caluwaerts A, Draper M, et al. Personal Protective Equipment for Filovirus Epidemics: A Call for Better Evidence. *J Infect Dis*. 2015;212(suppl 2):S98-S100. doi:[10.1093/infdis/jiv153](https://doi.org/10.1093/infdis/jiv153)

Brown L, Munro J, Rogers S. Use of personal protective equipment in nursing practice. *Nursing Standard*. 2019;34(5):59-66. doi:[10.7748/ns.2019.e11260](https://doi.org/10.7748/ns.2019.e11260)

Hall S, Poller B, Bailey C, et al. Use of ultraviolet-fluorescence-based simulation in evaluation of personal protective equipment worn for first assessment and care of a patient with suspected high-consequence infectious disease. *Journal of Hospital Infection*. 2018;99(2):218-228. doi:[10.1016/j.jhin.2018.01.002](https://doi.org/10.1016/j.jhin.2018.01.002)

**Full text Unavailable**

Drew J, Turner J, Cooper D, Zaiser R, Duncan T, Mugele J. Novel use of ultraviolet tracer contagion in multiple-patient simulation and the effect of personal protective equipment on contagion spread: A feasibility study. *Academic Emergency Medicine*. Published online 2015.

Somers Y, Verbiest M. Suspecting ebola: When the dress code becomes life saving! Personal protective equipment-a practical demonstration. *Anaesthesiology Intensive Therapy*. Published online 2014.

Bosl E, Dersch W, Fehling SK, et al. Ebola virus disease - handling of personal protective equipment (ppe). [German]. *Intensiv- und Notfallbehandlung*. Published online 2014.

Sikka MK, Moritz D, Bleasdale SC, Fritzen-Pedicini C, Brosseau L, Jones R. Experiences of hospitals in the Chicago area with ppe acquisition and use during the 2014 ebola virus disease outbreak. *Open Forum Infectious Diseases*. Published online 2017.

Chu M, Bausch D, Velazquez-Berumen A, Vallenas C, A. Committees. Report from the world health organization’s advisory committees on innovative personal protective equipment for front line health workers. *American Journal of Tropical Medicine and Hygiene*. Published online 2017.

Lee MH, Meerbach A, Straub J, et al. Which personal protective equipment to provide?-Challenges during the Ebola outbreak and lessons learned. *Tropical Medicine and International Health*. Published online 2017.

With strengthened guidelines for health care workers, the CDC ups its game against the deadly Ebola virus. *ED management : the monthly update on emergency department management*. 2014;26(12):133-136.

Abstract only

Barratt R, Gilbert L, Shaban R. Healthcare worker personal protective equipment (PPE) training programs in Australia and New Zealand hospitals – a survey. *Infection, Disease & Health*. 2019;24:S3. doi:[10.1016/j.idh.2019.09.011](https://doi.org/10.1016/j.idh.2019.09.011)

Abela N, Bonnici ET, Parascandalo A, Borg M. Lessons learnt and challenges in adopting the ECDC and who Ebola guidelines at Mater Dei Hospital. *Antimicrob Resist Infect Control*. 2015;4(S1):P5, 2047-2994-4-S1-P5. doi:[10.1186/2047-2994-4-S1-P5](https://doi.org/10.1186/2047-2994-4-S1-P5)

# Additional PICO 1 and 2

**Abstract Only**

Abela N, Bonnici ET, Parascandalo A, Borg M. Lessons learnt and challenges in adopting the ECDC and who Ebola guidelines at Mater Dei Hospital. Antimicrob Resist Infect Control. 2015;4(S1):P5, 2047-2994-4-S1-P5. doi:10.1186/2047-2994-4-S1-P5

Doshi RH, Hoff NA, Mukadi P, et al. Seroprevalence of ebola virus among health care workers in the Tshuapa district democratic republic of congo. Am J Tropic Med Hygiene. Published online 2016.

Garde DL, Hall AMR, Marsh RH, Barron KP, Dierberg KL, Koroma AP. Implementation of the first dedicated Ebola screening and isolation for maternity patients in Sierra Leone. Annals of Global Health. 2016;82(3):418. doi:10.1016/j.aogh.2016.04.164

Houlihan CF, Roberts C h., Baguelin M, Mabey D, Tedder R, Glynn JR. Antibodies to Ebola in International Responders to the West Africa Ebola Epidemic.

Huber K, Jones I, Dousa T, et al. An Evidence Based Approach to Testing PPE for Enhanced Isolation Precautions during Ebola Virus Disease Preparedness Planning. American Journal of Infection Control. 2015;43(6):S69. doi:10.1016/j.ajic.2015.04.170

Kilinc-Balci FS, Nwoko J, Hillam T. Evaluation of the Performance of Isolation Gowns. American Journal of Infection Control. 2015;43(6):S44. doi:10.1016/j.ajic.2015.04.112

Lee M a, Huh K, Jeong J, et al. Adherence to Protocols by Healthcare Workers and Self-Contamination During Doffing of Personal Protective Equipment. American Journal of Infection Control. 2018;46(6):S11. doi:10.1016/j.ajic.2018.04.024

Musene KK, Hoff NA, Spencer D, et al. Occupational Exposure of Health Care Workers in Kinshasa, Democratic Republic Of the Congo.

Okamoto K, Rhee Y, Schoeny M, et al. Importance of healthcare worker personal protective equipment in reducing doffing errors-correlation with HCW characteristics and perceptions. Open Forum Infectious Diseases Conference: ID Week. Published online 2016.

Perpoint T, Valour F, Gerbier-Colomban S, et al. Knowledge Attitude and Practice (KAP) on Ebola Virus Disease (EVD) Among Health Care Workers (HCWs) From the Lyon Teaching Hospitals, France. Open Forum Infectious Diseases. 2016;3(suppl_1):602. doi:10.1093/ofid/ofw172.465

Raj D. What Are the Appropriate Personal Protective Equipment (PPE) for Front line Workers (FLW) Caring for Filovirus/Ebola Virus Disease (EVD) Patients?

Senga M, Pringle K, Ramsay A, et al. Factors Underlying Ebola Virus Infection Among Health Workers, Kenema, Sierra Leone, 2014–2015. Clin Infect Dis. 2016;63(4):454-459. doi:10.1093/cid/ciw327

Tartari E, Parascandalo AF, Borg M. Ensuring healthcare workers’ safety in the management of Ebola virus disease: a novel competency assessment checklist for proper PPE use. Antimicrob Resist Infect Control. 2015;4(S1):P6, 2047-2994-4-S1-P6. doi:10.1186/2047-2994-4-S1-P6

**Does not assess EVD/Marburg transmission**

Den Boon S, Vallenas C, Ferri M, Norris S. Incorporating health workers’ perspectives into a WHO guideline on personal protective equipment developed during and Ebola virus disease outbreak [version 2; referees: 2 approved]. Published online March 9, 2018.

Gozel MG, Dokmetas I, Oztop AY, Engin A, Elaldi N, Bakir M. Recommended precaution procedures protect healthcare workers from Crimean-Congo hemorrhagic fever virus. International Journal of Infectious Diseases. 2013;17(11):e1046-e1050. doi:10.1016/j.ijid.2013.05.005

Ijarotimi IT, Ilesanmi OS, Aderinwale A, Abiodun-Adewusi O. Knowledge of Lassa fever and use of infection prevention and control facilities among health care workers during Lassa fever outbreak in Ondo State, Nigeria.

MacIntyre CR, Chughtai AA, Seale H, Richards GA, Davidson PM. Uncertainty, risk analysis and change for Ebola personal protective equipment guidelines. International Journal of Nursing Studies. 2015;52(5):899-903. doi:10.1016/j.ijnurstu.2014.12.001

Mallow M, Gary L, Jeng T, et al. WASH activities at two Ebola treatment units in Sierra Leone. Ikegami T, ed. PLoS ONE. 2018;13(5):e0198235. doi:10.1371/journal.pone.0198235

Ortega R, Bhadelia N, Obanor O, et al. Putting On and Removing Personal Protective Equipment. N Engl J Med. 2015;372(12):e16. doi:10.1056/NEJMvcm1412105

Reddy SC, Valderrama AL, Kuhar DT. Improving the Use of Personal Protective Equipment: Applying Lessons Learned. Clinical Infectious Diseases. 2019;69(Supplement_3):S165-S170. doi:10.1093/cid/ciz619

Soeters HM, Koivogui L, de Beer L, et al. Infection prevention and control training and capacity building during the Ebola epidemic in Guinea. Andrei G, ed. PLoS ONE. 2018;13(2):e0193291. doi:10.1371/journal.pone.0193291

Sprecher AG, Caluwaerts A, Draper M, et al. Personal Protective Equipment for Filovirus Epidemics: A Call for Better Evidence. J Infect Dis. 2015;212(suppl 2):S98-S100. doi:10.1093/infdis/jiv153

**HCWs not performing screening or triage activities**

Andonian J, Kazi S, Therkorn J, et al. Effect of an Intervention Package and Teamwork Training to Prevent Healthcare Personnel Self-contamination During Personal Protective Equipment Doffing. Clinical Infectious Diseases. 2019;69(Supplement_3):S248-S255. doi:10.1093/cid/ciz618

Baller A, Padoveze MC, Mirindi P, et al. Ebola virus disease nosocomial infections in the Democratic Republic of the Congo: a descriptive study of cases during the 2018–2020 outbreak. International Journal of Infectious Diseases. 2022;115:126-133. doi:10.1016/j.ijid.2021.11.039

Baloh J, Reisinger HS, Dukes K, et al. Healthcare Workers’ Strategies for Doffing Personal Protective Equipment. Clinical Infectious Diseases. 2019;69(Supplement_3):S192-S198. doi:10.1093/cid/ciz613

Casalino E, Astocondor E, Sanchez JC, Díaz-Santana DE, del Aguila C, Carrillo JP. Personal protective equipment for the Ebola virus disease: A comparison of 2 training programs. American Journal of Infection Control. 2015;43(12):1281-1287. doi:10.1016/j.ajic.2015.07.007

Chughtai AA, Chen X, Macintyre CR. Risk of self-contamination during doffing of personal protective equipment. American Journal of Infection Control. 2018;46(12):1329-1334. doi:10.1016/j.ajic.2018.06.003

Coca A, DiLeo T, Kim JH, Roberge R, Shaffer R. Baseline Evaluation With a Sweating Thermal Manikin of Personal Protective Ensembles Recommended for Use in West Africa. Disaster med public health prep. 2015;9(5):536-542. doi:10.1017/dmp.2015.97

Dunn AC, Walker TA, Redd J, et al. Nosocomial transmission of Ebola virus disease on pediatric and maternity wards: Bombali and Tonkolili, Sierra Leone, 2014. American Journal of Infection Control. 2016;44(3):269-272. doi:10.1016/j.ajic.2015.09.016

Fischer WA, Weber DJ, Wohl DA. Personal Protective Equipment: Protecting Health Care Providers in an Ebola Outbreak. Clinical Therapeutics. 2015;37(11):2402-2410. doi:10.1016/j.clinthera.2015.07.007

Hall S, Poller B, Bailey C, et al. Use of ultraviolet-fluorescence-based simulation in evaluation of personal protective equipment worn for first assessment and care of a patient with suspected high-consequence infectious disease. Journal of Hospital Infection. 2018;99(2):218-228. doi:10.1016/j.jhin.2018.01.002

Kiiza P, Mullin SI, Teo K, et al. Establishing Healthcare Worker Performance and Safety in Providing Critical Care for Patients in a Simulated Ebola Treatment Unit: Non-Randomized Pilot Study. Viruses. 2021;13(11):2205. doi:10.3390/v13112205

Kogutt BK, Sheffield JS, Garibaldi BT. 680: Assessing effectiveness of PPE in a simulated SVD of a highly infectious disease patient. American Journal of Obstetrics and Gynecology. 2019;220(1):S451. doi:10.1016/j.ajog.2018.11.703

Kwon JH, Burnham CAD, Reske KA, et al. Assessment of Healthcare Worker Protocol Deviations and Self-Contamination During Personal Protective Equipment Donning and Doffing. Infect Control Hosp Epidemiol. 2017;38(9):1077-1083. doi:10.1017/ice.2017.121

Robinson G, Kpadeh ZZ, Alserehi H, et al. Effect of glove disinfection on bacterial contamination of healthcare worker hands. Open Forum Infectious Diseases. Published online 2018.

Suen LKP, Guo YP, Tong DWK, et al. Self-contamination during doffing of personal protective equipment by healthcare workers to prevent Ebola transmission. Antimicrob Resist Infect Control. 2018;7(1):157. doi:10.1186/s13756-018-0433-y

**Narrative Review**

Franklin SM. A Comparison of Personal Protective Standards: Caring for Patients With Ebola Virus. *Clinical Nurse Specialist*. 2016;30(2):E1-E8. doi:[10.1097/NUR.0000000000000183](https://doi.org/10.1097/NUR.0000000000000183)

Honda H, Iwata K. Personal protective equipment and improving compliance among healthcare workers in high-risk settings: *Current Opinion in Infectious Diseases*. 2016;29(4):400-406. doi:[10.1097/QCO.0000000000000280](https://doi.org/10.1097/QCO.0000000000000280)

Kilinc FS. A Review of Isolation Gowns in Healthcare: Fabric and Gown Properties. *Journal of Engineered Fibers and Fabrics*. 2015;10(3):155892501501000. doi:[10.1177/155892501501000313](https://doi.org/10.1177/155892501501000313)

**Not Published in English**

Bosc J, Sanchez O, Carrie C, et al. Faisabilité des gestes d’urgence en tenue de protection individuelle du virus Ebola : pose d’abords vasculaires et contrôle des voies aériennes supérieures sur mannequin. Ann Fr Med Urgence. 2016;6(3):172-178. doi:10.1007/s13341-016-0631-2

Gertler M, Loik S, Kleine C, et al. Ebolafieberepidemie in Westafrika – schnelle und praxisnahe Ausbildung: Das Vorbereitungstraining für Einsatzkräfte des Deutschen Roten Kreuzes, anderer Hilfsorganisationen und der Bundeswehr, Würzburg, 2014 und 2015. Bundesgesundheitsbl. 2018;61(4):394-403. doi:10.1007/s00103-018-2710-6

Schmiedel S, Kreuels B. [Ebola virus disease in West Africa and Germany : clinical presentation, management and practical experience with medevacuated patients in Germany]. Bundesgesundheitsblatt Gesundheitsforschung Gesundheitsschutz. 2015;58(7):679-685. doi:10.1007/s00103-015-2162-1

**No Valid Intervention/comparator**

Abualenain JT, Al-Alawi MM. Simulation-based training in Ebola Personal Protective Equipment for healthcare workers: Experience from King Abdulaziz University Hospital in Saudi Arabia. Journal of Infection and Public Health. 2018;11(6):796-800. doi:10.1016/j.jiph.2018.05.002

Adler MD, Krug S, Eiger C, et al. Impact of Personal Protective Equipment on the Performance of Emergency Pediatric Tasks. Pediatr Emer Care. 2021;37(12):e1326-e1330. doi:10.1097/PEC.0000000000002028

Bell T, Smoot J, Patterson J, Smalligan R, Jordan R. Ebola virus disease: The use of fluorescents as markers of contamination for personal protective equipment. IDCases. 2015;2(1):27-30. doi:10.1016/j.idcr.2014.12.003

Biedron C, Lyman M, Stuckey MJ, et al. Evaluation of Infection Prevention and Control Readiness at Frontline Health Care Facilities in High-Risk Districts Bordering Ebola Virus Disease–Affected Areas in the Democratic Republic of the Congo — Uganda, 2018. MMWR Morb Mortal Wkly Rep. 2019;68(39):851-854. doi:10.15585/mmwr.mm6839a4

Casanova LM, Erukunuakpor K, Kraft CS, et al. Assessing Viral Transfer During Doffing of Ebola-Level Personal Protective Equipment in a Biocontainment Unit. Clinical Infectious Diseases. 2018;66(6):945-949. doi:10.1093/cid/cix956

Casanova LM, Teal LJ, Sickbert-Bennett EE, et al. Assessment of Self-Contamination During Removal of Personal Protective Equipment for Ebola Patient Care. Infect Control Hosp Epidemiol. 2016;37(10):1156-1161. doi:10.1017/ice.2016.169

Doshi RH, Hoff NA, Bratcher A, et al. Risk Factors for Ebola Exposure in Health Care Workers in Boende, Tshuapa Province, Democratic Republic of the Congo. The Journal of Infectious Diseases. 2022;226(4):608-615. doi:10.1093/infdis/jiaa747

Drew JL, Turner J, Mugele J, et al. Beating the Spread: Developing a Simulation Analog for Contagious Body Fluids. Simulation in Healthcare: The Journal of the Society for Simulation in Healthcare. 2016;11(2):100-105. doi:10.1097/SIH.0000000000000157

Drews FA, Mulvey D, Stratford K, Samore MH, Mayer J. Evaluation of a Redesigned Personal Protective Equipment Gown. Clinical Infectious Diseases. 2019;69(Supplement_3):S199-S205. doi:10.1093/cid/ciz520

DuBose JR, Matić Z, Sala MFW, et al. Design strategies to improve healthcare worker safety in biocontainment units: learning from ebola preparedness. Infect Control Hosp Epidemiol. 2018;39(8):961-967. doi:10.1017/ice.2018.125

Forrester JD, Hunter JC, Pillai SK, et al. Cluster of Ebola Cases Among Liberian and U.S. Health Care Workers in an Ebola Treatment Unit and Adjacent Hospital — Liberia, 2014. 2022;63(41):6.

Gao P, Horvatin M, Niezgoda G, Weible R, Shaffer R. Effect of multiple alcohol-based hand rub applications on the tensile properties of thirteen brands of medical exam nitrile and latex gloves. Journal of Occupational and Environmental Hygiene. 2016;13(12):905-914. doi:10.1080/15459624.2016.1191640

Garibaldi BT, Ruparelia C, Shaw-Saliba K, et al. A novel personal protective equipment coverall was rated higher than standard Ebola virus personal protective equipment in terms of comfort, mobility and perception of safety when tested by health care workers in Liberia and in a United States biocontainment unit. American Journal of Infection Control. 2019;47(3):298-304. doi:10.1016/j.ajic.2018.08.014

Gleason B, Redd J, Kilmarx P, et al. Establishment of an Ebola Treatment Unit and Laboratory — Bombali District, Sierra Leone, July 2014–January 2015. MMWR Morb Mortal Wkly Rep. 2015;64(39):1108-1111. doi:10.15585/mmwr.mm6439a4

Grélot L, Koulibaly F, Maugey N, et al. Moderate Thermal Strain in Healthcare Workers Wearing Personal Protective Equipment During Treatment and Care Activities in the Context of the 2014 Ebola Virus Disease Outbreak. J Infect Dis. 2016;213(9):1462-1465. doi:10.1093/infdis/jiv585

Grinnell M, Dixon MG, Patton M, et al. Ebola Virus Disease in Health Care Workers — Guinea, 2014. MMWR Morb Mortal Wkly Rep. 2015;64(38):1083-1087. doi:10.15585/mmwr.mm6438a6

Gurses AP, Dietz AS, Nowakowski E, et al. Human factors–based risk analysis to improve the safety of doffing enhanced personal protective equipment. Infect Control Hosp Epidemiol. 2019;40(2):178-186. doi:10.1017/ice.2018.292

Jaques PA, Gao P, Kilinc-Balci S, et al. Evaluation of gowns and coveralls used by medical personnel working with Ebola patients against simulated bodily fluids using an Elbow Lean Test. Journal of Occupational and Environmental Hygiene. 2016;13(11):881-893. doi:10.1080/15459624.2016.1186279

Jinadatha C, Simmons S, Dale C, et al. Disinfecting personal protective equipment with pulsed xenon ultraviolet as a risk mitigation strategy for health care workers. American Journal of Infection Control. 2015;43(4):412-414. doi:10.1016/j.ajic.2015.01.013

Kilmarx PH, Clarke KR, Dietz PM, et al. Ebola Virus Disease in Health Care Workers — Sierra Leone, 2014. 2014;63(49):4.

Lim SM, Cha WC, Chae MK, Jo IJ. Contamination during doffing of personal protective equipment by healthcare providers. Clin Exp Emerg Med. 2015;2(3):162-167. doi:10.15441/ceem.15.019

Matanock A, Arwady MA, Ayscue P, et al. Ebola Virus Disease Cases Among Health Care Workers Not Working in Ebola Treatment Units — Liberia, June–August, 2014. 2014;63(46):5.

Mumma JM, Durso FT, Casanova LM, et al. Common Behaviors and Faults When Doffing Personal Protective Equipment for Patients With Serious Communicable Diseases. Clinical Infectious Diseases. 2019;69(Supplement_3):S214-S220. doi:10.1093/cid/ciz614

Mumma JM, Durso FT, Ferguson AN, et al. Human Factors Risk Analyses of a Doffing Protocol for Ebola-Level Personal Protective Equipment: Mapping Errors to Contamination. Clinical Infectious Diseases. 2018;66(6):950-958. doi:10.1093/cid/cix957

Olu O, Kargbo B, Kamara S, et al. Epidemiology of Ebola virus disease transmission among health care workers in Sierra Leone, May to December 2014: a retrospective descriptive study. BMC Infect Dis. 2015;15(1):416. doi:10.1186/s12879-015-1166-7

Pathmanathan I, O’Connor KA, Adams ML, et al. Rapid Assessment of Ebola Infection Prevention and Control Needs — Six Districts, Sierra Leone, October 2014. 2022;63(49):4.

Poller B, Hall S, Bailey C, et al. ‘VIOLET’: a fluorescence-based simulation exercise for training healthcare workers in the use of personal protective equipment. Journal of Hospital Infection. 2018;99(2):229-235. doi:10.1016/j.jhin.2018.01.021

Poller B, Tunbridge A, Hall S, et al. A unified personal protective equipment ensemble for clinical response to possible high consequence infectious diseases: A consensus document on behalf of the HCID programme. Journal of Infection. 2018;77(6):496-502. doi:10.1016/j.jinf.2018.08.016

Raj D, Hornsey E, Perl TM. Personal protective equipment for viral hemorrhagic fevers: Current Opinion in Infectious Diseases. 2019;32(4):337-347. doi:10.1097/QCO.0000000000000562

Ratnayake R, Ho LS, Ansumana R, et al. Improving Ebola infection prevention and control in primary healthcare facilities in Sierra Leone: a single-group pretest post-test, mixed-methods study. BMJ Glob Health. 2016;1(4):e000103. doi:10.1136/bmjgh-2016-000103

Reidy P, Fletcher T, Shieber C, et al. Personal protective equipment solution for UK military medical personnel working in an Ebola virus disease treatment unit in Sierra Leone. Journal of Hospital Infection. 2017;96(1):42-48. doi:10.1016/j.jhin.2017.03.018

Selvaraj SA, Lee KE, Harrell M, Ivanov I, Allegranzi B. Infection Rates and Risk Factors for Infection Among Health Workers During Ebola and Marburg Virus Outbreaks: A Systematic Review. The Journal of Infectious Diseases. 2018;218(suppl_5):S679-S689. doi:10.1093/infdis/jiy435

Wong MF, Matić Z, Campiglia GC, et al. Design Strategies for Biocontainment Units to Reduce Risk During Doffing of High-level Personal Protective Equipment. Clinical Infectious Diseases. 2019;69(Supplement_3):S241-S247. doi:10.1093/cid/ciz617

Zellmer C, Van Hoof S, Safdar N. Variation in health care worker removal of personal protective equipment. American Journal of Infection Control. 2015;43(7):750-751. doi:10.1016/j.ajic.2015.02.005

**Full text Unavailable**

Chu M, Bausch D, Velazquez-Berumen A, Vallenas C, A. Committees. Report from the world health organization’s advisory committees on innovative personal protective equipment for front line health workers. 2017;American Journal of Tropical Medicine and Hygiene.

Drew J, Turner J, Cooper D, Zaiser R, Duncan T, Mugele J. Novel use of ultraviolet tracer contagion in multiple-patient simulation and the effect of personal protective equipment on contagion spread: A feasibility study. Academic Emergency Medicine. Published online 2015.

Garibaldi BT, Rainwater-Lovett K, Pilholski T, et al. Transmission of fluorescent aerosolized particles in a clinical biocontainment unit. American Journal of Respiratory and Critical Care Medicine Conference: American Thoracic Society International Conference ATS. Published online 2017.

Ho L, Ratnayake R, Brown H, et al. Precious. Lifesaving but not without problems; a mixed-methods study examining barriers and facilitators to infection prevention and control in health facilities during the ebola virus disease epidemic in Sierra Leone. Published online 2015.

Kabore RP, Kabore DSR, Zida S, Ouedraogo NW, Verbeek L, Gies S. Improving basic hygiene among health care workers through Ebola-training: A field perspective. Tropical Medicine and International Health. Published online 2017.

Kratz T, Verbeek L. Discussion of two infection prevention and control training approaches to enhance biosafety in primary healthcare facilities during an outbreak of Ebola virus disease. Tropical Medicine and International Health. Published online 2017.

Lee MH, Meerbach A, Straub J, et al. Which personal protective equipment to provide?-Challenges during the Ebola outbreak and lessons learned. Tropical Medicine and International Health. Published online 2017.

Nelson AR, Fiedler A, Zikeh T, Moses N, Ruparelia C, Oseni L. Institutionalizing infection prevention and control practices in health facilities in liberia following the Ebola epidemic. American Journal of Tropical Medicine and Hygiene. Published online 2018.

Senga M, Pringle K, Brett-Major D, et al. Largest documented cluster of ebola virus disease among health workers. Published online 2015.

Somers Y, Verbiest M. Suspecting ebola: When the dress code becomes life saving! Personal protective equipment-a practical demonstration. Anaesthesiology Intensive Therapy. Published online 2014.

**Systematic Review (Assessed included studies in SR)**

Hersi M, Stevens A, Quach P, et al. Effectiveness of Personal Protective Equipment for Healthcare Workers Caring for Patients with Filovirus Disease: A Rapid Review. Kuhn JH, ed. *PLoS ONE*. 2015;10(10):e0140290. doi:[10.1371/journal.pone.0140290](https://doi.org/10.1371/journal.pone.0140290)

Verbeek JH, Ijaz S, Mischke C, et al. Personal protective equipment for preventing highly infectious diseases due to exposure to contaminated body fluids in healthcare staff. Cochrane Work Group, ed. *Cochrane Database of Systematic Reviews*. Published online April 19, 2016. doi:[10.1002/14651858.CD011621.pub2](https://doi.org/10.1002/14651858.CD011621.pub2)

Hopman J, Kubilay Z, Allen T, Edrees H, Pittet D, Allegranzi B. Efficacy of chlorine solutions used for hand hygiene and gloves disinfection in Ebola settings: a systematic review. *Antimicrob Resist Infect Control*. 2015;4(S1):O13. doi:[10.1186/2047-2994-4-S1-O13](https://doi.org/10.1186/2047-2994-4-S1-O13)

# Theme 3: Disinfection/Decontamination

# KQ9

**Does not examine Ebola or Marburg (or surrogate viruses)**

Amadin, Jacob. Comparison of the Effectiveness of Disinfectant-Impregnated Wipes Versus Detergent Wipes for Surface Decontamination. University of South Florida; 2021.

**Intervention not of interest**

Calfee MW, Ryan SP, Abdel‐Hady A, et al. Virucidal efficacy of antimicrobial surface coatings against the enveloped bacteriophage Φ6. *J of Applied Microbiology*. 2022;132(3):1813-1824. doi:[10.1111/jam.15339](https://doi.org/10.1111/jam.15339)

Cutts TA, Robertson C, Theriault SS, et al. Assessing the Contributions of Inactivation, Removal, and Transfer of Ebola Virus and Vesicular Stomatitis Virus by Disinfectant Pre-soaked Wipes. *Front Public Health*. 2020;8:183. doi:[10.3389/fpubh.2020.00183](https://doi.org/10.3389/fpubh.2020.00183)

Tomas ME, Cadnum JL, Jencson A, Donskey CJ. The Ebola Disinfection Booth: Evaluation of an Enclosed Ultraviolet Light Booth for Disinfection of Contaminated Personal Protective Equipment Prior to Removal. *Infect Control Hosp Epidemiol*. 2015;36(10):1226-1228. doi:[10.1017/ice.2015.166](https://doi.org/10.1017/ice.2015.166)

Jinadatha C, Simmons S, Dale C, et al. Disinfecting personal protective equipment with pulsed xenon ultraviolet as a risk mitigation strategy for health care workers. *American Journal of Infection Control*. 2015;43(4):412-414. doi:[10.1016/j.ajic.2015.01.013](https://doi.org/10.1016/j.ajic.2015.01.013)

Cutts TA, Ijaz MK, Nims RW, Rubino JR, Theriault SS. Effectiveness of Dettol Antiseptic Liquid for Inactivation of Ebola Virus in Suspension. *Sci Rep*. 2019;9(1):6590. doi:[10.1038/s41598-019-42386-5](https://doi.org/10.1038/s41598-019-42386-5)

Pearlmutter BS, Haq MF, Cadnum JL, Jencson AL, Carlisle M, Donskey CJ. Efficacy of relatively low-cost ultraviolet-C light devices against *Candida auris*. *Infect Control Hosp Epidemiol*. 2022;43(6):747-751. doi:[10.1017/ice.2021.206](https://doi.org/10.1017/ice.2021.206)

Sagripanti JL, Lytle CD. Sensitivity to ultraviolet radiation of Lassa, vaccinia, and Ebola viruses dried on surfaces. *Arch Virol*. 2011;156(3):489-494. doi:[10.1007/s00705-010-0847-1](https://doi.org/10.1007/s00705-010-0847-1)

Lowe JJ, Hewlett AL, Iwen PC, Smith PW, Gibbs SG. Surrogate Testing Suggests That Chlorine Dioxide Gas Exposure Would Not Inactivate Ebola Virus Contained in Environmental Blood Contamination. *Journal of Occupational and Environmental Hygiene*. 2015;12(9):D211-D215. doi:[10.1080/15459624.2015.1043058](https://doi.org/10.1080/15459624.2015.1043058)

Otter JA, Mepham S, Athan B, et al. Terminal decontamination of the Royal Free London’s high-level isolation unit after a case of Ebola virus disease using hydrogen peroxide vapor. *American Journal of Infection Control*. 2016;44(2):233-235. doi:[10.1016/j.ajic.2015.08.025](https://doi.org/10.1016/j.ajic.2015.08.025)

**No relevant comparisons**

Casey ML, Nguyen DT, Idriss B, Bennett S, Dunn A, Martin S. Potential Exposure to Ebola Virus from Body Fluids due to Ambulance Compartment Permeability in Sierra Leone. *Prehosp Disaster med*. 2015;30(6):625-627. doi:[10.1017/S1049023X15005294](https://doi.org/10.1017/S1049023X15005294)

Cook B, Cutts T, Nikiforuk A, et al. Evaluating Environmental Persistence and Disinfection of the Ebola Virus Makona Variant. *Viruses*. 2015;7(4):1975-1986. doi:[10.3390/v7041975](https://doi.org/10.3390/v7041975)

Cook BWM, Cutts TA, Nikiforuk AM, Leung A, Kobasa D, Theriault SS. The Disinfection Characteristics of Ebola Virus Outbreak Variants. *Sci Rep*. 2016;6(1):38293. doi:[10.1038/srep38293](https://doi.org/10.1038/srep38293)

Cutts TA, Robertson C, Theriault SS, et al. Efficacy of microbicides for inactivation of Ebola–Makona virus on a non-porous surface: a targeted hygiene intervention for reducing virus spread. *Sci Rep*. 2020;10(1):15247. doi:[10.1038/s41598-020-71736-x](https://doi.org/10.1038/s41598-020-71736-x)

Gallandat K, Lantagne D. Selection of a Biosafety Level 1 (BSL-1) surrogate to evaluate surface disinfection efficacy in Ebola outbreaks: Comparison of four bacteriophages. Galdiero M, ed. *PLoS ONE*. 2017;12(5):e0177943. doi:[10.1371/journal.pone.0177943](https://doi.org/10.1371/journal.pone.0177943)

Gallandat K, Wolfe MK, Lantagne D. Surface Cleaning and Disinfection: Efficacy Assessment of Four Chlorine Types Using Escherichia coli and the Ebola Surrogate Phi6. *Environ Sci Technol*. Published online 2017:8.

Ijaz MK. Comparison of the Efficacy of Disinfectant Pre-impregnated Wipes for Decontaminating Stainless Steel Carriers Experimentally Inoculated With Ebola Virus and Vesicular Stomatitis Virus. *Frontiers in Public Health*. 2021;9:9.

Julian TR, Trumble JM, Schwab KJ. Evaluating Efficacy of Field-Generated Electrochemical Oxidants on Disinfection of Fomites Using Bacteriophage MS2 and Mouse Norovirus MNV-1 as Pathogenic Virus Surrogates. *Food Environ Virol*. 2014;6(2):145-155. doi:[10.1007/s12560-014-9136-6](https://doi.org/10.1007/s12560-014-9136-6)

Park GW, Sobsey MD. Simultaneous Comparison of Murine Norovirus, Feline Calicivirus, Coliphage MS2, and GII.4 Norovirus to Evaluate the Efficacy of Sodium Hypochlorite Against Human Norovirus on a Fecally Soiled Stainless Steel Surface. *Foodborne Pathogens and Disease*. 2011;8(9):1005-1010. doi:[10.1089/fpd.2010.0782](https://doi.org/10.1089/fpd.2010.0782)

Poliquin PG, Vogt F, Kasztura M, et al. Environmental Contamination and Persistence of Ebola Virus RNA in an Ebola Treatment Center. *J Infect Dis*. 2016;214(suppl 3):S145-S152. doi:[10.1093/infdis/jiw198](https://doi.org/10.1093/infdis/jiw198)

String GM, Kamal Y, Gute DM, Lantagne DS. Chlorine efficacy against bacteriophage Phi6, a surrogate for enveloped human viruses, on porous and non-porous surfaces at varying temperatures and humidity. *Journal of Environmental Science and Health, Part A*. 2022;57(8):685-693. doi:[10.1080/10934529.2022.2101845](https://doi.org/10.1080/10934529.2022.2101845)

Smither S, Phelps A, Eastaugh L, et al. Effectiveness of Four Disinfectants against Ebola Virus on Different Materials. *Viruses*. 2016;8(7):185. doi:[10.3390/v8070185](https://doi.org/10.3390/v8070185)

Smither SJ, Eastaugh L, Filone CM, et al. Two-Center Evaluation of Disinfectant Efficacy against Ebola Virus in Clinical and Laboratory Matrices. *Emerg Infect Dis*. 2018;24(1). doi:[10.3201/eid2401.170504](https://doi.org/10.3201/eid2401.170504)

Wood JP, Richter W, Sunderman M, Calfee MW, Serre S, Mickelsen L. Evaluating the Environmental Persistence and Inactivation of MS2 Bacteriophage and the Presumed Ebola Virus Surrogate Phi6 Using Low Concentration Hydrogen Peroxide Vapor. *Environ Sci Technol*. 2020;54(6):3581-3590. doi:[10.1021/acs.est.9b06034](https://doi.org/10.1021/acs.est.9b06034)

Wells E, Wolfe MK, Murray A, Lantagne D. Accuracy, Precision, Ease-Of-Use, and Cost of Methods to Test Ebola-Relevant Chlorine Solutions. Munster VJ, ed. *PLoS ONE*. 2016;11(5):e0152442. doi:[10.1371/journal.pone.0152442](https://doi.org/10.1371/journal.pone.0152442)

Youkee D, Brown CS, Lilburn P, et al. Assessment of Environmental Contamination and Environmental Decontamination Practices within an Ebola Holding Unit, Freetown, Sierra Leone. Kuhn JH, ed. *PLoS ONE*. 2015;10(12):e0145167. doi:[10.1371/journal.pone.0145167](https://doi.org/10.1371/journal.pone.0145167)

Johnston AMcD, Lewis SE. Decontaminating Ebola-infected ultrasound probes. *Anaesthesia*. 2015;70(5):628-629. doi:[10.1111/anae.13060](https://doi.org/10.1111/anae.13060)

Gaillard T, Delaune D, Flusin O, et al. Decontamination of a field laboratory dedicated to Ebola virus–infected patients. *American Journal of Infection Control*. 2016;44(12):1687-1688. doi:[10.1016/j.ajic.2016.05.034](https://doi.org/10.1016/j.ajic.2016.05.034)

Lowe JJ, Olinger PL, Gibbs SG, et al. Environmental infection control considerations for Ebola. *American Journal of Infection Control*. 2015;43(7):747-749. doi:[10.1016/j.ajic.2015.03.006](https://doi.org/10.1016/j.ajic.2015.03.006)

Kang J, Tyan KS, Jin K, Kyle AM. Field testing of a novel colour indicator added to chlorine solutions used for decontamination of surfaces in Ebola treatment units. *Journal of Hospital Infection*. 2018;99(2):188-191. doi:[10.1016/j.jhin.2017.11.004](https://doi.org/10.1016/j.jhin.2017.11.004)

Doona CJ, Feeherry FE, Kustin K, et al. Fighting Ebola with novel spore decontamination technologies for the military. *Front Microbiol*. 2015;6. doi:[10.3389/fmicb.2015.00663](https://doi.org/10.3389/fmicb.2015.00663)

Jelden KC, Gibbs SG, Smith PW, et al. Nebraska Biocontainment Unit patient discharge and environmental decontamination after Ebola care. *American Journal of Infection Control*. 2015;43(3):203-205. doi:[10.1016/j.ajic.2014.12.005](https://doi.org/10.1016/j.ajic.2014.12.005)

Mollura DJ, Palmore TN, Folio LR, Bluemke DA. Radiology Preparedness in Ebola Virus Disease: Guidelines and Challenges for Disinfection of Medical Imaging Equipment for the Protection of Staff and Patients. *Radiology*. 2015;275(2):538-544. doi:[10.1148/radiol.15142670](https://doi.org/10.1148/radiol.15142670)

**Narrative review (Study design not of interest)**

Lantagne D, Wolfe M, Gallandat K, Opryszko M. Determining the Efficacy, Safety and Suitability of Disinfectants to Prevent Emerging Infectious Disease Transmission. *Water*. 2018;10(10):1397. doi:[10.3390/w10101397](https://doi.org/10.3390/w10101397)

Boyce JM. Alcohols as Surface Disinfectants in Healthcare Settings. *Infect Control Hosp Epidemiol*. 2018;39(3):323-328. doi:[10.1017/ice.2017.301](https://doi.org/10.1017/ice.2017.301)

**Full text Unavailable**

Khou-Bouvattier C, Rogee S. [Effect of virucidal components on highly pathogenic viral agents]. TITLE: Actions des composÃ©s virucides sur les agents viraux hautement pathogÃ¨nes. *Virologie*.

Sharp PE, Ferguson L, Faseeh I, et al. High-intensity ultraviolet light as an adjunct to conventional cleaning methods in a laboratory animal facility. *Journal of the American Association for Laboratory Animal Science*. Published online 2017.

Commentary

Walker N, Youkee D, Brown C, Lado M, Johnson O. Management of Ebola virus disease: is environmental decontamination effective? *J Infect Dis*. Published online December 23, 2016:jiw548. doi:[10.1093/infdis/jiw548](https://doi.org/10.1093/infdis/jiw548)

# KQ10

**Does not examine Ebola or Marburg**

Martin D, Balermpas P, Gollrad J, et al. RADIANCE – Radiochemotherapy with or without Durvalumab in the treatment of anal squamous cell carcinoma: A randomized multicenter phase II trial. Clinical and Translational Radiation Oncology. 2020;23:43-49. doi:10.1016/j.ctro.2020.04.010

Ukwenya VO, Fuwape TA, Fadahunsi TI, Ilesanmi OS. Disparities in knowledge, attitude, and practices of infection prevention and control of lassa fever among health care workers at The Federal Medical Centre, Owo, Ondo State, Nigeria. *Pan Afr Med J*. 2021;38. doi:[10.11604/pamj.2021.38.357.26208](https://doi.org/10.11604/pamj.2021.38.357.26208)

Obionu IM, Ochu CL, Ukponu W, et al. Evaluation of infection prevention and control practices in Lassa fever treatment centers in north-central Nigeria during an ongoing Lassa fever outbreak. *Journal of Infection Prevention*. 2021;22(6):275-282. doi:[10.1177/17571774211035838](https://doi.org/10.1177/17571774211035838)

Ilesanmi OS, Ayodeji OO, Bakare AA, et al. Status of Infection prevention and control (IPC) at a Lassa fever treatment center before and after the implementation of an intensive IPC program. *jidhealth*. 2020;3(3):213-216. doi:[10.47108/jidhealth.Vol3.Iss3.66](https://doi.org/10.47108/jidhealth.Vol3.Iss3.66)

**Full text Unavailable**

Drew J, Turner J, Cooper D, Zaiser R, Duncan T, Mugele J. Novel use of ultraviolet tracer contagion in multiple-patient simulation and the effect of personal protective equipment on contagion spread: A feasibility study. Academic Emergency Medicine. Published online 2015.

Garibaldi BT, Rainwater-Lovett K, Pilholski T, et al. Transmission of fluorescent aerosolized particles in a clinical biocontainment unit. American Journal of Respiratory and Critical Care Medicine Conference: American Thoracic Society International Conference ATS. Published online 2017.

Somers Y, Verbiest M. Suspecting ebola: When the dress code becomes life saving! Personal protective equipment-a practical demonstration. Anaesthesiology Intensive Therapy. Published online 2014.

Kratz T, Verbeek L. Discussion of two infection prevention and control training approaches to enhance biosafety in primary healthcare facilities during an outbreak of Ebola virus disease. *Tropical Medicine and International Health*. Published online 2017.

Nelson AR, Fiedler A, Zikeh T, Moses N, Ruparelia C, Oseni L. Institutionalizing infection prevention and control practices in health facilities in liberia following the Ebola epidemic. *American Journal of Tropical Medicine and Hygiene*. Published online 2018.

Ho L, Ratnayake R, Brown H, et al. Precious. Lifesaving but not without problems; a mixed-methods study examining barriers and facilitators to infection prevention and control in health facilities during the ebola virus disease epidemic in Sierra Leone. Published online 2015.

Theodora AA, Aissi K, Affovehounde L, et al. Setting up of a national action plan for prevention and control of healthcare-associated infections in Benin. *Antimicrobial Resistance and Infection Control Conference: International Conference on Prevention and Infection Control ICPIC*. Published online 2017.

Raj D. What are the appropriate personal protective equipment (PPE) for frontline workers (FLW) caring for filovirus/ebola virus disease (EVD) patients? *Open Forum Infectious Diseases*. Published online 2017.

**Narrative review**

Fischer WA, Weber DJ, Wohl DA. Personal Protective Equipment: Protecting Health Care Providers in an Ebola Outbreak. Clinical Therapeutics. 2015;37(11):2402-2410. doi:10.1016/j.clinthera.2015.07.007

Hageman JC, Hazim C, Wilson K, et al. Infection Prevention and Control for Ebola in Health Care Settings — West Africa and United States. *MMWR Suppl*. 2016;65(3):50-56. doi:[10.15585/mmwr.su6503a8](https://doi.org/10.15585/mmwr.su6503a8)

Honda H, Iwata K. Personal protective equipment and improving compliance among healthcare workers in high-risk settings: *Current Opinion in Infectious Diseases*. 2016;29(4):400-406. doi:[10.1097/QCO.0000000000000280](https://doi.org/10.1097/QCO.0000000000000280)

Raj D, Hornsey E, Perl TM. Personal protective equipment for viral hemorrhagic fevers: *Current Opinion in Infectious Diseases*. 2019;32(4):337-347. doi:[10.1097/QCO.0000000000000562](https://doi.org/10.1097/QCO.0000000000000562)

**Non comparative study**

Casanova LM, Erukunuakpor K, Kraft CS, et al. Assessing Viral Transfer During Doffing of Ebola-Level Personal Protective Equipment in a Biocontainment Unit. Clinical Infectious Diseases. 2018;66(6):945-949. doi:10.1093/cid/cix956

Ortega R, Bhadelia N, Obanor O, et al. Putting On and Removing Personal Protective Equipment. N Engl J Med. 2015;372(25):2464-2465. doi:10.1056/NEJMc1504851

Lee M a, Huh K, Jeong J, et al. Adherence to Protocols by Healthcare Workers and Self-Contamination During Doffing of Personal Protective Equipment. American Journal of Infection Control. 2018;46(6):S11. doi:10.1016/j.ajic.2018.04.024

Lim SM, Cha WC, Chae MK, Jo IJ. Contamination during doffing of personal protective equipment by healthcare providers. Clin Exp Emerg Med. 2015;2(3):162-167. doi:10.15441/ceem.15.019

Russo N, Archer M, Kinzie L, Pfeiffer CD. Beyond Ebola: Standardizing the Approach to High Consequence Infection Preparation. American Journal of Infection Control. 2018;46(6):S110-S111. doi:10.1016/j.ajic.2018.04.196

Levy B, Rao CY, Miller L, et al. Ebola infection control in Sierra Leonean health clinics: A large cross-agency cooperative project. *American Journal of Infection Control*. 2015;43(7):752-755. doi:[10.1016/j.ajic.2015.03.011](https://doi.org/10.1016/j.ajic.2015.03.011)

Matanock A, Arwady MA, Ayscue P, et al. Ebola Virus Disease Cases Among Health Care Workers Not Working in Ebola Treatment Units — Liberia, June–August, 2014. 2014;63(46).

Weah VD, Doedeh JS, Wiah SQ, Nyema E, Lombeh S, Naiene J. Enhancing Ebola Virus Disease Surveillance and Prevention in Counties Without Confirmed Cases in Rural Liberia: Experiences from Sinoe County During the Flare-up in Monrovia, April to June, 2016. *American Journal of Obstetrics and Gynecology*.

Ndede PO, Senkungu JK, Shakpeh JK, Jones TE, Sky R, McDonnell S. Health Services and Infrastructure Recovery of a Major Public Hospital in Liberia During the 2014–2016 Ebola Epidemic. *Disaster med public health prep*. 2019;13(4):767-773. doi:[10.1017/dmp.2018.124](https://doi.org/10.1017/dmp.2018.124)

Pathmanathan I, O’Connor KA, Adams ML, et al. Rapid Assessment of Ebola Infection Prevention and Control Needs — Six Districts, Sierra Leone, October 2014. 2014;63(49).

**No outcome data**

McLaws ML, Chughtai AA, Salmon S, MacIntyre CR. A highly precautionary doffing sequence for health care workers after caring for wet Ebola patients to further reduce occupational acquisition of Ebola. American Journal of Infection Control. 2016;44(7):740-744. doi:10.1016/j.ajic.2015.12.034

**Systematic review**

Verbeek JH, Rajamaki B, Ijaz S, et al. Personal protective equipment for preventing highly infectious diseases due to exposure to contaminated body fluids in healthcare staff. Cochrane Work Group, ed. Cochrane Database of Systematic Reviews. Published online April 15, 2020. doi:10.1002/14651858.CD011621.pub4

**Wrong intervention/comparator (Does not compare spraying vs. not spraying for HCWs)**

Andonian J, Kazi S, Therkorn J, et al. Effect of an Intervention Package and Teamwork Training to Prevent Healthcare Personnel Self-contamination During Personal Protective Equipment Doffing. Clinical Infectious Diseases. 2019;69(Supplement_3):S248-S255. doi:10.1093/cid/ciz618

Bell T, Smoot J, Patterson J, Smalligan R, Jordan R. Ebola virus disease: The use of fluorescents as markers of contamination for personal protective equipment. IDCases. 2015;2(1):27-30. doi:10.1016/j.idcr.2014.12.003

Berry L, Button T, Fonnie C, King M. How to set up an Ebola isolation unit: Lessons learned from Rokupa. Journal of Clinical Virology. 2015;70:S17. doi:10.1016/j.jcv.2015.07.046

Chughtai AA, Chen X, Macintyre CR. Risk of self-contamination during doffing of personal protective equipment. American Journal of Infection Control. 2018;46(12):1329-1334. doi:10.1016/j.ajic.2018.06.003

Cummings KJ, Choi MJ, Esswein EJ, et al. Addressing Infection Prevention and Control in the First U.S. Community Hospital to Care for Patients With Ebola Virus Disease: Context for National Recommendations and Future Strategies. Ann Intern Med. 2016;165(1):41. doi:10.7326/M15-2944

Drew JL, Turner J, Mugele J, et al. Beating the Spread: Developing a Simulation Analog for Contagious Body Fluids. Simulation in Healthcare: The Journal of the Society for Simulation in Healthcare. 2016;11(2):100-105. doi:10.1097/SIH.0000000000000157

DuBose JR, Matić Z, Sala MFW, et al. Design strategies to improve healthcare worker safety in biocontainment units: learning from ebola preparedness. Infect Control Hosp Epidemiol. 2018;39(8):961-967. doi:10.1017/ice.2018.125

Kwon JH, Burnham CAD, Reske K, et al. Healthcare Worker Self-Contamination During Standard and Ebola Virus Disease Personal Protective Equipment Doffing. Open Forum Infectious Diseases. 2016;3(suppl_1):1387. doi:10.1093/ofid/ofw172.1090

Kwon JH, Burnham CAD, Reske KA, et al. Assessment of Healthcare Worker Protocol Deviations and Self-Contamination During Personal Protective Equipment Donning and Doffing. Infect Control Hosp Epidemiol. 2017;38(9):1077-1083. doi:10.1017/ice.2017.121

Mehtar S, Bulabula ANH, Nyandemoh H, Jambawai S. Deliberate exposure of humans to chlorine-the aftermath of Ebola in West Africa. Antimicrob Resist Infect Control. 2016;5(1):45. doi:10.1186/s13756-016-0144-1

Mumma JM, Durso FT, Casanova LM, et al. Common Behaviors and Faults When Doffing Personal Protective Equipment for Patients With Serious Communicable Diseases. Clinical Infectious Diseases. 2019;69(Supplement_3):S214-S220. doi:10.1093/cid/ciz614

Mumma JM, Durso FT, Ferguson AN, et al. Human Factors Risk Analyses of a Doffing Protocol for Ebola-Level Personal Protective Equipment: Mapping Errors to Contamination. Clinical Infectious Diseases. 2018;66(6):950-958. doi:10.1093/cid/cix957

Poller B, Hall S, Bailey C, et al. ‘VIOLET’: a fluorescence-based simulation exercise for training healthcare workers in the use of personal protective equipment. Journal of Hospital Infection. 2018;99(2):229-235. doi:10.1016/j.jhin.2018.01.021

Poller B, Tunbridge A, Hall S, et al. A unified personal protective equipment ensemble for clinical response to possible high consequence infectious diseases: A consensus document on behalf of the HCID programme. Journal of Infection. 2018;77(6):496-502. doi:10.1016/j.jinf.2018.08.016

Reidy P, Fletcher T, Shieber C, et al. Personal protective equipment solution for UK military medical personnel working in an Ebola virus disease treatment unit in Sierra Leone. Journal of Hospital Infection. 2017;96(1):42-48. doi:10.1016/j.jhin.2017.03.018

Suen LKP, Guo YP, Tong DWK, et al. Self-contamination during doffing of personal protective equipment by healthcare workers to prevent Ebola transmission. Antimicrob Resist Infect Control. 2018;7(1):157. doi:10.1186/s13756-018-0433-y

Tartari E, Parascandalo AF, Borg M. Ensuring healthcare workers’ safety in the management of Ebola virus disease: a novel competency assessment checklist for proper PPE use. Antimicrob Resist Infect Control. 2015;4(S1):P6, 2047-2994-4-S1-P6. doi:10.1186/2047-2994-4-S1-P6

Zellmer C, Van Hoof S, Safdar N. Variation in health care worker removal of personal protective equipment. American Journal of Infection Control. 2015;43(7):750-751. doi:10.1016/j.ajic.2015.02.005

Ho LS yi, Ratnayake R, Ansumana R, Brown H. A mixed-methods investigation to understand and improve the scaled-up infection prevention and control in primary care health facilities during the Ebola virus disease epidemic in Sierra Leone. *BMC Public Health*. 2021;21(1):1603. doi:[10.1186/s12889-021-11634-7](https://doi.org/10.1186/s12889-021-11634-7)

Gao P, Horvatin M, Niezgoda G, Weible R, Shaffer R. Effect of multiple alcohol-based hand rub applications on the tensile properties of thirteen brands of medical exam nitrile and latex gloves. *Journal of Occupational and Environmental Hygiene*. 2016;13(12):905-914. doi:[10.1080/15459624.2016.1191640](https://doi.org/10.1080/15459624.2016.1191640)

Olu O, Kargbo B, Kamara S, et al. Epidemiology of Ebola virus disease transmission among health care workers in Sierra Leone, May to December 2014: a retrospective descriptive study. *BMC Infect Dis*. 2015;15(1):416. doi:[10.1186/s12879-015-1166-7](https://doi.org/10.1186/s12879-015-1166-7)

Drews FA, Mulvey D, Stratford K, Samore MH, Mayer J. Evaluation of a Redesigned Personal Protective Equipment Gown. *Clinical Infectious Diseases*. 2019;69(Supplement_3):S199-S205. doi:[10.1093/cid/ciz520](https://doi.org/10.1093/cid/ciz520)

Oji MO, Haile M, Baller A, et al. Implementing infection prevention and control capacity building strategies within the context of Ebola outbreak in a “Hard-to-Reach” area of Liberia. *Pan Afr Med J*. 2018;31. doi:[10.11604/pamj.2018.31.107.15517](https://doi.org/10.11604/pamj.2018.31.107.15517)

Ratnayake R, Ho LS, Ansumana R, et al. Improving Ebola infection prevention and control in primary healthcare facilities in Sierra Leone: a single-group pretest post-test, mixed-methods study. *BMJ Glob Health*. 2016;1(4):e000103. doi:[10.1136/bmjgh-2016-000103](https://doi.org/10.1136/bmjgh-2016-000103)

Ousman K, Kabego L, Talisuna A, et al. The impact of **Infection Prevention and control (IPC) bundle implementation** on IPC compliance during the Ebola virus outbreak in Mbandaka/Democratic Republic of the Congo: a before and after design. *BMJ Open*. 2019;9(9):e029717. doi:[10.1136/bmjopen-2019-029717](https://doi.org/10.1136/bmjopen-2019-029717)

Hall S, Poller B, Bailey C, et al. Use of ultraviolet-fluorescence-based simulation in evaluation of personal protective equipment worn for first assessment and care of a patient with suspected high-consequence infectious disease. *Journal of Hospital Infection*. 2018;99(2):218-228. doi:[10.1016/j.jhin.2018.01.002](https://doi.org/10.1016/j.jhin.2018.01.002)

Mallow M, Gary L, Jeng T, et al. WASH activities at two Ebola treatment units in Sierra Leone. Ikegami T, ed. *PLoS ONE*. 2018;13(5):e0198235. doi:[10.1371/journal.pone.0198235](https://doi.org/10.1371/journal.pone.0198235)

**Wrong intervention (UV radiation)**

Jinadatha C, Simmons S, Dale C, et al. Disinfecting personal protective equipment with pulsed xenon ultraviolet as a risk mitigation strategy for health care workers. American Journal of Infection Control. 2015;43(4):412-414. doi:10.1016/j.ajic.2015.01.013

Keïta M, Camara AY, Traoré F, et al. Impact of infection prevention and control training on health facilities during the Ebola virus disease outbreak in Guinea. *BMC Public Health*. 2018;18(1):547. doi:[10.1186/s12889-018-5444-3](https://doi.org/10.1186/s12889-018-5444-3)

Tremblay N, Musa E, Cooper C, et al. Infection prevention and control in health facilities in post-Ebola Liberia: don’t forget the private sector! *public health action*. 2017;7(1):94-99. doi:[10.5588/pha.16.0098](https://doi.org/10.5588/pha.16.0098)

**Abstract Only**

Kogutt BK, Sheffield JS, Garibaldi BT. 680: Assessing effectiveness of PPE in a simulated SVD of a highly infectious disease patient. *American Journal of Obstetrics and Gynecology*. 2019;220(1):S451. doi:[10.1016/j.ajog.2018.11.703](https://doi.org/10.1016/j.ajog.2018.11.703)

Huber K, Jones I, Dousa T, et al. An Evidence Based Approach to Testing PPE for Enhanced Isolation Precautions during Ebola Virus Disease Preparedness Planning. *American Journal of Infection Control*. 2015;43(6):S69. doi:[10.1016/j.ajic.2015.04.170](https://doi.org/10.1016/j.ajic.2015.04.170)

Diallo A, Diallo M, Hyjazi Y, Waxman R, Pleah T. Baseline evaluation of infection prevention and control (IPC) in the context of Ebola virus disease (EVD) in nine healthcare facilities in the city of Conakry, Guinea. *Antimicrob Resist Infect Control*. 2015;4(S1):O10. doi:[10.1186/2047-2994-4-S1-O10](https://doi.org/10.1186/2047-2994-4-S1-O10)

McCulloch KL, Michael F, Goren M, et al. Creating an Environment of Safety for the Treatment of Patients with Ebola. *American Journal of Infection Control*. 2015;43(6):S73. doi:[10.1016/j.ajic.2015.04.193](https://doi.org/10.1016/j.ajic.2015.04.193)

Okwor T, Tobin-West C, Oduyebo O, et al. Identifying infection prevention and control gaps in healthcare facilities operating in Rivers state during the EVD outbreak in Nigeria 2014. *Antimicrob Resist Infect Control*. 2015;4(S1):O11. doi:[10.1186/2047-2994-4-S1-O11](https://doi.org/10.1186/2047-2994-4-S1-O11)

Barratt R. Infection prevention & control challenges with the management of a suspected case of Ebola Virus Disease. *Infection, Disease & Health*. 2016;21(3):131. doi:[10.1016/j.idh.2016.09.068](https://doi.org/10.1016/j.idh.2016.09.068)

Buregyeya E. Leveraging ebola viral disease emergency preparedness for infection prevention and control in health care facilities. *International Journal of Infectious Diseases*. 2020;101:318. doi:[10.1016/j.ijid.2020.09.829](https://doi.org/10.1016/j.ijid.2020.09.829)

# KQ11

**Not a relevant study**

Haque M. Handwashing in averting infectious diseases: Relevance to COVID-19. *jptcp*. 2020;27(SP1):e37-e52. doi:[10.15586/jptcp.v27SP1.711](https://doi.org/10.15586/jptcp.v27SP1.711)

Omess S, Kaplow R, Green A, et al. Implementation of a Warm Zone Model During the COVID-19 Pandemic. *AJN, American Journal of Nursing*. 2021;121(1):48-54. doi:[10.1097/01.NAJ.0000731664.58705.c3](https://doi.org/10.1097/01.NAJ.0000731664.58705.c3)

Dan-Nwafor CC, Ipadeola O, Smout E, et al. A cluster of nosocomial Lassa fever cases in a tertiary health facility in Nigeria: Description and lessons learned, 2018. *International Journal of Infectious Diseases*. 2019;83:88-94. doi:[10.1016/j.ijid.2019.03.030](https://doi.org/10.1016/j.ijid.2019.03.030)

Degan C. Increased hand hygiene compliance following hospital hygiene measures implemented after an epidemic of Lassa haemorrhagic fever in a network of hospitals republic of Benin. *Antimicrobial Resistance and Infection Control Conference: International Conference on Prevention and Infection Control ICPIC*. Published online 2017.

Ilesanmi OS, Ayodeji OO, Bakare AA, et al. Infection prevention and control (IPC) at a Lassa fever treatment center before and after the implementation of an intensive IPC program. *jidhealth*. 2020;3(3):213-216. doi:[10.47108/jidhealth.Vol3.Iss3.66](https://doi.org/10.47108/jidhealth.Vol3.Iss3.66)

Adeke AS, Onoh RC, Umeokonkwo CD, Azuogu BN, Ogah EO. Knowledge, attitude and practice of infection prevention and control among healthcare workers: one year after an outbreak of nosocomial Lassa fever in a tertiary hospital in southeast NigeriaKnowledge, attitude and practice of infection prevention and con. *Af J Clin Exp Micro*. 2021;22(4):457-464. doi:[10.4314/ajcem.v22i4.5](https://doi.org/10.4314/ajcem.v22i4.5)

Mba S, Ukponu W, Saleh M, et al. Lassa fever infection among health care workers in Nigeria, 2019. *International Journal of Infectious Diseases*. 2020;101:279. doi:[10.1016/j.ijid.2020.09.731](https://doi.org/10.1016/j.ijid.2020.09.731)

Ilesanmi OS, Ayodeji OO, Bakare AA, et al. Status of Infection prevention and control (IPC) at a Lassa fever treatment center before and after the implementation of an intensive IPC program. *jidhealth*. 2020;3(3):213-216. doi:[10.47108/jidhealth.Vol3.Iss3.66](https://doi.org/10.47108/jidhealth.Vol3.Iss3.66)

**No information on the type of hand hygiene method**

Kwon JH, Burnham CAD, Reske KA, et al. Assessment of Healthcare Worker Protocol Deviations and Self-Contamination During Personal Protective Equipment Donning and Doffing. *Infect Control Hosp Epidemiol*. 2017;38(9):1077-1083. doi:[10.1017/ice.2017.121](https://doi.org/10.1017/ice.2017.121)

Ratnayake R, Ho LS, Ansumana R, et al. Improving Ebola infection prevention and control in primary healthcare facilities in Sierra Leone: a single-group pretest post-test, mixed-methods study. *BMJ Glob Health*. 2016;1(4):e000103. doi:[10.1136/bmjgh-2016-000103](https://doi.org/10.1136/bmjgh-2016-000103)

**Population not of interest**

Cook B, Cutts T, Nikiforuk A, et al. Evaluating Environmental Persistence and Disinfection of the Ebola Virus Makona Variant. *Viruses*. 2015;7(4):1975-1986. doi:[10.3390/v7041975](https://doi.org/10.3390/v7041975)

Cutts TA, Robertson C, Theriault SS, et al. Efficacy of microbicides for inactivation of Ebola–Makona virus on a non-porous surface: a targeted hygiene intervention for reducing virus spread. *Sci Rep*. 2020;10(1):15247. doi:[10.1038/s41598-020-71736-x](https://doi.org/10.1038/s41598-020-71736-x)

Gallandat K, Lantagne D. Selection of a Biosafety Level 1 (BSL-1) surrogate to evaluate surface disinfection efficacy in Ebola outbreaks: Comparison of four bacteriophages. Galdiero M, ed. *PLoS ONE*. 2017;12(5):e0177943. doi:[10.1371/journal.pone.0177943](https://doi.org/10.1371/journal.pone.0177943)

Gidado S, Oladimeji AM, Roberts AA, et al. Public Knowledge, Perception and Source of Information on Ebola Virus Disease – Lagos, Nigeria; September, 2014. *PLoS Curr*. Published online 2015. doi:[10.1371/currents.outbreaks.0b805cac244d700a47d6a3713ef2d6db](https://doi.org/10.1371/currents.outbreaks.0b805cac244d700a47d6a3713ef2d6db)

Lantagne D, Wolfe M, Gallandat K, Opryszko M. Determining the Efficacy, Safety and Suitability of Disinfectants to Prevent Emerging Infectious Disease Transmission. *Water*. 2018;10(10):1397. doi:[10.3390/w10101397](https://doi.org/10.3390/w10101397)

Squire JS, Conteh I, Abrahamya A, et al. Gaps in Infection Prevention and Control in Public Health Facilities of Sierra Leone after the 2014–2015 Ebola Outbreak. *TropicalMed*. 2021;6(2):89. doi:[10.3390/tropicalmed6020089](https://doi.org/10.3390/tropicalmed6020089)

Ilesanmi OS, Alele FO. The effect of Ebola Virus Disease outbreak on hand washing among secondary school students in Ondo State Nigeria, October, 2014. *Pan Afr Med J*. 2015;22(Supp 1). doi:[10.11604/pamj.supp.2015.22.1.6614](https://doi.org/10.11604/pamj.supp.2015.22.1.6614)

**Irrelevant method of disinfection**

Cutts TA, Ijaz MK, Nims RW, Rubino JR, Theriault SS. Effectiveness of Dettol Antiseptic Liquid for Inactivation of Ebola Virus in Suspension. *Sci Rep*. 2019;9(1):6590. doi:[10.1038/s41598-019-42386-5](https://doi.org/10.1038/s41598-019-42386-5)

Cutts TA, Nims RW, Theriault SS, Bruning E, Rubino JR, Ijaz MK. Hand hygiene: virucidal efficacy of a liquid hand wash product against Ebola virus. *Infection Prevention in Practice*. 2021;3(1):100122. doi:[10.1016/j.infpip.2021.100122](https://doi.org/10.1016/j.infpip.2021.100122)

Eggers M, Eickmann M, Kowalski K, Zorn J, Reimer K. Povidone-iodine hand wash and hand rub products demonstrated excellent in vitro virucidal efficacy against Ebola virus and modified vaccinia virus Ankara, the new European test virus for enveloped viruses. *BMC Infect Dis*. 2015;15(1):375. doi:[10.1186/s12879-015-1111-9](https://doi.org/10.1186/s12879-015-1111-9)

**No comparator**

Lim SM, Cha WC, Chae MK, Jo IJ. Contamination during doffing of personal protective equipment by healthcare providers. *Clin Exp Emerg Med*. 2015;2(3):162-167. doi:[10.15441/ceem.15.019](https://doi.org/10.15441/ceem.15.019)

Reidy P, Fletcher T, Shieber C, et al. Personal protective equipment solution for UK military medical personnel working in an Ebola virus disease treatment unit in Sierra Leone. *J Hosp Infect*. Published online 2017.

Kogutt BK, Sheffield JS, Garibaldi BT. 680: Assessing effectiveness of PPE in a simulated SVD of a highly infectious disease patient. *American Journal of Obstetrics and Gynecology*. 2019;220(1):S451. doi:[10.1016/j.ajog.2018.11.703](https://doi.org/10.1016/j.ajog.2018.11.703)

Diallo A, Aribot J, Hyjazi Y, Waxman R, Pleah T. Baseline evaluation of infection prevention and control (IPC) in the context of Ebola virus disease (EVD) in nine healthcare facilities in the city of Conakry, Guinea. *Antimicrob Resist Infect Control*. 2015;4(S1):O10. doi:[10.1186/2047-2994-4-S1-O10](https://doi.org/10.1186/2047-2994-4-S1-O10)

Martins SO, Osiyemi AO. HAND HYGIENE PRACTICES POST EBOLA VIRUS DISEASE OUTBREAK IN A NIGERIAN TEACHING HOSPITAL. *Annals of Ibadan postgraduate medicine*. 2017;15.

Kwon JH, Burnham CAD, Reske K, et al. Healthcare Worker Self-Contamination During Standard and Ebola Virus Disease Personal Protective Equipment Doffing. *Open Forum Infectious Diseases*. 2016;3(suppl_1):1387. doi:[10.1093/ofid/ofw172.1090](https://doi.org/10.1093/ofid/ofw172.1090)

Okamoto K, Rhee Y, Schoeny M, et al. Importance of healthcare worker personal protective equipment in reducing doffing errors-correlation with HCW characteristics and perceptions. *Open Forum Infectious Diseases Conference: ID Week*. Published online 2016.

**No relevant comparisons**

Abdulsalam M, Ibrahim A, Michael G, Mijinyawa A. Hand washing practices and techniques among health professionals in a tertiary hospital in Kano. 2015;10(1):5.

Buregyeya E. Leveraging ebola viral disease emergency preparedness for infection prevention and control in health care facilities. *International Journal of Infectious Diseases*. 2020;101:318. doi:[10.1016/j.ijid.2020.09.829](https://doi.org/10.1016/j.ijid.2020.09.829)

Casanova LM, Erukunuakpor K, Kraft CS, et al. Assessing Viral Transfer During Doffing of Ebola-Level Personal Protective Equipment in a Biocontainment Unit. *Clinical Infectious Diseases*. 2018;66(6):945-949. doi:[10.1093/cid/cix956](https://doi.org/10.1093/cid/cix956)

Casanova LM, Teal LJ, Sickbert-Bennett EE, et al. Assessment of Self-Contamination During Removal of Personal Protective Equipment for Ebola Patient Care. *Infect Control Hosp Epidemiol*. 2016;37(10):1156-1161. doi:[10.1017/ice.2016.169](https://doi.org/10.1017/ice.2016.169)

Hopman J, Kubilay Z, Allen T, Edrees H, Pittet D, Allegranzi B. Efficacy of chlorine solutions used for hand hygiene and gloves disinfection in Ebola settings: a systematic review. *Antimicrob Resist Infect Control*. 2015;4(S1):O13. doi:[10.1186/2047-2994-4-S1-O13](https://doi.org/10.1186/2047-2994-4-S1-O13)

Jacquerioz Bausch FA, Heller O, Bengaly L, et al. Building Local Capacity in Hand-Rub Solution Production during the 2014-2016 Ebola Outbreak Disaster: The Case of Liberia and Guinea. *Prehosp Disaster med*. 2018;33(6):660-667. doi:[10.1017/S1049023X18000985](https://doi.org/10.1017/S1049023X18000985)

Kanagasabai U, Enriquez K, Gelting R, et al. The Impact of Water Sanitation and Hygiene (WASH) Improvements on Hand Hygiene at Two Liberian Hospitals during the Recovery Phase of an Ebola Epidemic. *IJERPH*. 2021;18(7):3409. doi:[10.3390/ijerph18073409](https://doi.org/10.3390/ijerph18073409)

Kubilay Z, Hopman J, Allen T, Edrees H, Allegranzi B. Skin side effects of chlorine solutions used for hand hygiene: a systematic review. *Antimicrob Resist Infect Control*. 2015;4(S1):P9, 2047-2994-4-S1-P9. doi:[10.1186/2047-2994-4-S1-P9](https://doi.org/10.1186/2047-2994-4-S1-P9)

Mallow M, Gary L, Jeng T, et al. WASH activities at two Ebola treatment units in Sierra Leone. Ikegami T, ed. *PLoS ONE*. 2018;13(5):e0198235. doi:[10.1371/journal.pone.0198235](https://doi.org/10.1371/journal.pone.0198235)

Mumma JM, Durso FT, Casanova LM, et al. Common Behaviors and Faults When Doffing Personal Protective Equipment for Patients With Serious Communicable Diseases. *Clinical Infectious Diseases*. 2019;69(Supplement_3):S214-S220. doi:[10.1093/cid/ciz614](https://doi.org/10.1093/cid/ciz614)

Ogoina D, Oyeyemi AS, Ayah O, et al. Preparation and Response to the 2014 Ebola Virus Disease Epidemic in Nigeria—The Experience of a Tertiary Hospital in Nigeria. Warburton D, ed. *PLoS ONE*. 2016;11(10):e0165271. doi:[10.1371/journal.pone.0165271](https://doi.org/10.1371/journal.pone.0165271)

Siddharta A, Pfaender S, Vielle NJ, et al. Virucidal Activity of World Health Organization–Recommended Formulations Against Enveloped Viruses, Including Zika, Ebola, and Emerging Coronaviruses. *The Journal of Infectious Diseases*. 2017;215(6):902-906. doi:[10.1093/infdis/jix046](https://doi.org/10.1093/infdis/jix046)

Suen LKP, Guo YP, Tong DWK, et al. Self-contamination during doffing of personal protective equipment by healthcare workers to prevent Ebola transmission. *Antimicrob Resist Infect Control*. 2018;7(1):157. doi:[10.1186/s13756-018-0433-y](https://doi.org/10.1186/s13756-018-0433-y)

Tantum LK, Gilstad JR, Bolay FK, et al. Barriers and Opportunities for Sustainable Hand Hygiene Interventions in Rural Liberian Hospitals. *IJERPH*. 2021;18(16):8588. doi:[10.3390/ijerph18168588](https://doi.org/10.3390/ijerph18168588)

Verbeek JH, Rajamaki B, Ijaz S, et al. Personal protective equipment for preventing highly infectious diseases due to exposure to contaminated body fluids in healthcare staff. Cochrane Work Group, ed. *Cochrane Database of Systematic Reviews*. Published online April 15, 2020. doi:[10.1002/14651858.CD011621.pub4](https://doi.org/10.1002/14651858.CD011621.pub4)

Wells E, Wolfe MK, Murray A, Lantagne D. Accuracy, Precision, Ease-Of-Use, and Cost of Methods to Test Ebola-Relevant Chlorine Solutions. Munster VJ, ed. *PLoS ONE*. 2016;11(5):e0152442. doi:[10.1371/journal.pone.0152442](https://doi.org/10.1371/journal.pone.0152442)

Wolfe MK, Gallandat K, Daniels K, Desmarais AM, Scheinman P, Lantagne D. Handwashing and Ebola virus disease outbreaks: A randomized comparison of soap, hand sanitizer, and 0.05% chlorine solutions on the inactivation and removal of model organisms Phi6 and E. coli from hands and persistence in rinse water. Cameron DW, ed. *PLoS ONE*. 2017;12(2):e0172734. doi:[10.1371/journal.pone.0172734](https://doi.org/10.1371/journal.pone.0172734)

Wolfe MK, Wells E, Mitro B, Desmarais AM, Scheinman P, Lantagne D. Seeking Clearer Recommendations for Hand Hygiene in Communities Facing Ebola: A Randomized Trial Investigating the Impact of Six Handwashing Methods on Skin Irritation and Dermatitis. Cameron DW, ed. *PLoS ONE*. 2016;11(12):e0167378. doi:[10.1371/journal.pone.0167378](https://doi.org/10.1371/journal.pone.0167378)

Lee M a, Huh K, Jeong J, et al. Adherence to Protocols by Healthcare Workers and Self-Contamination During Doffing of Personal Protective Equipment. *American Journal of Infection Control*. 2018;46(6):S11. doi:[10.1016/j.ajic.2018.04.024](https://doi.org/10.1016/j.ajic.2018.04.024)

Almutairi KM, Alodhayani AA, Moussa M, Aboshaiqah AE, Tumala RB, Vinluan JM. Ebola outbreak preparedness and preventive measures among healthcare providers in Saudi Arabia. *J Infect Dev Ctries*. 2016;10(08):829-836. doi:[10.3855/jidc.6941](https://doi.org/10.3855/jidc.6941)

Robinson G, Kpadeh ZZ, Alserehi H, et al. Effect of glove disinfection on bacterial contamination of healthcare worker hands. *Open Forum Infectious Diseases*. Published online 2018.

Gao P, Horvatin M, Niezgoda G, Weible R, Shaffer R. Effect of multiple alcohol-based hand rub applications on the tensile properties of thirteen brands of medical exam nitrile and latex gloves. *Journal of Occupational and Environmental Hygiene*. 2016;13(12):905-914. doi:[10.1080/15459624.2016.1191640](https://doi.org/10.1080/15459624.2016.1191640)

Obionu IM, Ochu CL, Ukponu W, et al. Evaluation of infection prevention and control practices in Lassa fever treatment centers in north-central Nigeria during an ongoing Lassa fever outbreak. *Journal of Infection Prevention*. 2021;22(6):275-282. doi:[10.1177/17571774211035838](https://doi.org/10.1177/17571774211035838)

Akinyinka MR, Bakare OQ, Oluwole EO, Odugbemi BA. Hand hygiene practices in the context of Ebola virus disease: A cross-sectional survey of Lagos residents. *Journal of Infection Prevention*. 2019;20(4):179-184. doi:[10.1177/1757177419830779](https://doi.org/10.1177/1757177419830779)

Soeters HM, Koivogui L, De Beer L, et al. Infection prevention and control training and capacity building during the Ebola epidemic in Guinea. Andrei G, ed. *PLoS ONE*. 2018;13(2):e0193291. doi:[10.1371/journal.pone.0193291](https://doi.org/10.1371/journal.pone.0193291)

Infection Control Africa Network Education and Training Working Group, Mehtar S, Hakizimana B. IPC training in Sierra Leone- ICAN’s role in fighting Ebola. *Antimicrob Resist Infect Control*. 2015;4(S1):O12. doi:[10.1186/2047-2994-4-S1-O12](https://doi.org/10.1186/2047-2994-4-S1-O12)

Aissi A, Ahoyo A, Yorou CO, et al. Knowledge of health care workers and ability of healthcare facilities in preventing of Ebola virus diseases/lassa fever in Benin. *Antimicrob Resist Infect Control*. 2015;4(S1):P4, 2047-2994-4-S1-P4. doi:[10.1186/2047-2994-4-S1-P4](https://doi.org/10.1186/2047-2994-4-S1-P4)

Ijarotimi IT, Ilesanmi OS, Aderinwale A, Abiodun-Adewusi O. Knowledge of Lassa fever and use of infection prevention and control facilities among health care workers during Lassa fever outbreak in Ondo State, Nigeria.

Pululu D, Mukendi S, Formenty P, Eremin S, Pessoa-Silva C. O090: Impact on nurses of ebola outbreak. *Antimicrob Resist Infect Control*. 2013;2(S1):O90. doi:[10.1186/2047-2994-2-S1-O90](https://doi.org/10.1186/2047-2994-2-S1-O90)

Anders PL, Townsend NE, Davis EL, McCall WD. Observed infection control compliance in a dental school: A natural experiment. *American Journal of Infection Control*. 2016;44(9):e153-e156. doi:[10.1016/j.ajic.2016.01.036](https://doi.org/10.1016/j.ajic.2016.01.036)

Eiras D, Echeverri A, Toale K, Tennill P, Evans L. Painting the gown red: Using a colored paint quality improvement process to evaluate healthcare worker personal protective equipment for highly pathogenic infections. *Open Forum Infectious Diseases*. Published online 2017.

Iqbal Q, Lubeck-Schricker M, Wells E, Wolfe MK, Lantagne D. Shelf-Life of Chlorine Solutions Recommended in Ebola Virus Disease Response. Munster VJ, ed. *PLoS ONE*. 2016;11(5):e0156136. doi:[10.1371/journal.pone.0156136](https://doi.org/10.1371/journal.pone.0156136)

Gallandat K, Wolfe MK, Lantagne D. Surface Cleaning and Disinfection: Efficacy Assessment of Four Chlorine Types Using *Escherichia coli* and the Ebola Surrogate Phi6. *Environ Sci Technol*. 2017;51(8):4624-4631. doi:[10.1021/acs.est.6b06014](https://doi.org/10.1021/acs.est.6b06014)

Birnbach DJ, Thiesen TC, McKenty NT, et al. Targeted Use of Alcohol-Based Hand Rub on Gloves During Task Dense Periods: One Step Closer to Pathogen Containment by Anesthesia Providers in the Operating Room. *Anesthesia & Analgesia*. 2019;129(6):1557-1560. doi:[10.1213/ANE.0000000000004107](https://doi.org/10.1213/ANE.0000000000004107)

Mehtar S. The impact of education on reducing Ebola virus disease transmission in healthcare facilities. *International Journal of Infectious Diseases*. 2016;45:66-67. doi:[10.1016/j.ijid.2016.02.193](https://doi.org/10.1016/j.ijid.2016.02.193)

Ousman K, Kabego L, Talisuna A, et al. The impact of **Infection Prevention and control (IPC) bundle implementation** on IPC compliance during the Ebola virus outbreak in Mbandaka/Democratic Republic of the Congo: a before and after design. *BMJ Open*. 2019;9(9):e029717. doi:[10.1136/bmjopen-2019-029717](https://doi.org/10.1136/bmjopen-2019-029717)

**Full text Unavailable**

Garibaldi BT, Rainwater-Lovett K, Pilholski T, et al. Transmission of fluorescent aerosolized particles in a clinical biocontainment unit. *American Journal of Respiratory and Critical Care Medicine Conference: American Thoracic Society International Conference ATS*. Published online 2017.

Kratz T, Verbeek L. Discussion of two infection prevention and control training approaches to enhance biosafety in primary healthcare facilities during an outbreak of Ebola virus disease. *Tropical Medicine and International Health*. Published online 2017.

Ibeneme S, Maduako G, Ibeneme G. Hand hygiene practices among physiotherapists in an ebola endemic region: Implication for public health. *Physiotherapy (United Kingdom)*. Published online 2015.

Nelson AR, Fiedler A, Zikeh T, Moses N, Ruparelia C, Oseni L. Institutionalizing infection prevention and control practices in health facilities in liberia following the Ebola epidemic. *American Journal of Tropical Medicine and Hygiene*. Published online 2018.

Ngauja RE, Conteh CA, Maruta A. Assessment of hand hygiene compliance among 4 wards at the university of Sierra Leone teaching hospital. Published online 2019.

Ahoyo T, Gazard DK, Gounongbe M. Evaluation of basic infection prevention practices in health care set-ups in Benin. *Antimicrobial Resistance and Infection Control Conference: International Conference on Prevention and Infection Control ICPIC*. Published online 2017.

Kolee TS. Hand hygiene compliance among healthcare workers in Liberia: Hand hygiene audit result. *Antimicrobial Resistance and Infection Control Conference: International Conference on Prevention and Infection Control ICPIC*. Published online 2017.

Kerwillain G. Hand hygiene self-assessment framework: Baseline assessments to determine hand hygiene practice level and facilitate planning for improvement in healthcare facilities in Liberia. *Antimicrobial Resistance and Infection Control Conference: International Conference on Prevention and Infection Control ICPIC*. Published online 2017.

Mayah-Toto F, Enriquez K, Udhayashankar KK, Niescierenko M. Hand hygiene: Linking training to implementation and outcomes. *Antimicrobial Resistance and Infection Control Conference: International Conference on Prevention and Infection Control ICPIC*. Published online 2017.

Kabore RP, Kabore DSR, Zida S, Ouedraogo NW, Verbeek L, Gies S. Improving basic hygiene among health care workers through Ebola-training: A field perspective. *Tropical Medicine and International Health*. Published online 2017.

Somers Y, Verbiest M. Suspecting ebola: When the dress code becomes life saving! Personal protective equipment-a practical demonstration. *Anaesthesiology Intensive Therapy*. Published online 2014.

Cooper CT. Using data to enhance implementation in a low resource setting-Liberia expereince. *Antimicrobial Resistance and Infection Control Conference: International Conference on Prevention and Infection Control ICPIC*. Published online 2017.

Raj D. What are the appropriate personal protective equipment (PPE) for frontline workers (FLW) caring for filovirus/ebola virus disease (EVD) patients? *Open Forum Infectious Diseases*. Published online 2017.

**Duplicate Study**

Brill FH, Siddharta A, Pfaender S, Vielle NJ, et al. Virucidal Activity of World Health Organization–Recommended Formulations Against Enveloped Viruses, Including Zika, Ebola, and Emerging Coronaviruses. *The Journal of Infectious Diseases*. 2017;215(6):902-906. doi:[10.1093/infdis/jix046](https://doi.org/10.1093/infdis/jix046)

Squire JS, Conteh I, Abrahamya A, et al. Gaps in Infection Prevention and Control in Public Health Facilities of Sierra Leone after the 2014–2015 Ebola Outbreak. *TropicalMed*. 2021;6(2):89. doi:[10.3390/tropicalmed6020089](https://doi.org/10.3390/tropicalmed6020089)

Mumma JM, Durso FT, Casanova LM, et al. Common Behaviors and Faults When Doffing Personal Protective Equipment for Patients With Serious Communicable Diseases. *Clinical Infectious Diseases*. 2019;69(Supplement_3):S214-S220. doi:[10.1093/cid/ciz614](https://doi.org/10.1093/cid/ciz614)

Narrative Review

Raj D, Hornsey E, Perl TM. Personal protective equipment for viral hemorrhagic fevers: *Current Opinion in Infectious Diseases*. 2019;32(4):337-347. doi:[10.1097/QCO.0000000000000562](https://doi.org/10.1097/QCO.0000000000000562)

# KQ12

**Does not examine Ebola or Marburg (or surrogate viruses)**

Rhee SW. Management of used personal protective equipment and wastes related to COVID-19 in South Korea. *Waste Manag Res*. 2020;38(8):820-824. doi:[10.1177/0734242X20933343](https://doi.org/10.1177/0734242X20933343)

Otter J, Barnicoat M, Down J, Smyth D, Yezli S, Jeanes A. Hydrogen peroxide vapor (HPV) decontamination of an intensive care unit room used to treat a patient with Lassa fever. *Journal of Hospital Infection*. Published online 2010.

Roy KM, Ahmed S, Inkster T, Smith A, Penrice G. Managing the risk of viral haemorrhagic fever transmission in a non-high-level intensive care unit: experiences from a case of Crimean-Congo haemorrhagic fever in Scotland. *Journal of Hospital Infection*. 2016;93(3):304-308. doi:[10.1016/j.jhin.2016.02.023](https://doi.org/10.1016/j.jhin.2016.02.023)

**Non-comparative study**

Cummings KJ, Choi MJ, Esswein EJ, et al. Addressing Infection Prevention and Control in the First U.S. Community Hospital to Care for Patients With Ebola Virus Disease: Context for National Recommendations and Future Strategies. *Ann Intern Med*. 2016;165(1):41. doi:[10.7326/M15-2944](https://doi.org/10.7326/M15-2944)

Edmunds KL, Elrahman SA, Bell DJ, et al. Recommendations for dealing with waste contaminated with Ebola virus: a Hazard Analysis of Critical Control Points approach. *Bull World Health Organ*. 2016;94(6):424-432. doi:[10.2471/BLT.15.163931](https://doi.org/10.2471/BLT.15.163931)

Garibaldi BT, Kelen GD, Brower RG, et al. The Creation of a Biocontainment Unit at a Tertiary Care Hospital. The Johns Hopkins Medicine Experience. *Annals ATS*. 2016;13(5):600-608. doi:[10.1513/AnnalsATS.201509-587PS](https://doi.org/10.1513/AnnalsATS.201509-587PS)

Garibaldi B, Ernst N, Reimers M, et al. Establishing a New Biocontainment and Treatment Unit. *Chest*. 2015;148(4):248A. doi:[10.1378/chest.2268190](https://doi.org/10.1378/chest.2268190)

Hewlett AL, Varkey JB, Smith PW, Ribner BS. Ebola virus disease: preparedness and infection control lessons learned from two biocontainment units. *Current Opinion in Infectious Diseases*. 2015;28(4):343-348. doi:[10.1097/QCO.0000000000000176](https://doi.org/10.1097/QCO.0000000000000176)

Herstein JJ, Biddinger PD, Gibbs SG, et al. High-Level Isolation Unit Infection Control Procedures. *Health Security*. 2017;15(5):519-526. doi:[10.1089/hs.2017.0026](https://doi.org/10.1089/hs.2017.0026)

Herstein JJ, Biddinger PD, Kraft CS, et al. Current Capabilities and Capacity of Ebola Treatment Centers in the United States. *Infect Control Hosp Epidemiol*. 2016;37(3):313-318. doi:[10.1017/ice.2015.300](https://doi.org/10.1017/ice.2015.300)

Haverkort JJM, Minderhoud ALC (Ben), Wind JDD, Leenen LPH, Hoepelman AIM, Ellerbroek PM. Hospital Preparations for Viral Hemorrhagic Fever Patients and Experience Gained from Admission of an Ebola Patient. *Emerg Infect Dis*. 2016;22(2):184-191. doi:[10.3201/eid2202.151393](https://doi.org/10.3201/eid2202.151393)

Kyomba GK, Konde JNN, Saila-Ngita D, Solo TK, Kiyombo GM. Assessing the management of healthcare waste for disease prevention and environment protection at selected hospitals in Kinshasa, Democratic Republic of Congo. *Waste Manag Res*. 2021;39(10):1237-1244. doi:[10.1177/0734242X211048132](https://doi.org/10.1177/0734242X211048132)

Le AB, Hoboy S, Germain A, et al. A pilot survey of the U.S. medical waste industry to determine training needs for safely handling highly infectious waste. *American Journal of Infection Control*. 2018;46(2):133-138. doi:[10.1016/j.ajic.2017.08.017](https://doi.org/10.1016/j.ajic.2017.08.017)

McCulloch KL, Michael F, Goren M, et al. Creating an Environment of Safety for the Treatment of Patients with Ebola. *American Journal of Infection Control*. 2015;43(6):S73. doi:[10.1016/j.ajic.2015.04.193](https://doi.org/10.1016/j.ajic.2015.04.193)

Otter JA, Barnicoat M, Down J, Smyth D, Yezli S, Jeanes A. Hydrogen peroxide vapour decontamination of a critical care unit room used to treat a patient with Lassa fever. *Journal of Hospital Infection*. 2010;75(4):335-337. doi:[10.1016/j.jhin.2010.02.025](https://doi.org/10.1016/j.jhin.2010.02.025)

Onoh R, Adeke A, Umeokonkwo C, Ekwedigwe K, Agboeze J, Ogah E. Knowledge and practices of health-care waste management among health Workers in Lassa fever treatment facility in Southeast Nigeria. *Niger Med J*. 2019;60(5):257. doi:[10.4103/nmj.NMJ_161_18](https://doi.org/10.4103/nmj.NMJ_161_18)

Perpoint T, Valour F, Gerbier-Colomban S, et al. Knowledge Attitude and Practice (KAP) on Ebola Virus Disease (EVD) Among Health Care Workers (HCWs) From the Lyon Teaching Hospitals, France. *Open Forum Infectious Diseases*. 2016;3(suppl_1):602. doi:[10.1093/ofid/ofw172.465](https://doi.org/10.1093/ofid/ofw172.465)

Sarti AJ, Sutherland S, Robillard N, et al. Ebola preparedness: a rapid needs assessment of critical care in a tertiary hospital. *CMAJ Open*. 2015;3(2):E198-E207. doi:[10.9778/cmajo.20150025](https://doi.org/10.9778/cmajo.20150025)

Sisler L, Hanlon V. Supporting Emerging Infectious Disease Education Through Utilization of “At-A-Glance” Guides for Infection Prevention and Containment Unit Staff. *American Journal of Infection Control*. 2016;44(6):S124-S125. doi:[10.1016/j.ajic.2016.04.151](https://doi.org/10.1016/j.ajic.2016.04.151)

**No relevant comparisons**

Garibaldi BT, Reimers M, Ernst N, et al. Validation of Autoclave Protocols for Successful Decontamination of Category A Medical Waste Generated from Care of Patients with Serious Communicable Diseases. McAdam AJ, ed. *J Clin Microbiol*. 2017;55(2):545-551. doi:[10.1128/JCM.02161-16](https://doi.org/10.1128/JCM.02161-16)

**Full text Unavailable**

Bangura I, Conteh C. The Impact of Quality Improvement Methodology to Improve Infection Control Practices. Antimicrobial Resistance & Infection Control. 2019;8(1):P405.

Bustamante ND, O’Keeffe D, Bradley D, Pozner CN. Targeted interprofessional simulation-based training for safe patient management of Ebola virus disease. Academic Emergency Medicine. Published online 2015.

Cazares M, Hutson M, Lakhani U, Herndon D. Implementation of an infectious disease control plan requiring category-a personal protective equipment. Journal of Burn Care and Research. Published online 2016.

**Search 1 - List of Excluded Studies**

**Full text Unavailable (n=5)**

Hayman DTS. Integrating risk analyses in Ebola virus disease outbreak surveillance. Virologie.

Mbala-Kingebeni P, Kinganda-Lusamaki E, Amuri-Aziza A, et al. Sequencing Ebola outbreaks in the Democratic Republic of the Congo (DRC). *Virologie*.

Mvumbi G, Okitolonda V, Toi A, Braga C, Kimpanga P. Secondary attack rate of ebola virus disease 2019 in Beni Democratic Republic of Congo Households from January to November 2019. *American Journal of Tropical Medicine and Hygiene*.

Saez A. Localizing Ebola virus emergences: the intertwining of daily life and EVD outbreaks in Guinea and in the Democratic Republic of the Congo. *Virologie*.

Sikorska K. Selected viral infections in the tropical zone. *European Journal of Translational and Clinical Medicine*.

**Wrong intervention (n=69)**

Abdul-Rahman T, Lawal L, Meale E, et al. Inequitable access to Ebola vaccines and the resurgence of Ebola in Africa: A state of arts review. *J Med Virol*. 2023;95(8):e28986.

Abramowitz S, Stevens LA, Kyomba G, Mayaka S, Grépin KA. Data flows during public health emergencies in LMICs: A people-centered mapping of data flows during the 2018 ebola epidemic in Equateur, DRC. *Social Science & Medicine*. 2023;318:115116. doi:[10.1016/j.socscimed.2022.115116](https://doi.org/10.1016/j.socscimed.2022.115116)

Aderinto N. A reflection on the Marburg virus outbreak in Tanzania: the importance of preparedness and prevention in public health – a correspondence. *Annals of Medicine & Surgery*. 2023;85(5):2247-2249. doi:[10.1097/MS9.0000000000000596](https://doi.org/10.1097/MS9.0000000000000596)

Ahmed I, Salsabil L, Hossain MdJ, Shahriar M, Bhuiyan MA, Islam MdR. The recent outbreaks of Marburg virus disease in African countries are indicating potential threat to the global public health: Future prediction from historical data. *Health Science Reports*. 2023;6(7):e1395. doi:[10.1002/hsr2.1395](https://doi.org/10.1002/hsr2.1395)

Ankunda C, Kanyesigye SM, Nakubulwa S, et al. Assessment of health care workers preparedness to epidemics: A case of Ebola virus disease preparedness in private hospitals in Kampala, Uganda. *J Infect Dev Ctries*. 2024;18(04):556-564. doi:[10.3855/jidc.17642](https://doi.org/10.3855/jidc.17642)

Bisimwa P, Biamba C, Aborode AT, Cakwira H, Akilimali A. Ebola virus disease outbreak in the Democratic Republic of the Congo: A mini-review. *Annals of Medicine & Surgery*. 2022;80. doi:[10.1016/j.amsu.2022.104213](https://doi.org/10.1016/j.amsu.2022.104213)

Bouba A, Helle KB, Schneider KA. Predicting the combined effects of case isolation, safe funeral practices, and contact tracing during Ebola virus disease outbreaks. Rychtář J, ed. *PLoS ONE*. 2023;18(1):e0276351. doi:[10.1371/journal.pone.0276351](https://doi.org/10.1371/journal.pone.0276351)

Bulimbe DB, Masunga DS, Paul IK, et al. Marburg virus disease outbreak in Tanzania: current efforts and recommendations – a short communication. *Annals of Medicine & Surgery*. 2023;85(8):4190-4193. doi:[10.1097/MS9.0000000000001063](https://doi.org/10.1097/MS9.0000000000001063)

Cénat JM, Broussard C, Darius WP, et al. Social mobilization, education, and prevention of the Ebola virus disease: A scoping review. *Preventive Medicine*. 2023;166:107328. doi:[10.1016/j.ypmed.2022.107328](https://doi.org/10.1016/j.ypmed.2022.107328)

Collier KM, Klein EK, Sevalie S, et al. Ebola Virus Disease Sensitization: Community-Driven Efforts in Sierra Leone. *J Community Health*. 2024;49(1):108-116. doi:[10.1007/s10900-023-01265-x](https://doi.org/10.1007/s10900-023-01265-x)

Crea TM, Collier KM, Klein EK, et al. Social distancing, community stigma, and implications for psychological distress in the aftermath of Ebola virus disease. Hodges MH, ed. *PLoS ONE*. 2022;17(11):e0276790. doi:[10.1371/journal.pone.0276790](https://doi.org/10.1371/journal.pone.0276790)

Diarra T, Okeibunor J, Diallo B, et al. Community Involvement in Response to Ebola Virus Disease Epidemic in North Kivu and Ituri, Democratic Republic of Congo: A Mixed-Methods Study. *J Immunological Sci*. 2023;S3(3):131-140. doi:[10.29245/2578-3009/2023/S3.1110](https://doi.org/10.29245/2578-3009/2023/S3.1110)

Dine RD, Umutoni AU, Umulisa MM, et al. Best practices and lessons learned from implementing a massive Ebola vaccination program: Summarizing UMURINZI team experience. *Health Science Reports*. 2023;6(10):e1618. doi:[10.1002/hsr2.1618](https://doi.org/10.1002/hsr2.1618)

Doshi RH, Garbern SC, Kulkarni S, et al. Ebola vaccine uptake and attitudes among healthcare workers in North Kivu, Democratic Republic of the Congo, 2021. *Front Public Health*. 2023;11:1080700. doi:[10.3389/fpubh.2023.1080700](https://doi.org/10.3389/fpubh.2023.1080700)

Elsheikh R, Makram AM, Selim H, et al. Reemergence of Marburgvirus disease: Update on current control and prevention measures and review of the literature. *Reviews in Medical Virology*. 2023;33(5):e2461. doi:[10.1002/rmv.2461](https://doi.org/10.1002/rmv.2461)

Fairhead J, Leach M, Millimouno D. Spillover or endemic? Reconsidering the origins of Ebola virus disease outbreaks by revisiting local accounts in light of new evidence from Guinea. *BMJ Glob Health*. 2021;6(4):e005783. doi:[10.1136/bmjgh-2021-005783](https://doi.org/10.1136/bmjgh-2021-005783)

Hanson‑DeFusco J, Shi M, Du Z, Zounon O, Hounnouvi FM, DeFusco A. Systems analysis of the effects of the 2014‑16 Ebola crisis on WHO‑reporting nations’ policy adaptations and 2020‑21 COVID‑19 response: a systematized review. *Globalization and Health*. 2023;19(96).

Ho LS, Bertone MP, Mansour W, Masaka C, Kakesa J. Health system resilience during COVID-19 understanding SRH service adaptation in North Kivu. *Reprod Health*. 2022;19(1):135. doi:[10.1186/s12978-022-01443-5](https://doi.org/10.1186/s12978-022-01443-5)

Ibrahim SK, Ndwandwe DE, Thomas K, Sigfrid L, Norton A. Sudan virus disease outbreak in Uganda: urgent research gaps. *BMJ Glob Health*. 2022;7(12):e010982. doi:[10.1136/bmjgh-2022-010982](https://doi.org/10.1136/bmjgh-2022-010982)

Jain S, Khaiboullina S, Martynova E, Morzunov S, Baranwal M. Epidemiology of Ebolaviruses from an Etiological Perspective. *Pathogens*. 2023;12(2):248. doi:[10.3390/pathogens12020248](https://doi.org/10.3390/pathogens12020248)

Judson SD. 1366. Re-examining the Origins of Ebola virus Emergence. *Open Forum Infectious Diseases*. 2022;9(Supplement_2):ofac492.1195. doi:[10.1093/ofid/ofac492.1195](https://doi.org/10.1093/ofid/ofac492.1195)

Kallay R, Mbuyi G, Eggers C, et al. Assessment of the integrated disease surveillance and response system implementation in health zones at risk for viral hemorrhagic fever outbreaks in North Kivu, Democratic Republic of the Congo, following a major Ebola outbreak, 2021. *BMC Public Health*. 2024;24(1):1150. doi:[10.1186/s12889-024-18642-3](https://doi.org/10.1186/s12889-024-18642-3)

Karuhije J, Nkeshimana M, Zakham F, et al. Understanding knowledge, attitudes and practices on Ebola Virus Disease: a multi-site mixed methods survey on preparedness in Rwanda. *BMC Public Health*. 2023;23(1):2417. doi:[10.1186/s12889-023-17251-w](https://doi.org/10.1186/s12889-023-17251-w)

Keita M, Cherif IS, Polonsky JA, et al. Factors Associated with Reliable Contact Tracing During the 2021 Ebola Virus Disease Outbreak in Guinea. *J Epidemiol Glob Health*. Published online February 19, 2024. doi:[10.1007/s44197-024-00202-y](https://doi.org/10.1007/s44197-024-00202-y)

Keita M, Polonsky J, Finci I, et al. Investigation of and Strategies to Control the Final Cluster of the 2018–2020 Ebola Virus Disease Outbreak in the Eastern Democratic Republic of Congo. *Open Forum Infectious Diseases*. 2022;9(9):ofac329. doi:[10.1093/ofid/ofac329](https://doi.org/10.1093/ofid/ofac329)

Keita M, Talisuna A, Chamla D, et al. Investing in preparedness for rapid detection and control of epidemics: analysis of health system reforms and their effect on 2021 Ebola virus disease epidemic response in Guinea. *BMJ Glob Health*. 2023;8(1):e010984. doi:[10.1136/bmjgh-2022-010984](https://doi.org/10.1136/bmjgh-2022-010984)

Kennedy SB, Doumbia S, Mason-Ross G, et al. PA-807 West African consortium for clinical research on epidemic pathogens (WAC-CREP): sub-regional collaborative model to strengthen health systems for emerging infectious diseases (EIDs). In: *Abstracts of Poster and E-Poster Presentations*. BMJ Publishing Group Ltd; 2023:A125.1-A125. doi:[10.1136/bmjgh-2023-EDC.306](https://doi.org/10.1136/bmjgh-2023-EDC.306)

Kibuuka R, Kagoya EK, Nsubuga AllanG, et al. Ebola Virus Disease: Knowledge, Attitude and Perception - The Case of Uganda. Published online September 26, 2023. doi:[10.21203/rs.3.rs-3384769/v1](https://doi.org/10.21203/rs.3.rs-3384769/v1)

Kinganda-Lusamaki E, Whitmer S, Lokilo-Lofiko E, et al. 2020 Ebola virus disease outbreak in Équateur Province, Democratic Republic of the Congo: a retrospective genomic characterisation. *The Lancet Microbe*. 2024;5(2):e109-e118. doi:[10.1016/S2666-5247(23)00259-8](https://doi.org/10.1016/S2666-5247(23)00259-8)

Kousoulis A, Grant I, Duncan J, Larson H. Revisiting the Ebola Epidemic in West Africa: The Role Of Emotional Determinants In Public Responses. *Afr J Infect Dis*. 17(2).

Kritsky AA, Keita S, uba NM, et al. Ebola virus disease outbreak in the Republic of Guinea 2021: hypotheses of origin. *bioRxiv preprint*. Published online 2021.

Kyomba GK, Kiyombo GM, Grépin KA, et al. Assessing routine health information system performance during the tenth outbreak of Ebola virus disease (2018–2020) in the Democratic Republic of the Congo: A qualitative study in North Kivu. Probandari A, ed. *PLOS Glob Public Health*. 2022;2(7):e0000429. doi:[10.1371/journal.pgph.0000429](https://doi.org/10.1371/journal.pgph.0000429)

Letafati A, Salahi Ardekani O, Karami H, Soleimani M. Ebola virus disease: A narrative review. *Microbial Pathogenesis*. 2023;181:106213. doi:[10.1016/j.micpath.2023.106213](https://doi.org/10.1016/j.micpath.2023.106213)

Mahon B, Reynolds M, Russell K, Dunning J. Using Epidemic Intelligence to Inform UK Public Health Response to Infectious Disease Threats, such as Ebola Virus Disease. *International Journal of Infectious Diseases*. 2022;116:S113. doi:[10.1016/j.ijid.2021.12.266](https://doi.org/10.1016/j.ijid.2021.12.266)

Meltzer E, Schwartz E. Ebola and Marburg Virus Infections in Resource-Rich Countries: Implications for Future Outbreaks. *Curr Infect Dis Rep*. 2023;25(9):181-188. doi:[10.1007/s11908-023-00810-y](https://doi.org/10.1007/s11908-023-00810-y)

Millimouno TM, Meessen B, Put WVD, et al. How has Guinea learnt from the response to outbreaks? A learning health system analysis. *BMJ Glob Health*. 2023;8(2):e010996. doi:[10.1136/bmjgh-2022-010996](https://doi.org/10.1136/bmjgh-2022-010996)

Moso MA, Lim CK, Williams E, Marshall C, McCarthy J, Williamson DA. Prevention and post-exposure management of occupational exposure to Ebola virus. *The Lancet Infectious Diseases*. 2024;24(2):e93-e105. doi:[10.1016/S1473-3099(23)00376-6](https://doi.org/10.1016/S1473-3099(23)00376-6)

Mukadi-Bamuleka D, Nkuba-Ndaye A, Mbala-Kingebeni P, Ahuka-Mundeke S, Muyembe-Tamfum JJ. Impact of Ebola epidemics on the daily operation of existing systems in Eastern Democratic Republic of the Congo: a brief review. *Journal of Medical Economics*. 2024;27(1):184-192. doi:[10.1080/13696998.2024.2305009](https://doi.org/10.1080/13696998.2024.2305009)

Mulenga-Cilundika P, Ekofo J, Kabanga C, Criel B, Van Damme W, Chenge F. Indirect Effects of Ebola Virus Disease Epidemics on Health Systems in the Democratic Republic of the Congo, Guinea, Sierra Leone and Liberia: A Scoping Review Supplemented with Expert Interviews. *IJERPH*. 2022;19(20):13113. doi:[10.3390/ijerph192013113](https://doi.org/10.3390/ijerph192013113)

Naiga HN, Zalwango JF, Kizito SN, et al. The Role of Community Beliefs and Practices on the Spread of Ebola in Uganda, September 2022. Published online December 6, 2023. doi:[10.1101/2023.12.05.23299506](https://doi.org/10.1101/2023.12.05.23299506)

Ndaliko Augustin M, Kahindo Mbeva JB. Knowledge, attitudes, and behaviors of healthcare professionals at the start of an Ebola virus epidemic. *Infectious Diseases Now*. 2021;51(1):50-54. doi:[10.1016/j.medmal.2020.04.010](https://doi.org/10.1016/j.medmal.2020.04.010)

Nyakarahuka L, Mulei S, Whitmer S, et al. First laboratory confirmation and sequencing of Zaire ebolavirus in Uganda following two independent introductions of cases from the 10th Ebola Outbreak in the Democratic Republic of the Congo, June 2019. Yakob L, ed. *PLoS Negl Trop Dis*. 2022;16(2):e0010205. doi:[10.1371/journal.pntd.0010205](https://doi.org/10.1371/journal.pntd.0010205)

Obeng-Kusi M, Martin J, Abraham I. The economic burden of Ebola virus disease: a review and recommendations for analysis. *Journal of Medical Economics*. 2024;27(1):309-323. doi:[10.1080/13696998.2024.2313358](https://doi.org/10.1080/13696998.2024.2313358)

Okeibunor J, Diarra T, Onyeneho N, et al. Survivors and the Response to the Ebola Virus Disease in the Provinces of North Kivu and Ituri in the Democratic Republic of Congo. *J Immunological Sci*. 2023;S3(3):31-43. doi:[10.29245/2578-3009/2023/S3.1105](https://doi.org/10.29245/2578-3009/2023/S3.1105)

Okesanya OJ, Manirambona E, Olaleke NO, et al. Rise of Marburg virus in Africa: a call for global preparedness. *Annals of Medicine & Surgery*. 2023;85(10):5285-5290. doi:[10.1097/MS9.0000000000001257](https://doi.org/10.1097/MS9.0000000000001257)

Olum R, Ahaisibwe B, Atuhairwe I, et al. Readiness To Manage Ebola Virus Disease Among Emergency Healthcare Workers in Uganda: A Nationwide Multicenter Survey. Published online April 9, 2024. doi:[10.21203/rs.3.rs-4212996/v1](https://doi.org/10.21203/rs.3.rs-4212996/v1)

Onyekuru NA, Ihemezie EJ, Ezea CP, Apeh CC, Onyekuru BO. Impacts of Ebola disease outbreak in West Africa: Implications for government and public health preparedness and lessons from COVID-19. *Scientific African*. 2023;19:e01513. doi:[10.1016/j.sciaf.2022.e01513](https://doi.org/10.1016/j.sciaf.2022.e01513)

Onyeneho NG, Aronu NI, Igwe I, et al. Two Obstacles in Response Efforts to the Ebola Epidemic in the Provinces of North Kivu and Ituri in the Democratic Republic of the Congo: Denial of and Rumors about the Disease. *J Immunological Sci*. 2023;S3(3):44-57. doi:[10.29245/2578-3009/2023/S3.1104](https://doi.org/10.29245/2578-3009/2023/S3.1104)

Owusu I, Adu C, Aboagye RG, et al. Preparing for future outbreaks in Ghana: An overview of current COVID-19, monkeypox, and Marburg disease outbreaks. *Health Promot Perspect*. 2023;13(3):202-211. doi:[10.34172/hpp.2023.25](https://doi.org/10.34172/hpp.2023.25)

Pare BC, Camara AM, Camara A, et al. Ebola outbreak in Guinea, 2021: Clinical care of patients with Ebola virus disease. *Southern African Journal of Infectious Diseases*. 2023;38(1). doi:[10.4102/sajid.v38i1.454](https://doi.org/10.4102/sajid.v38i1.454)

Perera SM, Garbern SC, Mbong EN, et al. Perceptions toward Ebola vaccination and correlates of vaccine uptake among high-risk community members in North Kivu, Democratic Republic of the Congo. Kampalath VN, ed. *PLOS Glob Public Health*. 2024;4(1):e0002566. doi:[10.1371/journal.pgph.0002566](https://doi.org/10.1371/journal.pgph.0002566)

Potter C, Mullen L, Ssendagire S, et al. Retrospective identification of key activities in Uganda’s preparedness measures related to the 2018–2020 EVD outbreak in eastern DRC utilizing a framework evaluation tool. Kiggundu R, ed. *PLOS Glob Public Health*. 2022;2(5):e0000428. doi:[10.1371/journal.pgph.0000428](https://doi.org/10.1371/journal.pgph.0000428)

Rai A, Khatri G. Resurgence of the Ebola Virus in the Democratic Republic of Congo: A Perspective. *Disaster med public health prep*. 2023;17:e327. doi:[10.1017/dmp.2022.309](https://doi.org/10.1017/dmp.2022.309)

Rai A, Hamiidah N, Abbass M, et al. Ebola Virus Disease in Uganda: A global emergency call. *Annals of Medicine & Surgery*. 2022;84. doi:[10.1016/j.amsu.2022.104825](https://doi.org/10.1016/j.amsu.2022.104825)

Reddy M. Flattening the curve: voluntary association participation and the 2013–16 West Africa Ebola epidemic. *Disasters*. 2023;47(2):366-388. doi:[10.1111/disa.12548](https://doi.org/10.1111/disa.12548)

Regmi K, Gilbert R, Thunhurst C. How can health systems be strengthened to control and prevent an Ebola outbreak? A narrative review. *Infection Ecology & Epidemiology*. 2015;5(1):28877. doi:[10.3402/iee.v5.28877](https://doi.org/10.3402/iee.v5.28877)

Reuben RC, Abunike SA. Marburg virus disease: the paradox of Nigeria’s preparedness and priority effects in co-epidemics. *Bull Natl Res Cent*. 2023;47(1):10. doi:[10.1186/s42269-023-00987-1](https://doi.org/10.1186/s42269-023-00987-1)

Riolexus A, Kadobera D, Kwesiga B, Kabwama SN, Bulage L. Preparing for the worst: opportunities to prevent trans-boundary disease transmission in Uganda: a case study. *Pan Afr Med J*. 2022;41. doi:[10.11604/pamj.supp.2022.41.1.31195](https://doi.org/10.11604/pamj.supp.2022.41.1.31195)

Rwagasore E, Nsekuye O, El-Khatib Z, et al. Lessons Learned from Sudan Ebola Virus Disease (SUDV) Preparedness in Rwanda: A Comprehensive Review and Way Forward. *J Epidemiol Glob Health*. 2023;13(3):528-538. doi:[10.1007/s44197-023-00133-0](https://doi.org/10.1007/s44197-023-00133-0)

Ryan CS, Belizaire MRD, Nanyunja M, et al. Sustainable strategies for Ebola virus disease outbreak preparedness in Africa: a case study on lessons learnt in countries neighbouring the Democratic Republic of the Congo. *Infect Dis Poverty*. 2022;11(1):118. doi:[10.1186/s40249-022-01040-5](https://doi.org/10.1186/s40249-022-01040-5)

Shannon, FQ, Bawo LL, Crump JA, Sharples K, Egan R, Hill PC. Evaluation of Ebola virus disease surveillance system capability to promptly detect a new outbreak in Liberia. *BMJ Glob Health*. 2023;8(8):e012369. doi:[10.1136/bmjgh-2023-012369](https://doi.org/10.1136/bmjgh-2023-012369)

Sibomana O, Kubwimana E. First‐ever Marburg virus disease outbreak in Equatorial Guinea and Tanzania: An imminent crisis in West and East Africa. *Immunity Inflam &amp; Disease*. 2023;11(8):e980. doi:[10.1002/iid3.980](https://doi.org/10.1002/iid3.980)

Sospeter SB, Udohchukwu OP, Ruaichi J, et al. Ebola outbreak in DRC and Uganda; an East African public health concern. *Health Science Reports*. 2023;6(8):e1448. doi:[10.1002/hsr2.1448](https://doi.org/10.1002/hsr2.1448)

Srivastava D, Kutikuppala LVS, Shanker P, et al. The neglected continuously emerging Marburg virus disease in Africa: A global public health threat. *Health Science Reports*. 2023;6(11):e1661. doi:[10.1002/hsr2.1661](https://doi.org/10.1002/hsr2.1661)

Stephens MT, Juniastuti, Sulistiawati, Dossen PC. The potential risk components and prevention measures of the Ebola virus disease outbreak in Liberia: An in-depth interview with the health workers and stakeholders. *Belitung Nurs J*. 2024;10(1):67-77. doi:[10.33546/bnj.3069](https://doi.org/10.33546/bnj.3069)

Sun J, Uwishema O, Kassem H, et al. Ebola virus outbreak returns to the Democratic Republic of Congo: An urgent rising concern. *Annals of Medicine & Surgery*. 2022;79. doi:[10.1016/j.amsu.2022.103958](https://doi.org/10.1016/j.amsu.2022.103958)

Tusabe F, Tahir IM, Akpa CI, et al. Lessons Learned from the Ebola Virus Disease and COVID-19 Preparedness to Respond to the Human Monkeypox Virus Outbreak in Low- and Middle-Income Countries. *IDR*. 2022;Volume 15:6279-6286. doi:[10.2147/IDR.S384348](https://doi.org/10.2147/IDR.S384348)

Umutesi G, Moon TD, Makam JK, et al. Evaluation of acute flaccid paralysis surveillance performance before and during the 2014-2015 Ebola virus disease outbreak in Guinea and Liberia. *Pan Afr Med J*. 2023;45. doi:[10.11604/pamj.2023.45.190.21480](https://doi.org/10.11604/pamj.2023.45.190.21480)

Wirsiy FS, Nkfusai CN, Bain LE. The SPIN framework to control and prevent the Marburg virus disease outbreak in Equatorial Guinea.

Zeng W, Samaha H, Yao M, et al. The cost of public health interventions to respond to the 10th Ebola outbreak in the Democratic Republic of the Congo. *BMJ Glob Health*. 2023;8(10):e012660. doi:[10.1136/bmjgh-2023-012660](https://doi.org/10.1136/bmjgh-2023-012660)

**Wrong language (n=5)**

Kovyrshina AV, Sizikova TE, Lebedev VN, et al. Vaccines to prevent Ebola virus disease: current challenges and perspectives. *Problems of Virology*. 2023;68(5):372-384. doi:[10.36233/0507-4088-193](https://doi.org/10.36233/0507-4088-193)

Furuse Y. Epidemiology of Viral Hemorrhagic Fever in Africa. *Uirusu*. 2021;71(1):11-18.

Yin Q, Liang G. Ebola virus disease: a zoonotic disease with an extremely high fatality rate. *China Tropical Medicine*. 2023;23(1).

The PLOS ONE Staff. Correction: Re-analysing Ebola spread in Sierra Leone: The importance of local social dynamics. *PLoS ONE*. 2023;18(8):e0290847. doi:[10.1371/journal.pone.0290847](https://doi.org/10.1371/journal.pone.0290847)

[The latest research findings on Ebola virus]. *Uirusu*. 2021;71(2):137-150.

**Wrong population (n=32)**

Amoako Johnson F, Sakyi B. Geospatial clustering and correlates of deaths during the Ebola outbreak in Liberia: a Bayesian geoadditive semiparametric analysis of nationally representative cross-sectional survey data. *BMJ Open*. 2022;12(6):e054095. doi:[10.1136/bmjopen-2021-054095](https://doi.org/10.1136/bmjopen-2021-054095)

Branda F, Mahal A, Maruotti A, Pierini M, Mazzoli S. The challenges of open data for future epidemic preparedness: The experience of the 2022 Ebolavirus outbreak in Uganda. *Front Pharmacol*. 2023;14:1101894. doi:[10.3389/fphar.2023.1101894](https://doi.org/10.3389/fphar.2023.1101894)

Cénat JM, Rousseau C, Dalexis RD, et al. Knowledge and misconceptions related to the Ebola Virus Disease among adults in the Democratic Republic of the Congo: The venomous snake under the table of prevention. *Public Health in Practice*. 2021;2:100178. doi:[10.1016/j.puhip.2021.100178](https://doi.org/10.1016/j.puhip.2021.100178)

Charnley GEC, Green N, Kelman I, Malembaka EB, Gaythorpe KAM. Evaluating the risk of conflict on recent Ebola outbreaks in Guinea and the Democratic Republic of the Congo. *BMC Public Health*. 2024;24(1):860. doi:[10.1186/s12889-024-18300-8](https://doi.org/10.1186/s12889-024-18300-8)

Das U, Fielding D. Higher local Ebola incidence causes lower child vaccination rates. *Sci Rep*. 2024;14(1):1382. doi:[10.1038/s41598-024-51633-3](https://doi.org/10.1038/s41598-024-51633-3)

Davidson MC, Lu S, Barrie MB, et al. A post-outbreak assessment of exposure proximity and Ebola virus disease-related stigma among community members in Kono District, Sierra Leone: A cross-sectional study. *SSM - Mental Health*. 2022;2:100064. doi:[10.1016/j.ssmmh.2022.100064](https://doi.org/10.1016/j.ssmmh.2022.100064)

Diarra T, Okeibunor J, Diallo B, et al. Therapeutic Itineraries during the Ebola Epidemic in the Democratic Republic of Congo. *J Immunological Sci*. 2023;S3(3):88-101. doi:[10.29245/2578-3009/2023/S3.1101](https://doi.org/10.29245/2578-3009/2023/S3.1101)

Diarra T, Okeibunor J, Diallo B, et al. Involvement of Civil Society Organizations and Other Community Groups in the Response to the Ebola Virus Disease Outbreak in the North Kivu and Ituri Provinces of the Democratic Republic of Congo. *J Immunological Sci*. 2023;S3(3):113-130. doi:[10.29245/2578-3009/2023/S3.1109](https://doi.org/10.29245/2578-3009/2023/S3.1109)

Douno M, Asampong E, Magassouba N, Fichet-Calvet E, Marí Sáez A. Correction: Hunting and consumption of rodents by children in the Lassa fever endemic area of Faranah, Guinea. *PLoS Negl Trop Dis*. 2023;17(1):e0011078. doi:[10.1371/journal.pntd.0011078](https://doi.org/10.1371/journal.pntd.0011078)

Eggers C, Martel L, Dismer A, et al. Implementing a DHIS2 Ebola virus disease module during the 2021 Guinea Ebola outbreak. *BMJ Glob Health*. 2022;7(5):e009240. doi:[10.1136/bmjgh-2022-009240](https://doi.org/10.1136/bmjgh-2022-009240)

Ezie KN, Takoutsing BD, Modeste D, et al. Marburg Virus Outbreak in Equatorial Guinea: Need for Speed. *Annals of Global Health*. 2024;90(1):5. doi:[10.5334/aogh.4178](https://doi.org/10.5334/aogh.4178)

Frimpong SO, Paintsil E. Community engagement in Ebola outbreaks in sub-Saharan Africa and implications for COVID-19 control: A scoping review. *International Journal of Infectious Diseases*. 2023;126:182-192. doi:[10.1016/j.ijid.2022.11.032](https://doi.org/10.1016/j.ijid.2022.11.032)

Jing SW, Zhan JW, Jue L, Min L. Global Epidemic of Ebola Virus Disease and the Importation Risk into China: An Assessment Based on the Risk Matrix Method. *Biomed Environ Sci*.

Judson SD. 1366. Re-examining the Origins of Ebola virus Emergence. *Open Forum Infectious Diseases*. 2022;9(Supplement_2):ofac492.1195. doi:[10.1093/ofid/ofac492.1195](https://doi.org/10.1093/ofid/ofac492.1195)

Juga ML, Nyabadza F, Chirove F. An Ebola virus disease model with fear and environmental transmission dynamics. *Infectious Disease Modelling*. 2021;6:545-559. doi:[10.1016/j.idm.2021.03.002](https://doi.org/10.1016/j.idm.2021.03.002)

Rugarabamu S, George J, Mbanzulu KM, Mwanyika GO, Misinzo G, Mboera LEG. Estimating Risk of Introduction of Ebola Virus Disease from the Democratic Republic of Congo to Tanzania: A Qualitative Assessment. *Epidemiologia*. 2022;3(1):68-80. doi:[10.3390/epidemiologia3010007](https://doi.org/10.3390/epidemiologia3010007)

Keita M, Polonsky JA, Ahuka-Mundeke S, et al. A community-based contact isolation strategy to reduce the spread of Ebola virus disease: an analysis of the 2018–2020 outbreak in the Democratic Republic of the Congo. *BMJ Glob Health*. 2023;8(6):e011907. doi:[10.1136/bmjgh-2023-011907](https://doi.org/10.1136/bmjgh-2023-011907)

Kim YE. Child mortality after the Ebola virus disease outbreak across Guinea, Liberia, and Sierra Leone. *International Journal of Infectious Diseases*. 2022;122:944-952. doi:[10.1016/j.ijid.2022.06.043](https://doi.org/10.1016/j.ijid.2022.06.043)

Koyuncu A, Carter RJ, Musaazi J, et al. Public perceptions of Ebola vaccines and confidence in health services to treat Ebola, malaria, and tuberculosis: Findings from a cross-sectional household survey in Uganda, 2020. Woolsey C, ed. *PLOS Glob Public Health*. 2023;3(12):e0001884. doi:[10.1371/journal.pgph.0001884](https://doi.org/10.1371/journal.pgph.0001884)

Musaazi J, Namageyo-Funa A, Carter VM, et al. Evaluation of Community Perceptions and Prevention Practices Related to Ebola Virus as Part of Outbreak Preparedness in Uganda, 2020. *Global Health*. 2022;10(3).

OKeeffe J, Takahashi E, Otshudiema JO, et al. Strengthening community-based surveillance: lessons learned from the 2018–2020 Democratic Republic of Congo (DRC) Ebola outbreak. *Confl Health*. 2023;17(1):41. doi:[10.1186/s13031-023-00536-7](https://doi.org/10.1186/s13031-023-00536-7)

Onyeneho NG, Aronu NI, Igwe I, et al. Traditional therapists in Ebola virus disease outbreak response: Lessons learned from the fight against the Ebola virus disease epidemic in North Kivu and Ituri, Democratic Republic of the Congo. *J Immunological Sci*. 2023;S3(3):102-112. doi:[10.29245/2578-3009/2023/S3.1108](https://doi.org/10.29245/2578-3009/2023/S3.1108)

Onyeneho NG, Aronu NI, Igwe I, et al. The Impact of the Ebola Virus Disease Epidemic among Women in the Provinces of North Kivu and Ituri in the Democratic Republic of the Congo. *J Immunological Sci*. 2023;S3(3):11-19. doi:[10.29245/2578-3009/2023/S3.1103](https://doi.org/10.29245/2578-3009/2023/S3.1103)

Onyeneho NG, Aronu NI, Igwe I, et al. Exploring Alternative Care Platforms for Symptomatic People in the Fight against the Ebola Virus Disease Outbreak. *J Immunological Sci*. 2023;S3(3):81-87. doi:[10.29245/2578-3009/2023/S3.1106](https://doi.org/10.29245/2578-3009/2023/S3.1106)

Onyeneho N, Okeibunor J, Igwe I, et al. Perceptions, Disease Representations, and Response Obstacles Regarding the Ebola Virus Disease Epidemic in the North Kivu and Ituri Provinces of the Democratic Republic of the Congo. *J Immunological Sci*. 2023;S3(3):69-80. doi:[10.29245/2578-3009/2023/S3.1112](https://doi.org/10.29245/2578-3009/2023/S3.1112)

Park C. Lessons learned from the World Health Organization’s late initial response to the 2014-2016 Ebola outbreak in West Africa. *J Public Health Afr*. 2022;13(1). doi:[10.4081/jphia.2022.1254](https://doi.org/10.4081/jphia.2022.1254)

Pham PN, Sharma M, Bindu KK, et al. Protective Behaviors Associated With Gender During the 2018-2020 Ebola Outbreak in Eastern Democratic Republic of the Congo. *JAMA Netw Open*. 2022;5(2):e2147462. doi:[10.1001/jamanetworkopen.2021.47462](https://doi.org/10.1001/jamanetworkopen.2021.47462)

Rupani N, Ngole ME, Lee JA, et al. Effect of Recombinant Vesicular Stomatitis Virus–Zaire Ebola Virus Vaccination on Ebola Virus Disease Illness and Death, Democratic Republic of the Congo. *Emerg Infect Dis*. 2022;28(6). doi:[10.3201/eid2806.212223](https://doi.org/10.3201/eid2806.212223)

Vivalya BMN, Vagheni MM, Kitoko GMB, et al. Developing mental health services during and in the aftermath of the Ebola virus disease outbreak in armed conflict settings: a scoping review. *Global Health*. 2022;18(1):71. doi:[10.1186/s12992-022-00862-0](https://doi.org/10.1186/s12992-022-00862-0)

Vossler H, Akilimali P, Pan Y, KhudaBukhsh WR, Kenah E, Rempała GA. Analysis of individual-level data from 2018–2020 Ebola outbreak in Democratic Republic of the Congo. *Sci Rep*. 2022;12(1):5534. doi:[10.1038/s41598-022-09564-4](https://doi.org/10.1038/s41598-022-09564-4)

Warsame A, Eamer G, Kai A, et al. Performance of a safe and dignified burial intervention during an Ebola epidemic in the eastern Democratic Republic of the Congo, 2018–2019. *BMC Med*. 2023;21(1):484. doi:[10.1186/s12916-023-03194-x](https://doi.org/10.1186/s12916-023-03194-x)

Zwick H, Asobee MS, Mitton IK, Headley J, Eagle DE. Burial workers’ perceptions of community resistance and support systems during an Ebola outbreak in the Eastern Democratic Republic of the Congo: a qualitative study. *Confl Health*. 2023;17(1):25. doi:[10.1186/s13031-023-00521-0](https://doi.org/10.1186/s13031-023-00521-0)

**Wrong study design (n=5)**

Correction: Implementing a DHIS2 Ebola virus disease module during the 2021 Guinea Ebola outbreak. *BMJ Glob Health*. 2022;7(6):e009240corr1. doi:[10.1136/bmjgh-2022-009240corr1](https://doi.org/10.1136/bmjgh-2022-009240corr1)

Correction to: Quantifying the value of viral genomics when inferring who infected whom in the 2014–16 Ebola virus outbreak in Guinea. *Virus Evolution*. 2023;9(1):vead021. doi:[10.1093/ve/vead021](https://doi.org/10.1093/ve/vead021)

Bratcher A, Hoff N, Doshi R, et al. Correction: Zoonotic risk factors associated with seroprevalence of Ebola virus GP antibodies in the absence of diagnosed Ebola virus disease in the Democratic Republic of Congo. *PLoS neglected tropical diseases*.

Kavulikirwa OK, Sikakulya FK. Recurrent Ebola outbreaks in the eastern Democratic Republic of the Congo: A wake-up call to scale up the integrated disease surveillance and response strategy. *One Health*. 2022;14:100379. doi:[10.1016/j.onehlt.2022.100379](https://doi.org/10.1016/j.onehlt.2022.100379)

Diarra T, Onyeneho N, Okeibunor J, et al. Response of Healthcare Service Providers to the Ebola Virus Disease Epidemic in the Democratic Republic of Congo’s North Kivu and Ituri Provinces. *Journal of Immunological Sciences*.:2023.

**Search #2 - Excluded Studies List**

**Abstract only (n=3)**

Kesande MS, Katwesigye E, Nanyonjo R, Namusoke M, Nabawanuka D, Baller A. Implementation of the IPC ring approach during the Uganda Sudan Ebola virus disease response at the epicentre. Antimicrobial Resistance and Infection Control.

Nanyondo J, Wailagala A, Nakato S, et al. Rapid deployment of an infection prevention and control response strategy to control the spread of Sudan Ebola virus disease in an urban setting, the Kampala metropolitan area, Uganda, 2022. Antimicrobial Resistance and Infection Control.

Wailagala A, Ainembabazi P. Establishing infection prevention and control capacity in an Ebola treatment unit within the first seven days of the 2022 Sudan virus disease outbreak in Uganda. Antimicrobial Resistance and Infection Control.

**No information on disinfection (n=11)**

Alruwili TO, Batool R, Kariri ME, et al. Strategies And Technologies To Prevent Hospital-Acquired Infections: Lessons From Sars, Ebola, And Mers In Saudi Arabia; A Systematic Review. 2023;30.

Bouba A, Helle KB, Schneider KA. Predicting the combined effects of case isolation, safe funeral practices, and contact tracing during Ebola virus disease outbreaks. Rychtář J, ed. PLoS ONE. 2023;18(1):e0276351. doi:10.1371/journal.pone.0276351

Ephraim OE, Cyrus S, Myer P, Steve A. The impact of supportive supervision of infection prevention and control practices on Ebola outbreak in Liberia. Journal of Infection Prevention. 2018;19(6):287-293. doi:10.1177/1757177418780994

Freeman AYS, Rumunu JP, Modi ZA, et al. Assessment of infection prevention and control readiness for Ebola virus and other diseases outbreaks in a humanitarian crisis setting: a cross-sectional study of health facilities in six high-risk States of South Sudan.

Kabego L, Kourouma M, Ousman K, et al. Impact of multimodal strategies including a pay for performance strategy in the improvement of infection prevention and control practices in healthcare facilities during an Ebola virus disease outbreak. BMC Infect Dis. 2023;23(1):12. doi:10.1186/s12879-022-07956-5

Ndaliko Augustin M, Kahindo Mbeva JB. Knowledge, attitudes, and behaviors of healthcare professionals at the start of an Ebola virus epidemic. Infectious Diseases Now. 2021;51(1):50-54. doi:10.1016/j.medmal.2020.04.010

Potter C, Mullen L, Ssendagire S, et al. Retrospective identification of key activities in Uganda’s preparedness measures related to the 2018–2020 EVD outbreak in eastern DRC utilizing a framework evaluation tool. Kiggundu R, ed. PLOS Glob Public Health. 2022;2(5):e0000428. doi:10.1371/journal.pgph.0000428

Rwagasore E, Nsekuye O, El-Khatib Z, et al. Lessons Learned from Sudan Ebola Virus Disease (SUDV) Preparedness in Rwanda: A Comprehensive Review and Way Forward. J Epidemiol Glob Health. 2023;13(3):528-538. doi:10.1007/s44197-023-00133-0

Stephens MT, Juniastuti, Sulistiawati, Dossen PC. The potential risk components and prevention measures of the Ebola virus disease outbreak in Liberia: An in-depth interview with the health workers and stakeholders. Belitung Nurs J. 2024;10(1):67-77. doi:10.33546/bnj.3069

Tu J, Liu F, Wang K, Mao Y, Qi Q, Zhang J. Donning and doffing of personal protective equipment for health care workers in a tertiary hospital in China: A simulation study. Journal of Occupational and Environmental Hygiene. Published online October 9, 2023:1-11. doi:10.1080/15459624.2023.2268727

Warsame A, Eamer G, Kai A, et al. Performance of a safe and dignified burial intervention during an Ebola epidemic in the eastern Democratic Republic of the Congo, 2018–2019. BMC Med. 2023;21(1):484. doi:10.1186/s12916-023-03194-x

**Not about EBOD or MARD (n=3)**

Kamara KN, Squire JS, Kanu JS, et al. Assessment of Infection Prevention and Control Measures at Points of Entry in Sierra Leone in 2021: A Cross-Sectional Study. IJERPH. 2022;19(10):5936. doi:10.3390/ijerph19105936

Lompo P, Heroes AS, Ouédraogo K, et al. Knowledge, awareness, and risk practices related to bacterial contamination of antiseptics, disinfectants, and hand hygiene products among healthcare workers in sub-saharan Africa: a cross-sectional survey in three tertiary care hospitals (Benin, Burkina Faso, and DR Congo). Antimicrob Resist Infect Control. 2024;13(1):44. doi:10.1186/s13756-024-01396-3

Njuguna C, Vandi M, Liyosi E, et al. A challenging response to a Lassa fever outbreak in a non endemic area of Sierra Leone in 2019 with export of cases to The Netherlands. International Journal of Infectious Diseases. 2022;117:295-301. doi:10.1016/j.ijid.2022.02.020

**Full-text unavailable (n=4)**

Nakato S, Kwiringira A, Nanyondo J, Wailagala A, Kesande M, Lamorde M. Approach to rapidly improve screening and hand hygiene capacity in health facilities during the 2022 Sudan Ebola Virus Disease (SUDV) outbreak in Uganda. Antimicrobial Resistance and Infection Control. 2023;12(Suppl 1:P131).

Padoveze on behalf of IPC Public Health Emergencies Working, Willet on behalf of IPC EBOD & MARD Guideline Development, Dixit D, Zarro M, Pecchia L, Baller A. Research priorities for infection prevention and control for Ebola disease and Marburg disease. Antimicrobial Resistance & Infection Control. 2023;12(Suppl 1:P328).

Willet V, Baller A, Mearns S, G. Honein‑AbouHaidar, J. Khabsa, E. Akl. Mixed methods study assessing valuation and contextual factors related to infection prevention and control measures for Ebola disease. Antimicrobial Resistance & Infection Control. 2023;12(Suppl 1:P325).

Willet V, Mirindi P, Mearns S, Barrera‑Cancedda A ‑E., Katswesigye E, Baller A. Scorecard validation assessment for infection prevention and control in health facilities during an Ebola or Marburg disease outbreak. Antimicrobial Resistance & Infection Control. 2023;12(Suppl 1:P327).

**Wrong study design (n=1)**

Willet V, Dixit D, Fisher D, et al. Summary of WHO infection prevention and control guideline for Ebola and Marburg disease: a call for evidence based practice. BMJ. Published online February 26, 2024:p2811. doi:10.1136/bmj.p2811

**Search 3 - List of Excluded Studies**

**Full text Unavailable**

Parham EHM. Hemorrhagic Fever Successfully Treated without Quinine. South Med Rec. 1881;11(2):48-50.

Nakato S, Kwiringira A, Nanyondo J, Wailagala A, Kesande M, Lamorde M. Approach to rapidly improve screening and hand hygiene capacity in health facilities during the 2022 Sudan Ebola Virus Disease (SUDV) outbreak in Uganda. Antimicrobial Resistance and Infection Control. 2023;12(Suppl 1:P131).

Bush OB. Hemorrhagic Fever. Atlanta medical and surgical journal.

**Wrong intervention (n=39)**

Adepoju P. Experts hopeful about Ebola control in Uganda. *The Lancet*. 2022;400(10359):1184. doi:[10.1016/S0140-6736(22)01924-9](https://doi.org/10.1016/S0140-6736(22)01924-9)

Al-Tammemi AB, Sallam M, Rebhi A, et al. The outbreak of Ebola virus disease in 2022: A spotlight on a re-emerging global health menace. *Narra J*. 2022;2(3):e97. doi:[10.52225/narra.v2i3.97](https://doi.org/10.52225/narra.v2i3.97)

Ali U, Naveed M, Ijaz A, Jabeen K, Mughal MS, Ul Hasan J. The Outbreak of the Ebola Virus: Sudan Strain in Uganda and its Clinical Management. *Prehosp Disaster med*. 2022;37(6):860-862. doi:[10.1017/S1049023X22002199](https://doi.org/10.1017/S1049023X22002199)

Ankunda C, Kanyesigye SM, Nakubulwa S, et al. Assessment of health care workers preparedness to epidemics: A case of Ebola virus disease preparedness in private hospitals in Kampala, Uganda. *J Infect Dev Ctries*. 2024;18(04):556-564. doi:[10.3855/jidc.17642](https://doi.org/10.3855/jidc.17642)

Barranca E. Quand la sérologie contredit le vécu de la maladie : Éthique, recherche et annonce à propos d’Ebola en Guinée: *Santé Publique*. 2023;Vol. 35(1):65-73. doi:[10.3917/spub.231.0065](https://doi.org/10.3917/spub.231.0065)

Bellizzi S, Pichierri G, Popescu C. Migrant health during public health emergencies: The Ebola crisis in Uganda. *One Health*. 2023;16:100488. doi:[10.1016/j.onehlt.2023.100488](https://doi.org/10.1016/j.onehlt.2023.100488)

Bouba A, Helle KB, Schneider KA. Predicting the combined effects of case isolation, safe funeral practices, and contact tracing during Ebola virus disease outbreaks. Rychtář J, ed. *PLoS ONE*. 2023;18(1):e0276351. doi:[10.1371/journal.pone.0276351](https://doi.org/10.1371/journal.pone.0276351)

Branda F, Mahal A, Maruotti A, Pierini M, Mazzoli S. The challenges of open data for future epidemic preparedness: The experience of the 2022 Ebolavirus outbreak in Uganda. *Frontiers in Pharmacology*. 14(1101894).

Branda F, Maruotti A. 2022 Uganda Ebola outbreak: Early descriptions and open data. *Journal of Medical Virology*. 2023;95(1):e28344. doi:[10.1002/jmv.28344](https://doi.org/10.1002/jmv.28344)

Burki T. New guidelines for treatment of Ebola virus disease. *The Lancet Infectious Diseases*. 2022;22(10):1428-1429. doi:[10.1016/S1473-3099(22)00607-7](https://doi.org/10.1016/S1473-3099(22)00607-7)

Bwire G, Sartorius B, Guerin P, Tegegne MA, Okware SI, Talisuna AO. Sudan Ebola virus (SUDV) outbreak in Uganda, 2022: lessons learnt and future priorities for sub-Saharan Africa. *BMC Med*. 2023;21(1):144. doi:[10.1186/s12916-023-02847-1](https://doi.org/10.1186/s12916-023-02847-1)

eBioMedicine. Ebola outbreak in Uganda: urgent call for better prevention and surveillance. *eBioMedicine*. 2022;85:104366. doi:[10.1016/j.ebiom.2022.104366](https://doi.org/10.1016/j.ebiom.2022.104366)

Fallah M. To beat Ebola in Uganda, fund what worked in Liberia. *Nature*. 2022;611.

Frankfurter R. Discerning Epidemic Preparedness in Sierra Leone. *Medical Anthropology*. 2021;40(8):699-702. doi:[10.1080/01459740.2021.1961250](https://doi.org/10.1080/01459740.2021.1961250)

Ibrahim SK, Ndwandwe DE, Thomas K, Sigfrid L, Norton A. Sudan virus disease outbreak in Uganda: urgent research gaps. *BMJ Glob Health*. 2022;7(12):e010982. doi:[10.1136/bmjgh-2022-010982](https://doi.org/10.1136/bmjgh-2022-010982)

Kibuuka R, Kagoya EK, Nsubuga AllanG, et al. Ebola Virus Disease: Knowledge, Attitude and Perception - The Case of Uganda. Published online September 26, 2023. doi:[10.21203/rs.3.rs-3384769/v1](https://doi.org/10.21203/rs.3.rs-3384769/v1)

Koyuncu A, Carter RJ, Musaazi J, et al. Public perceptions of Ebola vaccines and confidence in health services to treat Ebola, malaria, and tuberculosis: Findings from a cross-sectional household survey in Uganda, 2020. Woolsey C, ed. *PLOS Glob Public Health*. 2023;3(12):e0001884. doi:[10.1371/journal.pgph.0001884](https://doi.org/10.1371/journal.pgph.0001884)

Kozlov M. Ebola outbreak in Uganda: how worried are researchers? *Nature*. Published online October 7, 2022:d41586-022-03192-03198. doi:[10.1038/d41586-022-03192-8](https://doi.org/10.1038/d41586-022-03192-8)

Maltais S, Brière S, Yaya S. Comment la résilience post-Ebola en Guinée contribue à la gestion de la COVID-19 ? *Sante Publique (Vandoeuvre-les-Nancy, France)*. 34(4).

Marziano V, Guzzetta G, Longini I, Merler S. Estimates of Serial Interval and Reproduction Number of Sudan Virus, Uganda, August–November 2022. *Emerg Infect Dis*. 2023;29(7). doi:[10.3201/eid2907.221718](https://doi.org/10.3201/eid2907.221718)

Moso MA, Lim CK, Williams E, Marshall C, McCarthy J, Williamson DA. Prevention and post-exposure management of occupational exposure to Ebola virus. *The Lancet Infectious Diseases*. 2024;24(2):e93-e105. doi:[10.1016/S1473-3099(23)00376-6](https://doi.org/10.1016/S1473-3099(23)00376-6)

Musaazi J, Namageyo-Funa A, Carter VM, et al. Evaluation of Community Perceptions and Prevention Practices Related to Ebola Virus as Part of Outbreak Preparedness in Uganda, 2020. *Global Health*. 2022;10(3).

Musoke P, Bongomin F. Sudan virus disease outbreak in Uganda in 2022: the case of patient zero. *International Journal of Infectious Diseases*. 2023;128:318-320. doi:[10.1016/j.ijid.2023.01.008](https://doi.org/10.1016/j.ijid.2023.01.008)

Nakkazi E. A trial for Ebola Sudan virus in Uganda. *The Lancet Infectious Diseases*. 2023;23(2):158. doi:[10.1016/S1473-3099(23)00014-2](https://doi.org/10.1016/S1473-3099(23)00014-2)

Okamoto F, Nishikawa Y, Twalla Y, Nakayama T, Masai S. Ebola outbreak in Uganda: patient values and preferences. *The Lancet*. 2022;400(10367):1925-1926. doi:[10.1016/S0140-6736(22)02321-2](https://doi.org/10.1016/S0140-6736(22)02321-2)

Okware S. Community contribution to the control of Ebola outbreaks in Uganda, 2000-2022. *Africa Health*. Published online 2022.

Olum R, Ahaisibwe B, Atuhairwe I, et al. Readiness To Manage Ebola Virus Disease Among Emergency Healthcare Workers in Uganda: A Nationwide Multicenter Survey. Published online April 9, 2024. doi:[10.21203/rs.3.rs-4212996/v1](https://doi.org/10.21203/rs.3.rs-4212996/v1)

Potter C, Mullen L, Ssendagire S, et al. Retrospective identification of key activities in Uganda’s preparedness measures related to the 2018–2020 EVD outbreak in eastern DRC utilizing a framework evaluation tool. Kiggundu R, ed. *PLOS Glob Public Health*. 2022;2(5):e0000428. doi:[10.1371/journal.pgph.0000428](https://doi.org/10.1371/journal.pgph.0000428)

Rai A, Hamiidah N, Abbass M, et al. Ebola Virus Disease in Uganda: A global emergency call. *Annals of Medicine & Surgery*. 2022;84. doi:[10.1016/j.amsu.2022.104825](https://doi.org/10.1016/j.amsu.2022.104825)

Reardon S. Flu, MERS and Ebola - the disease outbreaks most frequently reported. *Nature*. 2023;614.

Riolexus A, Kadobera D, Kwesiga B, Kabwama SN, Bulage L. Preparing for the worst: opportunities to prevent trans-boundary disease transmission in Uganda: a case study. *Pan Afr Med J*. 2022;41. doi:[10.11604/pamj.supp.2022.41.1.31195](https://doi.org/10.11604/pamj.supp.2022.41.1.31195)

Rwagasore E, Nsekuye O, El-Khatib Z, et al. Lessons Learned from Sudan Ebola Virus Disease (SUDV) Preparedness in Rwanda: A Comprehensive Review and Way Forward. *J Epidemiol Glob Health*. 2023;13(3):528-538. doi:[10.1007/s44197-023-00133-0](https://doi.org/10.1007/s44197-023-00133-0)

Sah R, Hada V, Mohanty A, et al. Re-emergence of Sudan ebolavirus after a decade: new challenge to Ebola control. *International Journal of Surgery*. 2023;109(2):131-133. doi:[10.1097/JS9.0000000000000089](https://doi.org/10.1097/JS9.0000000000000089)

Sauer LM, Mukherjee V, on behalf of the NETEC Leadership Team. Special Pathogens Readiness in the United States: From Ebola to COVID-19 to Disease X and Beyond. *Health Security*. 2022;20(S1):S-1-S-3. doi:[10.1089/hs.2022.0072](https://doi.org/10.1089/hs.2022.0072)

Sospeter SB, Udohchukwu OP, Ruaichi J, et al. Ebola outbreak in DRC and Uganda; an East African public health concern. *Health Science Reports*. 2023;6(8):e1448. doi:[10.1002/hsr2.1448](https://doi.org/10.1002/hsr2.1448)

Tsou TP. Sudan virus disease – A quick review. *Journal of the Formosan Medical Association*. 2024;123(1):16-22. doi:[10.1016/j.jfma.2023.06.001](https://doi.org/10.1016/j.jfma.2023.06.001)

Wasswa H. Ebola: Uganda battles fresh epidemic as infections threaten to spread. *BMJ*. Published online October 7, 2022:o2420. doi:[10.1136/bmj.o2420](https://doi.org/10.1136/bmj.o2420)

Wasswa H. Ebola: Uganda plans trials of two vaccines as cases rise. *BMJ*. Published online October 28, 2022:o2589. doi:[10.1136/bmj.o2589](https://doi.org/10.1136/bmj.o2589)

WHO News Release. WHO makes new recommendations for Ebola treatments, calls for improved access. Published online 2022. <https://www.who.int/news/item/22-07-2022-who-releases-global-covid-19-vaccinationstrategy-update-to-reach-unprotected>

**Wrong language (n=2)**

Furuse Y. Epidemiology of Viral Hemorrhagic Fever in Africa. Uirusu. 2021;71(1):11-18.

Yin Q, Liang G. Ebola virus disease: a zoonotic disease with an extremely high fatality rate. Chinese Tropical Medicone. 23(1):1-9.

**Wrong Outcomes (n=1)**

Nanyondo J, Wailagala A, Nakato S, et al. Rapid deployment of an infection prevention and control response strategy to control the spread of Sudan Ebola virus disease in an urban setting, the Kampala metropolitan area, Uganda, 2022. Antimicrobial Resistance and Infection Control.

**Search 4 - List of Excluded Studies**

**Exclusion Reason: Abstract only (n=3)**

Garland J, Cuzzolina J, Dodd B, Grein J, Smith RM, Nawathe P. An innovative infection control training approach P- using immersive 360 video for special pathogens patient care in a biocontainment unit (BCU) setting at an academic medical center in Los Angeles.

Poller B, Hall S, Bailey C, et al. OP05 Visualising infection with optimised light for education and training (violet). BMJ Simulation & Technology Enhanced Learning, suppl Supplement 1. 2018;4. doi:10.1136/bmjstel-2018-heeconf.11

Cook SC, North M, Wood B, Bradburn S. PG21 In-situ simulation to ‘live’ process testing: developing and training a safe donning and doffing process for the staff of a large UK critical care unit in preparation for the COVID-19 pandemic. BMJ Simulation & Technology Enhanced Learning. 2020;6(supp 1).

**Exclusion reason: No comparator (n=3)**

Tu J, Liu F, Wang K, Mao Y, Qi Q, Zhang J. Donning and doffing of personal protective equipment for health care workers in a tertiary hospital in China: A simulation study. Journal of Occupational and Environmental Hygiene. Published online October 9, 2023:1-11. doi:10.1080/15459624.2023.2268727

Doos D, Hughes AM, Pham T, et al. Front-Line Health Care Workers’ COVID-19 Infection Contamination Risks: A Human Factors and Risk Analysis Study of Personal Protective Equipment. American Journal of Medical Quality. 2024;39(1):4-13. doi:10.1097/JMQ.0000000000000159

Hunt A, Ting J, Schweitzer D, Laakso E, Stewart I. Personal protective equipment for COVID ‐19 among healthcare workers in an emergency department: An exploratory survey of workload, thermal discomfort and symptoms of heat strain. Emerg Medicine Australasia. 2023;35(3):483-488. doi:10.1111/1742-6723.14152

**Exclusion reason: Study not in English (n=1)**

Zhang H, Kang H, Luo H, Zhu J, You J. [Practical Application of Body Mechanics Principles in the Process of Health Workers Doffing Personal Protective Equipment]. Sichuan Da Xue Xue Bao Yi Xue Ban. 2023;54(4):798-803. doi:10.12182/20230760102

**Exclusion reason: Wrong intervention (n=6)**

Schwerin MR, Portnoff L, Furlong JL, et al. Evaluation of Apparatus Used to Test Liquid through Protective Materials: Comparison of a Modified Dot-Blot Apparatus to the ASTM Penetration Cell. J Test Eval. 2020;48(1):20180350. doi:10.1520/JTE20180350

Somri M, Hochman O, Somri-Gannam L, et al. Removal of Contaminated Personal Protective Equipment With and Without Supervision. A Randomized Crossover Simulation-Based Study. Sim Healthcare. 2024;19(3):137-143. doi:10.1097/SIH.0000000000000726

Bailey C, Johnson P, Moran J, et al. Simulating the Environmental Spread of SARS-CoV-2 via Cough and the Effect of Personal Mitigations. Microorganisms. 2022;10(11):2241. doi:10.3390/microorganisms10112241

Dawson JN, Guha S, Schwerin M, Lucas A. Evaluation of Glove Performance after Decontamination. Biomedical Instrumentation & Technology. 2023;57(1):31-39. doi:10.2345/0899-8205-57.1.31

Lazar MS, Ganesh V, Naik B N, Singh A, Puri GD, Kaur S. Efficacy of remote audio‐visual system versus standard onsite buddy system to monitor the doffing of personal protective equipment during COVID‐19 pandemic: An observational study. Health Planning & Management. Published online January 2024:hpm.3754. doi:10.1002/hpm.3754

Park J. The role of base-layer cooling conditions in human error occurrences during doffing of personal protective equipment in health care. International Journal of Occupational Safety and Ergonomics. 2022;28(2):1016-1024. doi:10.1080/10803548.2020.1858611

**Exclusion reason: Wrong outcomes (n=1)**

Bolas T, Werner K, Alkenbrack S, Uribe MV, Wang M, Risko N. The economic value of personal protective equipment for healthcare workers. Christian CS, ed. PLOS Glob Public Health. 2023;3(6):e0002043. doi:10.1371/journal.pgph.0002043

**Exclusion reason: Wrong study design (n=11)**

Stephens MT, Juniastuti, Sulistiawati, Dossen PC. The potential risk components and prevention measures of the Ebola virus disease outbreak in Liberia: An in-depth interview with the health workers and stakeholders. Belitung Nurs J. 2024;10(1):67-77. doi:10.33546/bnj.3069

Matić Z, Sala MFW, Tonetto LM, et al. Understanding Experience of Patients With Highly Infectious Diseases During Extended Isolation: A Design Perspective. HERD. 2023;16(1):97-112. doi:10.1177/19375867221128916

Sowole L, Kainth R, Tuudah C, Delmonte Sen A, Price N, O’Hara G. High-consequence infectious diseases: the conception and development of a multi-disciplinary, interprofessional simulation training programme. Journal of Hospital Infection. 2024;147:87-97. doi:10.1016/j.jhin.2024.02.003

Willet V, Dixit D, Fisher D, et al. Summary of WHO infection prevention and control guideline for Ebola and Marburg disease: a call for evidence based practice. BMJ. Published online February 26, 2024:p2811. doi:10.1136/bmj.p2811

Corvetto MA, Altermatt FR, Belmar F, Escudero E. Health Care Simulation as a Training Tool for Epidemic Management: A Systematic Review. Sim Healthcare. 2023;18(6):382-391. doi:10.1097/SIH.0000000000000716

Edgar M, Selvaraj S, Lee K, Caraballo-Arias Y, Harrell M, Rodriguez-Morales A. Healthcare workers, epidemic biological risks - recommendations based on the experience with COVID-19 and Ebolavirus. Infez Med. 2022;30(2). doi:10.53854/liim-3002-2

Bai X, Gan X, Yang R, et al. The analysis of reliability and validity of a competency evaluation model for front‐line nursing staff during the outbreak of major infectious diseases. Nursing in Critical Care. 2023;28(6):976-984. doi:10.1111/nicc.12954

Ngauja R, Fofanah BD, C. Conteh on behalf of National IPC Program. Assessment of the minimum requirements for infection prevention and control (IPC) programme at the national level in Sierra Leone. Antimicrobial Resistance & Infection Control. 2023;12(Suppl 1:O26).

Willet V, Baller A, Mearns S, G. Honein‑AbouHaidar, J. Khabsa, E. Akl. Mixed methods study assessing valuation and contextual factors related to infection prevention and control measures for Ebola disease. Antimicrobial Resistance & Infection Control. 2023;12(Suppl 1:P325).

Padoveze MC, Willet V, Dunn KA, M. M, Baller A. Infection prevention and control (IPC) public health emergencies working group. Antimicrobial Resistance & Infection Control. 2023;12(Suppl 1:P372).

Padoveze on behalf of IPC Public Health Emergencies Working, Willet on behalf of IPC EBOD & MARD Guideline Development, Dixit D, Zarro M, Pecchia L, Baller A. Research priorities for infection prevention and control for Ebola disease and Marburg disease. Antimicrobial Resistance & Infection Control. 2023;12(Suppl 1:P328).

**Search 5 - List of Excluded Studies**

**Full-text unavailable (n=4)**

Lozier M, Kesande M, Kim S, et al. Access to alcohol-based hand rub and hand hygiene adherence among healthcare professionals in kabarole district, Uganda, 2018-2019.

Nakato S, Kwiringira A, Nanyondo J, Wailagala A, Kesande M, Lamorde M. Approach to rapidly improve screening and hand hygiene capacity in health facilities during the 2022 Sudan Ebola Virus Disease (SUDV) outbreak in Uganda. Antimicrobial Resistance and Infection Control. 2023;12(Suppl 1:P131).

Padoveze MC, Willet V, Dunn KA, M. M, Baller A. Infection prevention and control (IPC) public health emergencies working group. Antimicrobial Resistance & Infection Control. 2023;12(Suppl 1:P372).

Willet V, Mirindi P, Mearns S, Barrera‑Cancedda A ‑E., Katswesigye E, Baller A. Scorecard validation assessment for infection prevention and control in health facilities during an Ebola or Marburg disease outbreak. Antimicrobial Resistance & Infection Control. 2023;12(Suppl 1:P327).

**Wrong comparator (n=2)**

Dawson JN, Guha S, Schwerin M, Lucas A. Evaluation of Glove Performance after Decontamination. Biomedical Instrumentation & Technology. 2023;57(1):31-39. doi:10.2345/0899-8205-57.1.31

Wolfe MK, Wells E, Mitro B, Desmarais AM, Scheinman P, Lantagne D. Seeking Clearer Recommendations for Hand Hygiene in Communities Facing Ebola: A Randomized Trial Investigating the Impact of Six Handwashing Methods on Skin Irritation and Dermatitis. Cameron DW, ed. PLoS ONE. 2016;11(12):e0167378. doi:10.1371/journal.pone.0167378

**Wrong intervention (n=14)**

String GM, Kamal Y, Gute DM, Lantagne DS. Chlorine efficacy against bacteriophage Phi6, a surrogate for enveloped human viruses, on porous and non-porous surfaces at varying temperatures and humidity. Journal of Environmental Science and Health, Part A. 2022;57(8):685-693. doi:10.1080/10934529.2022.2101845

String GM, Kamal Y, Kelly C, Gute DM, Lantagne DS. Disinfection of Phi6, MS2, and Escherichia coli by Natural Sunlight on Healthcare Critical Surfaces. The American Journal of Tropical Medicine and Hygiene. 2023;109(1):182-190. doi:10.4269/ajtmh.22-0464

Tu J, Liu F, Wang K, Mao Y, Qi Q, Zhang J. Donning and doffing of personal protective equipment for health care workers in a tertiary hospital in China: A simulation study. Journal of Occupational and Environmental Hygiene. 2024;21(2):108-118. doi:10.1080/15459624.2023.2268727

Huang Y, Xiao S, Song D, Yuan Z. Efficacy of disinfectants for inactivation of Ebola virus in suspension by integrated cell culture coupled with real-time RT–PCR. Journal of Hospital Infection. 2022;125:67-74. doi:10.1016/j.jhin.2022.04.008

Schwerin MR, Portnoff L, Furlong JL, et al. Evaluation of Apparatus Used to Test Liquid through Protective Materials: Comparison of a Modified Dot-Blot Apparatus to the ASTM Penetration Cell. Journal of Testing and Evaluation. 2020;48(1):368-379. doi:10.1520/JTE20180350

Archer J, Mikelonis A, Wyrzykowska-Ceradini B, et al. Evaluation of disinfection methods for personal protective equipment (PPE) items for reuse during a pandemic. Oliveira SMDVLD, ed. PLoS ONE. 2023;18(7):e0287664. doi:10.1371/journal.pone.0287664

Scholte FEM, Kabra KB, Tritsch SR, et al. Exploring inactivation of SARS-CoV-2, MERS-CoV, Ebola, Lassa, and Nipah viruses on N95 and KN95 respirator material using photoactivated methylene blue to enable reuse. American Journal of Infection Control. 2022;50(8):863-870. doi:10.1016/j.ajic.2022.02.016

Zdybel S, Sosnowska A, Kowalska D, et al. Hybrid Machine Learning and Experimental Studies of Antiviral Potential of Ionic Liquids against P100, MS2, and Phi6. J Chem Inf Model. 2024;64(6):1996-2007. doi:10.1021/acs.jcim.3c02037

Kabego L, Kourouma M, Ousman K, et al. Impact of multimodal strategies including a pay for performance strategy in the improvement of infection prevention and control practices in healthcare facilities during an Ebola virus disease outbreak. BMC Infect Dis. 2023;23(1):12. doi:10.1186/s12879-022-07956-5

Ma B, Linden YS, Gundy PM, Gerba CP, Sobsey MD, Linden KG. Inactivation of Coronaviruses and Phage Phi6 from Irradiation across UVC Wavelengths. Environ Sci Technol Lett. 2021;8(5):425-430. doi:10.1021/acs.estlett.1c00178

Anderson C, Tong J, Zambrana W, B. Boehm A, K. Wolfe M. Investigating the Efficacy of Various Handwashing Methods against Enveloped and Non-Enveloped Viruses. The American Journal of Tropical Medicine and Hygiene. 2023;108(4):820-828. doi:10.4269/ajtmh.22-0287

Somri M, Hochman O, Somri-Gannam L, et al. Removal of Contaminated Personal Protective Equipment With and Without Supervision. A Randomized Crossover Simulation-Based Study. Sim Healthcare. 2024;19(3):137-143. doi:10.1097/SIH.0000000000000726

Anderson CE, Boehm AB. Sunlight Inactivation of Enveloped Viruses in Clear Water. Environ Sci Technol. 2023;57(50):21395-21404. doi:10.1021/acs.est.3c06680

Jonsdottir HR, Zysset D, Lenz N, et al. Virucidal activity of three standard chemical disinfectants against Ebola virus suspended in tripartite soil and whole blood. Sci Rep. 2023;13(1):15718. doi:10.1038/s41598-023-42376-8

**Wrong study design (n=3)**

Khou-Bouvattier C, Rogée S. Effect of virucidal components on highly pathogenic viral agents. Virologie. 2021;25(6):301-316. doi:10.1684/vir.2021.0924

Willet V, Baller A, Mearns S, G. Honein‑AbouHaidar, J. Khabsa, E. Akl. Mixed methods study assessing valuation and contextual factors related to infection prevention and control measures for Ebola disease. Antimicrobial Resistance & Infection Control. 2023;12(Suppl 1:P325).

Padoveze on behalf of IPC Public Health Emergencies Working, Willet on behalf of IPC EBOD & MARD Guideline Development, Dixit D, Zarro M, Pecchia L, Baller A. Research priorities for infection prevention and control for Ebola disease and Marburg disease. Antimicrobial Resistance & Infection Control. 2023;12(Suppl 1:P328).

**Search 6 – List of Excluded Studies**

**Wrong intervention (n=2)**

Kabego L, Kourouma M, Ousman K, et al. Impact of multimodal strategies including a pay for performance strategy in the improvement of infection prevention and control practices in healthcare facilities during an Ebola virus disease outbreak. *BMC Infect Dis*. 2023;23(1):12. doi:[10.1186/s12879-022-07956-5](https://doi.org/10.1186/s12879-022-07956-5)

Flinn J, Michalek A, Bow L, Hynes NA, Philpot D, Garibaldi BT. The Use of Temperature and Pressure Data Loggers to Validate the Steam Sterilization of Category A Clinical Waste. *Applied Biosafety*. 2022;27(2):106-115. doi:[10.1089/apb.2022.0003](https://doi.org/10.1089/apb.2022.0003)

**Commentary (n=1)**

Corse T, Thomas K, Broderick J, et al. Using Ebola as a Lens to Examine Medical Waste Sterilization: Examine Medical Waste Sterilization. *WHM*. 2015;7(4):402-411. doi:[10.1002/wmh3.164](https://doi.org/10.1002/wmh3.164)
