# Supplementary material for: Infection prevention and control measures for Ebola and Marburg disease: a series of rapid reviews
Source: BMJ Open. 2026 Jul 9;16(7):e115610. doi: 10.1136/bmjopen-2025-115610 (PMC13358256; doi:10.1136/bmjopen-2025-115610)
Supplement: online supplemental file 7 [file bmjopen-16-7-s007.docx]

**Supplementary file 7. Additional/Contextual Data Summary**

Contents

[Theme 1: Transmission/Exposure 3](#_Toc157695899)

[KQ1. Should health workers who have had EVD exposure other than high-risk be excluded versus not excluded from work? 3](#_Toc157695900)

[KQ2. Should bodies of patients deceased from Ebola or Marburg disease be disinfected versus not disinfected prior to handling/moving into a body bag? 5](#_Toc157695901)

[KQ3. Should the IPC ring approach be used versus not used to prevent and control transmission of Ebola Virus Disease (EVD) in health care facility and community settings?   8](#_Toc157695902)

[Theme 2: Personal Protective Equipment (PPE) 11](#_Toc157695903)

[KQ4. Should health workers conducting EVD related screening and triage activities wear a face shield alone versus in combination with a medical (non-structured) mask? (Contexts to consider: ETU use vs. healthcare facility; outbreak vs readiness vs. high alert scenario). 11](#_Toc157695904)

[KQ7. Should health workers conducting Ebola or Marburg virus related screening and triage activities wear a gown versus wear a coverall? (Contexts to be considered: ETU use vs. healthcare facility) 11](#_Toc157695905)

[KQ5. Should Health workers in direct contact and/or indirect contact to patients with EVD or Marburg virus disease cover head and neck skin and mucous membranes or just cover mucous membranes?  12](#_Toc157695906)

[KQ6. Should health workers providing direct care or indirect care to patients with Ebola or Virus Marburg disease and using eye protection (goggles /face shield) wear them under versus over the head and neck covering? 14](#_Toc157695907)

[KQ8. Should health workers using waterproof aprons to cover gowns or coveralls while providing direct or indirect care to patients with Ebola or Marburg virus disease, use disposable versus reusable versus biodegradable types of aprons? 15](#_Toc157695908)

[Additional PICO 1 and 2: 18](#_Toc157695909)

[Theme 3: Disinfection/Decontamination 20](#_Toc157695910)

[KQ9. Should surfaces and materials in healthcare facilities, ETUs and community settings providing care to patients with Ebola or Marburg disease be disinfected using a wiping method versus a spraying method? 20](#_Toc157695911)

[KQ10. Should health workers who have direct or indirect contact with patients who have Ebola or Marburg disease be sprayed versus not sprayed during the doffing of personal protective equipment? 22](#_Toc157695912)

[KQ11 24](#_Toc157695913)

[KQ12. Should heavily soiled linen resulting from care to patients with Ebola or Marburg in health care, ETUs or community settings be incinerated versus disinfected? 26](#_Toc157695914)

# **Theme 1: Transmission/Exposure**

# KQ1. **Should health workers who have had EVD exposure other than high-risk be excluded versus not excluded from work?**

| ETD Domain | Narrative Summary |
| --- | --- |
|  | - No studies evaluated the effect of work exclusion compared to not excluding HCWs after exposure to EVD or Marburg. Additionally, we identified limited data to support the practice of identifying health workers with low/intermediate risk of EVD infection from a checklist of EVD exposure based upon patient care activities. We noted data below that may be relevant to (1) performing a risk assessment on HCWs to ascertain “high-risk” vs. “non high-risk” EVD or Marburg exposures and (2) implementing a policy to exclude exposed health care workers from work. |
| Implementation | - Health workers had numerous risk factors for virus exposure in ETUs, other areas of the hospital, and in the community, making it difficult to ascertain where Ebola infection occurred.^1^ As such, comprehensive assessment of EVD exposure may be challenging and the sensitivity of the prescribed care activities for the detection of Ebola infection is uncertain. - An important feature of the Kikwit outbreak was that health care facility workers with jobs that in most settings do not usually involve patient contact appear to have had broader job descriptions, including patient contact.^2^ - Health workers with low/intermediate EVD exposure were active monitored and those with high-risk exposure quarantine, with considerations regarding whether all contacts accepted these measures.^3^ |
| Resources/Costs | - Health workers with EVD exposure signified basic deficiencies in implementation of and adherence to core IPC practices. Building IPC capacity will generally be of great benefit to the safety of patients and health workers.^4^ - Appropriate infection control precautions and personal protective equipment should be available.^5^ |
| Health Equity | - Health equity should be considered when considering the implementation of work exclusion, as females were the most affected in previously reported outbreaks from other African countries, including the concurrent outbreak in West Africa sub region. This may be explained by the role that the female gender plays in care giving and nursing in our society, thereby exposing them to infection.^6^ |
| Social and Legal Implications | - Recent EVD outbreaks had a huge psychological impact on both the members of affected communities and those caring for infected individuals. This suggests the necessity for relief care providers to be mentally prepared to respond to such disasters and for them to be taken care of while in the field. “When we left for Monrovia we had made our wills; I made it three times and tore it up three times and the fourth one went through. As you approach Monrovia, you pray and you pray, and as the planes arrive, you wonder what to expect.”^7^ - The WHO and International Labor Organization recommend that HWs with EVD and MVD resulting from work activities should have the right to compensation, as well as free rehabilitation and access to curative services.^8^ |
| Acceptability | - Acceptability of a risk assessment using the list of patient care activities may be important since a risk assessment may rely on self-reporting. We however could not identify any contextual data relevant to the acceptability of a risk assessment. |

# KQ2. **Should bodies of patients deceased from Ebola or Marburg disease be disinfected versus not disinfected prior to handling/moving into a body bag?**

| ETD Domain | Summary |
| --- | --- |
|  | - We did not find any studies to support the disinfection of a body before moving the body to a body bag. We collected the contextual data on the broad question of handling dead bodies with respect to Ebola and Marburg transmission. - Overall, disinfection of dead bodies may be justifiable given the transmission risk associated with handling the bodies, but we found no comparative studies to justify the intervention. The context of using disinfection of dead bodies as an intervention for reducing the transmission risk of Ebola and Marburg infection is complex. Potential harms associated with disinfecting bodies (e.g., chlorine exposure) should also be considered. Understanding this context requires more data through qualitative and quantitative research methods, especially how to position this intervention within the continuum of IPC control in hospitals, operation of burial teams and safe burial practices. |
| Implementation | - Unsafe dead body management includes direct contact with biological liquids by multiple people close to the deceased. Safer approaches include informing the head of the health area, using chlorinated water during funeral baths, wearing household gloves when touching the dead body, and reducing the number of people in contact with the body. This strategy was implemented in Western districts bordering Liberia, Guinea, and Mali and demonstrates that sensitization efforts led by well-integrated and respected community leaders can be conducive of behavior change.^9^ - The higher prevalence of Ebola virus infection in contact persons who participated in burial rituals emphasizes the importance of safe and dignified burials during Ebola outbreaks and the need to systematically interview contact persons regarding participation in burial rituals.^10^ - Public health messages promoted by community and religious leaders may have influenced safe burial behaviors during the Ebola outbreak in Sierra Leone. Nearly all respondents (3049; 86%) intended to avoid touching or washing the corpse of a family member, regardless of exposure to religious leaders’ messages (adjusted odds ratio: 0.89; 95% CI: 0.53–1.48).^11^ - Barriers include fears about how bodies are handled, lack of ability to view or participate in the burial at the cemetery, and the potential for quarantine and stigma when a family member requests collection of a body or following a burial. Facilitating factors for community acceptance may include community participation in digging the grave, as well as the possibility of participating on local burial teams, following appropriate training.^12^ - In the context of EVD in North Kivu, two-way dialogue and community consultations ensured community members understand the need for Safe Dignified Burial (SDB) and to raise awareness about the use of locally appropriate SDB. Rumors about the care of the deceased and the intentions of the burial teams were also reduced thanks to a well- managed and open process^13^ - A 2015 report from Sierra Leone noted that burial teams performed most infection prevention and control practices well, including using PPE and chlorine for disinfection.^14^ |
| Health Equity | - As study of the transmission chain in Sierra Leone found that female EVD cases generated more secondary cases than their male counterparts did.^15^ |
| Acceptability | - When community leaders, religious leaders, community members, and community health workers’ supervisors were asked which unsafe practice was the most difficult to give up, dead body management and greetings with hands were the most frequently mentioned.^9^ - Safe burial using plastic bags, lack of burial clothes, and the absence of women in the burial team were described as showing a lack of honor for the deceased. Burials were described as being more compliant to control measures when practices such as community prayer were permitted^16^ - When individuals in the infected region of Guinea in 2015 were asked about intended burial preparations for family members suspected to have died from Ebola at home, only 3% of participants reported that they would wash or touch the body, and most stated that they would accept special Ebola burial teams (89%). The majority preferred to observe corpses of deceased family members from a safe distance at burials, but this attitude varied widely by region, as well as attitudes about other alternatives to touching Ebola-affected corpses also varied by region.^17^ |
| Other | - Preparations for burial and the associated funeral rites were a major transmission risk factor among cases in the EVD outbreak in West Africa^18–20^ - While not specifically about disinfection, Nyakarahuka and colleagues noted that cleaning corpses was a significant risk factor for filovirus seropositivity among all participants (AOR = 3.1; 95% CI 1.04–9.1)^21^ - Reducing transmission from exposure to dead bodies (e.g., contact with corpses, touching of bodies at funerals) through safe burial practices have been successful.^22,23^ - Health workers were half as likely to have touched a body at a funeral compared with non-health workers.^1^ - We noted there is a high degree of variation in transmission sources and high transmission risk around the time of death, before and after. Reduced funeral attendance and faster hospitalization independently influenced local transmission intensity.^24^ - Transmissions within the community decreased to substantially low rates once isolation into community care centers was implemented. Transmission during funerals was reduced after the safe dignified burials were put in place.^15^ - Understanding community experiences during the devastating Ebola epidemic provides practical lessons for engaging similar communities in risk communication and social mobilization during future outbreaks and public health emergencies. There should be targeted social mobilization and risk communication efforts particularly around safe burial practices and personal protective actions such as hand washing. There should be targeted communication with survivors.^25^ |

# KQ3. **Should the IPC ring approach be used versus not used to prevent and control transmission of Ebola Virus Disease (EVD) in health care facility and community settings?**

| ETD Domain | Narrative Summary |
| --- | --- |
|  | - The IPC Ring approach is based upon the premise that early cluster detection can trigger a rapid, localized response in the high-risk radius around one or several health facilities to reduce transmission sufficiently to extinguish an outbreak or reduce its spread. This premise is the operating principle in case-area targeted interventions against cholera epidemics^26^ - Although IPC Ring shows promise for outbreak control in Liberia, Guinea, Sierra Leone and the Democratic Republic of Congo, it is critically dependent on IPC training, contact tracing and triage capacities^27–31^ - IPC Ring is an IPC approach that requires effectiveness evaluation. It was developed rapidly and collaboratively in response to an urgent public health need; as such, data were not collected and aggregated systematically across all facilities, potentially limiting the generalizability of these results^28^ |
| Implementation | - Dahl 2016 published a summary report of CDC’s Response to the 2014–2016 Ebola Epidemic — Guinea, Liberia, and Sierra Leone. The MoHS in Sierra Leone used CDC’s concept of Ring Infection Prevention and Control (Ring IPC), and CDC was integral to implementing the strategy. This strategy supported improved screening, isolation, referral for treatment, use of hand hygiene and personal protective equipment, waste management, and cleaning and decontamination practices for health care facilities and health care workers at highest risk for Ebola exposure and infection. CDC staff commonly coordinated Ring IPC activities in collaboration with WHO, the United Kingdom’s Department for International Development, and nongovernment organizational partners^27^ - Nyenswah 2016 noted that the IPC ring approach was an innovative strategy to strengthen IPC practices and support improved screening, isolation, referral for treatment, use of hand hygiene and personal protective equipment, waste management, and cleaning and decontamination practices for health care facilities and health care workers at highest risk for Ebola exposure and infection^32^ - Hageman 2016 noted that the strategy seemed to be acceptable and effective. For example, in Liberia, three febrile HCWs were identified when screened for work; all were properly isolated and transferred to an ETU for testing. Sierra Leone integrated Ring IPC around clusters of Ebola patients in three districts. Guinea focused on minimizing transmission by rapidly investigating infected HCWs and remediating IPC lapses^33^ - Cooper 2016 noted that Montserrado County was divided into four geographic sectors, each with its own team. Each team focused primarily on healthcare facility readiness, with an emphasis on triage. Although the national IPC Task Force continued to set priorities and establish minimum standards, the implementation and monitoring of these standards in Montserrado was delegated to sector teams. These intensified efforts, implemented in a “ring approach”, helped Liberia approach its goal of “getting to zero” after identification of the cluster of 22 EVD infections near St Paul Bridge in Monrovia in February 2015.^30^ - Keita 2018 highlighted the importance of OPC training as part of the intervention. Throughout the EVD outbreak in Guinea, individual healthcare workers (usually 1 or 2 per healthcare facility) were selected to take part in an intensive five-day IPC training with a focus on EVD, organised by the Ministry of Health and partners (WHO, CDC and others). The participants were strongly encouraged to organise cascade training, i.e. training to other medical staff within their respective healthcare structures, following guidelines developed by the Ministry of Health and as previously described.^34^ - Mobula 2020 noted that the ring IPC with supervision (IPC focal point at health facilities) and frequent evaluations (use of IPC score card) was successfully implemented during the tenth outbreak of Ebola virus disease (EVD) in North Kivu, the Democratic Republic of the Congo (DRC). A standardized package for IPC/water, sanitation, and hygiene was established to ensure a coordinated IPC strategy. Supervision (establishing an IPC focal point at health facilities) and frequent evaluations (use of an IPC score card) were put into place. Evaluations helped in developing plans to fill gaps and monitor response progress. Traditional healers and pharmacists were involved in IPC training, albeit late, as they played an important role in the spread of Ebola. Triage systems set up in health facilities helped to ensure health service continuity, allowing access to health services for regular health care.^31^ - A CDC report on controlling the Last Known Cluster of Ebola Virus Disease in Liberia, January–February 2015^29^ highlighted that the last chain of transmission was controlled due to sector-based intensified contact tracing and in-depth case investigation, widespread infection prevention and control efforts, and coordination of case investigation and contact tracing activities between Montserrado and other counties. - Lewnard 2014 concluded from their modelling analysis that accelerated case ascertainment is needed to maximize effectiveness of expanding the capacity of EVD treatment centers^35^ - Using a stochastic transmission mode modelling analysis, Yamin noted that the isolation of 75% of infected individuals in critical condition within 4 days from symptom onset has a high chance of eliminating the disease, underscoring the importance of early isolation of severely ill patients^36^ |
| Other | - Although not focused on the IPC ring intervention, Palagyi 2019 introduced a conceptual framework potentially relevant to the implementation of IPC Ring intervention. It includes six core constructs: (1) Surveillance, (2) Infrastructure and medical supplies, (3) Workforce, (4) Communication mechanisms, (5) Governance, and (6) Trust. The article reinforces the interconnectedness of the traditional health system building blocks to emerging infectious disease (EID) detection, prevention and response, and highlights the critical role of system ‘software’ (i.e. governance and trust) in enabling LMIC health systems to achieve and maintain EID preparedness.^37^ - A rapid scale-up of IPC measures is required at facilities where the risk for encountering patients with Ebola is high. The 2014–2016 West Africa EVD outbreak occurred urban areas with access to health care facilities, leading to infected patients with unrecognized Ebola initiating multiple chains of transmission at unprepared facilities. Implementation of robust IPC practices in general health care facilities was critical to ending health care–associated transmission.^38^ |

# **Theme 2: Personal Protective Equipment (PPE)**

# **KQ4. Should health workers conducting EVD related screening and triage activities wear a face shield alone versus in combination with a medical (non-structured) mask? (Contexts to consider: ETU use vs. healthcare facility; outbreak vs readiness vs. high alert scenario).**

# **KQ7. Should health workers conducting Ebola or Marburg virus related screening and triage activities wear a gown versus wear a coverall? (Contexts to be considered: ETU use vs. healthcare facility)**

| ETD Domain | Narrative Summary |
| --- | --- |
|  | With respect to the extraction of contextual data, the key findings are as follows:  (1) Basic PPE ensemble did not seem to work as well as more protected PPE ensemble^39^  (2) gowns led to less contamination than aprons^40^  (3) two pairs of gloves led to less contamination than only one pair of gloves^40^  (4) PPEs with more protective gear protected against contamination in simulation studies slightly better but felt more uncomfortable to health workers^40^  (5) The peak of contagiousness is around the time of death but patients presenting to HFs and undergoing screening/triage often do so after the onset of symptoms. They are therefore likely contagious at the time of screening and triage^41^  To protect HWs, the limited data (of very low quality) suggest that one would need to err on the side of extra protection, not less. However, the tradeoff between more protection and usability (e.g., to be able to work comfortably for longer hours with less protective PPEs) is unclear. |
| Acceptability | - Although not in a screening or triage scenario, Den Boon and colleagues noted that in frontline physicians and nurses deployed to West Africa between March and September of 2014, heat and dehydration were significant or major concern for the majority of participants surveyed. However, the authors also noted that there was no significant different between those wearing a gown or coverall.^42^ - Qualitative data collected by Hall 2018 in a simulation study, suggested that the use of a gown was more convenient and familiar. It also made unassisted doffing possible. The coverall ‘felt protective’; however, limited availability of sizes meant that they were too large for most volunteers, resulting in discomfort, excessive material restricting movement and a potential slip risk. Coveralls also require assisted doffing, thus potentially exposing an increased number of healthcare workers.^39^ |
| Other | - Two simulation studies were identified that compared use of a gown versus a coverall for heat-related outcomes using sweating thermal manikins. Both studies noted that manikins who wore ensembles with additional PPE, including a coverall, compared to ensembles with less PPE and only a gown, had worse heat-related outcomes.^43,44^ |

# KQ5. **Should Health workers in direct contact and/or indirect contact to patients with EVD or Marburg virus disease cover head and neck skin and mucous membranes or just cover mucous membranes?**

| ETD Domain | Narrative Summary |
| --- | --- |
|  | - In summary, the use of head and neck protection, such as hoods and PAPRs, is recommended in various studies and observations, as it can enhance the safety and comfort of healthcare workers when dealing with EVD or Marburg virus disease. These recommendations align with the guidance from WHO, which suggests covering the head and neck skin for healthcare workers. However, it's important to consider the practical aspects, including donning and communication issues, when selecting PPE ensembles. Additionally, further research and empirical evidence may help refine PPE recommendations in the future. |
| Implementation | - Boon 2014 noted that in survey of frontline physicians and nurses during the 2014-2016 EVD outbreak, the participants found that the use of hoods and goggles was associated with heat stress and dehydration. Hoods were perceived as low risk in terms of safety, with minimal communication and patient care impairment.^42^ - Grelot 2016 monitored thermal strain in healthcare workers during the 2014 EVD outbreak. The PPE used, which included waterproof garments covering head to toe and surgical hoods for head and neck, resulted in a moderate but safe increase in core body temperature after approximately 1 hour of wearing.^45^ - Sprecher highlighted changes in PPE recommendations made by experts in response to filovirus epidemics, including the preference for head and neck coverage using polyethylene fabric hoods. However, these changes were made with limited empirical evidence.^46^ - A relevant 2015 non-randomized simulation study by Potter and colleagues^43^ tested five levels of five PPE ensembles recommended by the WHO and MSF at two hot humid conditions. Two of the ensembles had additional head and neck covering compared to the ensembles with fewer components and no neck covering. The study found with each ensemble with more PPE equipment, there was an associated reduction in evaporative heat loss, and reduction in heat tolerance. |
| Acceptability | - Abela 2015 mentioned the preference for using PAPRs over goggles and particulate respirators (N95) based on WHO and ECDC guidelines, primarily due to the comfort and sense of protection provided by PAPRs. ^47^ - The 2014 review by Roberts and colleagues discussed the advantages and disadvantages of using PAPR versus N95 masks. PAPRs were noted to provide head and neck protection without fit testing requirements, making them suitable for healthcare workers. However, they have drawbacks, including bulkiness, noise, and the need for electricity.^48^ |
| Other | - We captured data on prior PPE recommendations by the WHO, US CDC, European CDC, and OSHA on head/neck cover. All organizations recommended that healthcare workers in direct and indirect contact with patients suffering from EVD or Marburg virus disease should cover both head and neck skin, in addition to using other essential personal protective equipment (PPE) components, to minimize the risk of exposure to these highly contagious and dangerous diseases. The type of head/neck cover varied between a surgical hood or complete coverage using a PAPR.^49–52^ |

# KQ6. **Should health workers providing direct care or indirect care to patients with Ebola or Virus Marburg disease and using eye protection (goggles /face shield) wear them under versus over the head and neck covering?**

| ETD Domain | Narrative Summary |
| --- | --- |
| Implementation | - Poller 2018 conducted a simulation study and consensus panel to identify a unified PPE ensemble for clinical response to possible high consequence infectious diseases in the United Kingdom. The consensus PPE ensemble were tested in the study; it attained no contamination events. In the ensemble, a disposable full-face visor was worn over the hood.^53^ |
| Other | - We captured data on prior recommendations by the WHO, US CDC and European CDC related to the order of donning/ doffing PPEs for eye and head/neck protection. The WHO noted that PPEs to protect mucosae should be taken off as late as possible during the PPE removal process, preferably at the end, to prevent inadvertent exposure of the mucous membranes and recommended that PPE for eye protection should be worn under a PPE for head/neck skin protection. In the procedures for donning PPE with a N95 respirator option, the US CDC recommends putting on a hood before putting on a face shield, wearing eye protection over head/neck skin protection. In the suggested steps for donning PPEs, the European CDC also recommended to don the hood before eye protection, wearing PPE for eye protection over head/neck skin protection.^49,50,52^ |

# KQ8. **Should health workers using waterproof aprons to cover gowns or coveralls while providing direct or indirect care to patients with Ebola or Marburg virus disease, use disposable versus reusable versus biodegradable types of aprons?**

| ETD Domain | Narrative Summary |
| --- | --- |
|  | - Studies suggest that when providing care to patients with Ebola or Marburg virus disease, health workers should consider using disposable aprons for better infection control. Disposable aprons are recommended by the CDC and WHO due to their lower risk of contamination and the need to minimize the spread of infectious body fluids. |
| Implementation | - A 2018 simulation study by Poller and colleagues identified the need for a wide, extra-long medium thickness plastic apron to protect the upper chest effectively. Modifying the apron by tearing the neck loop in the middle to tie it around the neck and waist was considered an acceptable way to improve protection.^53^ - Reidy 2017 noted that aprons were recommended as part of the PPE solution, with specific properties stipulated, including being disposable, fluid-repellent, plastic, and lightweight (minimum 16-mm thickness). Changing aprons and gloves between patients was recommended to reduce the risk of cross-contamination.^54^ - Fischer 2015 noted that the CDC and WHO recommend using disposable aprons when feasible, as reusable ones would require decontamination after each use. This recommendation is based on the need to minimize contamination risks.^55^ - Although focused on gowns, and not apron materials, a review by Kilinic 2015 highlighted that disposable isolation gowns are designed for single use and are typically constructed from nonwoven materials with plastic films to enhance liquid protection. Reusable gowns are made of materials like cotton or polyester and can withstand multiple laundering cycles, as suggested by the manufacturer.^56^ |
| Acceptability | - Lee 2021 conducted a survey of health workers in the US and found that fit, comfort, mobility, and donning and doffing of current PPE (including aprons) met their needs. However, it's important to note that the study focused on various aspects of PPE, not specifically on apron material types.^57^ |
| Other | - We captured data on prior PPE recommendations by the WHO, MSF and the US CDC on apron use. The WHO recommended a disposable waterproof apron, and in the case these are not available, heavy duty, reusable waterproof aprons with appropriate cleaning and disinfection between patients. It was noted that feasibility issues, such as availability of new aprons and waste disposal within isolation areas, must be addressed. Health workers wearing a reusable apron were recommended to leave the ward to clean, disinfect and remove the apron. The US CDC recommended a single-use (disposable) apron over the gown or coveralls if patients with Ebola are vomiting or have diarrhea. MSF protocol requires that when treating Ebola patients an extra protective layer (plastic apron) must be worn in order to further limit the chance of infected bodily fluids from reaching the health worker through tears or rips in the main protective suit, but does not specify if the apron is disposable, reusable, or biodegradable.^49,50,52^ - Guo 2014 conducted a simulation study with 50 participants found that plastic aprons had a higher chance of contaminating the environment compared to cotton and water-resistant gowns. Plastic aprons had a smaller covered area and were more prone to contamination.^58^ |

**Additional PICO 1 and 2:**

**Additional PICO 1: Should health workers conducting screening where at least 1m distance and a no touch technique can be maintained and PPE (such as gown, and facial protection) is not expected wear no gloves and perform hand hygiene versus wear 1 pair of gloves?**

**Additional PICO 2: Should health workers conducting screening and/or triage activities where at least 1m distance cannot be maintained and PPE (such as gown, and facial protection) is expected, wear 2 pairs of gloves versus 1 pair of gloves?**

| ETD Domain | Narrative Summary |
| --- | --- |
| Implementation | - We found very limited data reporting on infection risk among HCWs conducting triage. Dunn et al. 2016 report on two instances in Sierra Leone where patients unknowingly infected with EVD were admitted to a general hospital ward, exposing HCWs to EVD. Among those exposed, 82 contacts were identified, including 64 health care workers, 7 caregivers, 4 patients, 4 newborns, and 3 children of patients. Seven contacts became symptomatic and tested positive for EVD. Among the 82 contacts, 6 contacts took vital signs with short gloves and none of the six contacts developed EVD.^59^ - Kwon et al. 2017 conducted a prospective study of 36 HCWs (18 HCWs in each PPE ensemble) in a US hospital to evaluate HCW risk of self-contamination when donning and doffing PPE for Ebola patient care and contact precaution using fluorescence and MS2 bacteriophage. EVD PPE consisted of inner and outer gloves, boot covers, impervious gown with Velcro on the back of the neck, a powered air-purifying respirator (PAPR) and hood with face shield, and an outer apron. CP PPE consisted of a single pair of gloves and a gown. Hand hygiene and glove removal protocol deviations were common during doffing of both EVD and CP PPE (67% and 39% of HCWs made ≥1 error, respectively). Fluorescence was detected on 8 EVD PPE HCWs (44%) and 5 CP PPE HCWs (28%), most commonly on hands. MS2 was recovered from 2 EVD PPE HCWs (11%) and 3 CP PPE HCWs (17%). The authors concluded that protocol deviations were common during both EVD and CP PPE doffing and self-contamination was common.^60^ - Casanova et al 2012 compared HCW self-contamination after the simulation of doffing PPE (not performing screening or triage activities) with single gloves versus double gloves, using MS2 as a marker. Although double gloves reduced viral transfer, MS2 was still recovered from the hands of 23% of HCWs after doffing double gloves. This is compared to participants who wore single gloves, where MS2 was recovered from hands in 78% of HCWs. Hand hygiene and glove removal appear to be high-risk opportunities for HCW self-contamination.^61^ |
| Acceptability | - Honda et al. published a narrative review of advantages/disadvantages of PPE used among HCWs in high-risk settings including EVD outbreaks. Regarding double gloving, advantages mentioned were that (1) it decreases the potential risk of transmission of highly virulent pathogens through glove holes or glove damage due to using disinfectant, (2) reduces the risk of contamination of hands when removing gloves, (3) reduces the risk of needlestick injury. Disadvantages mentioned were (1) decreased tactile sensation and dexterity, and (2) a cumbersome removal process.^62^ |
| Other | The European CDC recommends that "HCWs should put on PPE for the first assessment". First assessment is performed in two steps, 1) first encounter (triage) and 2) PUI care and full history taking. Staff protection measures are applied according to the level of risk of EVD transmission for both steps. These measures include double gloves, hair cover, an impermeable gown, a surgical Type IIR face mask or FFP2 respirator, and a face shield or goggles. This implies that wearing gloves is an integral part of the recommended PPE for the first assessment, regardless of whether a minimum distance of 1 meter can be maintained or a no-touch technique is applied.^63^ For non-triage contexts, MSF also recommends two sets of gloves to be worn at all times. |

# Theme 3: Disinfection/Decontamination

# KQ9. **Should surfaces and materials in healthcare facilities, ETUs and community settings providing care to patients with Ebola or Marburg disease be disinfected using a wiping method versus a spraying method?**

| ETD Domain | Narrative Summary |
| --- | --- |
|  |  |
| Implementation | - Gallandat 2021 conducted a systematic review of chlorine-based surface disinfection efficacy to inform recommendations for low-resource outbreak settings. They noted that wiping was found to have an effect on viruses and spores even in the absence of disinfectant, indicating that the mechanical action of wiping contributes to reducing contamination levels on surfaces. Comparing wiping and spraying, both showed similar efficacies against C difficile spores, but spraying was considered less appropriate for healthcare settings due to extended drying times and ineffectiveness in removing dirt and debris.^64^ - Gallandat 2017 supported the recommendation of a 15-minute exposure to 0.5% chlorine as an effective measure to interrupt disease transmission from uncontrolled spills in Ebola outbreaks. The type of chlorine used, surface type, pre-cleaning practices, and the presence of organic matter did not significantly impact disinfection efficacy when using 0.5% chlorine.^65^ - In a multiple-thread research study, Lantagne 2018 concluded that the surface type influenced disinfection efficacy, but that chlorine type and soil load did not significantly impact disinfection efficacy when using 0.5% chlorine. The authors also noted that contact time did impact efficacy against the Ebola surrogate Φ6. Wiping or covering did not increase disinfection efficacy, but the latter could limit splashing.^66^ - Cutts 2020 and Cutts 2020 noted that different microbicides, including sodium hypochlorite, ethanol, and other active ingredients, effectively inactivate Ebola virus on stainless steel surfaces at specified concentrations and contact times.^67,68^ - Cutts 2021 found that disinfectant pre-soaked wipes with appropriate microbicidal actives effectively remove/inactivate Ebola virus, preventing unintended transfer to clean secondary surfaces when used as directed.^69^ - Poliquin 2016 found in their environmental surveillance study that the use of a 0.5% chlorine solution can preserve RNA in contaminated samples, potentially affecting the detection of viral material on surfaces.^70^ - Casey 2015 emphasized the importance of proper vehicle design and sealing to prevent contamination transfer between patient compartments and driver cabins. Pressure from chlorine sprayers may push body fluids through gaps.^71^ - Cook 2015 noted that sodium hypochlorite and ethanol were effective in decontaminating Ebola virus in a simulated organic load, but the selection of concentration and contact time is critical.^72^ |
| Other | We captured data on recommendations from WHO, US CDC, European CDC, and Médecins Sans Frontières. The 2014 guidelines from WHO advise against spraying occupied or unoccupied clinical areas with disinfectants, as this practice has no proven disease control benefit. The 2014 US CDC's considerations for chlorine use do not mention spraying as a mode for disinfectant application, suggesting a cautious approach. The European CDC recommends the application of liquid chemical disinfectants by manually wiping surfaces in a guide for general considerations for decontaminating surfaces in airplanes. MSF's recommendations note that vigorous spraying of contaminated surfaces and corpses can create aerosols, so it is advised to be cautious and wear full protective gear. They note that spraying is recommended in specific situations, such as for reusable items. For disinfecting patient excreta, urine, vomit, or blood, it is suggested to pour a 0.5% chlorine solution onto fluids on the, let it stand for 15 minutes, remove with a rag or paper towels, and discard in an infected waste bin. In summary, the consensus from these organizations is to avoid spraying as a general disinfection method due to the potential risks of creating aerosols. Manual wiping is recommended for most surfaces and materials, with the use of sprays limited to specific situations where it is deemed necessary and appropriate while taking precautions to prevent aerosol generation.^73–76^ |

# KQ10. **Should health workers who have direct or indirect contact with patients who have Ebola or Marburg disease be sprayed versus not sprayed during the doffing of personal protective equipment?**

| ETD Domain | Narrative Summary |
| --- | --- |
|  | - We identified several studies discussing EVD PPE doffing protocols. None of the doffing protocols includes a discrete step describing the practice of spraying PPEs.^54,60,77–86^ PPE can both protect and put health workers at risk for self-contamination throughout the doffing process, even among experienced HCWs doffing with a trained observer. |
| Implementation | - A 2015 cross sectional survey^87^ of HCWs, EVD survivors and contacts sprayed with chlorine found a number of negative health impacts. 64% of HCWs reported chest conditions (including coughing, difficulty breathing, chest tightness, and burning throat), and 48% had eye problems following exposure. Significant increases in eye symptoms were also reported after a single chlorine exposure. Skin irritation was highest amongst the HCWs (33.6 %), which could be attributed the use of chlorine for washing hands. Additionally, multiple chlorine exposures compared to single exposures resulted in increases in eye, chest and skin symptoms. - Reidy et al. 2017 describe the process of selecting the combination of personal protective equipment (PPE) together with donning and doffing protocols for British and Canadian military medical personnel in the Kerry Town Ebola Treatment Unit (ETU) in Sierra Leone. In the last step of the doffing protocol, the HWs step on rubber disinfection mat, scrape soles of boots on mat, step out of chlorine bath and boot-spraying area, and exit. The doffing protocol calls for repeated washing gloved hands in 0.5% chlorine; clean tap by rinsing with chlorine before turning tap off. The authors suggested that this PPE sequence minimized potential contamination of the doffing area with infectious material by reducing the requirement to spray PPE with hypochlorite.^54^ - During PPE doffing, common protocol deviations included touching outer gloves with inner-gloved hands and touching the outside of gloves with bare hands. Hand hygiene and glove removal are high-risk opportunities for health-worker self-contamination.^60,81–83,86^ - Doffing protocols need to incorporate highly effective glove and hand hygiene agents.^78^ Optimizing doffing protocols may require reinforcing careful handling of scrubs and good glove/hand hygiene with effective agents.^77^ - Hands-free alcohol based hand rub delivered directly into the HCWs’ palm keeping the dispenser uncontaminated.^88^ - In the UK, a consensus protocol calls for three layers of gloves: Inner personal protection glove (standard short non-sterile glove), middle glove (long cuffed glove), taped to gown , outer glove comprising either standard short non-sterile gloves for basic care, or heavier duty gloves for cleaning up of extreme bodily fluid episodes.^53^ |
| Other | Existing guideline recommendations regarding doffing of PPE by the WHO, US and European CDC do not involve spraying of the HCWs during doffing. Most doffing protocols identified recommend disinfection of hands with ABHR, with a hands-free dispenser method or using chlorine wipes (Reidy 2017; Cummings 2016). In the Médecins Sans Frontières 2008 Filovirus Haemorrhagic Fever (FHF) Guideline, “Dressing Protocol for Leaving the High-risk Zone (no consensus)” it was noted that HCWs should be sprayed during doffing. They recommended that “[w]hen undressing, one person should be disinfected and undress first; then the clean person should use the non-contaminated spraying machine to undress the other sprayer and the contaminated spraying machine. The undressed person should not be too close (2 meter distance and should not stand in the wind to avoid droplets during spraying). If both sprayers are dressed than they can disinfect each other.”^52,74,89^ |

# **KQ11**

**Question (11)-(a): Should health workers providing direct or indirect care to patients with Ebola or Marburg disease in ETUs and healthcare facilities wash hands (soap & water) OR wash the glove (soap & water) between patients?**

**Question (11)-(b): Should health workers providing direct or indirect care to patients with Ebola or Marburg disease in ETUs and healthcare facilities disinfect hands with ABHR OR disinfect the glove with ABHR between patients?**

**Question (11)-(c): Should health workers providing direct or indirect care to patients with Ebola or Marburg disease in ETUs and healthcare facilities disinfect hands (with chlorine) OR disinfect the glove (with chlorine) between patients?**

| ETD Domain | Narrative Summary |
| --- | --- |
|  | Although no studies met the eligibility criteria for appropriate interventions and comparators, we noted evidence that addressed hand hygiene protocols for health care workers handling highly infectious diseases in our contextual data. In summary, these studies provide valuable insights into hand hygiene methods, PPE doffing, and chlorine chemistry, emphasizing the importance of choosing appropriate methods while considering their benefits and drawbacks in different contexts. |
| Implementation | - Wolfe 2016 conducted an RCT to evaluate skin irritation caused by frequent handwashing. The study found that sanitizer had the smallest increases in skin irritation, followed by higher pH chlorine solutions, while soap and water resulted in greater irritation. This information is relevant to the choice of hand hygiene methods for healthcare workers. Sanitizer is effective but expensive, while chlorine solutions, although effective, can be challenging to produce and distribute.^90^ - Casanova 2018 and 2016 conducted studies on doffing practices and the use of hand hygiene agents. They found that structured, observed doffing using alcohol-based hand rub (ABHR) protected against hand contamination with enveloped viruses. The results emphasize the critical importance of hand hygiene after doffing, even with extensive PPE use.^78,91^ - Gao 2016 examined the effects of ABHR applications on glove integrity. The study found that multiple ABHR applications on certain gloves could be safe for PPE doffing, but changes in glove properties should be addressed through training and practice.^92^ - Lantagne 2018 conducted a comprehensive research study on chlorine chemistry, surface cleaning, and hand hygiene. The findings suggest that responders should choose the appropriate chlorine source compound for their context and ensure proper storage and testing. The study also indicates that all handwashing methods are roughly equally efficacious, but higher pH chlorine solutions are consistently safer and more efficacious, with the added benefit of reducing pathogen persistence in rinsing water. However, challenges related to explosive risks and precipitate formation should be addressed.^66^ |
| Resources/Costs | - Tantum 2021 conducted a study on hand hygiene in rural Liberian hospitals. The study highlighted challenges in the availability of water and soap, suggesting that low-cost, sustainable interventions should address supply and infrastructure-related obstacles to hospital hand hygiene improvement.^93^ |
| Acceptability | - Reidy 2017 noted that for PPE selection for UK military medical personnel working in an Ebola virus disease treatment unit in Sierra Leone, tactility and dexterity through two pairs of gloves was of key importance.^54^ |
| Other | WHO, US CDC, the European CDC and MSF all recommend wearing at least two pairs of gloves while providing patient care to maintain protection during the changing of the outer layer of gloves. The WHO noted that washing gloves with soap and water may not be practical. Both the WHO and the US CDC recommend using ABHR (Alcohol-Based Hand Rubs) for disinfecting hands and gloved hands between patients, while MSF noted that Soap, chlorine-based products and UV from sunlight all destroy the viruses. MSF also noted that chlorine solutions can weaken the second pair of gloves (surgical) and rubber household gloves.^49,52,63,74^ |

# KQ12. **Should heavily soiled linen resulting from care to patients with Ebola or Marburg in health care, ETUs or community settings be incinerated versus disinfected?**

| ETD Domain | Narrative Summary |
| --- | --- |
|  | Studies provided insights into different aspects of managing medical waste in the context of highly infectious diseases like Ebola and Marburg. While some studies mention incineration as a method of waste disposal, others focus on the importance of proper disinfection, autoclave protocols, and rigorous waste management procedures. The choice between incineration and disinfection may depend on the specific circumstances and resources available in different healthcare settings. |
| Implementation | - Cummings 2016 conducted a practice reflection, in which a multidisciplinary team from the Centers for Disease Control and Prevention (CDC) collaborated with a hospital's infection prevention team to implement occupational safety and health controls for the care of patients with Ebola Virus Disease (EVD). The focus was on managing regulated medical waste generated during patient care. The study noted that their waste management system involved incineration as the final step in the disposal of highly infectious medical waste from EVD patients.^77^ - Edmunds 2016 conducted a hazard analysis focused on the management of waste products generated from the care of individuals with Ebola Virus Disease (EVD). The study aimed to identify critical control points and transmission risks associated with different waste management activities. The authors concluded that risk can be reduced through full personal protective equipment (PPE), proper hand hygiene, and appropriate disinfectants after careful cleaning. However, it does not explicitly specify whether incineration or disinfection is recommended.^94^ - Garibaldi 2016 describes the creation of a biocontainment and treatment unit (BCU) at Johns Hopkins Medicine (JHM) for the safe care of patients with EVD. The study emphasized the use of an autoclave waste management system within the BCU to minimize the transport of infectious materials out of the unit. Infectious material is sterilized within the autoclave, and biological and chemical indicators are used to ensure proper sterilization.^95^ - Garibaldi 2017 focused on the validation of autoclave protocols for the successful decontamination of category A medical waste generated from the care of patients with serious communicable diseases, which could include Ebola. It was found that the most challenging loads to sterilize were those containing saturated linens. Factory default settings and laboratory waste guidelines were insufficient, indicating that autoclave parameters might need adjustments.^96^ - Haverkort 2016 assessed a hospital's preparations for an outbreak of viral hemorrhagic fever, such as Ebola and recommended designated, sealable waste containers for storage and the outsourcing of waste destruction to an external facility.^97^ - Otter 2010 discussed the decontamination of a critical care unit room that was contaminated with blood and body fluids while treating a patient with Lassa fever. The study employed hydrogen peroxide vapor (HPV) decontamination, which is a virucidal vapor-phase method used in healthcare settings. This method was selected due to the prolonged survival of viral hemorrhagic fever viruses on contaminated fabrics and equipment.^98^ |
| Other | MSF recommended that heavily soiled items should be soaked in 0.05% chlorine solution overnight and then burned the following day. They also recommended that clothes and bed linen of deceased patients should be buried with the corpse or they should be burned.  The 2014 WHO recommendations noted that for waste management, an incinerator may be used for short periods during an outbreak to destroy solid waste. However, it was recommended to ensure that total incineration has taken place and that caution is required when handling flammable material and when wearing gloves due to the risk of burn injuries if gloves are ignited. The US CDC noted that EVD-related waste may be inactivated through incineration or by autoclaving using properly maintained equipment with appropriate biological indicators.^73,99^ |

**References:**

1. Senga M, Pringle K, Ramsay A, et al. Factors Underlying Ebola Virus Infection Among Health Workers, Kenema, Sierra Leone, 2014–2015. *Clin Infect Dis*. 2016;63(4):454-459. doi:10.1093/cid/ciw327

2. Tomori O, Bertolli J, Rollin PE, et al. Serologic Survey among Hospital and Health Center Workers during the Ebola Hemorrhagic Fever Outbreak in Kikwit, Democratic Republic of the Congo, 1995. *J INFECT DIS*. 1999;179(s1):S98-S101. doi:10.1086/514307

3. Lópaz MA, Amela C, Ordobas M, et al. First secondary case of Ebola outside Africa: epidemiological characteristics and contact monitoring, Spain, September to November 2014. *Eurosurveillance*. 2015;20(1). doi:10.2807/1560-7917.ES2015.20.1.21003

4. Olu O, Kargbo B, Kamara S, et al. Epidemiology of Ebola virus disease transmission among health care workers in Sierra Leone, May to December 2014: a retrospective descriptive study. *BMC Infect Dis*. 2015;15(1):416. doi:10.1186/s12879-015-1166-7

5. Forrester JD, Hunter JC, Pillai SK, et al. Cluster of Ebola Cases Among Liberian and U.S. Health Care Workers in an Ebola Treatment Unit and Adjacent Hospital — Liberia, 2014. 2014;63(41):5.

6. Musa EO, Adedire E, Adeoye O, et al. Epidemiological profile of the Ebola virus disease outbreak in Nigeria, July-September 2014. *Pan Afr Med J*. 2015;21. doi:10.11604/pamj.2015.21.331.5834

7. Ngatu NR, Kayembe NJM, Phillips EK, et al. Epidemiology of ebolavirus disease (EVD) and occupational EVD in health care workers in Sub-Saharan Africa: Need for strengthened public health preparedness. *Journal of Epidemiology*. 2017;27(10):455-461. doi:10.1016/j.je.2016.09.010

8. Selvaraj SA, Lee KE, Harrell M, Ivanov I, Allegranzi B. Infection Rates and Risk Factors for Infection Among Health Workers During Ebola and Marburg Virus Outbreaks: A Systematic Review. *The Journal of Infectious Diseases*. 2018;218(suppl_5):S679-S689. doi:10.1093/infdis/jiy435

9. Gautier L, Houngbedji KA, Uwamaliya J, Coffee M. Use of a community-led prevention strategy to enhance behavioral changes towards Ebola virus disease prevention: a qualitative case study in Western Côte d’Ivoire. *glob health res policy*. 2017;2(1):35. doi:10.1186/s41256-017-0055-6

10. Diallo MSK, Rabilloud M, Ayouba A, et al. Prevalence of infection among asymptomatic and paucisymptomatic contact persons exposed to Ebola virus in Guinea: a retrospective, cross-sectional observational study. *The Lancet Infectious Diseases*. 2019;19(3):308-316. doi:10.1016/S1473-3099(18)30649-2

11. Lyons P, Winters M, Zeebari Z, et al. Engaging religious leaders to promote safe burial practices during the 2014–2016 Ebola virus disease outbreak, Sierra Leone. *Bull World Health Organ*. 2021;99(4):271-279. doi:10.2471/BLT.20.263202

12. Lee-Kwan SH, DeLuca N, Bunnell R, Clayton HB, Turay AS, Mansaray Y. Facilitators and Barriers to Community Acceptance of Safe, Dignified Medical Burials in the Context of an Ebola Epidemic, Sierra Leone, 2014. *Journal of Health Communication*. 2017;22(sup1):24-30. doi:10.1080/10810730.2016.1209601

13. Sikakulya FK, Ilumbulumbu MK, Djuma SF, Bunduki GK, Sivulyamwenge AK, Jones MK. Safe and dignified burial of a deceased from a highly contagious infectious disease ebolavirus: Socio-cultural and anthropological implications in the Eastern DR Congo. *One Health*. 2021;13:100309. doi:10.1016/j.onehlt.2021.100309

14. Nielsen CF, Kidd S, Sillah ARM, Davis E, Mermin J, Kilmarx PH. Improving Burial Practices and Cemetery Management During an Ebola Virus Disease Epidemic — Sierra Leone, 2014. 2015;64(1):8.

15. Muoghalu IS, Moses F, Conteh I, Swaray P, Ajudua A, Nordström A. The Transmission Chain Analysis of 2014–2015 Ebola Virus Disease Outbreak in Koinadugu District, Sierra Leone: An Observational Study. *Front Public Health*. 2017;5:160. doi:10.3389/fpubh.2017.00160

16. Caleo G, Duncombe J, Jephcott F, et al. The factors affecting household transmission dynamics and community compliance with Ebola control measures: a mixed-methods study in a rural village in Sierra Leone. *BMC Public Health*. 2018;18(1):248. doi:10.1186/s12889-018-5158-6

17. Jalloh MF, Robinson SJ, Corker J, et al. Knowledge, Attitudes, and Practices Related to Ebola Virus Disease at the End of a National Epidemic — Guinea, August 2015. *MMWR Morb Mortal Wkly Rep*. 2017;66(41):1109-1115. doi:10.15585/mmwr.mm6641a4

18. Namahoro J, Hogan U. A surveillance and control of Ebola Outbreak Disease at Télimélé, Guinea Conakry 2014. *Antimicrob Resist Infect Control*. 2015;4(S1):P3, 2047-2994-4-S1-P3. doi:10.1186/2047-2994-4-S1-P3

19. Miglietta A, Solimini A, Djeunang Dongho GB, et al. The Ebola virus disease outbreak in Tonkolili district, Sierra Leone: a retrospective analysis of the Viral Haemorrhagic Fever surveillance system, July 2014–June 2015. *Epidemiol Infect*. 2019;147:e103. doi:10.1017/S0950268819000177

20. Brainard J, Hooper L, Pond K, Edmunds K, Hunter PR. Risk factors for transmission of Ebola or Marburg virus disease: a systematic review and meta-analysis. *Int J Epidemiol*. 2016;45(1):102-116. doi:10.1093/ije/dyv307

21. Nyakarahuka L, Schafer IJ, Balinandi S, et al. A retrospective cohort investigation of seroprevalence of Marburg virus and ebolaviruses in two different ecological zones in Uganda. *BMC Infect Dis*. 2020;20(1):461. doi:10.1186/s12879-020-05187-0

22. Dietz PM, Jambai A, Paweska JT, Yoti Z, Ksaizek TG. Epidemiology and Risk Factors for Ebola Virus Disease in Sierra Leone—23 May 2014 to 31 January 2015. *Clin Infect Dis*. Published online July 15, 2015:civ568. doi:10.1093/cid/civ568

23. Tiffany A, Dalziel BD, Kagume Njenge H, et al. Estimating the number of secondary Ebola cases resulting from an unsafe burial and risk factors for transmission during the West Africa Ebola epidemic. Akogun OB, ed. *PLoS Negl Trop Dis*. 2017;11(6):e0005491. doi:10.1371/journal.pntd.0005491

24. International Ebola Response Team, Agua-Agum J, Ariyarajah A, et al. Exposure Patterns Driving Ebola Transmission in West Africa: A Retrospective Observational Study. von Seidlein L, ed. *PLoS Med*. 2016;13(11):e1002170. doi:10.1371/journal.pmed.1002170

25. Nuriddin A, Jalloh MF, Meyer E, et al. Trust, fear, stigma and disruptions: community perceptions and experiences during periods of low but ongoing transmission of Ebola virus disease in Sierra Leone, 2015. *BMJ Glob Health*. 2018;3(2):e000410. doi:10.1136/bmjgh-2017-000410

26. Ratnayake R, Finger F, Azman AS, et al. Highly targeted spatiotemporal interventions against cholera epidemics, 2000–19: a scoping review. *The Lancet Infectious Diseases*. 2021;21(3):e37-e48. doi:10.1016/S1473-3099(20)30479-5

27. Dahl BA, Kinzer MH, Raghunathan PL, et al. CDC’s Response to the 2014–2016 Ebola Epidemic — Guinea, Liberia, and Sierra Leone. *MMWR Suppl*. 2016;65(3):12-20. doi:10.15585/mmwr.su6503a3

28. Nyenswah T, Massaquoi M, Gbanya MZ, et al. Initiation of a Ring Approach to Infection Prevention and Control at Non-Ebola Health Care Facilities — Liberia, January–February 2015. 2015;64(18):4.

29. Nyenswah T, Fallah M, Sieh S, et al. Controlling the last known cluster of Ebola virus disease - Liberia, January-February 2015. *MMWR Morb Mortal Wkly Rep*. 2015;64(18):500-504.

30. Cooper C, Fisher D, Gupta N, MaCauley R, Pessoa-Silva CL. Infection prevention and control of the Ebola outbreak in Liberia, 2014–2015: key challenges and successes. *BMC Med*. 2016;14(1):2. doi:10.1186/s12916-015-0548-4

31. Mobula LM, Samaha H, Yao M, et al. Recommendations for the COVID-19 Response at the National Level Based on Lessons Learned from the Ebola Virus Disease Outbreak in the Democratic Republic of the Congo. *The American Journal of Tropical Medicine and Hygiene*. 2020;103(1):12-17. doi:10.4269/ajtmh.20-0256

32. Nyenswah TG, Kateh F, Bawo L, et al. Ebola and Its Control in Liberia, 2014–2015. *Emerg Infect Dis*. 2016;22(2):169-177. doi:10.3201/eid2202.151456

33. Hageman JC, Hazim C, Wilson K, et al. Infection Prevention and Control for Ebola in Health Care Settings — West Africa and United States. *MMWR Suppl*. 2016;65(3):50-56. doi:10.15585/mmwr.su6503a8

34. Keïta M, Camara AY, Traoré F, et al. Impact of infection prevention and control training on health facilities during the Ebola virus disease outbreak in Guinea. *BMC Public Health*. 2018;18(1):547. doi:10.1186/s12889-018-5444-3

35. Lewnard JA, Ndeffo Mbah ML, Alfaro-Murillo JA, et al. Dynamics and control of Ebola virus transmission in Montserrado, Liberia: a mathematical modelling analysis. *The Lancet Infectious Diseases*. 2014;14(12):1189-1195. doi:10.1016/S1473-3099(14)70995-8

36. Yamin D, Gertler S, Ndeffo-Mbah ML, et al. Effect of Ebola Progression on Transmission and Control in Liberia. *Ann Intern Med*. 2015;162(1):11-17. doi:10.7326/M14-2255

37. Palagyi A, Marais BJ, Abimbola S, Topp SM, McBryde ES, Negin J. Health system preparedness for emerging infectious diseases: A synthesis of the literature. *Global Public Health*. 2019;14(12):1847-1868. doi:10.1080/17441692.2019.1614645

38. Biedron C, Lyman M, Stuckey MJ, et al. Evaluation of Infection Prevention and Control Readiness at Frontline Health Care Facilities in High-Risk Districts Bordering Ebola Virus Disease–Affected Areas in the Democratic Republic of the Congo — Uganda, 2018. *MMWR Morb Mortal Wkly Rep*. 2019;68(39):851-854. doi:10.15585/mmwr.mm6839a4

39. Hall S, Poller B, Bailey C, et al. Use of ultraviolet-fluorescence-based simulation in evaluation of personal protective equipment worn for first assessment and care of a patient with suspected high-consequence infectious disease. *Journal of Hospital Infection*. 2018;99(2):218-228. doi:10.1016/j.jhin.2018.01.002

40. Verbeek JH, Rajamaki B, Ijaz S, et al. Personal protective equipment for preventing highly infectious diseases due to exposure to contaminated body fluids in healthcare staff. Cochrane Work Group, ed. *Cochrane Database of Systematic Reviews*. Published online April 15, 2020. doi:10.1002/14651858.CD011621.pub4

41. WHO. Ebola virus disease. https://www.who.int/health-topics/ebola#tab=tab_2

42. Den Boon S, Vallenas C, Ferri M, Norris SL. Incorporating health workers’ perspectives into a WHO guideline on personal protective equipment developed during an Ebola virus disease outbreak. *F1000Res*. 2018;7:45. doi:10.12688/f1000research.12922.2

43. Potter AW, Gonzalez JA, Xu X. Ebola Response: Modeling the Risk of Heat Stress from Personal Protective Clothing. Bouchama A, ed. *PLoS ONE*. 2015;10(11):e0143461. doi:10.1371/journal.pone.0143461

44. Coca A, DiLeo T, Kim JH, Roberge R, Shaffer R. Baseline Evaluation With a Sweating Thermal Manikin of Personal Protective Ensembles Recommended for Use in West Africa. *Disaster med public health prep*. 2015;9(5):536-542. doi:10.1017/dmp.2015.97

45. Grélot L, Koulibaly F, Maugey N, et al. Moderate Thermal Strain in Healthcare Workers Wearing Personal Protective Equipment During Treatment and Care Activities in the Context of the 2014 Ebola Virus Disease Outbreak. *J Infect Dis*. 2016;213(9):1462-1465. doi:10.1093/infdis/jiv585

46. Sprecher AG, Caluwaerts A, Draper M, et al. Personal Protective Equipment for Filovirus Epidemics: A Call for Better Evidence. *J Infect Dis*. 2015;212(suppl 2):S98-S100. doi:10.1093/infdis/jiv153

47. Abela N, Bonnici ET, Parascandalo A, Borg M. Lessons learnt and challenges in adopting the ECDC and who Ebola guidelines at Mater Dei Hospital. *Antimicrob Resist Infect Control*. 2015;4(S1):P5, 2047-2994-4-S1-P5. doi:10.1186/2047-2994-4-S1-P5

48. Roberts R. To PAPR or not to PAPR? *Can J respir ther*. 2014;50(3):87-90.

49. Personal protective equipment in the context of filovirus disease outbreak response. Published online October 2014.

50. Centers for Disease Control. Guidance on Personal Protective Equipment (PPE) in U.S. Healthcare Settings during Management of Patients Confirmed to have Selected Viral Hemorrhagic Fevers or Patients Suspected to have Selected Viral Hemorrhagic Fevers who are Clinically Unstable or Have Bleeding, Vomiting, or Diarrhea. https://www.cdc.gov/vhf/ebola/healthcare-us/ppe/guidance.html

51. Brown CK. Protecting Critical US Workers from Occupational Exposure to Emerging Infectious Diseases: Toward a Universal Personal Protective Equipment Selection Matrix for Early Outbreak Response.

52. European Centre for Disease Prevention and Contro. Safe use of personal protective equipment in the treatment of infectious diseases of high consequence.

53. Poller B, Tunbridge A, Hall S, et al. A unified personal protective equipment ensemble for clinical response to possible high consequence infectious diseases: A consensus document on behalf of the HCID programme. *Journal of Infection*. 2018;77(6):496-502. doi:10.1016/j.jinf.2018.08.016

54. Reidy P, Fletcher T, Shieber C, et al. Personal protective equipment solution for UK military medical personnel working in an Ebola virus disease treatment unit in Sierra Leone. *Journal of Hospital Infection*. 2017;96(1):42-48. doi:10.1016/j.jhin.2017.03.018

55. Fischer WA, Weber DJ, Wohl DA. Personal Protective Equipment: Protecting Health Care Providers in an Ebola Outbreak. *Clinical Therapeutics*. 2015;37(11):2402-2410. doi:10.1016/j.clinthera.2015.07.007

56. Kilinc FS. A Review of Isolation Gowns in Healthcare: Fabric and Gown Properties. *Journal of Engineered Fibers and Fabrics*. 2015;10(3):155892501501000. doi:10.1177/155892501501000313

57. Lee Y, Salahuddin M, Gibson‐Young L, Oliver GD. Assessing personal protective equipment needs for healthcare workers. *Health Science Reports*. 2021;4(3):e370. doi:10.1002/hsr2.370

58. Guo YP, Li Y, Wong PLH. Environment and body contamination: A comparison of two different removal methods in three types of personal protective clothing. *American Journal of Infection Control*. 2014;42(4):e39-e45. doi:10.1016/j.ajic.2013.12.021

59. Dunn AC, Walker TA, Redd J, et al. Nosocomial transmission of Ebola virus disease on pediatric and maternity wards: Bombali and Tonkolili, Sierra Leone, 2014. *American Journal of Infection Control*. 2016;44(3):269-272. doi:10.1016/j.ajic.2015.09.016

60. Kwon JH, Burnham CAD, Reske KA, et al. Assessment of Healthcare Worker Protocol Deviations and Self-Contamination During Personal Protective Equipment Donning and Doffing. *Infect Control Hosp Epidemiol*. 2017;38(9):1077-1083. doi:10.1017/ice.2017.121

61. Casanova LM, Rutala WA, Weber DJ, Sobsey MD. Effect of single- versus double-gloving on virus transfer to health care workers’ skin and clothing during removal of personal protective equipment. *American Journal of Infection Control*. 2012;40(4):369-374. doi:10.1016/j.ajic.2011.04.324

62. Honda H, Iwata K. Personal protective equipment and improving compliance among healthcare workers in high-risk settings: *Current Opinion in Infectious Diseases*. 2016;29(4):400-406. doi:10.1097/QCO.0000000000000280

63. Centers for Disease Control. Guidance for Confirmed Ebola Patients or Clinically Unstable PUIs. Published 2023. https://www.cdc.gov/vhf/ebola/healthcare-us/ppe/guidance.html

64. Gallandat K, Kolus RC, Julian TR, Lantagne DS. A systematic review of chlorine-based surface disinfection efficacy to inform recommendations for low-resource outbreak settings. *American Journal of Infection Control*. 2021;49(1):90-103. doi:10.1016/j.ajic.2020.05.014

65. Gallandat K, Wolfe MK, Lantagne D. Surface Cleaning and Disinfection: Efficacy Assessment of Four Chlorine Types Using Escherichia coli and the Ebola Surrogate Phi6. *Environ Sci Technol*. Published online 2017:8.

66. Lantagne D, Wolfe M, Gallandat K, Opryszko M. Determining the Efficacy, Safety and Suitability of Disinfectants to Prevent Emerging Infectious Disease Transmission. *Water*. 2018;10(10):1397. doi:10.3390/w10101397

67. Cutts TA, Robertson C, Theriault SS, et al. Efficacy of microbicides for inactivation of Ebola–Makona virus on a non-porous surface: a targeted hygiene intervention for reducing virus spread. *Sci Rep*. 2020;10(1):15247. doi:10.1038/s41598-020-71736-x

68. Cutts TA, Robertson C, Theriault SS, et al. Assessing the Contributions of Inactivation, Removal, and Transfer of Ebola Virus and Vesicular Stomatitis Virus by Disinfectant Pre-soaked Wipes. *Front Public Health*. 2020;8:183. doi:10.3389/fpubh.2020.00183

69. Cutts TA, Kasloff SB, Krishnan J, et al. Comparison of the Efficacy of Disinfectant Pre-impregnated Wipes for Decontaminating Stainless Steel Carriers Experimentally Inoculated With Ebola Virus and Vesicular Stomatitis Virus. *Front Public Health*. 2021;9:657443. doi:10.3389/fpubh.2021.657443

70. Poliquin PG, Vogt F, Kasztura M, et al. Environmental Contamination and Persistence of Ebola Virus RNA in an Ebola Treatment Center. *J Infect Dis*. 2016;214(suppl 3):S145-S152. doi:10.1093/infdis/jiw198

71. Casey ML, Nguyen DT, Idriss B, Bennett S, Dunn A, Martin S. Potential Exposure to Ebola Virus from Body Fluids due to Ambulance Compartment Permeability in Sierra Leone. *Prehosp Disaster med*. 2015;30(6):625-627. doi:10.1017/S1049023X15005294

72. Cook B, Cutts T, Nikiforuk A, et al. Evaluating Environmental Persistence and Disinfection of the Ebola Virus Makona Variant. *Viruses*. 2015;7(4):1975-1986. doi:10.3390/v7041975

73. World Health Organization. *Interim Infection Prevention and Control Guidance for Care of Patients with Suspected or Confirmed Filovirus Haemorrhagic Fever in Health-Care Settings, with Focus on Ebola*. World Health Organization; 2014. https://apps.who.int/iris/handle/10665/130596

74. Centers for Disease Control. Rationale and Considerations for Chlorine Use in Infection Control for Non- U.S. General Healthcare Settings. Published March 24, 2023. https://www.cdc.gov/vhf/ebola/clinicians/non-us-healthcare-settings/chlorine-use.html

75. ECDC Technical Report. Assessing and planning medical evacuation flights to Europe for patients with Ebola virus disease and people exposed to Ebola virus. Published October 21, 2014. https://www.ecdc.europa.eu/sites/default/files/media/en/publications/Publications/ebola-guidance-air-transport-update-decontamination.pdf

76. Centers for Disease Control. Interim Guidance for Environmental Infection Control in Hospitals for Ebola Virus. Published October 20, 2022. https://www.cdc.gov/vhf/ebola/clinicians/cleaning/hospitals.html

77. Cummings KJ, Choi MJ, Esswein EJ, et al. Addressing Infection Prevention and Control in the First U.S. Community Hospital to Care for Patients With Ebola Virus Disease: Context for National Recommendations and Future Strategies. *Ann Intern Med*. 2016;165(1):41. doi:10.7326/M15-2944

78. Casanova LM, Erukunuakpor K, Kraft CS, et al. Assessing Viral Transfer During Doffing of Ebola-Level Personal Protective Equipment in a Biocontainment Unit. *Clinical Infectious Diseases*. 2018;66(6):945-949. doi:10.1093/cid/cix956

79. Andonian J, Kazi S, Therkorn J, et al. Effect of an Intervention Package and Teamwork Training to Prevent Healthcare Personnel Self-contamination During Personal Protective Equipment Doffing. *Clinical Infectious Diseases*. 2019;69(Supplement_3):S248-S255. doi:10.1093/cid/ciz618

80. Bell T, Smoot J, Patterson J, Smalligan R, Jordan R. Ebola virus disease: The use of fluorescents as markers of contamination for personal protective equipment. *IDCases*. 2015;2(1):27-30. doi:10.1016/j.idcr.2014.12.003

81. Chughtai AA, Chen X, Macintyre CR. Risk of self-contamination during doffing of personal protective equipment. *American Journal of Infection Control*. 2018;46(12):1329-1334. doi:10.1016/j.ajic.2018.06.003

82. Lim SM, Cha WC, Chae MK, Jo IJ. Contamination during doffing of personal protective equipment by healthcare providers. *Clin Exp Emerg Med*. 2015;2(3):162-167. doi:10.15441/ceem.15.019

83. Mumma JM, Durso FT, Ferguson AN, et al. Human Factors Risk Analyses of a Doffing Protocol for Ebola-Level Personal Protective Equipment: Mapping Errors to Contamination. *Clinical Infectious Diseases*. 2018;66(6):950-958. doi:10.1093/cid/cix957

84. Mumma JM, Durso FT, Casanova LM, et al. Common Behaviors and Faults When Doffing Personal Protective Equipment for Patients With Serious Communicable Diseases. *Clinical Infectious Diseases*. 2019;69(Supplement_3):S214-S220. doi:10.1093/cid/ciz614

85. Suen LKP, Guo YP, Tong DWK, et al. Self-contamination during doffing of personal protective equipment by healthcare workers to prevent Ebola transmission. *Antimicrob Resist Infect Control*. 2018;7(1):157. doi:10.1186/s13756-018-0433-y

86. Lee M a, Huh K, Jeong J, et al. Adherence to Protocols by Healthcare Workers and Self-Contamination During Doffing of Personal Protective Equipment. *American Journal of Infection Control*. 2018;46(6):S11. doi:10.1016/j.ajic.2018.04.024

87. Mehtar S, Bulabula ANH, Nyandemoh H, Jambawai S. Deliberate exposure of humans to chlorine-the aftermath of Ebola in West Africa. *Antimicrob Resist Infect Control*. 2016;5(1):45. doi:10.1186/s13756-016-0144-1

88. McLaws ML, Chughtai AA, Salmon S, MacIntyre CR. A highly precautionary doffing sequence for health care workers after caring for wet Ebola patients to further reduce occupational acquisition of Ebola. *American Journal of Infection Control*. 2016;44(7):740-744. doi:10.1016/j.ajic.2015.12.034

89. World Health Organization. *Personal Protective Equipment for Use in a Filovirus Disease Outbreak: Rapid Advice Guideline*. World Health Organization; 2016. Accessed June 23, 2023. https://apps.who.int/iris/handle/10665/251426

90. Wolfe MK, Gallandat K, Daniels K, Desmarais AM, Scheinman P, Lantagne D. Handwashing and Ebola virus disease outbreaks: A randomized comparison of soap, hand sanitizer, and 0.05% chlorine solutions on the inactivation and removal of model organisms Phi6 and E. coli from hands and persistence in rinse water. Cameron DW, ed. *PLoS ONE*. 2017;12(2):e0172734. doi:10.1371/journal.pone.0172734

91. Casanova LM, Teal LJ, Sickbert-Bennett EE, et al. Assessment of Self-Contamination During Removal of Personal Protective Equipment for Ebola Patient Care. *Infect Control Hosp Epidemiol*. 2016;37(10):1156-1161. doi:10.1017/ice.2016.169

92. Gao P, Horvatin M, Niezgoda G, Weible R, Shaffer R. Effect of multiple alcohol-based hand rub applications on the tensile properties of thirteen brands of medical exam nitrile and latex gloves. *Journal of Occupational and Environmental Hygiene*. 2016;13(12):905-914. doi:10.1080/15459624.2016.1191640

93. Tantum LK, Gilstad JR, Bolay FK, et al. Barriers and Opportunities for Sustainable Hand Hygiene Interventions in Rural Liberian Hospitals. *IJERPH*. 2021;18(16):8588. doi:10.3390/ijerph18168588

94. Edmunds KL, Elrahman SA, Bell DJ, et al. Recommendations for dealing with waste contaminated with Ebola virus: a Hazard Analysis of Critical Control Points approach. *Bull World Health Organ*. 2016;94(6):424-432. doi:10.2471/BLT.15.163931

95. Garibaldi BT, Kelen GD, Brower RG, et al. The Creation of a Biocontainment Unit at a Tertiary Care Hospital. The Johns Hopkins Medicine Experience. *Annals ATS*. 2016;13(5):600-608. doi:10.1513/AnnalsATS.201509-587PS

96. Garibaldi BT, Reimers M, Ernst N, et al. Validation of Autoclave Protocols for Successful Decontamination of Category A Medical Waste Generated from Care of Patients with Serious Communicable Diseases. McAdam AJ, ed. *J Clin Microbiol*. 2017;55(2):545-551. doi:10.1128/JCM.02161-16

97. Haverkort JJM, Minderhoud ALC (Ben), Wind JDD, Leenen LPH, Hoepelman AIM, Ellerbroek PM. Hospital Preparations for Viral Hemorrhagic Fever Patients and Experience Gained from Admission of an Ebola Patient. *Emerg Infect Dis*. 2016;22(2):184-191. doi:10.3201/eid2202.151393

98. Otter JA, Barnicoat M, Down J, Smyth D, Yezli S, Jeanes A. Hydrogen peroxide vapour decontamination of a critical care unit room used to treat a patient with Lassa fever. *Journal of Hospital Infection*. 2010;75(4):335-337. doi:10.1016/j.jhin.2010.02.025

99. Centers for Disease Control. Handling Ebola-Associated Waste. Published 2022. https://www.cdc.gov/vhf/ebola/clinicians/cleaning/handling-waste.html
