# Supplementary material for: Infection prevention and control measures for Ebola and Marburg disease: a series of rapid reviews
Source: BMJ Open. 2026 Jul 9;16(7):e115610. doi: 10.1136/bmjopen-2025-115610 (PMC13358256; doi:10.1136/bmjopen-2025-115610)

**Supplementary file 8. Summary of Risk of Bias Assessments**

KQ5. Quality assessment summary for randomized controlled trials (ROB-2 tool)


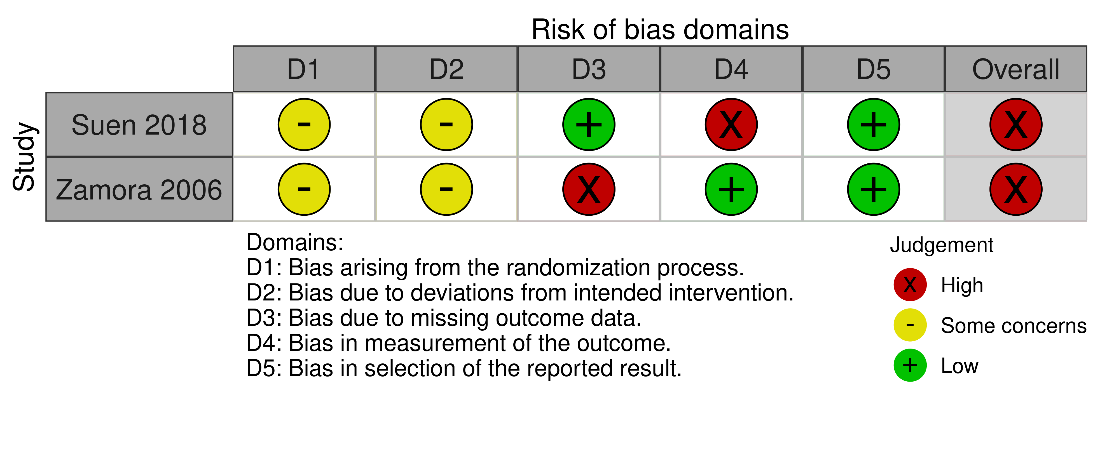


KQ5. Quality assessment summary for non-randomized studies (ROBINS-I tool)


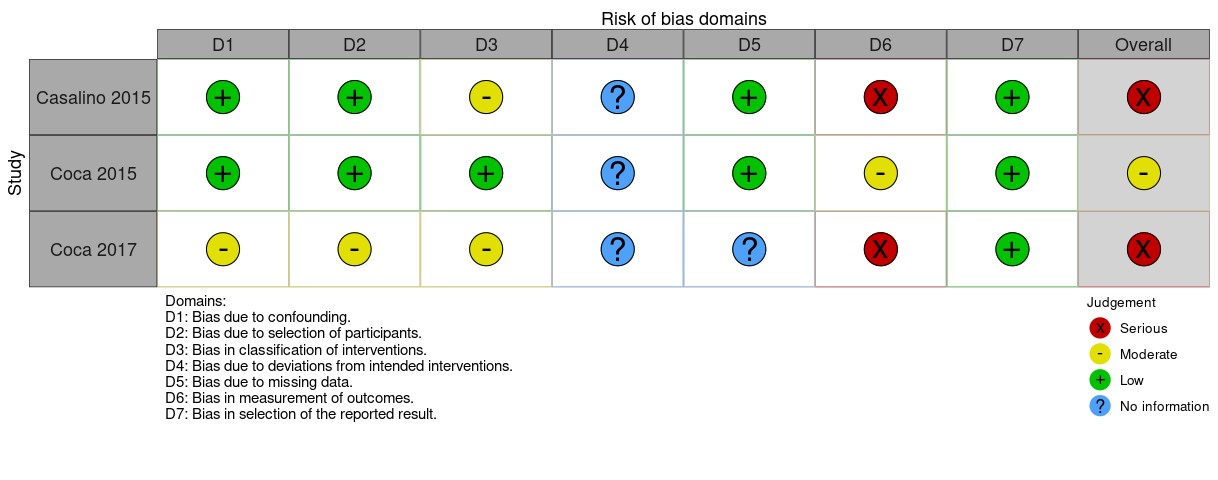


KQ6. Quality assessment summary for randomized controlled trials (ROB-2 tool)


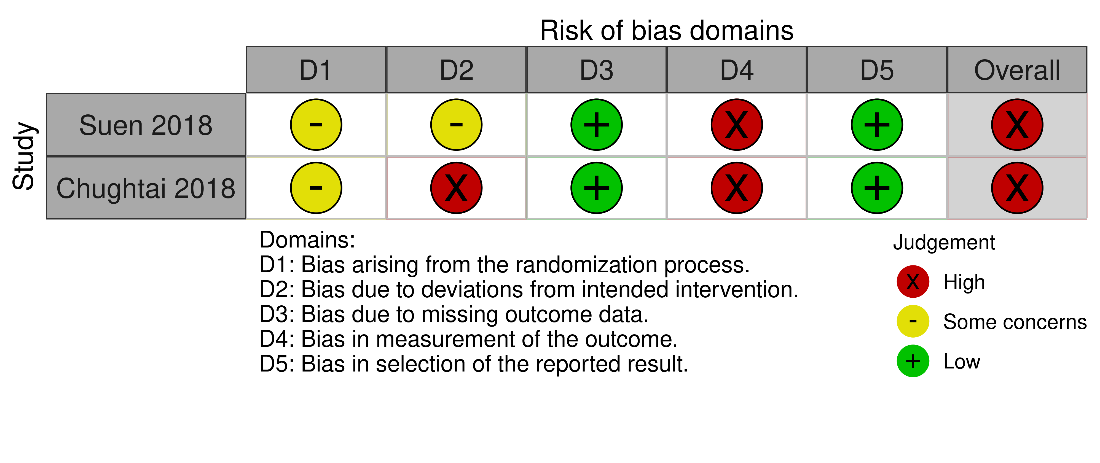


KQ10. Quality assessment summary for non-randomized studies (ROBINS-I tool)


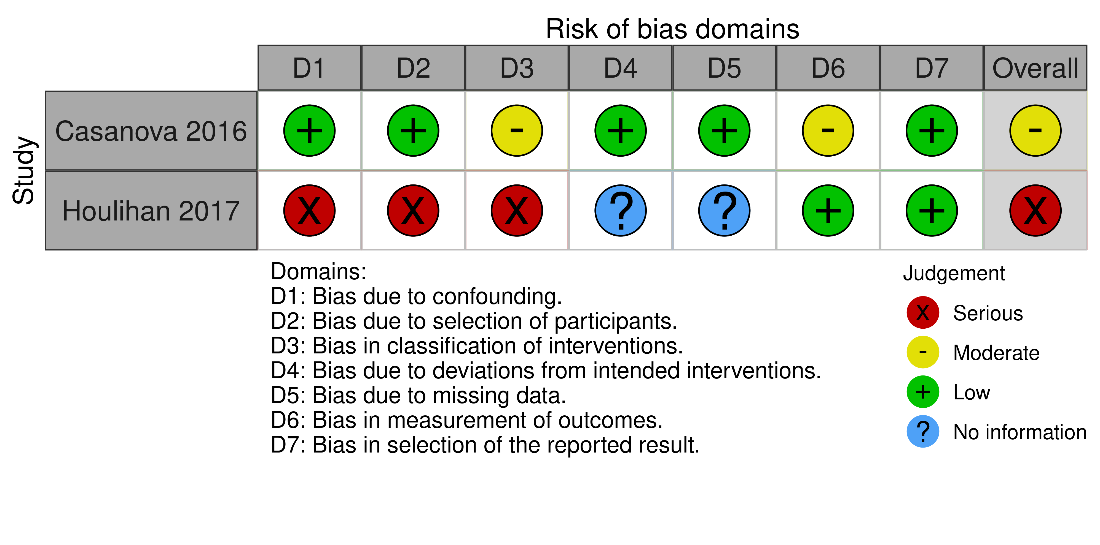

Supplement: online supplemental file 8 [file bmjopen-16-7-s008.docx]
