# Supplementary material for: Infection prevention and control measures for Ebola and Marburg disease: a series of rapid reviews
Source: BMJ Open. 2026 Jul 9;16(7):e115610. doi: 10.1136/bmjopen-2025-115610 (PMC13358256; doi:10.1136/bmjopen-2025-115610)
Supplement: online supplemental file 9 [file bmjopen-16-7-s009.docx]

**Supplementary file 9. Certainty of Evidence (GRADE) Tables**

Contents

[Supplementary File 9 – Table 1. KQ3: **Incidence of EVD** 2](#_Toc213164890)

[Supplementary File 9 - Table 2. KQ5: **Heat Tolerance** 3](#_Toc213164891)

[Supplementary File 9 - Table 3. KQ5: **Contamination during doffing of PPE** 16](#_Toc213164892)

[Supplementary File 9 - Table 4. KQ5: **Humans Errors during donning and doffing of personal protective equipment** 21](#_Toc213164893)

[Supplementary File 9 - Table 6. KQ6: **Contamination during doffing of PPE** 30](#_Toc213164894)

[Supplementary File 9 - Table 7. KQ6: **Deviation rate (%) during donning and doffing of personal protective equipment** 35](#_Toc213164895)

[Supplementary File 9 - Table 8. KQ10: **Transfer of Φ6 or MS2** 38](#_Toc213164896)

[Supplementary File 9 - Table 9. KQ10: **Infection with Ebola virus** 40](#_Toc213164897)

# Supplementary File 9 – Table 1. KQ3: **Incidence of EVD**

| **Certainty Assessment** | | | | | | | **№ of patients** | **Effect** | |  |
| --- | --- | --- | --- | --- | --- | --- | --- | --- | --- | --- |
| **Number of studies** | **Study Design** | **Risk of Bias^a^** | **Inconsistency** | **Indirectness** | **Imprecision** | **Other Considerations** | **Patients with outcome/**  **Intervention Group (IPC Ring Approach)** | **Relative**  **[Calculated 95% CI]** | **Absolute**  **Reported p-value and test**  **[Calculated 95% CI]** | **Certainty** |
| ***Incidence of EVD*** | | | | | | | | | | |
| IPC Ring Approach Intervention | | | | | | | | | | |
| 1^1^ | [Cohort] | Very serious^b^ | No serious^c^ | No serious^d^ | Serious^e^ | None | 1/166 | N/A | 6 per 1,000  p-value: NR  [95% CI: Undefined] | ⨁◯◯◯  Very low |

1. The risk of bias was assessed using a modified approach informed by established domains from the Newcastle-Ottawa Scale and JBI critical appraisal tools. Studies were evaluated for: demonstration that the outcome of interest was absent at study initiation, and completeness of outcome follow-up.
2. The risk of bias domain was downrated for lack of comparator group, no demonstration that the outcome of interest was not present at the start of study and a lack of reporting of outcome follow-up for study participants.
3. No inconsistency as only one study evaluated.
4. No serious indirectness as intervention evaluated was the IPC Ring Approach.
5. Downrated by 1 due to the small sample size and low event rate.

# Supplementary File 9 - Table 2. KQ5: **Heat Tolerance**

| **Certainty assessment** | | | | | | | **№ of patients** | | **Effect** | | **Certainty** | **Importance** |
| --- | --- | --- | --- | --- | --- | --- | --- | --- | --- | --- | --- | --- |
| **№ of studies** | **Study design** | **Risk of bias** | **Inconsistency** | **Indirectness** | **Imprecision** | **Other considerations** | **A cover for the head and neck** | **No cover for the head and neck** | **Relative**  **[Calculated 95% CI]** | **Absolute**  **Reported p-value and test**  **[Calculated 95% CI]** |  |  |
| **Time (min) to reach critical core temperature of 39°C under condition A** | | | | | | | | | | | | |
| 1 | observational studies | not serious^a^ | not serious | very serious^b^ | serious^c^ | none | 3 | 3 | - | MD 16 min fewer LSD test: P = 0.04 [95% CI: 30.78 fewer to 1.22 fewer] | ⨁◯◯◯ Very low |  |
|  |  |  |  |  |  |  |  |  | - | MD 13 min fewer LSD test: P=0.04 [95% CI: 25.2 fewer to 0.79 fewer] |  |  |
|  |  |  |  |  |  |  |  |  | - | MD 18 min fewer LSD test: P<0.05 [95% CI: Undefined] |  |  |
|  |  |  |  |  |  |  |  |  | - | MD 15 min fewer LSD test: P<0.05 [95% CI: Undefined] |  |  |
| **Body surface skin temperature (°C) at time to reach critical core temperature of 39°C under condition A** | | | | | | | | | | | | |
| 1 | observational studies | not serious^a^ | not serious | very serious^b^ | serious^c^ | none | 3 | 3 | - | MD 0.7 C higher LSD test: P>0.05 [95% CI: 0.62 lower to 2.02 higher] | ⨁◯◯◯ Very low |  |
|  |  |  |  |  |  |  |  |  | - | MD 0.6 C higher LSD test: P>0.05 [95% CI: 0.14 higher to 1.05 higher] |  |  |
|  |  |  |  |  |  |  |  |  | - | MD 1.1 C higher LSD test: P <0.05 [95% CI: 0.27 lower to 2.47 higher] |  |  |
|  |  |  |  |  |  |  |  |  | - | MD 1 C higher LSD test: P <0.05 [95% CI: 0.42 higher to 1.58 higher] |  |  |
| **Heat sensation at time to reach critical core temperature of 39°C under condition A** | | | | | | | | | | | | |
| 1 | observational studies | not serious^a^ | not serious | very serious^b^ | serious^c^ | none | 3 | 3 | - | MD 0.3 C higher LSD test: P>0.05 [95% CI: 0.06 lower to 0.66 higher] | ⨁◯◯◯ Very low |  |
|  |  |  |  |  |  |  |  |  | - | MD 0.2 C higher LSD test: P>0.05 [95% CI: 0.16 lower to 0.56 higher] |  |  |
|  |  |  |  |  |  |  |  |  | - | MD 0.2 C higher LSD test: P>0.05 [95% CI: 0.16 lower to 0.56 higher] |  |  |
|  |  |  |  |  |  |  |  |  | - | MD 0.1 C higher LSD test: P>0.05 [95% CI: 0.26 lower to 0.46 higher] |  |  |
| **Discomfort at time to reach critical core temperature of 39°C under condition A** | | | | | | | | | | | | |
| 1 | observational studies | not serious^a^ | not serious | very serious^b^ | serious^c^ | none | 3 | 3 | - | MD 0.2 C lower LSD test: P>0.05 [95% CI: 0.43 lower to 0.03 higher] | ⨁◯◯◯ Very low |  |
|  |  |  |  |  |  |  |  |  | - | MD 0.2 C lower LSD test: P>0.05 [95% CI: 0.43 lower to 0.03 higher] |  |  |
|  |  |  |  |  |  |  |  |  | - | MD 0.2 C lower LSD test: P>0.05 [95% CI: 0.43 lower to 0.03 higher] |  |  |
|  |  |  |  |  |  |  |  |  | - | MD 0.2 C lower LSD test: P>0.05 [95% CI: 0.43 lower to 0.03 higher] |  |  |
| **Core temperature (°C) after 80 minutes of activity under condition B** | | | | | | | | | | | | |
| 1 | observational studies | not serious^a^ | not serious | very serious^b^ | serious^c^ | none | 3 | 3 | - | MD 0.57 C higher LSD test: P <0.05 [95% CI: 0.21 higher to 0.93 higher] | ⨁◯◯◯ Very low |  |
|  |  |  |  |  |  |  |  |  | - | MD 0.37 C higher LSD test: P <0.05 [95% CI: 0.14 higher to 0.60 higher] |  |  |
|  |  |  |  |  |  |  |  |  | - | MD 0.85 C higher LSD test: P <0.05 [95% CI: 0.49 higher to 1.2 higher] |  |  |
|  |  |  |  |  |  |  |  |  | - | MD 0.65 C higher LSD test: P <0.05 [95% CI: 0.42 higher to 0.87 higher] |  |  |
| **Body surface skin temperature (°C) after 80 minutes of activity under condition B** | | | | | | | | | | | | |
| 1 | observational studies | not serious^a^ | not serious | very serious^b^ | serious^c^ | none | 3 | 3 | - | MD 1.2 C higher LSD test: P <0.05 [95% CI: 0.29 higher to 2.1 higher] | ⨁◯◯◯ Very low |  |
|  |  |  |  |  |  |  |  |  | - | MD 0.5 C higher LSD test: P>0.05 [95% CI: 0.22 lower to 1.22 higher] |  |  |
|  |  |  |  |  |  |  |  |  | - | MD 1.8 C higher LSD test: P <0.05 [95% CI: 0.64 higher to 2.96 higher] |  |  |
|  |  |  |  |  |  |  |  |  | - | MD 1.1 C higher LSD test: P>0.05 [95% CI: 0.09 higher to 2.11 higher] |  |  |
| **Heat sensation after 80 minutes of activity under condition B** | | | | | | | | | | | | |
| 1 | observational studies | not serious^a^ | not serious | very serious^b^ | serious^c^ | none | 3 | 3 | - | MD 0.7 higher LSD test: P>0.05 [95% CI: 0.66 lower to 2.06 higher] | ⨁◯◯◯ Very low |  |
|  |  |  |  |  |  |  |  |  | - | MD 0 LSD test: P>0.05 [95% CI: 1.16 lower to 1.16 higher] |  |  |
|  |  |  |  |  |  |  |  |  | - | MD 0.8 higher LSD test: P <0.05 [95% CI: 0.45 lower to 2.05 higher] |  |  |
|  |  |  |  |  |  |  |  |  | - | MD 0.1 higher LSD test: P>0.05 [95% CI: 0.93 lower to 1.13 higher] |  |  |
| **Discomfort after 80 minutes of activity under condition B** | | | | | | | | | | | | |
| 1 | observational studies | not serious^a^ | not serious | very serious^b^ | serious^c^ | none | 3 | 3 | - | MD 0.6 lower LSD test: P <0.05 [95% CI: 1.32 lower to 0.12 higher] | ⨁◯◯◯ Very low |  |
|  |  |  |  |  |  |  |  |  | - | MD 0.4 lower LSD test: P>0.05 [95% CI: 1.12 lower to 0.32 higher] |  |  |
|  |  |  |  |  |  |  |  |  | - | MD 0.9 lower LSD test: P <0.05 [95% CI: 1.48 lower to 0.32 lower] |  |  |
|  |  |  |  |  |  |  |  |  | - | MD 0.7 lower LSD test: P>0.05 [95% CI: 1.28 lower to 0.12 lower] |  |  |
| **Core Temperature (°C) at end of exercise** | | | | | | | | | | | | |
| 1 | observational studies | serious^d^ | not serious | very serious^b^ | serious^c^ | none | 3 | 3 | - | MD 0.73 C higher LSD test: P <0.05 [95% CI: 0.14 lower to 1.6 higher] | ⨁◯◯◯ Very low |  |
|  |  |  |  |  |  |  |  |  | - | MD 0.6 C higher LSD test: P <0.05 [95% CI: 0.34 lower to 1.54 higher] |  |  |
| **Skin Temperature (°C) at end of exercise** | | | | | | | | | | | | |
| 1 | observational studies | serious^d^ | not serious | very serious^b^ | serious^c^ | none | 3 | 3 | - | MD 1.8 C higher LSD test: P>0.05 [95% CI: 0.75 higher to 2.89 higher] | ⨁◯◯◯ Very low |  |
|  |  |  |  |  |  |  |  |  | - | MD 1.09 C higher LSD test: P>0.05 [95% CI: 0 to 2.19 higher] |  |  |
| **Heart Rate (beats per minute) at end of exercise** | | | | | | | | | | | | |
| 1 | observational studies | serious^d^ | not serious | very serious^b^ | serious^c^ | none | 3 | 3 | - | MD 27.43 BPM higher LSD test: P <0.05 [95% CI: 9.59 lower to 64.45 higher] | ⨁◯◯◯ Very low |  |
|  |  |  |  |  |  |  |  |  | - | MD 20.43 BPM higher LSD test: P <0.05 [95% CI: 15.62 higher to 56.48 higher] |  |  |
| **Average sweat weight loss (kg) per hour** | | | | | | | | | | | | |
| 1 | observational studies | serious^d^ | not serious | very serious^b^ | serious^c^ | none | 3 | 3 | - | MD 0.54 kg higher LSD test: P < 0.001 [95% CI: 0.45 lower to 1.53 higher] | ⨁◯◯◯ Very low |  |
|  |  |  |  |  |  |  |  |  | - | MD 0.32 kg higher LSD test: P = 0.03 [95% CI: 0.74 lower to 1.38 higher] |  |  |
| **Heat Sensation at end of exercise** | | | | | | | | | | | | |
| 1 | observational studies | serious^d^ | not serious | very serious^b^ | serious^c^ | none | 3 | 3 | - | MD 0.57 higher LSD test: P <0.05 [95% CI: 0.42 lower to 1.56 higher] | ⨁◯◯◯ Very low |  |
|  |  |  |  |  |  |  |  |  | - | MD 0.57 higher LSD test: P <0.05 [95% CI: 0.42 lower to 1.56 higher] |  |  |
| **Thermal Comfort at end of exercise** | | | | | | | | | | | | |
| 1 | observational studies | serious^d^ | not serious | very serious^b^ | serious^c^ | none | 3 | 3 | - | MD 0 LSD test: P>0.05 [95% CI: 4.28 lower to 4.28 higher] | ⨁◯◯◯ Very low |  |
|  |  |  |  |  |  |  |  |  | - | MD 0.86 higher LSD test: P <0.05 [95% CI: 0.90 lower to 2.62 higher] |  |  |
| **Rated perceived exertion at end of exercise** | | | | | | | | | | | | |
| 1 | observational studies | serious^d^ | not serious | very serious^b^ | serious^c^ | none | 3 | 3 | - | MD 3.43 higher LSD test: P <0.05 [95% CI: 1.82 lower to 8.68 higher] | ⨁◯◯◯ Very low |  |
|  |  |  |  |  |  |  |  |  | - | MD 2.57 higher LSD test: P <0.05 [95% CI: 3.45 lower to 8.59 higher] |  |  |
| **Breathing comfort at end of exercise** | | | | | | | | | | | | |
| 1 | observational studies | serious^d^ | not serious | very serious^b^ | serious^c^ | none | 3 | 3 | - | MD 1.57 higher LSD test: P <0.05 [95% CI: 0.75 lower to 3.89 higher] | ⨁◯◯◯ Very low |  |
|  |  |  |  |  |  |  |  |  | - | MD 1.72 higher LSD test: P <0.05 [95% CI: 0.98 lower to 4.42 higher] |  |  |
| **Wetness at end of exercise** | | | | | | | | | | | | |
| 1 | observational studies | serious^d^ | not serious | very serious^b^ | serious^c^ | none | 3 | 3 | - | MD 0 LSD test: P>0.05 [95% CI: 0.86 lower to 0.86 higher] | ⨁◯◯◯ Very low |  |
|  |  |  |  |  |  |  |  |  | - | MD 0 LSD test: P>0.05 [95% CI: 0.86 lower to 0.86 higher] |  |  |

**CI:** confidence interval; **MD:** mean difference

#### Explanations

a. Coca et al., 2015 was judged to be at moderate risk of bias. The mannequins were treated in the quality assessment, as if the mannequin were a volunteer. There was a lack of information reported for several ROBINS-I domains, including outcome measurement. There was no outcome assessor blinding, though outcomes were less vulnerable to bias, due to simulated nature of the study.

b. Downrated due to simulation study and non-human participants, as well as other differences in evaluated PPE equipment other than just head/neck cover vs. no cover.

c. Few participants and optimal information size (OIS) threshold not met.

d. We rated Coca et al., 2017, at a high risk of bias because of no demonstration of data availability for all the study participants and lack of blinding of the outcome assessor. Outcomes like thermal comfort, heat sensation, rating of perceived exertion, breathing comfort, and wetness were subjective measures which could potentially be more vulnerable to bias.

# Supplementary File 9 - Table 3. KQ5: **Contamination during doffing of PPE**

| **Certainty assessment** | | | | | | | **№ of patients** | | **Effect** | | **Certainty** | **Importance** |
| --- | --- | --- | --- | --- | --- | --- | --- | --- | --- | --- | --- | --- |
| **№ of studies** | **Study design** | **Risk of bias** | **Inconsistency** | **Indirectness** | **Imprecision** | **Other considerations** | **A cover for the head and neck** | **No cover for the head and neck** | **Relative**  **[Calculated 95% CI]** | **Absolute**  **Reported p-value and test**  **[Calculated 95% CI]** |  |  |
| **Overall contamination during doffing of PPE: Small sized contaminated patches (< 1 cm2), median - PPE1 vs PPE3** | | | | | | | | | | | | |
| 1 | randomised trials | serious^a^ | not serious | serious^b^ | serious^c^ | none | 59 | 59 | - | Difference in median **2 lower** | ⨁◯◯◯ Very low |  |
| **Overall contamination during doffing of PPE: Small sized contaminated patches (< 1 cm2), median - PPE2 vs PPE3** | | | | | | | | | | | | |
| 1 | randomised trials | serious^a^ | not serious | serious^b^ | serious^c^ | none | 59 | 59 | - | Difference of medians **0** | ⨁◯◯◯ Very low |  |
| **Hair and head contamination during doffing of PPE: Small sized contaminated patches (< 1 cm2), median - PPE1 vs PPE3** | | | | | | | | | | | | |
| 1 | randomised trials | serious^a^ | not serious | serious^b^ | Serious^c^ | none | 59 | 59 | - | Difference of medians **1.5 lower** | ⨁◯◯◯ Very low |  |
| **Hair and head contamination during doffing of PPE: Small sized contaminated patches (< 1 cm2), median - PPE2 vs PPE3** | | | | | | | | | | | | |
| 1 | randomised trials | serious^a^ | not serious | serious^b^ | Serious^c^ | none | 59 | 59 | - | Difference of medians **0.5 lower** | ⨁◯◯◯ Very low |  |
| **Neck (anterior) contamination during doffing of PPE: Small sized contaminated patches (< 1 cm2), median** | | | | | | | | | | | | |
| 1 | randomised trials | serious^a^ | not serious | serious^b^ | Serious^c^ | none | 59 | 59 | - | Difference of medians **8.5 lower** | ⨁◯◯◯ Very low |  |
| **Neck (anterior) contamination during doffing of PPE: Small sized contaminated patches (< 1 cm2), median - PPE2 vs PPE1** | | | | | | | | | | | | |
| 1 | randomised trials | serious^a^ | not serious | serious^b^ | Serious^c^ | none | 59 | 59 | - | Difference of medians **6 lower** | ⨁◯◯◯ Very low |  |
| **Neck (posterior) contamination during doffing of PPE: Small sized contaminated patches (< 1 cm2), median - PPE3 vs PPE1** | | | | | | | | | | | | |
| 1 | randomised trials | serious^a^ | not serious | serious^b^ | Serious^c^ | none | 59 | 59 | - | Difference of medians **16.5 lower** | ⨁◯◯◯ Very low |  |
| **Neck (posterior) contamination during doffing of PPE: Small sized contaminated patches (< 1 cm2), median - PPE2 vs PPE1** | | | | | | | | | | | | |
| 1 | randomised trials | serious^a^ | not serious | serious^b^ | Serious^c^ | none | 59 | 59 | - | Difference of medians **17.5 lower** | ⨁◯◯◯ Very low |  |
| **Overall contamination during doffing of PPE: Extra large sized contaminated patches (≥ 5cm2), median - PPE3 vs PPE1** | | | | | | | | | | | | |
| 1 | randomised trials | serious^a^ | not serious | serious^b^ | Serious^c^ | none | 59 | 59 | - | Difference of medians **8 lower** | ⨁◯◯◯ Very low |  |
| **Overall contamination during doffing of PPE: Extra large sized contaminated patches (≥ 5cm2), median - PPE2 vs PPE1** | | | | | | | | | | | | |
| 1 | randomised trials | serious^a^ | not serious | serious^b^ | Serious^c^ | none | 59 | 59 | - | Difference of medians **4 lower** | ⨁◯◯◯ Very low |  |
| **Hair and head contamination during doffing of PPE: Extra large sized contaminated patches (≥ 5cm2), median - PPE3 vs PPE1** | | | | | | | | | | | | |
| 1 | randomised trials | serious^a^ | not serious | serious^b^ | serious^c^ | none | 59 | 59 | - | Difference of medians **0** | ⨁◯◯◯ Very low |  |
| **Hair and head contamination during doffing of PPE: Extra large sized contaminated patches (≥ 5cm2), median - PPE2 vs PPE1** | | | | | | | | | | | | |
| 1 | randomised trials | serious^a^ | not serious | serious^b^ | serious^c^ | none | 59 | 59 | - | Difference of medians **17 higher** | ⨁◯◯◯ Very low |  |
| **Neck (anterior) contamination during doffing of PPE: Extra large sized contaminated patches (≥ 5cm2), median - PPE3 vs PPE1** | | | | | | | | | | | | |
| 1 | randomised trials | serious^a^ | not serious | serious^b^ | serious^c^ | none | 59 | 59 | - | Difference of medians **24 lower** | ⨁◯◯◯ Very low |  |
| **Neck (anterior) contamination during doffing of PPE: Extra large sized contaminated patches (≥ 5cm2), median - PPE2 vs PPE1** | | | | | | | | | | | | |
| 1 | randomised trials | serious^a^ | not serious | serious^b^ | serious^c^ | none | 59 | 59 | - | Difference of medians **24 lower** | ⨁◯◯◯ Very low |  |
| **Neck (posterior) contamination during doffing of PPE: Extra large sized contaminated patches (≥ 5cm2), median - PPE3 vs PPE1** | | | | | | | | | | | | |
| 1 | randomised trials | serious^a^ | not serious | serious^b^ | serious^c^ | none | 59 | 59 | - | Difference of medians **0** | ⨁◯◯◯ Very low |  |
| **Neck (posterior) contamination during doffing of PPE: Extra large sized contaminated patches (≥ 5cm2), median - PPE2 vs PPE1** | | | | | | | | | | | | |
| 1 | randomised trials | serious^a^ | not serious | serious^b^ | serious^c^ | none | 59 | 59 | - | Difference of medians **0** | ⨁◯◯◯ Very low |  |
| **Overall contamination during doffing of PPE, any size, n (%)** | | | | | | | | | | | | |
| 1 | randomised trials | Serious^d^ | not serious | Serious^e^ | serious^c^ | none | 13/50 (26.0%) | 48/50 (96.0%) | **RR 0.27** (0.17 to 0.43) | **701 fewer per 1,000** Mainland– Gart: p <0.001  (from 797 fewer to 547 fewer) | ⨁◯◯◯ Very low |  |
| **Face contamination during doffing of PPE, any size, n (%)** | | | | | | | | | | | | |
| 1 | randomised trials | Serious^d^ | not serious | Serious^e^ | serious^c^ | none | 0/50 (0.0%) | 2/50 (4.0%) | **RR 0.20** (0.0098 to 4.06) | **32 fewer per 1,000** Mainland– Gart: p=1 (from 40 fewer to 123 more) | ⨁◯◯◯ Very low |  |
| **Back of the head contamination during doffing of PPE, any size, n (%)** | | | | | | | | | | | | |
| 1 | randomised trials | Serious^d^ | not serious | Serious^e^ | serious^c^ | none | 0/50 (0.0%) | 0/50 (0.0%) | not estimable | **0 fewer per 1,000**  Mainland– Gart: undefined | ⨁◯◯◯ Very low |  |
| **Neck (anterior) contamination during doffing of PPE, any size, n (%)** | | | | | | | | | | | | |
| 1 | randomised trials | Serious^d^ | not serious | Serious^e^ | serious^c^ | none | 3/50 (6.0%) | 48/50 (96.0%) | **RR 0.12** (0.038 to 0.35) | **845 fewer per 1,000** Mainland– Gart: p<0.001 (from 924 fewer to 621 fewer) | ⨁◯◯◯ Very low |  |
| **Neck (posterior) contamination during doffing of PPE, any size, n (%)** | | | | | | | | | | | | |
| 1 | randomised trials | Serious^d^ | not serious | Serious^e^ | serious^c^ | none | 1/50 (2.0%) | 9/50 (18.0%) | **RR 0.13** (0.017 to 0.98) | **157 fewer per 1,000** Mainland– Gart: p=0.012 (from 177 fewer to 4 fewer) | ⨁◯◯◯ Very low |  |

**CI:** confidence interval; **RR:** risk ratio

#### Explanations

a. We rated Suen et al., 2018, at a high risk of bias as the study lacked information on pre-specified outcomes based on study’s objectives and methods. Also, there was no information on blinding of participants or outcome assessors.

b. Downrated due to simulation study: Fluorescent contamination as a surrogate outcome for EVD/Marburg Virus Disease, other differences in evaluated PPE equipment other than just head/neck cover vs. no cover.

c. Few participants and optimal information size (OIS) threshold not met.

d. Zamora et el. 2006 was downrated once due to concerns with risk of bias. Unclear risk of bias for several domains, including allocation bias, blinding of participants, and unclear if all outcomes were reported.

e. Downrated due to simulation study: Fluorescent contamination as a surrogate outcome, other differences in evaluated PPE equipment other than just head/neck cover vs. no cover.

# Supplementary File 9 - Table 4. KQ5: **Humans Errors during donning and doffing of personal protective equipment**

| **Certainty assessment** | | | | | | | **№ of patients** | | **Effect** | | **Certainty** | **Importance** |
| --- | --- | --- | --- | --- | --- | --- | --- | --- | --- | --- | --- | --- |
| **№ of studies** | **Study design** | **Risk of bias** | **Inconsistency** | **Indirectness** | **Imprecision** | **Other considerations** | **A cover for the head and neck** | **No cover for the head and neck** | **Relative**  **[Calculated 95% CI]** | **Absolute**  **Reported p-value and test**  **[Calculated 95% CI]** |  |  |
| **Donning hood vs. Donning goggles/mask/surgical cap, n (%) - CTP-E vs. CTP-B** | | | | | | | | | | | | |
| 1 | non-randomised studies | serious^a^ | not serious | serious^b^ | serious^c^ | none | 3/30 (10.0%) | 3/30 (10.0%) | **RR 1.00** (0.22 to 4.56) | **0 fewer per 1,000** p-value: NR (from 78 fewer to 356 more) | ⨁◯◯◯ Very low |  |
| **Donning hood vs. Donning goggles/mask/surgical cap, n (%) - RTP-E vs. RTP-B** | | | | | | | | | | | | |
| 1 | non-randomised studies | serious^a^ | not serious | serious^b^ | serious^c^ | none | 2/30 (6.7%) | 4/30 (13.3%) | **RR 0.50** (0.099 to 2.53) | **67 fewer per 1,000** p-value: NR (from 120 fewer to 204 more) | ⨁◯◯◯ Very low |  |
| **Doffing hood vs. Doffing goggles/mask/surgical cap, n (%) - CTP-E vs. CTP-B** | | | | | | | | | | | | |
| 1 | non-randomised studies | serious^a^ | not serious | serious^b^ | serious^c^ | none | 5/30 (16.7%) | 5/30 (16.7%) | **RR 1.00** (0.32 to 3.10) | **0 fewer per 1,000** p-value: NR (from 113 fewer to 350 more) | ⨁◯◯◯ Very low |  |
| **Doffing hood vs. Doffing goggles/mask/surgical cap, n (%) - RTP-E vs. RTP-B** | | | | | | | | | | | | |
| 1 | non-randomised studies | serious^a^ | not serious | serious^b^ | serious^c^ | none | 5/30 (16.7%) | 1/30 (3.3%) | **RR 5.00** (0.62 to 40.29) | **133 more per 1,000** p-value: NR (from 13 fewer to 1,000 more) | ⨁◯◯◯ Very low |  |
| **Total errors, n (%) - CTP-E vs. CTP-B** | | | | | | | | | | | | |
| 1 | non-randomised studies | serious^a^ | not serious | serious^b^ | serious^c^ | none | 28/30 (93.3%) | 27/30 (90.0%) | **RR 0.67** (0.12 to 3.71) | **297 fewer per 1,000** p-value: NR (from 792 fewer to 1,000 more) | ⨁◯◯◯ Very low |  |
| **Total errors, n (%) - RTP-E vs. RTP-B** | | | | | | | | | | | | |
| 1 | non-randomised studies | serious^a^ | not serious | serious^b^ | serious^c^ | none | 23/30 (76.7%) | 16/30 (53.3%) | **RR 1.44** (0.98 to 2.12) | **235 more per 1,000** p-value: NR (from 11 fewer to 597 more) | ⨁◯◯◯ Very low |  |
| **Total errors count (mean ± SD) - CTP-E vs CTP-B** | | | | | | | | | | | | |
| 1 | non-randomised studies | serious^a^ | not serious | serious^b^ | serious^c^ | none | 30 | 30 | - | MD **1.3 lower** p-value: NR (2.2 lower to 0.36 lower) | ⨁◯◯◯ Very low |  |
| **Total errors count (mean ± SD) - RTP-E vs RTP-B** | | | | | | | | | | | | |
| 1 | non-randomised studies | serious^a^ | not serious | serious^b^ | serious^c^ | none | 30 | 30 | - | MD **1.9 lower** p-value: NR (2.92 lower to 0.88 lower) | ⨁◯◯◯ Very low |  |
| **Total critical errors, n (%) - CTP-E vs CTP-B** | | | | | | | | | | | | |
| 1 | non-randomised studies | serious^a^ | not serious | serious^b^ | serious^c^ | none | 26/30 (86.7%) | 27/30 (90.0%) | **RR 0.96** (0.80 to 1.16) | **36 fewer per 1,000** p-value: NR (from 180 fewer to 144 more) | ⨁◯◯◯ Very low |  |
| **Total critical errors, n (%) - RTP-E vs RTP-B** | | | | | | | | | | | | |
| 1 | non-randomised studies | serious^a^ | not serious | serious^b^ | serious^c^ | none | 21/30 (70.0%) | 13/30 (43.3%) | **RR 1.62** (1.01 to 2.59) | **269 more per 1,000** p-value: NR (from 4 more to 689 more) | ⨁◯◯◯ Very low |  |
| **Critical error count, mean ± SD - CTP-E vs CTP-B** | | | | | | | | | | | | |
| 1 | non-randomised studies | serious^a^ | not serious | serious^b^ | serious^c^ | none | 30 | 30 | - | MD **1.4 lower** p-value: NR (2.29 lower to 0.52 lower) | ⨁◯◯◯ Very low |  |
| **Critical error count, mean ± SD - RTP-E vs RTP-B** | | | | | | | | | | | | |
| 1 | non-randomised studies | serious^a^ | not serious | serious^b^ | serious^c^ | none | 30 | 30 | - | MD **1.7 lower** p-value: NR (2.68 lower to 0.72 lower) | ⨁◯◯◯ Very low |  |
| **Overall deviation rate (%) during donning of PPE - PP1 vs PPE3** | | | | | | | | | | | | |
| 1 | randomised trials | not serious | not serious | serious^d^ | serious^c^ | none | 3.5754/59 (6.1%) | 2.183/59 (3.7%) | **RR 2.00** (0.38 to 10.5) | **37 more per 1,000** p-value: NR (from 23 fewer to 352 more) | ⨁⨁◯◯ Low |  |
| **Overall deviation rate (%) during donning of PPE - PPE2 vs PPE3** | | | | | | | | | | | | |
| 1 | randomised trials | not serious | not serious | serious^d^ | serious^c^ | none | 3.54/59 (6.0%) | 2.183/59 (3.7%) | **RR 2.00** (0.38 to 10.5) | **37 more per 1,000** p-value: NR  (from 23 fewer to 352 more) | ⨁⨁◯◯ Low |  |
| **Deviation rate (%) during donning of hood - PPE1 vs PPE3** | | | | | | | | | | | | |
| 1 | randomised trials | not serious | not serious | serious^d^ | serious^c^ | none | 11.8/59 (20.0%) | 0/59 (0.0%) | not estimable | 200 more per 1,000 p-value: NR [95% CI: Undefined] | ⨁⨁◯◯ Low |  |
| **Deviation rate (%) during donning of hood - PPE2 vs PPE3** | | | | | | | | | | | | |
| 1 | randomised trials | not serious | not serious | serious^d^ | serious^c^ | none | 1.9647/59 (3.3%) | 0/59 (0.0%) | not estimable | 33 more per 1,000 p-value: NR [95% CI: Undefined] | ⨁⨁◯◯ Low |  |
| **Deviation rate (%) during donning of faceshield - PPE1 vs PPE3** | | | | | | | | | | | | |
| 1 | randomised trials | not serious | not serious | serious^d^ | serious^c^ | none | 6.8853/59 (11.7%) | 3.9353/59 (6.7%) | **RR 1.75** (0.54 to 5.66) | **50 more per 1,000** p-value: NR (from 31 fewer to 311 more) | ⨁⨁◯◯ Low |  |
| **Deviation rate (%) during donning of faceshield - PPE2 vs PPE3** | | | | | | | | | | | | |
| 1 | randomised trials | not serious | not serious | serious^d^ | serious^c^ | none | 8.85/59 (15.0%) | 3.9353/59 (6.7%) | **RR 2.25** (0.73 to 6.90) | **83 more per 1,000** p-value: NR (from 18 fewer to 394 more) | ⨁⨁◯◯ Low |  |
| **Overall deviation rate (%) during donning of PPE - PPE1 vs PPE3** | | | | | | | | | | | | |
| 1 | randomised trials | not serious | not serious | serious^d^ | serious^c^ | none | 1.7405/59 (2.9%) | 2.0768/59 (3.5%) | **RR 1.00** (0.15 to 6.87) | **0 fewer per 1,000** p-value: NR (from 30 fewer to 207 more) | ⨁⨁◯◯ Low |  |
| **Overall deviation rate (%) during donning of PPE - PPE2 vs PPE3** | | | | | | | | | | | | |
| 1 | randomised trials | not serious | not serious | serious^d^ | serious^c^ | none | 5.5932/59 (9.5%) | 2.0768/59 (3.5%) | **RR 3.00** (0.63 to 14.3) | **70 more per 1,000** p-value: NR (from 13 fewer to 468 more) | ⨁⨁◯◯ Low |  |
| **Deviation rate (%) during doffing of hood - PPE1 vs PPE3** | | | | | | | | | | | | |
| 1 | randomised trials | not serious | not serious | serious^d^ | serious^c^ | none | 2.95/59 (5.0%) | 0/59 (0.0%) | not estimable | 50 more per 1,000 p-value: NR [95% CI: Undefined] | ⨁⨁◯◯ Low |  |
| **Deviation rate (%) during doffing of hood - PPE2 vs PPE3** | | | | | | | | | | | | |
| 1 | randomised trials | not serious | not serious | serious^d^ | serious^c^ | none | 4.9147/59 (8.3%) | 0/59 (0.0%) | not estimable | 83 more per 1,000 p-value: NR [95% CI: Undefined | ⨁⨁◯◯ Low |  |
| **Deviation rate (%) during donning of faceshield - PPE1 vs PPE3** | | | | | | | | | | | | |
| 1 | randomised trials | not serious | not serious | serious^d^ | serious^c^ | none | 3.9353/59 (6.7%) | 5.9/59 (10.0%) | **RR 0.67** (0.20 to 2.24) | **33 fewer per 1,000** p-value: NR (from 80 fewer to 124 more) | ⨁⨁◯◯ Low |  |
| **Deviation rate (%) during donning of faceshield - PPE2 vs PPE3** | | | | | | | | | | | | |
| 1 | randomised trials | not serious | not serious | serious^d^ | serious^c^ | none | 6.8853/59 (11.7%) | 5.9/59 (10.0%) | **RR 1.17** (0.42 to 3.26) | **17 more per 1,000** p-value: NR (from 58 fewer to 226 more) | ⨁⨁◯◯ Low |  |
| **Total donning errors, n (%)** | | | | | | | | | | | | |
| 1 | randomised trials | serious^e^ | not serious | serious^f^ | serious^c^ | none | 19/50 (38.0%) | 2/50 (4.0%) | **RR 9.50** (2.33 to 38.70) | **340 more per 1,000** p-value: NR (from 53 more to 1,000 more) | ⨁◯◯◯ Very low |  |
| **Total doffing errors, n (%)** | | | | | | | | | | | | |
| 1 | randomised trials | serious^e^ | not serious | serious^f^ | serious^c^ | none | 6/50 (12.0%) | 12/50 (24.0%) | **RR 0.42** (0.17 to 1.03) | **139 fewer per 1,000** p-value: NR (from 199 fewer to 7 more) | ⨁◯◯◯ Very low |  |
| **Error in application of goggles during donning, n (%)** | | | | | | | | | | | | |
| 1 | randomised trials | serious^e^ | not serious | serious^f^ | serious^c^ | none | 2/50 (4.0%) | 0/50 (0.0%) | not estimable | 40 more per 1,000 p-value: NR [95% CI: Undefined] | ⨁◯◯◯ Very low |  |
| **Failure to zip up coveralls or put hood over head during donning, n (%)** | | | | | | | | | | | | |
| 1 | randomised trials | serious^e^ | not serious | serious^f^ | serious^c^ | none | 1/50 (2.0%) | - | not estimable | 20 more per 1,000 p-value: NR [95% CI: Undefined] | ⨁◯◯◯ Very low |  |
| **Error in application of bouffant hair-cover during donning, n (%)** | | | | | | | | | | | | |
| 1 | randomised trials | serious^e^ | not serious | serious^f^ | serious^c^ | none | - | 1/50 (2.0%) | not estimable | 20 fewer per 1,000 p-value: NR [95% CI: Undefined] | ⨁◯◯◯ Very low |  |
| **Error in removal of face shield during doffing, n (%)** | | | | | | | | | | | | |
| 1 | randomised trials | serious^e^ | not serious | serious^f^ | serious^c^ | none | - | 1/50 (2.0%) | not estimable | 20 fewer per 1,000 p-value: NR [95% CI: Undefined] | ⨁◯◯◯ Very low |  |
| **Error in removal of hair-cover during doffing, n (%)** | | | | | | | | | | | | |
| 1 | randomised trials | serious^e^ | not serious | serious^f^ | serious^c^ | none | - | 2/50 (4.0%) | not estimable | 40 fewer per 1,000 p-value: NR [95% CI: Undefined] | ⨁◯◯◯ Very low |  |

**CI:** confidence interval; **MD:** mean difference; **RR:** risk ratio

#### Explanations

a. Downrated once due to concerns with risk of bias, including measurement of error outcomes by unblinded assessor.

b. Downrated due to simulation study and other important differences in evaluated PPE equipment other than just head/neck cover vs. no cover.

c. Observed event rate is low, small number of participants and optimal information size (OIS) threshold not met.

d. Downrated due to simulation study: Fluorescent contamination as a surrogate outcome for EVD/Marburg Virus Disease, other differences in evaluated PPE equipment other than just head/neck cover vs. no cover.

e. Downrated due to concerns with risk of bias. Unclear risk of bias for several domains, including allocation bias, blinding of participants, and unclear if all outcomes were reported.

f. Downrated due to simulation study: Fluorescent contamination as a surrogate outcome, other differences in evaluated PPE equipment other than just head/neck cover vs. no cover.

# Supplementary File 9 - Table 6. KQ6: **Contamination during doffing of PPE**

| **Certainty assessment** | | | | | | | **№ of patients** | | **Effect** | | **Certainty** | **Importance** |
| --- | --- | --- | --- | --- | --- | --- | --- | --- | --- | --- | --- | --- |
| **№ of studies** | **Study design** | **Risk of bias** | **Inconsistency** | **Indirectness** | **Imprecision** | **Other considerations** | **Intervention**  (Wearing (goggles /face shield) under the head/neck covering) | **Comparator**  (Wearing eye protection (goggles/face shield) over the head /neck covering) | **Relative**  **[Calculated 95% CI]** | **Absolute**  **Reported p-value and test**  **[Calculated 95% CI]** |  |  |
| **Number of participants (n/N, %) with small fluorescent patches after various personal protective equipment (PPE) protocols - WHO, coverall and N95 vs CDC, coverall and PAPR** | | | | | | | | | | | | |
| 1 | randomised trials | serious^a^ | not serious | serious^b^ | serious^c^ | none | 0/3 (0.0%) | 0/3 (0.0%) | not estimable | 0 more per 1,000 | ⨁◯◯◯ Very low |  |
| **Number of participants (n/N, %) with small fluorescent patches after various personal protective equipment (PPE) protocols - WHO, coverall and N95 vs CDC, coverall and N95** | | | | | | | | | | | | |
| 1 | randomised trials | serious^a^ | not serious | serious^b^ | serious^c^ | none | 0/3 (0.0%) | 1/3 (33.3%) | not estimable | 333 less per 1,000 | ⨁◯◯◯ Very low |  |
| **Number of participants (n/N, %) with small fluorescent patches after various personal protective equipment (PPE) protocols - WHO, coverall and N95 vs ECDC, coverall and N95** | | | | | | | | | | | | |
| 1 | randomised trials | serious^a^ | not serious | serious^b^ | serious^c^ | none | 0/3 (0.0%) | 0/3 (0.0%) | not estimable | 0 more per 1,000 | ⨁◯◯◯ Very low |  |
| **Number of participants (n/N, %) with small fluorescent patches after various personal protective equipment (PPE) protocols - WHO, coverall and N95 vs Health Canada, gown and N95** | | | | | | | | | | | | |
| 1 | randomised trials | serious^a^ | not serious | serious^b^ | serious^c^ | none | 0/3 (0.0%) | 1/3 (33.3%) | not estimable | 333 less per 1,000 | ⨁◯◯◯ Very low |  |
| **Number of participants (n/N, %) with small fluorescent patches after various personal protective equipment (PPE) protocols - WHO, coverall and N95 vs NC, coverall and N95** | | | | | | | | | | | | |
| 1 | randomised trials | serious^a^ | not serious | serious^b^ | serious^c^ | none | 0/3 (0.0%) | 0/3 (0.0%) | not estimable | 0 more per 1,000 | ⨁◯◯◯ Very low |  |
| **Number of participants (n/N, %) with small fluorescent patches after various personal protective equipment (PPE) protocols - WHO, coverall and N95 vs NSW DoH CEC, gown and PAPR** | | | | | | | | | | | | |
| 1 | randomised trials | serious^a^ | not serious | serious^b^ | serious^c^ | none | 0/3 (0.0%) | 0/3 (0.0%) | not estimable | 0 more per 1,000 | ⨁◯◯◯ Very low |  |
| **Number of participants (n/N, %) with small fluorescent patches after various personal protective equipment (PPE) protocols - WHO, coverall and N95 vs NSW DoH CEC, gown and N95** | | | | | | | | | | | | |
| 1 | randomised trials | serious^a^ | not serious | serious^b^ | serious^c^ | none | 0/3 (0.0%) | 0/3 (0.0%) | not estimable | 0 more per 1,000 | ⨁◯◯◯ Very low |  |
| **Number of participants (n/N, %) with small fluorescent patches after various personal protective equipment (PPE) protocols - WHO, coverall and N95 vs MSF, coverall and N95** | | | | | | | | | | | | |
| 1 | randomised trials | serious^a^ | not serious | serious^b^ | serious^c^ | none | 0/3 (0.0%) | 0/3 (0.0%) | not estimable | 0 more per 1,000 | ⨁◯◯◯ Very low |  |
| **Number of participants (n/N, %) with small fluorescent patches after various personal protective equipment (PPE) protocols - WHO, coverall and N95 vs WHO, gown and N95** | | | | | | | | | | | | |
| 1 | randomised trials | serious^a^ | not serious | serious^b^ | serious^c^ | none | 0/3 (0.0%) | 0/3 (0.0%) | not estimable | 0 more per 1,000 | ⨁◯◯◯ Very low |  |
| **Number of participants (n/N, %) with large fluorescent patches after various personal protective equipment (PPE) protocols - WHO, coverall and N95 vs CDC, coverall and PAPR** | | | | | | | | | | | | |
| 1 | randomised trials | serious^a^ | not serious | serious^b^ | serious^c^ | none | 1/3 (33.3%) | 0/3 (0.0%) | not estimable | 333 more per 1,000 | ⨁◯◯◯ Very low |  |
| **Number of participants (n/N, %) with large fluorescent patches after various personal protective equipment (PPE) protocols - WHO, coverall and N95 vs CDC, coverall and N95** | | | | | | | | | | | | |
| 1 | randomised trials | serious^a^ | not serious | serious^b^ | serious^c^ | none | 1/3 (33.3%) | 0/3 (0.0%) | not estimable | 333 more per 1,000 | ⨁◯◯◯ Very low |  |
| **Number of participants (n/N, %) with large fluorescent patches after various personal protective equipment (PPE) protocols - WHO, coverall and N95 vs ECDC, coverall and N95** | | | | | | | | | | | | |
| 1 | randomised trials | serious^a^ | not serious | serious^b^ | serious^c^ | none | 1/3 (33.3%) | 0/3 (0.0%) | not estimable | 333 more per 1,000 | ⨁◯◯◯ Very low |  |
| **Number of participants (n/N, %) with large fluorescent patches after various personal protective equipment (PPE) protocols - WHO, coverall and N95 vs Health Canada, gown and N95** | | | | | | | | | | | | |
| 1 | randomised trials | serious^a^ | not serious | serious^b^ | serious^c^ | none | 1/3 (33.3%) | 0/3 (0.0%) | not estimable | 333 more per 1,000 | ⨁◯◯◯ Very low |  |
| **Number of participants (n/N, %) with large fluorescent patches after various personal protective equipment (PPE) protocols - WHO, coverall and N95 vs NC, coverall and N95** | | | | | | | | | | | | |
| 1 | randomised trials | serious^a^ | not serious | serious^b^ | serious^c^ | none | 1/3 (33.3%) | 1/3 (33.3%) | not estimable | 0 more per 1,000 | ⨁◯◯◯ Very low |  |
| **Number of participants (n/N, %) with large fluorescent patches after various personal protective equipment (PPE) protocols - WHO, coverall and N95 vs NSW DoH CEC, gown and PAPR** | | | | | | | | | | | | |
| 1 | randomised trials | serious^a^ | not serious | serious^b^ | serious^c^ | none | 1/3 (33.3%) | 0/3 (0.0%) | not estimable | 333 more per 1,000 | ⨁◯◯◯ Very low |  |
| **Number of participants (n/N, %) with large fluorescent patches after various personal protective equipment (PPE) protocols - WHO, coverall and N95 vs NSW DoH CEC, gown and N95** | | | | | | | | | | | | |
| 1 | randomised trials | serious^a^ | not serious | serious^b^ | serious^c^ | none | 1/3 (33.3%) | 0/3 (0.0%) | not estimable | 333 more per 1,000 | ⨁◯◯◯ Very low |  |
| **Number of participants (n/N, %) with large fluorescent patches after various personal protective equipment (PPE) protocols - WHO, coverall and N95 vs MSF, coverall and N95** | | | | | | | | | | | | |
| 1 | randomised trials | serious^a^ | not serious | serious^b^ | serious^c^ | none | 1/3 (33.3%) | 0/3 (0.0%) | not estimable | 333 more per 1,000 | ⨁◯◯◯ Very low |  |
| **Number of participants (n/N, %) with large fluorescent patches after various personal protective equipment (PPE) protocols - WHO, coverall and N95 vs WHO, gown and N95** | | | | | | | | | | | | |
| 1 | randomised trials | serious^a^ | not serious | serious^b^ | serious^c^ | none | 1/3 (33.3%) | 0/3 (0.0%) | not estimable | 333 more per 1,000 | ⨁◯◯◯ Very low |  |
| **Overall contamination during doffing of PPE: Small sized contaminated patches (< 1 cm2), median** | | | | | | | | | | | | |
| 1 | randomised trials | serious^d^ | not serious | Serious^e^ | serious^c^ | none | 59 | 59 | - | Difference of medians **2 higher** | ⨁⨁◯◯ Low |  |
| **Hair and head contamination during doffing of PPE: Small sized contaminated patches (< 1 cm2), median** | | | | | | | | | | | | |
| 1 | randomised trials | serious^d^ | not serious | Serious^e^ | serious^c^ | none | 59 | 59 | - | Difference of medians **1 higher** | ⨁◯◯◯ Very low |  |
| **Neck (anterior) contamination during doffing of PPE: Small sized contaminated patches (< 1 cm2), median** | | | | | | | | | | | | |
| 1 | randomised trials | serious^d^ | not serious | Serious^e^ | serious^c^ | none | 59 | 59 | - | Difference of medians **2.5 higher** | ⨁◯◯◯ Very low |  |
| **Neck (posterior) contamination during doffing of PPE: Small sized contaminated patches (< 1 cm2), median** | | | | | | | | | | | | |
| 1 | randomised trials | serious^d^ | not serious | serious^d^ | serious^c^ | none | 59 | 59 | - | Difference of medians **1 lower** | ⨁◯◯◯ Very low |  |
| **Overall contamination during doffing of PPE: Extra large sized contaminated patches (≥ 5cm2), median** | | | | | | | | | | | | |
| 1 | randomised trials | serious^d^ | not serious | Serious^e^ | serious^c^ | none | 59 | 59 | - | Difference of medians **4 higher** | ⨁◯◯◯ Very low |  |
| **Hair and head contamination during doffing of PPE: Extra large sized contaminated patches (≥ 5cm2), median** | | | | | | | | | | | | |
| 1 | randomised trials | serious^d^ | not serious | Serious^e^ | serious^c^ | none | 59 | 59 | - | Difference of medians **17 higher** | ⨁◯◯◯ Very low |  |
| **Neck (anterior) contamination during doffing of PPE: Extra large sized contaminated patches (≥ 5cm2), median** | | | | | | | | | | | | |
| 1 | randomised trials | serious^d^ | not serious | Serious^e^ | serious^c^ | none | 59 | 59 | - | Difference of medians **0** | ⨁◯◯◯ Very low |  |
| **Neck (posterior) contamination during doffing of PPE: Extra large sized contaminated patches (≥ 5cm2), median** | | | | | | | | | | | | |
| 1 | randomised trials | serious^d^ | not serious | Serious^e^ | serious^c^ | none | 59 | 59 | - | Difference of medians **0** | ⨁◯◯◯ Very low |  |

**CI:** confidence interval

#### Explanations

a. Chughtai et al., 2018 was rated to have a high risk of bias as there is no information on randomization, allocation concealment and blinding of participants and outcome assessors. Additionally, the domains' effect of assignment to intervention (Domain 2) and Risk of bias in the measurement of the outcome (Domain 4) were rated to have a high risk of bias.

b. Downrated due to simulation study: Fluorescent contamination as a surrogate outcome for EVD/Marburg Virus Disease, other differences in evaluated PPE equipment beyond order of face cover and hood.

c. Few participants and optimal information size (OIS) threshold not met.

d. We rated Suen et al., 2018, at a high risk of bias as the study lacked information on pre-specified outcomes based on study’s objectives and methods. Also, there was no information on blinding of participants or outcome assessors.

e. Downrated due to simulation study: Fluorescent contamination as a surrogate outcome for EVD/Marburg Virus Disease, other differences in evaluated PPE equipment beyond order of face cover and hood.

# Supplementary File 9 - Table 7. KQ6: **Deviation rate (%) during donning and doffing of personal protective equipment**

| **Certainty assessment** | | | | | | | **№ of patients** | | **Effect** | | | | **Importance** |
| --- | --- | --- | --- | --- | --- | --- | --- | --- | --- | --- | --- | --- | --- |
| **№ of studies** | **Study design** | **Risk of bias** | **Inconsistency** | **Indirectness** | **Imprecision** | **Other considerations** | **Intervention**  (Wearing (goggles /face shield) under the head/neck covering) | **Comparator**  (Wearing eye protection (goggles/face shield) over the head /neck covering) | **Relative**  **[Calculated 95% CI]** | | **Absolute**  **Reported p-value and test**  **[Calculated 95% CI]** | **Certainty** |  |
| **Overall deviation rate (%) during donning of PPE** | | | | | | | | | | | | | |
| 1 | randomised trials | serious^a^ | not serious | Serious^b^ | Serious^c^ | none | 6.00% | 6.06% | **RR 1.00**  (0.26 to 3.81) | **0 fewer per 1,000**  p-value: NR (from 45 fewer to 170 more) | | ⨁◯◯◯ Very low | |
| **Deviation rate (%) during donning of hood** | | | | | | | | | | | | | |
| 1 | randomised trials | serious^a^ | not serious | Serious^b^ | Serious^c^ | none | 3.33% | 20.0% | **RR 0.17**  (0.039 to 0.71) | **166 fewer per 1,000**  p-value: NR (from 192 fewer to 58 fewer) | | ⨁◯◯◯ Very low | |
| **Deviation rate (%) during donning of faceshield** | | | | | | | | | | | | | |
| 1 | randomised trials | serious^a^ | not serious | Serious^b^ | Serious^c^ | none | 15.00% | 11.67% | **RR 1.29**  (0.51 to 3.22) | **34 more per 1,000**  p-value: NR (from 57 fewer to 259 more) | | ⨁◯◯◯ Very low | |
| **Overall deviation rate (%) during doffing of PPE** | | | | | | | | | | | | | |
| 1 | randomised trials | serious^a^ | not serious | Serious^b^ | Serious^c^ | none | 9.48% | 2.95% | **RR 3.00**  (0.63 to 14.30) | **59 more per 1,000**  p-value: NR (from 11 fewer to 392 more) | | ⨁◯◯◯ Very low | |
| **Deviation rate (%) during doffing of hood** | | | | | | | | | | | | | |
| 1 | randomised trials | serious^a^ | not serious | Serious^b^ | Serious^c^ | none | 8.33% | 5.00% | **RR 1.67**  (0.42 to 6.66) | **33 more per 1,000**  p-value: NR (from 29 fewer to 283 more) | | ⨁◯◯◯ Very low | |
| **Deviation rate (%) during doffing of faceshield** | | | | | | | | | | | | | |
| 1 | randomised trials | serious^a^ | not serious | Serious^b^ | Serious^c^ | none | 11.67% | 6.67% | **RR 1.75**  (0.54 to 5.66) | **50 more per 1,000**  p-value: NR (from 31 fewer to 311 more) | | ⨁◯◯◯ Very low | |

**CI:** confidence interval

#### Explanations

a. We rated Suen et al., 2018, at a high risk of bias as the study lacked information on pre-specified outcomes based on study’s objectives and methods. Also, there was no information on blinding of participants or outcome assessors.

b. Downrated due to simulation study design: Fluorescent contamination as a surrogate outcome for EVD/Marburg Virus Disease, other differences in evaluated PPE equipment beyond order of face cover and hood.

c. Few participants and optimal information size (OIS) threshold not met.

# Supplementary File 9 - Table 8. KQ10: **Transfer of Φ6 or MS2**

| **Certainty assessment** | | | | | | | **№ of patients** | | **Effect** | | **Certainty** | **Importance** |
| --- | --- | --- | --- | --- | --- | --- | --- | --- | --- | --- | --- | --- |
| **№ of studies** | **Study design** | **Risk of bias** | **Inconsistency** | **Indirectness** | **Imprecision** | **Other considerations** | **Chlorine spray of PPE** | **No chlorine spray of PPE** | **Relative**  **[Calculated 95% CI]** | **Absolute**  **Reported p-value and test**  **[Calculated 95% CI]** |  |  |
| **Transfer of Phi6 to inner gloves, hands, face or scrubs following doffing protocol**  **(n/N, %)** | | | | | | | | | | | | |
| 1 | observational studies | not serious^a^ | not serious^b^ | serious^c^ | very serious^d^ | none | 0/5 (0.0%) | 0/10 (0.0%) | not estimable | 0 more per 1,000 p-value: NR  [95% CI: Undefined] | ⨁◯◯◯  Very low |  |
| **Transfer of MS2 to inner gloves following doffing protocol**  **(n/N, %)** | | | | | | | | | | | | |
| 1 | observational studies | not serious^a^ | not serious^b^ | serious^e^ | very serious^f^ | none | 0/5 (0.0%) | 8/10 (80.0%) | not estimable | 800 less per 1,000 p-value: NR  [95% CI: Undefined] | ⨁◯◯◯  Very low |  |
| **Transfer of MS2 to hands following doffing protocol**  **(n/N, %)** | | | | | | | | | | | | |
| 1 | observational studies | not serious^a^ | not serious^b^ | serious^e^ | very serious^g^ | none | 1/5 (20.0%) | 0/10 (0.0%) | not estimable | 200 more per 1,000 p-value: NR  [95% CI: Undefined] | ⨁◯◯◯  Very low |  |
| **Transfer of MS2 to face following doffing protocol**  **(n/N, %)** | | | | | | | | | | | | |
| 1 | observational studies | not serious^a^ | not serious^b^ | serious^e^ | very serious^d^ | none | 0/5 (0.0%) | 0/10 (0.0%) | not estimable | 0 more per 1,000 p-value: NR  [95% CI: Undefined] | ⨁◯◯◯  Very low |  |
| **Transfer of MS2 to scrubs following doffing protocol**  **(n/N, %)** | | | | | | | | | | | | |
| 1 | observational studies | not serious^a^ | not serious^b^ | serious^e^ | very serious^g^ | none | 1/5 (20.0%) | 0/10 (0.0%) | not estimable | 200 more per 1,000 p-value: NR  [95% CI: Undefined] | ⨁◯◯◯  Very low |  |

**CI:** confidence interval

#### Explanations

a. The overall risk of bias was rated to be "moderate" using the ROBINS-I tool for non-randomized studies. The study was judged to be of low risk of bias for all but one domain. One domain was rated at moderate risk of bias due to a lack of blinding of the participants of the intervention and the trained monitor guiding participants through the doffing process.

b. Judged to be not serious as there was only one relevant study for this outcome.

c. Downrated once due to simulation study. Φ6 is a surrogate for enveloped viruses such as Ebola.

d. No events in either group, very small sample size and optimal information size (OIS) not met.

e. Downrated twice due to simulation study and use of MS2 as surrogate agent (non-enveloped virus surrogate).

f. Few events, very small sample size and OIS not met.

g. Only one event, very small samples size and OIS not met.

# Supplementary File 9 - Table 9. KQ10: **Infection with Ebola virus**

| **Certainty assessment** | | | | | | | **№ of patients** | | **Effect** | | **Certainty** | **Importance** |
| --- | --- | --- | --- | --- | --- | --- | --- | --- | --- | --- | --- | --- |
| **№ of studies** | **Study design** | **Risk of bias** | **Inconsistency** | **Indirectness** | **Imprecision** | **Other considerations** | **Chlorine spray of PPE** | **No chlorine spray of PPE** | **Relative**  **[Calculated 95% CI]** | **Absolute**  **Reported p-value and test**  **[Calculated 95% CI]** |  |  |
| **Infection with Ebola Virus** | | | | | | | | | | | | |
| 1 | observational studies | serious^a^ | not serious^b^ | not serious | serious^c^ | none | 33/132 (25.0%) | 7/98 (7.1%) | **RR 3.52**  (1.62 to 7.58) | **180 more per 1,000**  p-value: NR (from 44 more to 470 more) | ⨁◯◯◯  Very low |  |

**CI:** confidence interval; **RR:** risk ratio

#### Explanations

a. Risk of bias was judged to be serious using the ROBINS-I tool. The study was downrated due to use snowball sampling for a convenience sample, relying on self-reports for ascertainment of exposures and lack of reporting of details on PPE equipment or doffing protocols used between HCW roles.

b. No inconsistency detected as only one study included for this outcome.

c. Optimal information size not met and not a large sample size.
