# Supplementary material for: Infection prevention and control measures for Ebola and Marburg disease: a series of rapid reviews
Source: BMJ Open. 2026 Jul 9;16(7):e115610. doi: 10.1136/bmjopen-2025-115610 (PMC13358256; doi:10.1136/bmjopen-2025-115610)
Supplement: online supplemental file 10 [file bmjopen-16-7-s010.docx]

**Supplementary file 10.** Summary of Findings Tables

**Supplementary file 10 Table 1.** KQ5 Summary of Findings (Should HWs in direct contact and/or indirect contact to patients with EBOD or MARD cover head and neck skin and mucous membranes or just cover mucous membranes?)

| ***Study details*** | ***Intervention (cover head/neck skin and mucus membranes)*** | ***Comparator (cover mucous membranes)*** | ***Outcome in intervention group*** | ***Outcome in control group*** | ***Effect Magnitude, Reported Statistical Test [Calculated 95% CI^z^]*** | ***Quality Assessment^a^*** | ***GRADE Assessment*** |
| --- | --- | --- | --- | --- | --- | --- | --- |
| Heat Tolerance Outcomes | | | | | | | |
| Time (min) to reach critical core temperature of 39°C under condition A^b^  (mean, SD) | | | | | | | |
| Coca, 2015 [38] | E4^c^ | E2^d^ | 62±6 min | 78±7 min | MD 16 min fewer LSD test: P = 0.04 [95% CI: 1.22 fewer to 30.78 fewer] | Moderate risk of bias | ⨁◯◯◯  Very low |
|  | E3^e^ | E2^d^ | 65±3 min | 78±7 min | MD 13 min fewer LSD test: P=0.04 [95% CI: 0.79 fewer to 25.2 fewer] |  |  |
|  | E4^c^ | E1^f^ | 62±6 min | +80 min | MD 18 min fewer LSD test: P<0.05 [95% CI: Undefined] |  |  |
|  | E3^e^ | E1^f^ | 65±3 min | +80 min | MD 15 min fewer LSD test: P<0.05 [95% CI: Undefined] |  |  |
| Body surface skin temperature (°C) at time to reach critical core temperature of 39°C under condition A^b^  (mean, SD) | | | | | | | |
| Coca, 2015 [38] | E4^c^ | E2^d^ | 38.4 ± 0.8 | 37.7 ± 0.2 | MD 0.7 C higher LSD test: P>0.05 [95% CI: 0.62 lower to 2.02 higher] | Moderate risk of bias | ⨁◯◯◯  Very low |
|  | E3^e^ | E2^d^ | 38.3 ± 0.2 | 37.7 ± 0.2 | MD 0.6 C higher LSD test: P>0.05 [95% CI: 0.14 higher to 1.05 higher] |  |  |
|  | E4^c^ | E1^f^ | 38.4 ± 0.8 | 37.3 ± 0.3 | MD 1.1 C higher LSD test: P <0.05 [95% CI: 0.27 lower to 2.47 higher] |  |  |
|  | E3^e^ | E1^f^ | 38.3 ± 0.2 | 37.3 ± 0.3 | MD 1 C higher LSD test: P <0.05 [95% CI: 0.42 higher to 1.58 higher] |  |  |
| Heat sensation^g^ at time to reach critical core temperature of 39°C under condition A^b^  (mean, SD) | | | | | | | |
| Coca, 2015 [38] | E4^c^ | E2^d^ | 3.8 ± 0.1 | 3.5 ± 0.2 | MD 0.3 C higher LSD test: P>0.05 [95% CI: 0.06 lower to 0.66 higher] | Moderate risk of bias | ⨁◯◯◯  Very low |
|  | E3^e^ | E2^d^ | 3.7 ± 0.1 | 3.5 ± 0.2 | MD 0.2 C higher LSD test: P>0.05 [95% CI: 0.16 lower to 0.56 higher] |  |  |
|  | E4^c^ | E1^f^ | 3.8 ± 0.1 | 3.6 ± 0.2 | MD 0.2 C higher LSD test: P>0.05 [95% CI: 0.16 lower to 0.56 higher] |  |  |
|  | E3^e^ | E1^f^ | 3.7 ± 0.1 | 3.6 ± 0.2 | MD 0.1 C higher LSD test: P>0.05 [95% CI: 0.26 lower to 0.46 higher] |  |  |
| Discomfort^h^ at time to reach critical core temperature of 39°C under condition A^b^  (mean, SD) | | | | | | | |
| Coca, 2015 [38] | E4^c^ | E2^d^ | − 3.4 ± 0.1 | − 3.2 ± 0.1 | MD 0.2 C lower LSD test: P>0.05 [95% CI: 0.43 lower to 0.03 higher] | Moderate risk of bias | ⨁◯◯◯  Very low |
|  | E3^e^ | E2^d^ | − 3.4 ± 0.1 | − 3.2 ± 0.1 | MD 0.2 C lower LSD test: P>0.05 [95% CI: 0.43 lower to 0.03 higher] |  |  |
|  | E4^c^ | E1^f^ | − 3.4 ± 0.1 | − 3.2 ± 0.1 | MD 0.2 C lower LSD test: P>0.05 [95% CI: 0.43 lower to 0.03 higher] |  |  |
|  | E3^e^ | E1^f^ | − 3.4 ± 0.1 | − 3.2 ± 0.1 | MD 0.2 C lower LSD test: P>0.05 [95% CI: 0.43 lower to 0.03 higher] |  |  |
| Core temperature (°C) after 80 minutes of activity under condition B^i^ (mean, SD) | | | | | | | |
| Coca, 2015 [38] | E4^c^ | E2^d^ | 38.9 ± 0.2 | 38.33 ± 0.1 | MD 0.57 C higher LSD test: P <0.05 [95% CI: 0.21 higher to 0.93 higher] | Moderate risk of bias | ⨁◯◯◯  Very low |
|  | E3^e^ | E2^d^ | 38.7 ± 0.1 | 38.33 ± 0.1 | MD 0.37 C higher LSD test: P <0.05 [95% CI: 0.14 higher to 0.60 higher] |  |  |
|  | E4^c^ | E1^f^ | 38.9 ± 0.2 | 38.05 ± 0.1 | MD 0.85 C higher LSD test: P <0.05 [95% CI: 0.49 higher to 1.2 higher] |  |  |
|  | E3^e^ | E1^f^ | 38.7 ± 0.1 | 38.05 ± 0.1 | MD 0.65 C higher LSD test: P <0.05 [95% CI: 0.42 higher to 0.87 higher] |  |  |
| Body surface skin temperature (°C) after 80 minutes of activity under condition B^i^ (mean, SD) | | | | | | | |
| Coca, 2015 [38] | E4^c^ | E2^d^ | 37.6 ± 0.4 | 36.4 ± 0.4 | MD 1.2 C higher LSD test: P <0.05 [95% CI: 0.29 higher to 2.1 higher] | Moderate risk of bias | ⨁◯◯◯  Very low |
|  | E3^e^ | E2^d^ | 36.9 ± 0.2 | 36.4 ± 0.4 | MD 0.5 C higher LSD test: P>0.05 [95% CI: 0.22 lower to 1.22 higher] |  |  |
|  | E4^c^ | E1^f^ | 37.6 ± 0.4 | 35.8 ± 0.6 | MD 1.8 C higher LSD test: P <0.05 [95% CI: 0.64 higher to 2.96 higher] |  |  |
|  | E3^e^ | E1^f^ | 36.9 ± 0.2 | 35.8 ± 0.6 | MD 1.1 C higher LSD test: P>0.05 [95% CI: 0.09 higher to 2.11 higher] |  |  |
| Heat sensation^g^ after 80 minutes of activity under condition B^i^ (mean, SD) | | | | | | | |
| Coca, 2015 [38] | E4^c^ | E2^d^ | 3.2 ± 0.6 | 2.5 ± 0.6 | MD 0.7 higher LSD test: P>0.05 [95% CI: 0.66 lower to 2.06 higher] | Moderate risk of bias | ⨁◯◯◯  Very low |
|  | E3^e^ | E2^d^ | 2.5 ± 0.4 | 2.5 ± 0.6 | MD 0 LSD test: P>0.05 [95% CI: 1.16 lower to 1.16 higher] |  |  |
|  | E4^c^ | E1^f^ | 3.2 ± 0.6 | 2.4 ± 0.5 | MD 0.8 higher LSD test: P <0.05 [95% CI: 0.45 lower to 2.05 higher] |  |  |
|  | E3^e^ | E1^f^ | 2.5 ± 0.4 | 2.4 ± 0.5 | MD 0.1 higher LSD test: P>0.05 [95% CI: 0.93 lower to 1.13 higher] |  |  |
| Discomfort^h^ after 80 minutes of activity under condition B^i^ (mean, SD) | | | | | | | |
| Coca, 2015 [38] | E4^c^ | E2^d^ | − 3.2 ± 0.2 | − 2.6 ± 0.4 | MD 0.6 lower LSD test: P <0.05 [95% CI: 1.32 lower to 0.12 higher] | Moderate risk of bias | ⨁◯◯◯  Very low |
|  | E3^e^ | E2^d^ | − 3 ± 0.2 | − 2.6 ± 0.4 | MD 0.4 lower LSD test: P>0.05 [95% CI: 1.12 lower to 0.32 higher] |  |  |
|  | E4^c^ | E1^f^ | − 3.2 ± 0.2 | − 2.3 ± 0.3 | MD 0.9 lower LSD test: P <0.05 [95% CI: 0.32 lower to 1.48 lower] |  |  |
|  | E3^e^ | E1^f^ | − 3 ± 0.2 | − 2.3 ± 0.3 | MD 0.7 lower LSD test: P>0.05 [95% CI: 0.12 lower to 1.28 lower] |  |  |
| Core Temperature (°C) at end of exercise (mean, SD) | | | | | | | |
| Coca, 2017 [37] | E3^j^ | E1^k^ | 38.91 ± 0.29 | 38.18 ± 0.46 | MD 0.73 C higher LSD test: P <0.05 [95% CI: 0.14 lower to 1.6 higher] | Serious risk of bias | ⨁◯◯◯  Very low |
|  | E2^l^ | E1^k^ | 38.78 ± 0.36 | 38.18 ± 0.46 | MD 0.6 C higher LSD test: P <0.05 [95% CI: 0.34 lower to 1.54 higher] |  |  |
| Skin Temperature (°C) at end of exercise (mean, SD) | | | | | | | |
| Coca, 2017 [37] | E3^j^ | E1^k^ | 37.94 ± 0.15 | 36.12 ± 0.65 | MD 1.8 C higher LSD test: P>0.05 [95% CI: 0.75 higher to 2.89 higher] | Serious risk of bias | ⨁◯◯◯  Very low |
|  | E2^l^ | E1^k^ | 37.21 ± 0.21 | 36.12 ± 0.65 | MD 1.09 C higher LSD test: P>0.05 [95% CI: 0 to 2.19 higher] |  |  |
| Heart Rate (beats per minute) at end of exercise | | | | | | | |
| Coca, 2017 [37] | E3^j^ | E1^k^ | 163 ± 17.52 | 135.57 ± 15.05 | MD 27.43 BPM higher LSD test: P <0.05 [95% CI: 9.59 lower to 64.45 higher] | Serious risk of bias | ⨁◯◯◯  Very low |
|  | E2^l^ | E1^k^ | 156 ± 16.71 | 135.57 ± 15.05 | MD 20.43 BPM higher LSD test: P <0.05 [95% CI: 15.62 higher to 56.48 higher] |  |  |
| Average sweat weight loss (kg) per hour (mean, SD) | | | | | | | |
| Coca, 2017 [37] | E3^j^ | E1^k^ | 1.48 ± 0.47 kg | 0.94 ± 0.40 kg | MD 0.54 kg higher LSD test: P < 0.001 [95% CI: 0.45 lower to 1.53 higher] | Serious risk of bias | ⨁◯◯◯  Very low |
|  | E2^l^ | E1^k^ | 1.26 ± 0.53 kg | 0.94 ± 0.40 kg | MD 0.32 kg higher LSD test: P = 0.03 [95% CI: 0.74 lower to 1.38 higher] |  |  |
| Heat Sensation^g^ at end of exercise (mean, SD) | | | | | | | |
| Coca, 2017 [37] | E3^j^ | E1^k^ | 3.86 ± 0.38 | 3.29 ± 0.49 | MD 0.57 higher LSD test: P <0.05 [95% CI: 0.42 lower to 1.56 higher] | Serious risk of bias | ⨁◯◯◯  Very low |
|  | E2^l^ | E1^k^ | 3.86 ± 0.38 | 3.29 ± 0.49 | MD 0.57 higher LSD test: P <0.05 [95% CI: 0.42 lower to 1.56 higher] |  |  |
| Thermal Comfort^m^ at end of exercise (mean, SD) | | | | | | | |
| Coca, 2017 [37] | E3^j^ | E1^k^ | 2.71 ± 2.56 | 2.71 ± 0.76 | MD 0 LSD test: P>0.05 [95% CI: 4.28 lower to 4.28 higher] | Serious risk of bias | ⨁◯◯◯  Very low |
|  | E2^l^ | E1^k^ | 3.57 ± 0.79 | 2.71 ± 0.76 | MD 0.86 higher LSD test: P <0.05 [95% CI: 0.90 lower to 2.62 higher] |  |  |
| Rated perceived exertion^n^ at end of exercise (mean, SD) | | | | | | | |
| Coca, 2017 [37] | E3^j^ | E1^k^ | 15.29 ± 2.50 | 11.86 ± 2.12 | MD 3.43 higher LSD test: P <0.05 [95% CI: 1.82 lower to 8.68 higher] | Serious risk of bias | ⨁◯◯◯  Very low |
|  | E2^l^ | E1^k^ | 14.43 ± 3.10 | 11.86 ± 2.12 | MD 2.57 higher LSD test: P <0.05 [95% CI: 3.45 lower to 8.59 higher] |  |  |
| Breathing comfort^o^ at end of exercise (mean, SD) | | | | | | | |
| Coca, 2017 [37] | E3^j^ | E1^k^ | 5.14 ± 0.69 | 3.57 ± 1.27 | MD 1.57 higher LSD test: P <0.05 [95% CI: 0.75 lower to 3.89 higher] | Serious risk of bias | ⨁◯◯◯  Very low |
|  | E2^l^ | E1^k^ | 5.29 ± 1.11 | 3.57 ± 1.27 | MD 1.72 higher LSD test: P <0.05 [95% CI: 0.98 lower to 4.42 higher] |  |  |
| Wetness^p^ at end of exercise (mean, SD) | | | | | | | |
| Coca, 2017 [37] | E3^j^ | E1^k^ | 2.86 ± 0.38 | 2.86 ± 0.38 | MD 0 LSD test: P>0.05 [95% CI: 0.86 lower to 0.86 higher] | Serious risk of bias | ⨁◯◯◯  Very low |
|  | E2^l^ | E1^k^ | 2.86 ± 0.38 | 2.86 ± 0.38 | MD 0 LSD test: P>0.05 [95% CI: 0.86 lower to 0.86 higher] |  |  |
| **Contamination Outcomes** | | | | | | | |
| *Overall contamination during doffing of PPE: Small sized contaminated patches (< 1 cm^2^), median* | | | | | | | |
| Suen, 2018 [41] | PPE1^q^ | PPE3^r^ | 5.00 | 7.00 | Difference in medians 2 lower ANOVA: PPE1 vs. PPE2 vs. PPE3 : p-value = 0.05 | High risk of bias | ⨁◯◯◯  Very low |
|  | PPE2^s^ | PPE3^r^ | 7.00 | 7.00 | Difference of medians 0 ANOVA: PPE1 vs. PPE2 vs. PPE3 : p-value = 0.05 | High risk of bias | ⨁◯◯◯  Very low |
| *Hair and head contamination during doffing of PPE: Small sized contaminated patches (< 1 cm^2^), median* | | | | | | | |
| Suen, 2018 [41] | PPE1^q^ | PPE3^r^ | 1.00 | 2.50 | Difference of medians 1.5 lower ANOVA: PPE1 vs. PPE2 vs. PPE3 : p-value = 0.68 | High risk of bias | ⨁◯◯◯  Very low |
|  | PPE2^s^ | PPE3^r^ | 2.00 | 2.50 | Difference of medians 0.5 lower ANOVA: PPE1 vs. PPE2 vs. PPE3 : p-value = 0.68 | High risk of bias | ⨁◯◯◯  Very low |
| *Neck (anterior) contamination during doffing of PPE: Small sized contaminated patches (< 1 cm^2^), median* | | | | | | | |
| Suen, 2018 [41] | PPE1^q^ | PPE3^r^ | 2.50 | 11.00 | Difference of medians 8.5 lower ANOVA: PPE1 vs. PPE2 vs. PPE3 : p-value = 0.095 | High risk of bias | ⨁◯◯◯  Very low |
|  | PPE2^s^ | PPE3^r^ | 5.00 | 11.00 | Difference of medians 6 lower ANOVA: PPE1 vs. PPE2 vs. PPE3 : p-value = 0.095 | High risk of bias | ⨁◯◯◯  Very low |
| *Neck (posterior) contamination during doffing of PPE: Small sized contaminated patches (< 1 cm^2^), median* | | | | | | | |
| Suen, 2018 [41] | PPE1^q^ | PPE3^r^ | 2.00 | 18.50 | Difference of medians 16.5 lower ANOVA: PPE1 vs. PPE2 vs. PPE3 : p-value = 0.824 | High risk of bias | ⨁◯◯◯  Very low |
|  | PPE2^s^ | PPE3^r^ | 1.00 | 18.50 | Difference of medians 17.5 lower ANOVA: PPE1 vs. PPE2 vs. PPE3 : p-value = 0.824 | High risk of bias | ⨁◯◯◯  Very low |
| *Overall contamination during doffing of PPE: Extra large sized contaminated patches (≥ 5cm^2^), median* | | | | | | | |
| Suen, 2018 [41] | PPE1^q^ | PPE3^r^ | 39.00 | 47.00 | Difference of medians 8 lower ANOVA: PPE1 vs. PPE2 vs. PPE3 : p-value = < 0.001 | High risk of bias | ⨁◯◯◯  Very low |
|  | PPE2^s^ | PPE3^r^ | 43.00 | 47.00 | Difference of medians 4 lower ANOVA: PPE1 vs. PPE2 vs. PPE3 : p-value = < 0.001 | High risk of bias | ⨁◯◯◯  Very low |
| *Hair and head contamination during doffing of PPE: Extra large sized contaminated patches (≥ 5cm^2^), median* | | | | | | | |
| Suen, 2018 [41] | PPE1^q^ | PPE3^r^ | 0.00 | 0.00 | Difference of medians 0 ANOVA: PPE1 vs. PPE2 vs. PPE3 : p-value = N/A | High risk of bias | ⨁◯◯◯  Very low |
|  | PPE2^s^ | PPE3^r^ | 17.00 | 0.00 | Difference of medians 17 higher ANOVA: PPE1 vs. PPE2 vs. PPE3 : p-value = N/A | High risk of bias | ⨁◯◯◯  Very low |
| *Neck (anterior) contamination during doffing of PPE: Extra large sized contaminated patches (≥ 5cm^2^), median* | | | | | | | |
| Suen, 2018 [41] | PPE1^q^ | PPE3^r^ | 0.00 | 24.00 | Difference of medians 24 lower ANOVA: PPE1 vs. PPE2 vs. PPE3 : p-value = N/A | High risk of bias | ⨁◯◯◯  Very low |
|  | PPE2^s^ | PPE3^r^ | 0.00 | 24.00 | Difference of medians 24 lower ANOVA: PPE1 vs. PPE2 vs. PPE3 : p-value = N/A | High risk of bias | ⨁◯◯◯  Very low |
| *Neck (posterior) contamination during doffing of PPE: Extra large sized contaminated patches (≥ 5cm^2^), median* | | | | | | | |
| Suen, 2018 [41] | PPE1^q^ | PPE3^r^ | 0.00 | 0.00 | Difference of medians 0 ANOVA: PPE1 vs. PPE2 vs. PPE3 : p-value = N/A | High risk of bias | ⨁◯◯◯  Very low |
|  | PPE2^s^ | PPE3^r^ | 0.00 | 0.00 | Difference of medians 0 ANOVA: PPE1 vs. PPE2 vs. PPE3 : p-value = N/A | High risk of bias | ⨁◯◯◯  Very low |
| *Overall contamination during doffing of PPE, any size, n/N (%)* | | | | | | | |
| Zamora, 2006 [39] | PAPR^t^ | E-RCP^u^ | 13/50 (26%) | 48/50 (96%) | RR 0.27 Mainland– Gart: p <0.001 [95% CI: 0.17 to 0.43]  701 fewer per 1,000 [95% CI: 547 fewer to 797 fewer] | High risk of bias | ⨁◯◯◯  Very low |
| *Face contamination during doffing of PPE, any size, n (%)* | | | | | | | |
| Zamora, 2006 [39] | PAPR^t^ | E-RCP^u^ | 0/50 | 2/50 (4%) | RR 0.20 Mainland– Gart: p=1 [95% CI: 0.01 to 4.06]  32 fewer per 1,000 [95% CI: 40 fewer to 123 more] | High risk of bias | ⨁◯◯◯  Very low |
| *Back of the head contamination during doffing of PPE, any size, n (%)* | | | | | | | |
| Zamora, 2006 [39] | PAPR^t^ | E-RCP^u^ | 0/50 | 0/50 | RR not estimable Mainland– Gart: undefined  0 fewer per 1,000 [95% CI: Undefined] | High risk of bias | ⨁◯◯◯  Very low |
| *Neck (anterior) contamination during doffing of PPE, any size, n (%)* | | | | | | | |
| Zamora, 2006 [39] | PAPR^t^ | E-RCP^u^ | 3/50 (6%) | 48/50 (96%) | RR 0.12 Mainland– Gart: p<0.001 [95% CI: 0.038 to 0.35]  845 fewer per 1,000 [95% CI: 621 fewer to 924 fewer] | High risk of bias | ⨁◯◯◯  Very low |
| *Neck (posterior) contamination during doffing of PPE, any size, n (%)* | | | | | | | |
| Zamora, 2006 [39] | PAPR^t^ | E-RCP^u^ | 1/50 (2%) | 9/50 (18%) | RR 0.13 Mainland– Gart: p=0.012 [95% CI: 0.017 to 0.98]  157 fewer per 1,000 [95% CI: 4 fewer to 177 fewer] | High risk of bias | ⨁◯◯◯  Very low |
| **Human Factor Outcomes (Protocol Deviations)** | | | | | | | |
| *Donning hood vs. Donning goggles/mask/surgical cap, n/N (%)* | | | | | | | |
| Casalino 2015 [36] | CTP-E^v^ | CTP-B^w^ | 3/30 (10%) | 3/30 (10%) | RR 1.00 p-value: NR  [95% CI: 0.22 to 4.56]  0 fewer per 1,000 [95% CI: 78 fewer to 356 more] | Serious risk of bias | ⨁◯◯◯  Very low |
|  | RTP-E^x^ | RTP-B^y^ | 2/30 (6.7%) | 4/30 (13.3%) | RR 0.50 p-value: NR  [95% CI: 0.099 to 2.53]  67 fewer per 1,000 [95% CI: 120 fewer to 204 more] |  |  |
| *Doffing hood vs. Doffing goggles/mask/surgical cap, n/N (%)* | | | | | | | |
| Casalino 2015 [36] | CTP-E^v^ | CTP-B^w^ | 5/30 (16.7%) | 5/30 (16.7%) | RR 1.00 p-value: NR  [95% CI: 0.32 to 3.10]  0 fewer per 1,000 [95% CI: 113 fewer to 350 more] | Serious risk of bias | ⨁◯◯◯  Very low |
|  | RTP-E^x^ | RTP-B^y^ | 5/30 (16.7%) | 1/30 (3.3%) | RR 5.00 p-value: NR  [95% CI: 0.62 to 40.29]  133 more per 1,000 [95% CI: 13 fewer to 1,000 more] |  |  |
| *Total errors, n/N (%)* | | | | | | | |
| Casalino 2015 [36] | CTP-E^v^ | CTP-B^w^ | 28/30 (93.3%) | 27/30 (90%) | RR 0.67 p-value: NR  [95% CI: 0.12 to 3.71]  297 fewer per 1,000 [95% CI: 792 fewer to 1,000 more] | Serious risk of bias | ⨁◯◯◯  Very low |
|  | RTP-E^x^ | RTP-B^y^ | 23/30 (76.7%) | 16/30 (53.3%) | RR 1.44 p-value: NR  [95% CI: 0.98 to 2.12]  235 more per 1,000 [95% CI: 11 fewer to 597 more] |  |  |
| *Total errors count (mean ± SD)* | | | | | | | |
| Casalino 2015 [36] | CTP-E^v^ | CTP-B^w^ | 4.5 ± 2.1 | 3.2 ± 1.5 | MD 1.3 lower p-value: NR [95% CI: 0.36 lower to 2.2 lower] | Serious risk of bias | ⨁◯◯◯  Very low |
|  | RTP-E^x^ | RTP-B^y^ | 3.3 ± 2.3 | 1.4 ± 1.6 | MD 1.9 lower p-value: NR  [95% CI: 0.88 lower to 2.92 lower] |  |  |
| *Total critical errors, n/N (%)* | | | | | | | |
| Casalino 2015 [36] | CTP-E^v^ | CTP-B^w^ | 26/30 (86.7%) | 27/30 (90%) | RR 0.96 p-value: NR  [95% CI: 0.80 to 1.16]  36 fewer per 1,000 [95% CI: 180 fewer to 144 more] | Serious risk of bias | ⨁◯◯◯  Very low |
|  | RTP-E^x^ | RTP-B^y^ | 2/30 (70%) | 13/30 (43.3%) | RR 1.62 p-value: NR  [95% CI: 1.01 to 2.59]  269 more per 1,000 [95% CI: 4 more to 689 more] |  |  |
| *Critical error count, mean ± SD* | | | | | | | |
| Casalino 2015 [36] | CTP-E^v^ | CTP-B^w^ | 3.7 ± 1.9 | 2.3 ± 1.5 | MD 1.4 lower p-value: NR  [95% CI: 0.52 lower to 2.29 lower] | Serious risk of bias | ⨁◯◯◯  Very low |
|  | RTP-E^x^ | RTP-B^y^ | 2.9 ± 2.3 | 1.2 ± 1.4 | MD 1.7 lower p-value: NR [95% CI: 0.72 lower to 2.68 lower] |  |  |
| *Overall deviation rate (%) during donning of PPE* | | | | | | | |
| Suen, 2018 [41] | PPE1^q^ | PPE3^r^ | 6.06% | 3.70% | RR 2.00 p-value: NR  [95% CI: 0.38 to 10.5]  37 more per 1,000 [95% CI: 23 fewer to 352 more] | High risk of bias | ⨁⨁◯◯  Low |
|  | PPE2^s^ | PPE3^r^ | 6.00% | 3.70% | RR 2.00 p-value: NR  [95% CI: 0.38 to 10.5]  37 more per 1,000 [95% CI: 23 fewer to 352 more] |  |  |
| *Deviation rate (%) during donning of hood* | | | | | | | |
| Suen, 2018 [41] | PPE1^q^ | PPE3^r^ | 20.00% | N/A | RR not estimable;  200 more per 1,000 p-value: NR [95% CI: Undefined] | High risk of bias | ⨁⨁◯◯  Low |
|  | PPE2^s^ | PPE3^r^ | 3.33% | N/A | RR not estimable;  33 more per 1,000 p-value: NR [95% CI: Undefined] |  |  |
| *Deviation rate (%) during donning of faceshield* | | | | | | | |
| Suen, 2018 [41] | PPE1^q^ | PPE3^r^ | 11.67% | 6.67% | RR 1.75 p-value: NR  [95% CI: 0.54 to 5.66]  50 more per 1,000 [95% CI: 31 fewer to 311 more] | High risk of bias | ⨁⨁◯◯  Low |
|  | PPE2^s^ | PPE3^r^ | 15.00% | 6.67% | RR 2.25 p-value: NR  [95% CI: 0.73 to 6.90]  83 more per 1,000 [95% CI: 18 fewer to 394 more] |  |  |
| *Overall deviation rate (%) during doffing of PPE* | | | | | | | |
| Suen, 2018 [41] | PPE1^q^ | PPE3^r^ | 2.95% | 3.52% | RR 1.00 p-value: NR  [95% CI: 0.15 to 6.87]  0 fewer per 1,000 [95% CI: 30 fewer to 207 more] | High risk of bias | ⨁⨁◯◯  Low |
|  | PPE2^s^ | PPE3^r^ | 9.48% | 3.52% | RR 3.00 p-value: NR  [95% CI: 0.63 to 14.3]  70 more per 1,000 [95% CI: 13 fewer to 468 more] |  |  |
| *Deviation rate (%) during doffing of hood* | | | | | | | |
| Suen, 2018 [41] | PPE1^q^ | PPE3^r^ | 5.00% | N/A | RR not estimable;  50 more per 1,000 p-value: NR [95% CI: Undefined] | High risk of bias | ⨁⨁◯◯  Low |
|  | PPE2^s^ | PPE3^r^ | 8.33% | N/A | RR not estimable;  83 more per 1,000 p-value: NR [95% CI: Undefined] |  |  |
| *Deviation rate (%) during doffing of faceshield* | | | | | | | |
| Suen, 2018 [41] | PPE1^q^ | PPE3^r^ | 6.67% | 10.00% | RR 0.67 p-value: NR [95% CI: 0.20 to 2.24]  33 fewer per 1,000 [95% CI: 80 fewer to 124 more] | High risk of bias | ⨁⨁◯◯  Low |
|  | PPE2^s^ | PPE3^r^ | 11.67% | 10.00% | RR 1.17 p-value: NR [95% CI: 0.42 to 3.26]  17 more per 1,000 [95% CI: 58 fewer to 226 more] |  |  |
| *Total donning errors, n/N (%)* | | | | | | | |
| Zamora, 2006 [39] | PAPR^t^ | E-RCP^u^ | 19/50 (38%) | 2/50 (4%) | RR 9.50 p-value: NR [95% CI: 2.33 to 38.70]  340 more per 1,000 [95% CI: 53 more to 1,000 more] | Some concerns | ⨁◯◯◯  Very low |
| *Total doffing errors, n/N (%)* | | | | | | | |
| Zamora, 2006 [39] | PAPR^t^ | E-RCP^u^ | 6/50 (12%) | 12/50 (24%) | RR 0.42 p-value: NR [95% CI: 0.17 to 1.03]  139 fewer per 1,000 [95% CI: 199 fewer to 7 more] | Some concerns | ⨁◯◯◯  Very low |
| *Error in application of goggles during donning, n/N (%)* | | | | | | | |
| Zamora, 2006 [39] | PAPR^t^ | E-RCP^u^ | 2/50 (4%) | 0/50 | RR not estimable;  40 more per 1,000 p-value: NR [95% CI: Undefined] | Some concerns | ⨁◯◯◯  Very low |
| *Failure to zip up coveralls or put hood over head during donning, n/N (%)* | | | | | | | |
| Zamora, 2006 [39] | PAPR^t^ | E-RCP^u^ | 1/50 (2%) | N/A | RR not estimable;  20 more per 1,000 p-value: NR [95% CI: Undefined] | Some concerns | ⨁◯◯◯  Very low |
| *Error in application of bouffant hair-cover during donning, n/N (%)* | | | | | | | |
| Zamora, 2006 [39] | PAPR^t^ | E-RCP^u^ | N/A | 1/50 (2%) | RR not estimable;  20 fewer per 1,000 p-value: NR [95% CI: Undefined] | Some concerns | ⨁◯◯◯  Very low |
| *Error in removal of face shield during doffing, n/N (%)* | | | | | | | |
| Zamora, 2006 [39] | PAPR^t^ | E-RCP^u^ | N/A | 1/50 (2%) | RR not estimable;  20 fewer per 1,000 p-value: NR [95% CI: Undefined] | Some concerns | ⨁◯◯◯  Very low |
| *Error in removal of hair-cover during doffing, n/N (%)* | | | | | | | |
| Zamora, 2006 [39] | PAPR^t^ | E-RCP^u^ | N/A | 2/50 (4%) | RR not estimable;  40 fewer per 1,000 p-value: NR [95% CI: Undefined] | Some concerns | ⨁◯◯◯  Very low |

Abbreviations: CI: Confidence interval; LSD, least significant difference test; MD, mean difference; NR, not reported, PAPR; powered air-purifying respirator; RR: relative risk; SD, standard deviation

1. Quality assessment of studies was completed using the ROBINS-I scale for observational/non-randomized studies and the Cochrane RoB 2 for randomized trials.. For the mannequin simulation study (Coca et al. 2015), quality assessment was performed under assumption that mannequin could be treated as a volunteer and humanized.
2. Condition A consisted of 32°C, 92% relative humidity
3. Ensemble 4 (E4): medical scrubs, socks, and rubber boots, impermeable coverall, Tyvek hood with an integrated splash-resistant surgical mask; rubber surgical apron, splash-resistant goggles, surgical nitrile inner gloves, heavy-duty nitrile outer gloves, N95 mask, a fluid-resistant surgical cap. The Tyvek hood provided the head and neck cover.
4. Ensemble 2 (E2): medical scrubs, socks, rubber boots with a mid-calf-length, disposable, fluid-resistant surgical gown, a polyethylene surgical apron, a face shield, disposable nitrile examination inner gloves, N95 mask, a fluid-resistant surgical cap. The cap provided some head covering, but the majority of head and neck skin remained exposed.
5. Ensemble 3 (E3): medical scrubs, socks, rubber boots with a Tyvek coverall, Tyvek hood with an integrated splash-resistant surgical mask; a rubber surgical apron, splash-resistant goggles, surgical nitrile inner gloves; heavy-duty nitrile outer gloves, a duckbill N95 filtering face piece respirator, and a fluid-resistant surgical cap. The Tyvek hood provided the head and neck cover.
6. Ensemble 1 (E1): medical scrubs, socks, rubber boots with a mid-calf-length, disposable, fluid-resistant surgical gown, a fluid-resistant 3-ply surgical mask, a disposable polyester lens face shield, disposable nitrile examination gloves. Head and neck skin was exposed.
7. Heat sensation (rated from −4 [very cold] to 4 [very hot]
8. Thermal comfort rated from −4 [very uncomfortable] to 4 [very comfortable])
9. Condition B consisted of 26°C, 80% relative humidity
10. Ensemble 3 (E3): medical scrubs, socks and rubber boots, Tychem QC highly impermeable coverall (DuPont), Médecins Sans Frontières (MSF) custom-made Tyvek (DuPont) hood with integrated splash-resistant surgical mask, rubber surgical apron, splash-resistant goggles, surgical nitrile inner gloves, heavy-duty nitrile outer gloves, duckbill N95 filtering face piece respirator and fluid-resistant surgical cap
11. Ensemble 2 (E2): medical scrubs, socks and rubber boots, Microgard coverall, Tyvek hood with integrated splash-resistant surgical mask, rubber surgical apron, splash-resistant goggles, surgical nitrile inner gloves, heavy-duty nitrile outer gloves, duckbill N95 filtering face piece respirator, fluid-resistant surgical cap
12. Ensemble 1 (E1): medical scrubs; socks and rubber boots; a midcalf-length disposable, fluid-resistant surgical gown, Performance Surgical Gown 7696C; polyethylene surgical apron, face shield, disposable nitrile examination inner gloves, duckbill N95 surgical filtering face piece respirator, and fluid-resistant surgical cap
13. Subjective thermal comfort was measured on a scale of 1 to 4 (where 1 = not uncomfortable and 4 = very uncomfortable)
14. Rate of perceived exertion was measured by using the OMNI 6-20 exertional scale
15. Breathing comfort was measured by using a scale of 1 to 7 (where 1 = no discomfort and 7 = intolerable discomfort)
16. Subjective wetness was measured by using a scale of 1 to 5 (where 1 = dry and 5 = soaked)
17. Hospital Authority Standard Ebola PPE set (PPE 1): a neck-to-ankle overall with an overlying water-resistant gown double and long nitrate gloves, boots, hood, disposable face shield and N95 respirator. Order of doffing: gloves, gown, boots, hood, N95.
18. HA isolation gown for routine patient care and performing AGPs (PPE3): pure cotton surgical scrub suit, appropriate size gowns and gloves and the known best-fitted respirator model (3 M 1860, 1860s and 1870). No hood. Order of doffing: gloves, gown, full face shield, cap, N95 respirator.
19. DuPont™ Tyvek®, Model 1422A (PPE2): head-to-ankle overall with a zipper on the front. In addition to a hood with elasticated facial opening, the whole outfit includes double gloves, boots, disposable face shield and an N95 respirator. A plastic apron was used to cover up the front zipper before use. Order of doffing: apron, hood, coverall/outer gloves, face shield, N95 respirator, boots, inner gloves.
20. PAPR (powered air-purifying respirator): Tyvek hood, Bouffant hair cover, Economy impact goggle, Air-mate breathing tube, face-shield, HEPA filter unit, N95 mask - any of several modes (8210, 1860s, PFR95, 7210, 695), Gloves (Non-latex, latex, latex surgical), Tyvek coveralls with hood, Tyvek boot covers, Astound impervious surgical gown
21. E-RCP (Enhanced respiratory and contact precautions) contains a bouffant hair cover, economy impact goggle, face-shield, N95 mask - any of several modes (8210, 1860s, PFR95, 7210, 695), gloves (Non-latex, latex), astound impervious surgical gown
22. CTP-E, Conventional training program with enhanced PPE. Enhanced PPE includes boots, a full-body impermeable suit, hood with a surgical cap and mask, double gloves, and an impermeable protection apron.
23. CTP-B, Conventional training program with basic PPE. Basic PPE (B-PPE) includes boots, goggles, surgical mask, and surgical cap, impermeable surgical gown, double gloves, and an impermeable apron.
24. RTP-E, Reinforced training program with enhanced PPE. Enhanced PPE includes boots, a full-body impermeable suit, hood with a surgical cap and mask, double gloves, and an impermeable protection apron.
25. RTP-B, Reinforced training program with basic PPE. Basic PPE (B-PPE) includes boots, goggles, surgical mask, and surgical cap, impermeable surgical gown, double gloves, and an impermeable apron.
26. 95% confidence intervals were calculated using standard frequentist statistics.

**Supplementary file 10 Table 2.** KQ6 Summary of Findings (Should health workers providing direct care or indirect care to patients with EBOD or MARD and using eye protection (goggles /face shield) wear them under versus over the head and neck covering?)

| ***Study details*** | ***Intervention***  ***(*Wearing (goggles /face shield) under the head/neck covering)** | ***Comparator(s)***  **(Wearing eye protection (goggles/face shield) over the head /neck covering)** | ***Outcome in intervention group*** | ***Outcome in control group*** | ***Effect Magnitude, Reported Statistical Test [Calculated 95% CI^n^]*** | ***Quality Assessment^a^*** | ***GRADE*** | ***Notes*** |
| --- | --- | --- | --- | --- | --- | --- | --- | --- |
| **Contamination outcomes** | | | | | | | | |
| *Number of participants (n/N, %) with small fluorescent patches after various personal protective equipment (PPE) protocols* | | | | | | | | |
| Chughtai, 2018 [40] | WHO, coverall and N95^b^ | CDC, coverall and PAPR^c^ | 0/3 | 0/3 | 0 more per 1,000 p-value: NR [95% CI: Undefined] | High risk of bias | ⨁◯◯◯  Very low | The hood used in the WHO (coverall, N95) protocol is donned after the face shield. In all other doffing sequences, the face shield is donned after the hood and removed first. |
|  |  | CDC, coverall and N95^d^ |  | 1/3 (33%) | 333 less per 1,000 p-value: NR [95% CI: Undefined] |  |  |  |
|  |  | ECDC, coverall and N95^e^ |  | 0/3 | 0 more per 1,000 p-value: NR [95% CI: Undefined] |  |  |  |
|  |  | Health Canada, gown and N95^f^ |  | 1/3 (33%) | 333 less per 1,000 p-value: NR [95% CI: Undefined] |  |  |  |
|  |  | NC, coverall and N95^g^ |  | 0/3 | 0 more per 1,000 p-value: NR [95% CI: Undefined] |  |  |  |
|  |  | NSW DoH CEC, gown and PAPR^h^ |  | 0/3 | 0 more per 1,000 p-value: NR [95% CI: Undefined] |  |  |  |
|  |  | NSW DoH CEC, gown and N95^i^ |  | 0/3 | 0 more per 1,000 p-value: NR [95% CI: Undefined] |  |  |  |
|  |  | MSF, coverall and N95^j^ |  | 0/3 | 0 more per 1,000 p-value: NR [95% CI: Undefined] |  |  |  |
|  |  | WHO, gown and N95^k^ |  | 0/3 | 0 more per 1,000 p-value: NR [95% CI: Undefined] |  |  |  |
| *Number of participants (n/N, %) with large fluorescent patches after various personal protective equipment (PPE) protocols* | | | | | | | | |
| Chughtai, 2018 [40] | WHO, coverall and N95^b^ | CDC, coverall and PAPR^c^ | 1/3 (33%) | 0/3 | 333 more per 1,000 p-value: NR [95% CI: Undefined] | High risk of bias | ⨁◯◯◯  Very low | The hood used in the WHO (coverall, N95) protocol is donned after the face shield. In all other doffing sequences, the face shield is donned after the hood and removed first. |
|  |  | CDC, coverall and N95^d^ |  | 0/3 | 333 more per 1,000 p-value: NR [95% CI: Undefined] |  |  |  |
|  |  | ECDC, coverall and N95^e^ |  | 0/3 | 333 more per 1,000 p-value: NR [95% CI: Undefined] |  |  |  |
|  |  | Health Canada, gown and N95^f^ |  | 0/3 | 333 more per 1,000 p-value: NR [95% CI: Undefined] |  |  |  |
|  |  | NC, coverall and N95^g^ |  | 1/3 (33%) | 0 more per 1,000 p-value: NR [95% CI: Undefined] |  |  |  |
|  |  | NSW DoH CEC, gown and PAPR^h^ |  | 0/3 | 333 more per 1,000 p-value: NR [95% CI: Undefined] |  |  |  |
|  |  | NSW DoH CEC, gown and N95^i^ |  | 0/3 | 333 more per 1,000 p-value: NR [95% CI: Undefined] |  |  |  |
|  |  | MSF, coverall and N95^j^ |  | 0/3 | 333 more per 1,000 p-value: NR [95% CI: Undefined] |  |  |  |
|  |  | WHO, gown and N95^k^ |  | 0/3 | 333 more per 1,000 p-value: NR [95% CI: Undefined] |  |  |  |
| *Overall contamination during doffing of PPE: Small sized contaminated patches (< 1 cm^2^), median* | | | | | | | | |
| Suen 2018 [41] | PPE2^l^ | PPE1^m^ | 7.00 | 5.00 | Difference of medians 2 higher p-value: NR [95% CI: Undefined] | High risk of bias | ⨁◯◯◯  Very low | None |
| *Hair and head contamination during doffing of PPE: Small sized contaminated patches (< 1 cm^2^), median* | | | | | | | | |
| Suen 2018 [41] | PPE2^l^ | PPE1^m^ | 2.00 | 1.00 | Difference of medians 1 higher p-value: NR [95% CI: Undefined] | High risk of bias | ⨁◯◯◯  Very low | None |
| *Neck (anterior) contamination during doffing of PPE: Small sized contaminated patches (< 1 cm^2^), median* | | | | | | | | |
| Suen 2018 [41] | PPE2^l^ | PPE1^m^ | 5.00 | 2.50 | Difference of medians 2.5 higher p-value: NR [95% CI: Undefined] | High risk of bias | ⨁◯◯◯  Very low | None |
| *Neck (posterior) contamination during doffing of PPE: Small sized contaminated patches (< 1 cm^2^), median* | | | | | | | | |
| Suen 2018 [41] | PPE2^l^ | PPE1^m^ | 1.00 | 2.00 | Difference of medians 1 lower p-value: NR [95% CI: Undefined] | High risk of bias | ⨁◯◯◯  Very low | None |
| *Overall contamination during doffing of PPE: Extra large sized contaminated patches (≥ 5cm^2^), median* | | | | | | | | |
| Suen 2018 [41] | PPE2^l^ | PPE1^m^ | 43.00 | 39.00 | Difference of medians 4 higher p-value: NR [95% CI: Undefined] | High risk of bias | ⨁◯◯◯  Very low | None |
| *Hair and head contamination during doffing of PPE: Extra large sized contaminated patches (≥ 5cm^2^), median* | | | | | | | | |
| Suen 2018 [41] | PPE2^l^ | PPE1^m^ | 17.00 | 0.00 | Difference of medians 17 higher p-value: NR [95% CI: Undefined] | High risk of bias | ⨁◯◯◯  Very low | None |
| *Neck (anterior) contamination during doffing of PPE: Extra large sized contaminated patches (≥ 5cm^2^), median* | | | | | | | | |
| Suen 2018 [41] | PPE2^l^ | PPE1^m^ | 0.00 | 0.00 | Difference of medians 0 p-value: NR [95% CI: Undefined] | High risk of bias | ⨁◯◯◯  Very low | None |
| *Neck (posterior) contamination during doffing of PPE: Extra large sized contaminated patches (≥ 5cm^2^), median* | | | | | | | | |
| Suen 2018 [41] | PPE2^l^ | PPE1^m^ | 0.00 | 0.00 | Difference of medians 0 p-value: NR [95% CI: Undefined] | High risk of bias | ⨁◯◯◯  Very low | None |
| **Human Factors (Protocol Deviations)** | | | | | | | | |
| *Overall deviation rate (%) during donning of PPE* | | | | | | | | |
| Suen 2018 [41] | PPE2^l^ | PPE1^m^ | 6.00 | 6.06 | RR 1.00 p-value: NR  [95% CI: 0.26 to 3.81]  0 fewer per 1,000  [95% CI: 45 fewer to 170 more] | High risk of bias | ⨁◯◯◯  Very low | None |
| *Deviation rate (%) during donning of hood* | | | | | | | | |
| Suen 2018 [41] | PPE2^l^ | PPE1^m^ | 3.33 | 20.00 | RR 0.17 p-value: NR  [95% CI: 0.039 to 0.71]  166 fewer per 1,000  [95% CI: 58 fewer to 192 fewer] | High risk of bias | ⨁◯◯◯  Very low | None |
| *Deviation rate (%) during donning of faceshield* | | | | | | | | |
| Suen 2018 [41] | PPE2^l^ | PPE1^m^ | 15.00 | 11.67 | RR 1.29 p-value: NR  [95% CI: 0.51 to 3.22]  34 more per 1,000  [95% CI: 57 fewer to 259 more] | High risk of bias | ⨁◯◯◯  Very low | None |
| *Overall deviation rate (%) during doffing of PPE* | | | | | | | | |
| Suen 2018 [41] | PPE2^l^ | PPE1^m^ | 9.48 | 2.95 | RR 3.00 p-value: NR  [95% CI: 0.63 to 14.30]  59 more per 1,000  [95% CI: 11 fewer to 392 more] | High risk of bias | ⨁◯◯◯  Very low | None |
| *Deviation rate (%) during doffing of hood* | | | | | | | | |
| Suen 2018 [41] | PPE2^l^ | PPE1^m^ | 8.33 | 5.00 | RR 1.67 p-value: NR  [95% CI: 0.42 to 6.66]  33 more per 1,000  [95% CI: 29 fewer to 283 more] | High risk of bias | ⨁◯◯◯  Very low | None |
| *Deviation rate (%) during doffing of faceshield* | | | | | | | | |
| Suen 2018 [41] | PPE2^l^ | PPE1^m^ | 11.67 | 6.67 | RR 1.75 p-value: NR  [95% CI: 0.54 to 5.66]  50 more per 1,000  [95% CI: 31 fewer to 311 more] | High risk of bias | ⨁◯◯◯  Very low | None |

CI: Confidence interval; MD, mean difference; NR, not reported, PAPR; powered air-purifying respirator; RR: relative risk; SD, standard deviation

1. Quality assessment of studies was completed using the Cochrane RoB 2 for randomized trials.
2. Word Health Organization (WHO) recommended protocol from 2014 rapid advice guideline (with coverall). This protocol is different than the others, as it recommends wearing the face shield before the hood and removing the hood before the face shield. Other notable differences in this personal protective equipment (PPE) donning/ doffing protocol tested include: using coveralls/face shields, trained observer only for doffing instructions.
3. Centers for Disease Control and Prevention (CDC), coverall and PAPR. Notable differences in this personal protective equipment (PPE) donning/ doffing protocol tested include: using coveralls/face shields, trained observer with partial assisted doffing.
4. Centers for Disease Control and Prevention (CDC), coverall and N95. Notable differences in this personal protective equipment (PPE) donning/ doffing protocol tested include: using coveralls/face shields, trained observer with partial assisted doffing.
5. European Centre for Disease Prevention and Control (ECDC), coverall and N95. Notable differences in this personal protective equipment (PPE) donning/ doffing protocol tested include: using coveralls/face shields, assisted doffing by active assistant.
6. Health Canada, gown and N95. Notable differences in this personal protective equipment (PPE) donning/ doffing protocol tested include: using gowns, face shields, removing gown/coverall before face shield, trained observer with partial assisted doffing.
7. North Carolina (NC), coverall and N95. Notable differences in this personal protective equipment (PPE) donning/ doffing protocol tested include: using coveralls/face shields, removing outer gloves before apron, removing gown/coverall before face shield, trained observer only for doffing instructions.
8. New South Wales (NSW), Clinical Excellence Commission (CEC), gown and PAPR. Notable differences in this personal protective equipment (PPE) donning/ doffing protocol tested include: using gowns/face shields, removing shoe covers after apron and before all other PPE, trained observer only for doffing instructions.
9. New South Wales (NSW), Clinical Excellence Commission (CEC), gown and N95. Notable differences in this personal protective equipment (PPE) donning/ doffing protocol tested include: using gowns/face shields, removing shoe covers after apron and before all other PPE, trained observer only for doffing instructions.
10. Médecins Sans Frontières (MSF), coverall and N95. Notable differences in this personal protective equipment (PPE) donning/ doffing protocol tested include: using coveralls/face shields/goggles, removing outer gloves before apron, trained observer only for doffing instructions.
11. Word Health Organization (WHO) recommended protocol from 2014 rapid advice guideline (with gown): Notable differences in this personal protective equipment (PPE) donning/ doffing protocol tested include: using gowns/face shields, trained observer only for doffing instructions.
12. DuPont™ Tyvek®, Model 1422A (PPE2): head-to-ankle overall with a zipper on the front. The whole outfit includes double gloves, boots, disposable face shield and an N95 respirator. A plastic apron was used to cover up the front zipper before use. Order of doffing: apron, hood, coverall/outer gloves, face shield, N95 respirator, boots, inner gloves.
13. Hospital Authority Standard Ebola PPE set (PPE 1): a neck-to-ankle overall with an overlying water-resistant gown double and long nitrate gloves, boots, hood, disposable face shield and N95 respirator. Order of doffing: gloves, gown, boots, hood, N95.
14. 95% confidence intervals were calculated using standard frequentist statistics.

**Supplementary file 10 Table 3**. KQ10 Summary of Findings (Should health workers to patients with Ebola or Marburg disease be sprayed versus not sprayed during doffing of personal protective equipment?)

| **Study details** | **Intervention**  **(Spraying with chlorine solution prior to removing PPE)** | **Comparator(s)**  **(No spraying with chlorine solution prior to removing PPE)** | **Outcome in intervention group** | **Outcome in control group** | ***Effect Magnitude, Reported Statistical Test [Calculated 95% CI^d^]*** | **Quality Assessment^a^** | **GRADE** | **Notes** |
| --- | --- | --- | --- | --- | --- | --- | --- | --- |
| ***Contamination Outcomes:* Transfer of Φ6 or MS2** | | | | | | | | |
| *Transfer of* Φ6 *(n/N, %) to inner gloves, hands, face or scrubs following doffing protocol* | | | | | | | | |
| Casanova, 2016 [43] | Doffing protocol with extra glove sanitization with sprayed hypochlorite sanitizer^b^ | Doffing protocol with alcohol-based hand rub for extra glove sanitization^c^ | 0/5 (0%) | 0/10 (0%) | 0 more per 1,000  p-value: NR  [95% CI: Undefined] | Moderate risk of bias | ⨁◯◯◯  Very low | Hypochlorite spray or ABHR use for extra hand sanitization was the only alteration between doffing protocols |
| *Transfer of MS2 (n/N, %) to inner gloves following doffing protocol* | | | | | | | | |
| Casanova, 2016 [43] | Doffing protocol with extra glove sanitization with sprayed hypochlorite sanitizer^b^ | Doffing protocol with alcohol-based hand rub for extra glove sanitization^c^ | 0/5 (0%) | 8/10 (80%) | 800 less per 1,000  p-value: NR  [95% CI: Undefined] | Moderate risk of bias | ⨁◯◯◯  Very low | Hypochlorite spray or ABHR use for extra hand sanitization was the only alteration between doffing protocols |
| *Transfer of MS2 (n/N, %) to hands following doffing protocol* | | | | | | | | |
| Casanova, 2016 [43] | Doffing protocol with extra glove sanitization with sprayed hypochlorite sanitizer^b^ | Doffing protocol with alcohol-based hand rub for extra glove sanitization^c^ | 1/5 (20%) | 0/10 (0%) | 200 more per 1,000  p-value: NR  [95% CI: Undefined] | Moderate risk of bias | ⨁◯◯◯  Very low | Hypochlorite spray or ABHR use for extra hand sanitization was the only alteration between doffing protocols |
| *Transfer of MS2 (n/N, %) to face following doffing protocol* | | | | | | | | |
| Casanova, 2016 [43] | Doffing protocol with extra glove sanitization with sprayed hypochlorite sanitizer^b^ | Doffing protocol with alcohol-based hand rub for extra glove sanitization^c^ | 0/5 (0%) | 0/10 (0%) | 0 more per 1,000  p-value: NR  [95% CI: Undefined] | Moderate risk of bias | ⨁◯◯◯  Very low | Hypochlorite spray or ABHR use for extra hand sanitization was the only alteration between doffing protocols |
| *Transfer of MS2 (n/N, %) to scrubs following doffing protocol* | | | | | | | | |
| Casanova, 2016 [43] | Doffing protocol with extra glove sanitization with sprayed hypochlorite sanitizer^b^ | Doffing protocol with alcohol-based hand rub for extra glove sanitization^c^ | 1/5 (20%) | 0/10 (0%) | 200 more per 1,000  p-value: NR  [95% CI: Undefined] | Moderate risk of bias | ⨁◯◯◯  Very low | Hypochlorite spray or ABHR use for extra hand sanitization was the only alteration between doffing protocols |
| ***IgG antibody against Ebola Virus [as an indicator of previous infection]*** | | | | | | | | |
| Houlihan, 2017 [42] | PPE removal with chlorine spray | PPE removal without chlorine spray | 33/132 (25%) | 7/98 (7%) | RR 3.52, p-value: NR [95% CI: 1.62 to 7.58];  180 more per 1,000  [95% CI: 44 more to 470 more] | Serious risk of bias | ⨁◯◯◯  Very low | Authors did not include PPE removal in their analysis since method of PPE removal was almost collinear with HW role. Almost all HWs in clinical roles were sprayed with chlorine and had assistance, and almost all HWs in laboratory roles were not sprayed and removed PPE without assistance. |

CI: Confidence interval; NR, not reported, PAPR; powered air-purifying respirator; RR: relative risk; SD, standard deviation

1. Quality assessment of studies was completed using the ROBINS-I tool for non-randomized studies.
2. For each glove sanitizing step in steps 1-12 of the 16-step doffing protocol, liquid hypochlorite (Fuzion Healthcare Disinfectant, Clorox Co., Pleasanton, CA) at a concentration of 1850 ppm was sprayed onto gloves. The final hand hygiene steps (Steps 13 and 16) that called for sanitizing bare hands were performed using ABHR.
3. For each glove sanitizing step in steps 1-12 of the 16-step doffing protocol, ABHR (70% ethanol gel) was applied to gloves. The final hand hygiene steps (Steps 13 and 16) that called for sanitizing bare hands were performed using ABHR.
4. 95% confidence intervals were calculated using standard frequentist statistics.
