# Supplementary material for: Infection prevention and control measures for Ebola and Marburg disease: a series of rapid reviews
Source: BMJ Open. 2026 Jul 9;16(7):e115610. doi: 10.1136/bmjopen-2025-115610 (PMC13358256; doi:10.1136/bmjopen-2025-115610)
Supplement: Supplementary file 11 [file bmjopen-2025-115610.reviewer_comments.pdf]

PEER REVIEW HISTORY

BMJ Open publishes all reviews undertaken for accepted manuscripts. Reviewers are asked to complete a checklist review form and are provided with free text boxes to elaborate on their assessment. These free text comments are reproduced below.

This paper was submitted to another journal from BMJ but declined for publication following peer review. The authors addressed the reviewers' comments and submitted the revised paper to BMJ Open. The paper was subsequently accepted for publication at BMJ Open.

ARTICLE DETAILS

Title (Provisional)

Infection prevention and control measures for Ebola and Marburg disease: A Series of Rapid Reviews

Authors

Shaver, Nicole; Pham, Ba; Vyas, Niyati; Collins, Erin; Bennett, Alexandria; Beck, Andrew; Skidmore, Becky; Grossman, Maura R; Cormack, Gordon V; Mishra, Sharmistha; Chan, Adrienne K.; Moher, David; Brouwers, Melissa; Tricco, Andrea C; Willet, Victoria; Dixit, Devika; Baller, April; Little, Julian

---

VERSION 1 - REVIEW

**Reviewer**	**1**
**Name**	**Reviewer 1**
**Affiliation**	
**Date**	**11-Mar-2025**
**COI**	

Comments not published pending reviewer's consent.

**Reviewer**	**2**
**Name**	**Reviewer 2**
**Affiliation**	
**Date**	**11-Mar-2025**
**COI**	

Comments not published pending reviewer's consent

VERSION 2 - REVIEW

**Reviewer**	**3**
**Name**	**Reviewer 3**
**Affiliation**	
**Date**	**19-Nov-2025**
**COI**	

Comments not published pending reviewer's consent.

**Reviewer**	**4**
**Name**	**Reviewer 4**
**Affiliation**	
**Date**	**19-Nov-2025**
**COI**	

Comments not published pending reviewer's consent

VERSION 3 - REVIEW

**Reviewer**	**5**
**Name**	**Rugarabamu, Sima**
**Affiliation**	**Muhimbili National Hospital**
**Date**	**05-Feb-2026**
**COI**	**None**

Overall Assessment

This manuscript presents a thorough and well-conducted series of rapid reviews aimed at informing the 2023 WHO infection prevention and control (IPC) guideline for Ebola (EBOD) and Marburg (MARD) disease. The topic is highly relevant given the high case-fatality rates of these viruses and the significant risk they pose to healthcare workers.

You clearly report a very limited evidence base, with only nine studies meeting inclusion criteria across 14 key questions, and appropriately grade the certainty of the evidence as low or very low. The identification of major research gaps is particularly useful for guiding future

studies. Overall, your manuscript is well-organized and follows recognized reporting standards.

Strengths

Relevance

• Your study addresses a critical need for evidence-based IPC guidance for filovirus outbreaks. Findings are directly relevant for protecting healthcare workers and supporting WHO guideline development.

Strong Methodology

• Pre-registered protocol in PROSPERO enhances transparency.

• AI-assisted screening (CAL®) improved efficiency without compromising rigor.

• Risk of bias was assessed using RoB-2 and ROBINS-I, and evidence certainty was evaluated using GRADE.

• PRISMA standards were followed, and the inclusion of a guideline development group with a patient representative strengthened the review process.

Transparent Reporting

• You openly acknowledge “empty reviews” and low-certainty evidence.

• You provide clear contextual summaries when comparative data were lacking.

Practical Implications

• Your manuscript clearly highlights gaps in the evidence base.

• It offers practical guidance for future research and policy development.

Points to Address

1. Manuscript Length and Flow

• The results sections for each key question are quite detailed; some material could be moved to supplementary files to improve readability.

• Some sentences are long and could be simplified to make the text more accessible.

2. Supplementary Materials

• Supplementary File 4 (Eligibility Criteria) is detailed but could benefit from a short summary in the main methods section.

3. Discussion of “Empty Reviews”

• You note that narrow eligibility criteria contributed to empty reviews. Briefly discussing the balance between methodological rigor and inclusivity would strengthen the interpretation.

4. Ethical Statement

• The statement “No ethical approval was required” could be slightly expanded, e.g., “as this study involved synthesis of previously published data.”

Other issues to address

• Abstract: Ensure abbreviations are consistent (EBOD/MARD vs. EVD/MVD).

• Tables: Define all abbreviations in footnotes or main text.

• Formatting: Remove placeholder page numbers (“1 2 3 4 5…60”).

• Grammar/Clarity:

o P.6: “spraying of HWs during PPE doffing” → “spraying of healthcare workers during PPE doffing.”

o P.8: “AI-supported search” → “AI-supported search.”

o Ensure consistent use of “healthcare worker(s)” throughout.

Summary

This is a solid and timely evidence synthesis that clearly maps the limited available literature on IPC for Ebola and Marburg disease. my recommended revisions mainly focus on improving clarity, shortening lengthy sections, and ensuring the supplementary materials are fully integrated. Your manuscript provides a strong evidence base for WHO guidelines and identifies clear directions for future research.

**Reviewer**	**6**
**Name**	**Torgersen, Jessie**
**Affiliation**	**University of Pennsylvania, Medicine**
**Date**	**13-Mar-2026**
**COI**	**None**

Shaver and colleagues present a comprehensive series of rapid reviews with the intention of addressing 14 key questions related to prevention of transmission of Ebola and Marburg diseases. The manuscript presents a systematic and transparent approach for PICO question development, rapid reviews, data abstraction and synthesis using the standardized GRADE approach to assess the quality of the evidence. The authors ultimately identified nine studies to address four of the 14 key questions. While this work builds upon a prior rapid review in 2014 with the purpose of informing updates in best practices, the authors find significant remaining knowledge gaps in the prevention of transmission for viruses with significant mortality.

This is an incredibly well-written manuscript with excellent detail to allow for reproducibility and subsequent review updates. There are many strengths of this work the authors appropriately point out in the manuscript, including rigor of literature review and systematic,

standardized methodology. I applaud the authors for including a patient representative with lived experience in the consulting team and highlighting the need for further investigation to ensure interventions are both evidence-based and culturally sensitive. The work is very important overall to the field as it highlights the limited strength of evidence of current practices, identified ongoing knowledge gaps in prevention of transmission and may ultimately inform future data collection or pragmatic trials when outbreaks occur.

There are minor points that could be further clarified for the audience:

- Further comment on the performance characteristics of the CAP tool in scientific rapid review. The use of the CAP tool in literature review is an exciting application of machine learning. However, it is not clear to me how accurate this methodology is in the systematic review of scientific literature. The authors note this tool has been used in prior systematic reviews, yet indicate additional literature review identified additional studies the CAP tool did not (e.g., 22 studies KQ4/T Theme 2 in the supplement flow diagram).

- Further clarify the training and validation steps for the CAP Tool. Appendix 3 provides a figure of the CAP Tool supervised training; however, it is unclear how performance of the training and validation groups were evaluated as this figure suggests this is a continuous training loop.

- Provide a statement summarizing the publications not available after the 14-day window or note absence of accessible PDFs as a limitation. The authors appropriately list all reasons for exclusion of publications for each KQ and there are a number that were excluded due to "PDF not available", "PDF unavailable", or "full text unavailable" with titles that were seemingly highly relevant.

- Clarify in the Methods if English-only texts were included. The methods indicate the literature search included text of any language, yet many non-English studies were excluded in the supplemental flow diagrams.

**Reviewer**	**7**
**Name**	**Muir, Peter**
**Affiliation**	**UK Health Security Agency South West, Clinical Network Laboratory**
**Date**	**17-Mar-2026**
**COI**	**None**

This series of rapid reviews describes the methods and outcomes of a process to define the evidence (or lack of) relating to IPC guidelines for Ebola and Marburg disease. The evidence synthesis is useful, primary through documenting the paucity of evidence in this area, and the identification of significant gaps in knowledge which should be targeted by future research, which is highlighted in the Discussion. Nonetheless the review has identified data

which has contributed to the development of guidelines. The limitations of the study are discussed and relate mainly to the lack of evidence, the narrow eligibility criteria of the Key Questions, and uncertainties around the rapid review methodology, including the use of AI for the data search.

VERSION 3 - AUTHOR RESPONSE

Reviewer	Reviewer Comment	Response
Reviewer #5	Overall Assessment<br>This manuscript presents a thorough and well-conducted series of rapid reviews aimed at informing the 2023 WHO infection prevention and control (IPC) guideline for Ebola (EBOD) and Marburg (MARD) disease. The topic is highly relevant given the high case-fatality rates of these viruses and the significant risk they pose to healthcare workers.<br>You clearly report a very limited evidence base, with only nine studies meeting inclusion criteria across 14 key questions, and appropriately grade the certainty of the evidence as low or very low. The identification of major research gaps is particularly useful for guiding future studies. Overall, your manuscript is well-organized and follows recognized reporting standards.<br>Strengths<br>Relevance<br>• Your study addresses a critical need for evidence-based IPC guidance for filovirus outbreaks. Findings are directly relevant for protecting healthcare workers and supporting WHO guideline development.<br>Strong Methodology<br>• Pre-registered protocol in PROSPERO enhances transparency.<br>• AI-assisted screening (CAL®) improved efficiency without compromising rigor.<br>• Risk of bias was assessed using RoB-2 and ROBINS-I, and evidence certainty was evaluated using GRADE.	We thank the reviewer for their thoughtful evaluation and feedback on our manuscript. We are pleased that the relevance of the work and its practical implications for IPC guideline development were recognized. We have carefully considered all comments raised and addressed each below.

	• PRISMA standards were followed, and the inclusion of a guideline development group with a patient representative strengthened the review process.<br>Transparent Reporting<br>• You openly acknowledge "empty reviews" and low-certainty evidence.<br>• You provide clear contextual summaries when comparative data were lacking.<br>Practical Implications<br>• Your manuscript clearly highlights gaps in the evidence base.<br>• It offers practical guidance for future research and policy development.	
Reviewer #5	1. Manuscript Length and Flow<br>• The results sections for each key question are quite detailed; some material could be moved to supplementary files to improve readability.<br>• Some sentences are long and could be simplified to make the text more accessible.	We thank the reviewer for these suggestions. Regarding the level of detail in the results sections for each key question, we have retained the current structure in response to feedback from other reviewers who highlighted the thoroughness and reproducibility of the reporting as a strength of the manuscript. Detailed results within our summary of findings tables and GRADE tables have been moved to our supplementary files.<br><br>With respect to sentence complexity, we have reviewed the manuscript throughout and made revisions to simplify phrasing and improve accessibility where possible.
Reviewer #5	2. Supplementary Materials<br>• Supplementary File 4 (Eligibility Criteria) is detailed but could benefit from a short summary in the main methods section.	We have revised the methods section to include a structured PICOS summary of the eligibility criteria, covering populations, interventions (organized by thematic area), comparators, outcomes, and eligible study designs. Full eligibility criteria remain available in Supplementary File 4.
Reviewer #5	3. Discussion of "Empty Reviews"<br>• You note that narrow eligibility criteria contributed to empty reviews. Briefly discussing the balance between methodological rigor and inclusivity would strengthen the interpretation.	Thank you. We now more explicitly address the balance between methodological rigour and inclusivity in our limitations section, in the context of empty reviews. Specifically, we have added a discussion of the structural challenges inherent to generating comparative evidence during active filovirus outbreaks and elaborated on the tension between strict eligibility criteria and

		inclusivity. We also discuss indirect evidence as a potential avenue for future reviews.
Reviewer #5	4. Ethical Statement<br>• The statement "No ethical approval was required" could be slightly expanded, e.g., "as this study involved synthesis of previously published data."	We have added this to our statement to clarify why we were not required to seek additional ethics approval.
Reviewer #5	Other issues to address<br>• Abstract: Ensure abbreviations are consistent (EBOD/MARD vs. EVD/MVD).<br>• Tables: Define all abbreviations in footnotes or main text.<br>• Formatting: Remove placeholder page numbers ("1 2 3 4 5…60").<br>• Grammar/Clarity:<br>o P.6: "spraying of HWs during PPE doffing" → "spraying of healthcare workers during PPE doffing."<br>o P.8: "AI-supported search" → "AI-supported search."<br>o Ensure consistent use of "healthcare worker(s)" throughout.	We thank the reviewer for these helpful editorial suggestions.<br><br>We have now ensured we have defined all abbreviations in table in footnotes, and removed placeholder numbers. We have also corrected "AI-supported search", and reviewed the manuscript to ensure consistent use of "health workers" (HWs) terminology throughout in alignment with WHO language.<br><br>We would like to clarify the reasons behind inconsistency in the EBOD/MARD vs. EVD/MVD abbreviations, most notably in the key questions and PICO criteria.<br><br>The original KQs and PICO questions were formally developed and registered in PROSPERO using the terms EVD (Ebola Virus Disease) and MVD (Marburg Virus Disease). A WHO nomenclature change to EBOD and MARD occurred during the writing of the guideline, after the KQs and protocol had been finalised, and therefore the updated abbreviations are used throughout the main manuscript text and in the final WHO guideline. We have retained EVD and MVD in the KQs and PICO tables to accurately reflect the registered protocol and the terms under which the review was formally conducted, as retrospectively amending these would misrepresent the original scope and registration of the review. We have noted this explicitly in the methods section (Registration and Reporting) and confirmed that the original abbreviations were not used in the search strategy, and therefore this nomenclature change did not influence any aspect of the search or study selection.
Reviewer #6	Shaver and colleagues present a comprehensive series of rapid reviews with the intention of addressing 14 key questions related to prevention of transmission of	We sincerely thank Reviewer 2 for their generous and thoughtful assessment of our manuscript. We are pleased that the methodological rigour and practical importance of this work were recognized.

	Ebola and Marburg diseases. The manuscript presents a systematic and transparent approach for PICO question development, rapid reviews, data abstraction and synthesis using the standardized GRADE approach to assess the quality of the evidence. The authors ultimately identified nine studies to address four of the 14 key questions. While this work builds upon a prior rapid review in 2014 with the purpose of informing updates in best practices, the authors find significant remaining knowledge gaps in the prevention of transmission for viruses with significant mortality.<br><br>This is an incredibly well-written manuscript with excellent detail to allow for reproducibility and subsequent review updates. There are many strengths of this work the authors appropriately point out in the manuscript, including rigor of literature review and systematic, standardized methodology. I applaud the authors for including a patient representative with lived experience in the consulting team and highlighting the need for further investigation to ensure interventions are both evidence-based and culturally sensitive. The work is very important overall to the field as it highlights the limited strength of evidence of current practices, identified ongoing knowledge gaps in prevention of transmission and may ultimately inform future data collection or pragmatic trials when outbreaks occur.	We also agree and support the inclusion of patient representatives with lived experience. We have considered and addressed all comments below.
Reviewer #6	- Further comment on the performance characteristics of the CAP tool in scientific rapid review. The use of the CAP tool in literature review is an exciting application of machine learning. However, it is not clear to me how accurate this methodology is in the systematic review of scientific	We have expanded the methods section to provide additional detail on the CAL® tool's operating characteristics, selection rationale, and the steps taken to ensure comprehensiveness of literature capture.<br><br>Briefly, the CAL® tool uses a logistic regression algorithm that continuously re-ranks unscreened literature from most to least likely relevant based on relevance

	literature. The authors note this tool has been used in prior systematic reviews, yet indicate additional literature review identified additional studies the CAP tool did not (e.g., 22 studies KQ4/T Theme 2 in the supplement flow diagram).	decisions made by a reviewer, ensuring that the most likely relevant studies are always prioritised for review. The tool has been validated across seven international high-recall information retrieval competitions and has been used to expedite literature screening in systematic reviews. To further support high recall, multiple screening sessions using different seed (i.e., starting) studies were conducted for each key question. Consistent with recommended practice for active learning-based tools, screening continued until 100 sequential references were excluded or there was consensus among the review team that no further relevant studies were being identified.<br><br>As an additional precautionary measure to ensure comprehensiveness, the included reference lists any relevant systematic review identified during searching were screened. One such review was identified during CAL® searching for the KQ4/KQ7. Upon full-text review, none of the 22 included studies within this literature review met the eligibility criteria for inclusion. This is consistent with the expected operation of a high-recall screening tool, where supplementary searches may identify additional records that ultimately do not meet inclusion criteria.<br><br>These details have been incorporated into the revised methods section.
Reviewer #6	- Further clarify the training and validation steps for the CAP Tool. Appendix 3 provides a figure of the CAP Tool supervised training; however, it is unclear how performance of the training and validation groups were evaluated as this figure suggests this is a continuous training loop.	We appreciate the opportunity to clarify how performance was monitored within the continuous active learning framework.<br><br>As the reviewer correctly notes, CAL® operates as a continuous training loop: with each relevance decision made by a reviewer, the logistic regression algorithm is retrained and the remaining unscreened literature is re-ranked. In this framework, there is no discrete training and validation phase in the traditional supervised learning sense. Instead, performance is evaluated implicitly through the yield of relevant studies surfaced across successive screening sessions.<br><br>Briefly, seed studies (known relevant articles identified a priori) were used to initialize the algorithm for each key question. Multiple

		screening sessions with different seeds were conducted to reduce dependence on any single starting point and to support high recall. Performance was monitored throughout by tracking the rate at which relevant studies continued to be identified; screening was stopped when 100 sequential references were excluded or when the review team reached consensus that no further relevant studies were being suggested, consistent with recommended stopping criteria for active learning-based tools.<br><br>We acknowledge that formal quantitative validation metrics (e.g., recall or precision at stopping) were not available, and we have noted this in our limitations section.
Reviewer #6	- Provide a statement summarizing the publications not available after the 14-day window or note absence of accessible PDFs as a limitation. The authors appropriately list all reasons for exclusion of publications for each KQ and there are a number that were excluded due to "PDF not available", "PDF unavailable", or "full text unavailable" with titles that were seemingly highly relevant.	We thank the reviewer for this suggestion. Having reviewed the excluded studies lists across all key questions, we identified 61 unique studies excluded due to full-text unavailability. We have added a statement to the limitations section acknowledging this, noting the potential relevance of some of these studies and the constraints imposed by the 14-day retrieval window inherent to rapid review methodology. We also note that a proportion of the unavailable studies were conference abstracts, for which full-text retrieval is inherently limited regardless of the retrieval timeframe, and have reflected this distinction in the limitations statement
Reviewer #6	- Clarify in the Methods if English-only texts were included. The methods indicate the literature search included text of any language, yet many non-English studies were excluded in the supplemental flow diagrams.	We thank the reviewer for flagging this. To clarify, no language restrictions were applied at the search stage; however, non-English studies were excluded at the full-text review stage due to resource limitations in accessing translation services within the timeframe of the rapid review. We have revised the methods section to accurately reflect this, and added a brief note to the limitations section acknowledging that the exclusion of non-English full texts is a limitation of the current review.
Reviewer #7	This series of rapid reviews describes the methods and outcomes of a process to define the evidence (or lack of) relating to IPC guidelines for Ebola and Marburg disease. The evidence synthesis is useful, primary through documenting the paucity of	We thank Reviewer 3 for their thoughtful summary of our manuscript and for recognizing the value of this work in documenting the current state of evidence for IPC practices in EBOD and MARD, identifying critical knowledge gaps, and informing WHO guideline development.

	evidence in this area, and the identification of significant gaps in knowledge which should be targeted by future research, which is highlighted in the Discussion. Nonetheless the review has identified data which has contributed to the development of guidelines. The limitations of the study are discussed and relate mainly to the lack of evidence, the narrow eligibility criteria of the Key Questions, and uncertainties around the rapid review methodology, including the use of AI for the data search.	

VERSION 4 - REVIEW

**Reviewer**	**2**
**Name**	**Torgersen, Jessie**
**Affiliation**	**University of Pennsylvania, Medicine**
**Date**	**25-Jun-2026**
**COI**	

The authors have appropriately addressed all concerns in the revised manuscript.
